# Cross‐Dehydrogenative Coupling Reactions With Redox‐Active Guanidine Reagents

**DOI:** 10.1002/chem.71329

**Published:** 2026-07-03

**Authors:** Petra Walter, Olaf Hübner, Hans‐Jörg Himmel

**Affiliations:** ^1^ Anorganisch‐Chemisches Institut Ruprecht‐Karls‐Universität Heidelberg Heidelberg Germany

## Abstract

Cross‐dehydrogenative coupling (CDC) reactions are attractive and environmentally friendly ways to form new C‐C and other element‐element bonds. Here, we report the first CDC reactions triggered by a redox‐active guanidine reagent. *N*‐phenyltetrahydroisoquinoline and derivatives with electron‐donating and withdrawing substituents at the *N*‐phenyl group are reacted with nitromethane, dimethylmalonate and methylphenyl ketone in the presence of oxidized, dicationic 1,4‐bis(tetramethylguanidino)benzene as redox‐active guanidine reagent. The CDC products are obtained in good yield. The diprotonated 1,4‐bis(tetramethylguanidino)benzene by‐product can be easily separated from the product, and subsequently recycled through oxidation to the CDC reagent. The reaction mechanisms are discussed, and compared to the corresponding reactions with the high‐potential quinone 2,3‐dichloro‐5,6‐dicyano‐1,4‐benzoquinone (DDQ). The analysis discloses important advantages of redox‐active guanidine reagents compared to quinone reagents such as DDQ, arising from shorter lifetimes of radical intermediates.

## Graphical abstract

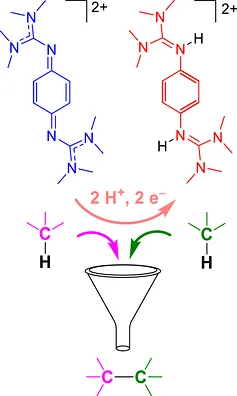

## 1 Introduction

Cross‐dehydrogenative coupling (CDC) reactions are of immense importance since they obey to modern efficient, green and waste‐reducing bond‐forming strategies [1, 2, 3, 4, 5, 6, 7]. A general, schematic equation for the formation of new carbon‐element bonds with CDC reactions is formulated in Scheme 1a. In this equation, two protons and two electrons are formed as co‐products to the desired CDC product. Consequently, a CDC reagent is needed that takes up these two protons and two electrons. The reactions are special proton‐coupled electron transfer (PCET) processes, that are extremely important both in synthetic chemistry and in biology [8, 9, 10, 11, 12]. Several CDC reagents and catalysts were reported in recent years, and thermally as well as photo‐induced reaction protocols were developed [1, 2].

**SCHEME 1:**
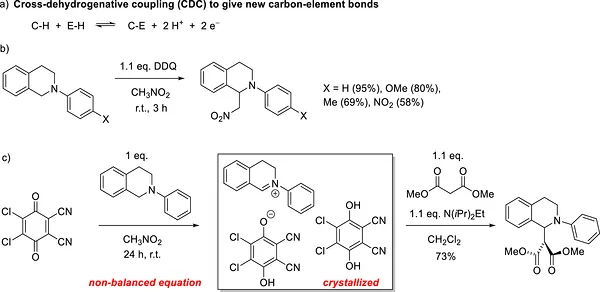
a) General equation for a cross‐dehydrogenative coupling reaction. b) Reaction between *N*‐phenyltetrahydroisoquinolines and nitromethane with DDQ as electron and proton acceptor. c) Isolation of an iminium salt and its reaction with dimethyl malonate, avoiding direct contact between DDQ and dimethyl malonate.

Within this research theme, Todd and coworkers reported reactions between *N*‐phenyltetrahydroisoquinolines and nitromethane, enabled by 2,3‐dichloro‐5,6‐dicyano‐1,4‐benzoquinone (DDQ) as CDC reagent (Scheme 1b) [13, 14]. The effect of an electron‐donating (OMe) and an electron‐withdrawing (NO_2_) substituent at the *N*‐phenyl group on the yield was also evaluated. The highest CDC yield was obtained without substituent (X = H), and the lowest yield for X = NO_2_. It was suggested that DDQ first oxidizes the *N*‐phenyltetrahydroisoquinoline to the corresponding iminium ion, that subsequently reacts with nitromethane to the CDC product. In a follow‐up publication, the authors showed that the analogue, direct reaction between *N*‐phenyltetrahydroisoquinoline and dimethylmalonate with DDQ failed to give the CDC product. The authors suggested the direct oxidation of dimethylmalonate by DDQ as a possible reason. According to this scenario, oxidation of dimethylmalonate leads initially to a reactive malonyl radical through H atom abstraction, that subsequently reacts unselectively to a complex product mixture. (The results obtained in this work do not support this scenario, see discussion below.) Todd and coworkers isolated and structurally characterized an iminium ion formed by reaction between DDQ and *N*‐phenyltetrahydroisoquinoline. Reaction of this iminium ion and dimethylmalonate eventually gave the CDC product (Scheme 1b) [14]. The results indicated that the iminium ion is an intermediate in the course of CDC reactions with *N*‐phenyltetrahydroisoquinoline. Later, Su and coworkers developed solvent‐free CDC reactions under ball‐milling conditions for the synthesis of functionalized tetrahydroisoquinolines mediated by DDQ [15].

It should be mentioned at this place that several groups studied the CDC reactivity of *N*‐phenyltetrahydroisoquinolines, due to the importance of such reactivity in synthetic chemistry (see, for example, reference 5 for a comprehensive discussion of CDC‐type aza‐Henry reactions). Antonchick et al. used hypervalent iodine reagents (e.g., PhI(OCOCF_3_)_2_) for the direct acylation of *N*‐phenyltetrahydroisoquinolines with aldehydes in the presence of TMSN_3_ as additive [16]. However, a drawback of this protocol is the requirement to apply two equivalents of the azide additive (relative to the *N*‐phenyltetrahydroisoquinoline reactant) to obtain a good yield. In photo‐catalytic reactions, air or dioxygen were used as oxidant. Hence, Blechert and coworkers reported metal‐free carbon nitride‐catalysed photoredox reactions of *N*‐phenyltetrahydroisoquinolines with nitromethane, dimethylmalonate and ketones (e.g. methylphenyl ketone) to the corresponding CDC products [17]. Sharma and coworkers prepared a ruthenium‐based nano‐sized photoredox catalyst that was used in the reactions of *N*‐phenyltetrahydroisoquinolines with methylarylketones and nitroalkanes [18]. Recently, an eosin‐based heterogeneous photocatalyst was reported for the room temperature CDC reaction between *N*‐phenyltetrahydroisoquinolines and nitroalkanes (nitromethane and nitropropane) [19]. Although such photocatalytic reactions are highly attractive, there are some major drawbacks. The preparation of the heterogeneous catalyst is generally a quite complex process, the scope of the reactions is limited, and the long‐term stability of the nanosized heterogeneous catalysts is at least questionable.

High‐potential quinones such as DDQ have been developed into powerful reagents for CDC chemistry [1, 2, 3, 4, 5, 6, 7, 20]. However, the accumulation of radical intermediates in the course of the reactions can lead to unselective reactions. In this context, a recent seminal study on CDC reactions between alkenes and diarylmethanes, mediated by DDQ, should be mentioned [21]. The use of 1,2‘‐diphenylethylene as the alkene component led to a modest yield (50%) of the CDC product, and the use of (*E*)‐buta‐1,3‐dienylbenzene gave complex product mixtures, indicating that the low stability of the radicals formed in the initial electron‐transfer step between the alkene and DDQ is responsible for unselective side‐reactions. Based on these considerations, the alkenes were modified with two dialkylthio groups that stabilize the radical cation due to their strong electron‐donating effect. Indeed, highly efficient CDC reactions were developed in this way, providing convenient access to 1*H*‐indenes.

Our group developed guanidino‐functionalized aromatics (GFAs) as a new class of compounds for dehydrogenative coupling reactions and proton‐coupled electron‐transfer (PCET) processes, representing readily available, environmentally friendly alternatives to DDQ and other high‐potential quinones [22, 23]. We reported the synthesis of GFAs such as 1,4‐bis(tetramethylguanidino)benzene (GFA1, Figure 1) [24] and 1,2,4,5‐tetrakis(tetramethylguanidino)benzene (GFA2) [25], their oxidation to stable salts of the dications, (GFA1)^2+^ and (GFA2)^2+^, and their use as potent reagents for a variety of dehydrogenative (C─C, C─O and P─P) coupling reactions [26, 27, 28, 29, 30, 31, 32]. The reduction potential of (GFA1)^2+^ (*E*_1/2_ = −0.21 V vs. Fc^+^/Fc in CH_2_Cl_2_ for the redox couple (GFA1)^2+^/GFA1)) is much higher than that of (GFA2)^2+^ (*E*_1/2_ = −0.70 V vs. Fc^+^/Fc in CH_2_Cl_2_ for the redox couple (GFA2)^2+^/GFA2) [33]). Since the difference in the Brønsted basicity is relatively small, (GFA1)^2+^ is a more potent PCET reagent than (GFA2)^2+^. On the other hand, the reduced, protonated form (GFA2+2H)^2+^, that is formed in the course of the PCET reactions, can be easily recycled to (GFA2)^2+^ by catalytic oxidation with dioxygen, rendering the use of (GFA2)^2+^ particularly attractive [34]. So far, we have tested the performance of (GFA1)^2+^, (GFA2)^2+^ and related oxidized redox‐active guanidines in reactions involving only one substrate type. Two examples for efficient intra‐ and intermolecular coupling reactions (with the substrates 2‐[(4‐methoxyphenyl)methyl]‐benzoic acid and naphthalene‐1,2‐dione) are included in Figure 1b [28]. The addition of an acid (HBF_4_·OEt_2_ or methanesulfonic acid) typically accelerates the reactions.

**FIGURE 1:**
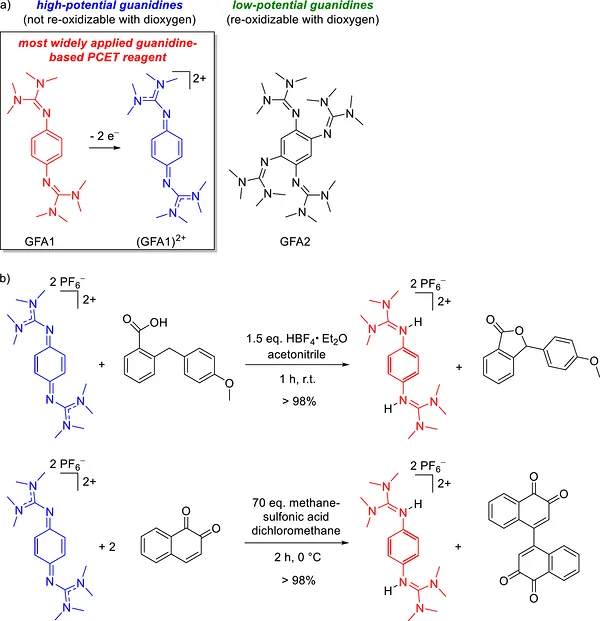
a) Comparison between a typical high‐ and low‐potential redox‐active guanidine. b) Representative intra‐ and intermolecular dehydrogenative coupling reactions with (GFA1)^2+^.

In this work, we show for the first time that (GFA1)^2+^ is a versatile reagent for CDC reactions, clearly outperforming DDQ in some CDC reactions of *N*‐phenyltetrahydroisoquinoline and derivatives. The reasons for the better performance of the redox‐active guanidine reagent (GFA1)^2+^ compared to the high‐potential quinone DDQ are disclosed by an analysis of the likely mechanism. Reactions with (GFA1)^2+^ lead to rapid transfer of two electrons, thereby avoiding long lifetimes of radical intermediates that give rise to the unselective formation of undesired side‐products. The analysis shows that redox‐active guanidines are more reliable CDC reagents than high‐potential quinones.

## 2 Results and Discussion

In our experiments, we used *N*‐phenyltetrahydroisoquinoline (PTQ) and the derivatives 2‐[4‐(trifluoromethyl)phenyl]‐1,2,3,4‐tetrahydroisoquinoline (PTQ‐CF_3_), 2‐[4‐(acetyl)phenyl]‐1,2,3,4‐tetrahydroisoquinoline (PTQ‐COMe), 2‐[4‐(methyl)phenyl]‐1,2,3,4‐tetrahydroisoquinoline (PTQ‐Me) and 2‐(4‐methoxyphenyl)‐1,2,3,4‐tetrahydroisoquinoline (PTQ‐OMe) [35] to test the influence of electron‐withdrawing and donating substituents on the reaction outcome and the kinetics. The tetrahydroisoquinolines were reacted with three reaction partners, namely nitromethane, dimethylmalonate, and methylphenyl ketone, in the presence of the salt GFA1(PF_6_)_2_ as CDC reagent. In some reactions, GFA1(PF_6_)_2_ was replaced by DDQ to study the reactivity differences. After completion of the reactions, a work‐up procedure was executed to separate the protonated, reduced CDC reagent (GFA1+2H)(PF_6_)_2_. This procedure was based on the low solubility of (GFA1+2H)(PF_6_)_2_ in CH_2_Cl_2_ or CHCl_3_. Hence, addition of CH_2_Cl_2_ or CHCl_3_ to the solid product and filtration was sufficient to separate (GFA1+2H)(PF_6_)_2_. For recycling of (GFA1+2H)(PF_6_)_2_ to the CDC reagent GFA1(PF_6_)_2_, it was subsequently dissolved in H_2_O, NaOH (25%) was added, and the solution was repeatedly extracted with CH_2_Cl_2_. The combined organic phases were dried over K_2_CO_3_, filtrated and condensed to yield pure neutral GFA1, that was transformed back to the CDC reagent GFA1(PF_6_)_2_ by oxidation (see Supporting Information). In most cases, the product was isolated after removal of the solvent, and the yield was determined by weighing (last column in Table 1). In some reactions, the yield was determined directly after the work‐up procedure by NMR spectroscopy. The results of all reactions are summarized in Table 1.

**TABLE 1:** CDC reactions of *N*‐phenyltetrahydroisoquinolines with nitromethane, dimethylmalonate or methylphenyl ketone, using GFA1(PF_6_)_2_ as CDC reagent (1‐2 equivalents, see Supporting Information).

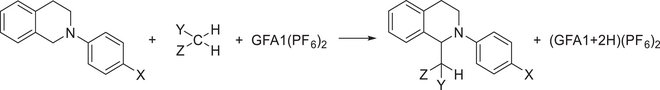						
Entry	X	Y	Z		Yield (NMR %)^a^	Yield (weighing %)^a^
1	H	H	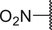	3 h, r.t.	93	91
2	Me	H	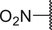	2 h, r.t.		95
3	OMe	H	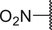	10 min, r.t.	96	
4	OMe	H	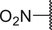	2 h, r.t.		85
5	CF_3_	H	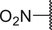	12 h, r.t.	0	
6	CF_3_	H	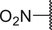	12 h, 50°C	>99	93^c^
7	CF_3_	H	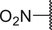	20 h, 50°C		78
8	C(O)Me	H	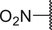	20 h, r.t.	66%	
9	C(O)Me	H	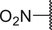	5 h, 50°C		98
10	H	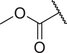	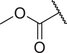	20 h, 50°C^b^		81
11	H	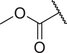	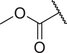	CD_3_CN 9 h, r.t.		55
12	OMe	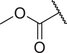	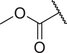	20 h, 50°C^b^		82
13	OMe	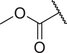	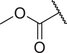	in CD_3_CN 1 h, r.t.		79
14	CF_3_	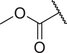	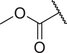	20 h, 50°C^b^	60	
15	CF_3_	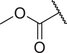	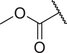	45 h, 50°C^b^		88
16	CF_3_	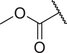	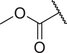	in CD_3_CN 20 h, r.t.	17	
17	CF_3_	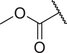	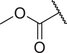	in CD_3_CN 20 h, 50°C		67
18	H	H	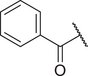	4 h, 50°C		68
19	OMe	H	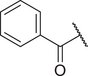	6 h, r.t.		69

^a^ After separation from the (GFA+2H)(PF_6_)_2_ by‐product.

^b^ Higher temperatures necessary due to low solubility of GFA1(PF_6_)_2_ in neat dimethylmalonate.

^c^ Small‐scale reaction (18.3 µmol of PTQ‐CF_3_). The difference in yield with respect to the reaction in entry 7 is due to the work‐up procedure (see text).

The reactions of *N*‐phenyltetrahydroisoquinolines with CH_3_NO_2_ to give 1‐nitromethyl‐2‐phenyl‐1,2,3,4‐tetrahydroisoquinoline (Scheme 2) were carried out in neat CH_3_NO_2_, using one equivalent of GFA1(PF_6_)_2_. The reactions with PTQ, PTQ‐Me and PTQ‐OMe proceeded at room temperature in high yield (Scheme 2 and entries 1–4 in Table 1). In the case of PTQ‐CF_3_ with an electron‐withdrawing CF_3_ substituent at the phenyl ring, reaction was extremely slow at room temperature (entry 5 in Table 1). Therefore, the reaction mixture was heated to a temperature of 50°C (Table 1, entries 6 and 7). Under these conditions, the product was obtained in 93% yield (>99% according to the NMR spectra recorded for a solution directly after removal of the (GFA1+2H)(PF_6_)_2_ by‐product) for reaction on a small scale (18.3 µmol of PTQ‐CF_3_), and 78% yield for a larger batch. The differences in yield are caused by the work‐up procedure to separate the redox‐active guanidine. In the reaction in entry 7, a larger amount of CH_2_Cl_2_ was required to remove the (GFA1+2H)(PF_6_)_2_ by‐product, leading to some loss of product. Finally, reaction of PTQ‐COMe also proceeded very slowly at room temperature; a reaction time of 20 h was necessary to obtain 66% yield (Table 1, entry 8). On the other hand, reaction at 50°C yielded the CDC product in 98% yield after 5 h reaction time (Table 1, entry 9).

**SCHEME 2:**
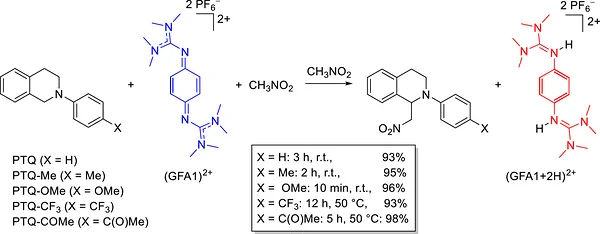
CDC reactions between *N*‐phenyltetrahydroisoquinolines and CH_3_NO_2_, accomplished with (GFA1)^2+^ as CDC reagent, and their yields (see the table for further reactions).

In Figure 2, the ^1^H NMR spectra of PTQ‐CF_3_, (GFA1)^2+^ and the isolated CDC product 1‐(nitromethyl)‐2‐[4‐(trifluoromethyl)phenyl‐1,2,3,4‐tetrahydroisoquinoline are compared, confirming the clean conversion of the CDC product (see Supporting Information for the assignment of the NMR signals). This reaction, with an electron‐withdrawing CF_3_ substituent at the phenyl group of the *N*‐phenyltetrahydroisoquinoline reagent, was not yet reported for DDQ. Therefore, we repeated the reaction under similar conditions (neat CH_3_NO_2_, 20 h at 50°C) with DDQ in place of (GFA1)^2+^. A complex product mixture was obtained for the direct reaction between PTQ‐CF_3_ and CH_3_NO_2_ with DDQ as CDC reagent (see Supporting Information for details), from which the CDC product cannot be isolated. Hence, (GFA1)^2+^ clearly outperforms DDQ in this reaction. The likely mechanism and an explanation of the differences between reactions with (GFA1)^2+^ and DDQ are presented below.

**FIGURE 2:**
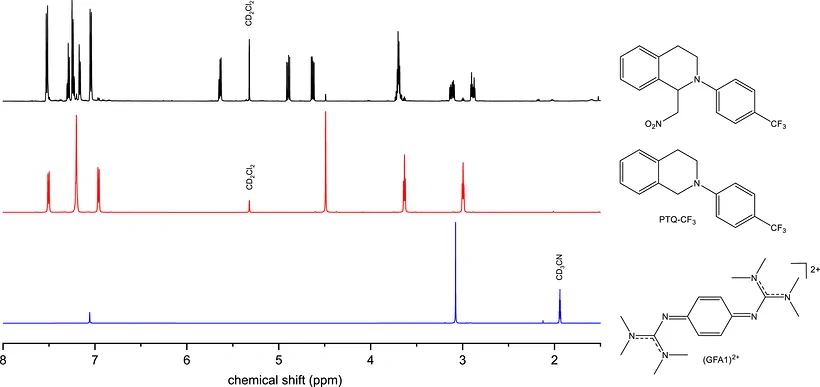
Comparison between the ^1^H NMR spectra of the diguanidine reagent (GFA1)^2+^ (in CD_3_CN), as well as the substrate PTQ‐CF_3_ and the CDC product 1‐(nitromethyl)‐2‐[4‐(trifluoromethyl)phenyl‐1,2,3,4‐tetrahydro‐isoquinoline after isolation (both in CD_2_Cl_2_). Please note that an analogue reaction with DDQ in place of (GFA1)^2+^ fails to give the CDC product.

Next, we studied the CDC reactions between *N*‐phenyltetrahydroisoquinolines and dimethylmalonate (Scheme 3 and entries 10–17 in Table 1). The reactions were carried out either in neat dimethylmalonate or in acetonitrile. The reactions in neat dimethylmalonate were carried out at higher temperature (50°C), due to the low solubility of GFA1(PF_6_)_2_ in dimethylmalonate at room temperature. Under these conditions, the CDC products were selectively formed in yields higher than 80% (entries 10, 12, 14 and 15 in Table 1). The highest yield (88%) was obtained for PTQ‐CF_3_ (neat dimethylmalonate, 40 h at 50°C, entry 15). Due to the high solubility of GFA1(PF_6_)_2_ in acetonitrile, the reactions in this solvent can be carried out at room temperature. In the case of PTQ‐OMe, a yield of 79% was obtained already after 1 h reaction time at room temperature (entry 13 in Table 1). The reaction time needs to be increased in the case of PTQ (X = H); 55% yield was achieved after 9 h at room temperature (entry 11). Finally, for PTQ‐CF_3_ (X = CF_3_), only 17% CDC product was obtained after 20 h at room temperature (entry 16). Therefore, the reaction was repeated at a higher reaction temperature of 50°C, yielding 67% CDC product after 20 h reaction time (entry 17). In line with the previous work by Todd and coworkers [14], the reactions with DDQ in place of (GFA1)^2+^ led to complex product mixtures, from which it was impossible to isolate pure CDC products (see Supporting Information).

**SCHEME 3:**
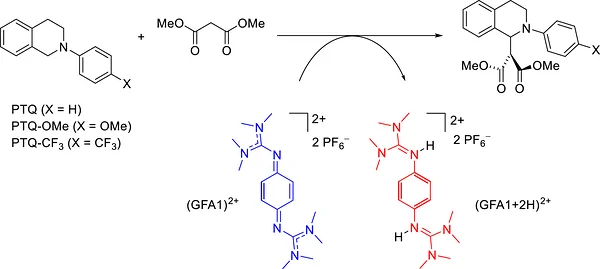
CDC reactions between *N*‐phenyltetrahydroisoquinolines and dimethylmalonate, with (GFA1)^2+^ as CDC reagent (see the table and the Supporting Information for more details and yields).

Finally, we tested the CDC reactions between one of the *N*‐phenyltetrahydroisoquinolines and methylphenyl ketone (Scheme 4 and entries 18 and 19 in Table 1). The reaction of PTQ‐OMe with methylphenyl ketone gave the desired CDC product in 69% yield after a reaction time of 6 h at room temperature (entry 19). The reaction with PTQ (X = H) required a temperature of 50°C; a yield of 68% was obtained after 20 h at room temperature (entry 18). Such reactions are completely unknown for DDQ.

**SCHEME 4:**
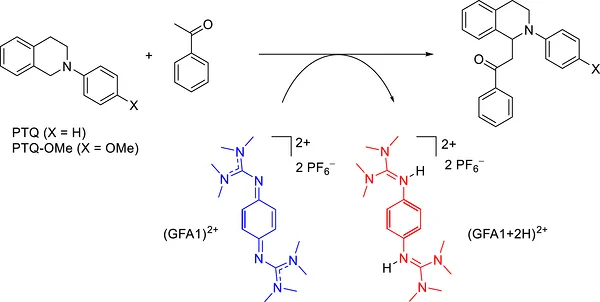
CDC reactions between *N*‐phenyltetrahydroisoquinolines and methylphenyl ketone, with (GFA1)^2+^ as CDC reagent (see the table and the Supporting Information for more details and yields).

### 2.1 Thermodynamic and Mechanistical Considerations

First, to verify that the CDC reactions with nitromethane are exothermic for all three different PTQ variants (X = H, CF_3_, OMe), we carried out quantum‐chemical calculations (B3LYP+D3/def2‐TZVP, see the Supporting Information). The calculations considered different configurational and conformational isomers, and the calculated reaction energies rely on the lowest‐energy isomers. The minimum energy structures are shown in Figure 3a. According to the calculations, for the CDC reactions with PTQ, PTQ‐CF_3_, and PTQ‐OMe, the Gibbs free energies at 298 K amount to −140, −137, and −140 kJ mol^−1^, respectively. Since the reaction converts three molecules into two molecules, the reaction entropy is negative. Thus, the reaction enthalpies at 298 K with values of −209, −208, and −208 kJ mol^−1^, respectively, are more negative than the corresponding free reaction enthalpies. The calculations indicate the reactions to proceed spontaneously for all reactions.

**FIGURE 3:**
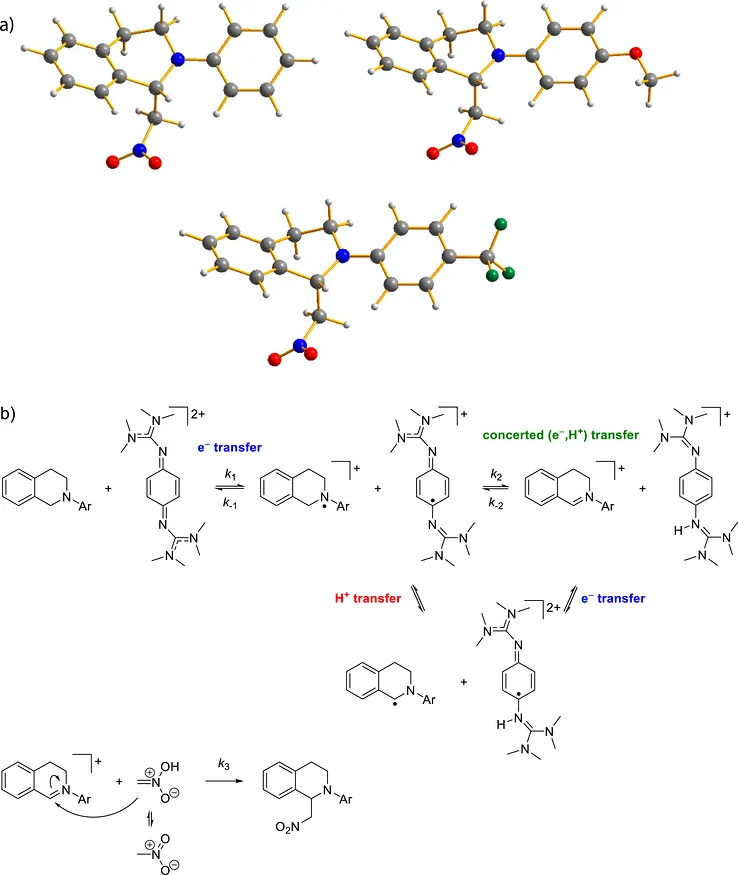
a) Energy‐minimum structures of the products of reaction with nitromethane. b) Possible pathway of the reaction between an *N*‐phenyltetrahydroisoquinoline and (GFA1)^2+^ in nitromethane to give 1‐nitromethyl‐2‐phenyl‐1,2,3,4‐tetrahydroisoquinoline.

A possible pathway for the CDC reactions between *N*‐phenyltetrahydroisoquinoline and nitromethane with GFA1(PF_6_)_2_ as CDC reagent is shown in Figure 3b. Almost certainly, the CDC reagent oxidizes *N*‐phenyltetrahydroisoquinoline to the corresponding iminium ion. Two electrons and one proton are transferred from *N*‐phenyltetrahydroisoquinoline to (GFA1)^2+^. It is unlikely that such a complex process occurs in a concerted way in one mechanistical step. It is rather assumed that iminium ion formation is a two or three step process. The basicity of (GFA1)^2+^ is far too low for significant PTQ deprotonation. Therefore, the reactions are most likely initiated by an electron transfer step to give an amine radical cation (nitrogen‐centered radical). This reversible electron transfer step exhibits a rate constant *k*_1_ for the forward direction, and another rate constant *k*_−1_ for the reverse direction (Figure 3b).

From this amine radical cation intermediate, the iminium ion is formed either in a concerted proton‐coupled electron transfer (PCET) step, or in a two‐step process (proton transfer followed by electron transfer), with a carbon‐centered radical as intermediate (Figure 3b). The formation of the iminium ion from the amine radical cation and the reverse reaction are associated with the two rate constants *k*_2_ and *k*_−2_. The iminium ion seems to be a relatively stable intermediate, because it can be isolated [14]. Finally, the addition of the second substrate (nitromethane, dimethylmalonate or methylphenyl ketone) to the iminium ion and tautomerisation yields the final CDC product. In Figure 3b, only the reaction with nitromethane is shown, but similar reaction pathways can be formulated with dimethylmalonate and methylphenyl ketone.

To test the hypothesis of a preliminary electron transfer step, we compared the redox potentials of the three *N*‐phenyltetrahydroisoquinolines with that of GFA1. Cyclic voltammetry (CV) curves were recorded for the five substrates PTQ, PTQ‐OMe, PTQ‐Me, PTQ‐COMe, and PTQ‐CF_3_ in CH_2_Cl_2_ solutions (Figure 4). The first oxidation wave is clearly visible; the absence of a corresponding reduction wave signals irreversible oxidation. The oxidation potentials *E*_ox_ measure 0.26 V for PTQ‐OMe, 0.49 V for PTQ, and 0.82 V for PTQ‐CF_3_ (all values with respect to the reference redox couple Fc^+^/Fc), showing a clear trend to higher potentials with increasing electron‐withdrawing character of the substituent. The position of the initiating electron‐transfer equilibrium depends on the difference Δ*E* between the oxidation potential of the substrate and the reduction potential of the GFA (*E*_red_ = −0.32 V for (GFA1)^2+^) [27]; this difference should not significantly exceed the assumed limit for conventional electron transfer at standard conditions of ca. 1 V (96.5 kJ mol^−1^) [36]. For PTQ, PTQ‐Me and PTQ‐OMe, the Δ*E* value is well within the acceptable range (Δ*E* = 0.81, 0.72 and 0.58 V, respectively). On the other hand, for PTQ‐CF_3_, the Δ*E* value of 1.14 V already exceeds slightly the rough limit of Δ*E* = 1 V for conventional electron transfer at ambient conditions. In the case of PTQ‐COMe, the Δ*E* value (1.04 V) is close to this limit. Indeed, the experiments show that the reactions of PTQ‐CF_3_ and PTQ‐COMe are very slow at room temperature (entries 5 and 8 in Table 1). A temperature of 50°C is necessary to achieve good CDC yields (entries 6 and 9). Hence, the experimental results support the mechanistical picture sketched in Figure 3b.

**FIGURE 4:**
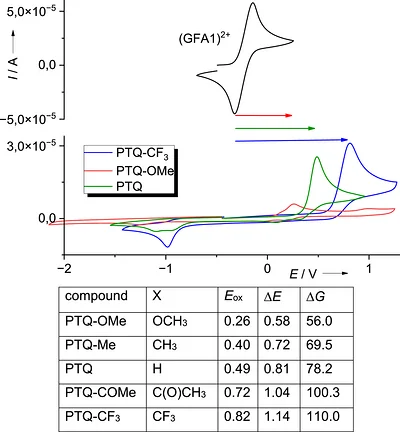
Comparison between the cyclic voltammetry (CV) curves for *N*‐phenyltetrahydroisoquinoline, *N*‐(4‐methoxyphenyl)tetrahydroisoquinoline, and *N*‐(4‐trifluoromethylphenyl)tetrahydroisoquinoline (see Supporting Information for CV curves of the other two PTQ derivatives). Potentials given vs. the reference redox couple ferrocenium/ferrocene (Fc^+^/Fc). The arrows highlight the potential difference between the potential for reduction of (GFA1)^2+^ and the potential for oxidation of the *N*‐phenyltetrahydroisoquinoline derivative. A table with the *E*_ox_, Δ*E*, and Δ*G* values is included.

Due to the relatively large potential differences Δ*E* between the *E*_ox_ value of the (substituted) *N*‐phenyltetrahydroisoquinolines and the *E*_red_ value of (GFA1)^2+^, only a very small amount of the amine radical is formed. The Δ*G* values for the electron transfer steps at room temperature (Δ*G* ≈ F·Δ*E*) vary between 56 kJ mol^−1^ (for X = OCH_3_) and 110.0 kJ mol^−1^ (for X = CF_3_).

Similar to the results obtained with DDQ by Todd and coworkers (references 8 and 9), radical trapping experiments with TEMPO did not lead to significant changes. The radicals, formed in a small amount in the electron‐transfer equilibrium, are quite rapidly consumed in the subsequent proton‐ and electron‐transfer steps. The reaction partner (GFA1)^2+^, to which the electron is transferred from PTQ in the first, radical‐forming process, directly reacts further with the PTQ radical cation, leading to transfer of a proton and an electron to give the diamagnetic iminium ion intermediate. Presumably, the reaction partners remain in the same solvent cage, hampering reactions of the PTQ radical cation with a radical trapping reagent.

To obtain additional information, we studied the influence of electron‐donating and withdrawing substituents at the *N*‐phenyltetrahydroisoquinoline on the reaction kinetics in more detail. In Figure 5a, the conversion to the CDC product from ^1^H NMR spectra with time is plotted for the PTQ derivatives in neat CD_3_NO_2_. The fastest reaction was observed for PTQ‐OMe; quantitative conversion at room temperature was achieved within 10 min. It can be seen that the reaction proceeds slower with PTQ‐Me (98% after 44 min) and PTQ (93% after 3 h) at room temperature. In the case of PTQ‐COMe and PTQ‐CF_3_, the reactions were very slow at room temperature. Therefore, the conversion was followed at 50°C (Figure 5a). Even at 50°C, the reactions were slower than with the other three tetrahydroisoquinolines at room temperature. Quantitative CDC product formation was observed after 5 h at 50°C for PTQ‐COMe and after 12 h at 50°C for PTQ‐CF_3_. These results indicate that the rate constant of the initial electron transfer step contributes crucially to the overall rate. The ratio *k*_el_/*k*_‐el_ increases with decreasing difference between the *E*_ox_ value of the *N*‐phenyltetrahydroisoquinoline and the *E*_red_ value of (GFA1)^2+^. A larger value of the equilibrium constant for the electron transfer step leads to faster conversion.

**FIGURE 5:**
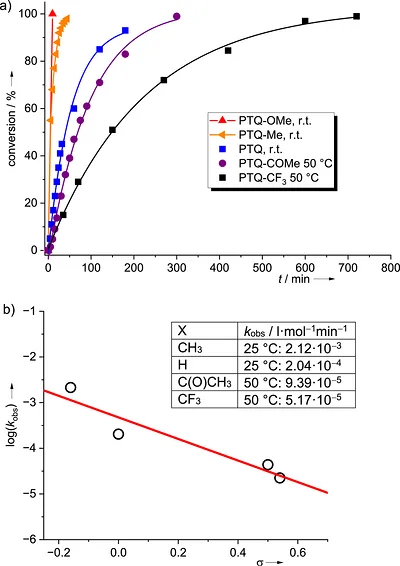
a) Conversion as a function of reaction time for the reaction of differently‐substituted *N*‐phenyltetrahydroisoquinolines with nitromethane in the presence of GFA1(PF_6_)_2_. b) Hammett plot on the basis of the second‐order rate constants *k*_obs_ estimated from the conversion vs. time plots.

On the assumption of a second‐order rate law, the rate constants *k*_obs_ at room temperature (for PTQ‐Me and PTQ) or 50°C (for PTQ‐COMe and PTQ‐CF_3_) were estimated from the conversion vs. time plots, yielding the values included in the table in Figure 5b. For the reactions with PTQ‐COMe and PTQ‐CF_3_, the *k*_obs_ values at 25°C were estimated from the *k*_obs_ values at 50°C by using the simple relationship ln(*k*_obs_ at 25°C) ≈ ln(*k*_obs_ at 50°C)·(323/298). A rough Hammett plot (log(*k*_obs_) (*k*_obs_ dimensionless) as a function of the Hammett constants σ) yielded a slope of ρ = −2.4 ± 0.5 (Figure 5b). The sign of the substituent parameter ρ is opposite to that in the reference Hammett relationship for the ionization of benzoic acid (ρ = +1), since electron‐withdrawing substituents slow down the CDC reaction. Moreover, the sensitivity to substitution is relatively large (see, for example, the discussion of the ρ parameter in references 37 and 38). This result can be rationalized by the development of positive charge on the nitrogen atom directly attached to the phenyl group in the transition state of the formation of the cationic amine radical (one‐electron oxidation of the tetrahydroisoquinolines by (GFA1)^2+^ in the first, rate‐determining step of the proposed mechanism (see Figure 3b).

Reactions with DDQ most likely follow a similar mechanism, being in line with the results reported by Todd et al. [13, 14]. Moreover, Kochi and coworkers isolated several anion radical salts of DDQ that resulted from an initial one‐electron transfer from a donor to DDQ [39]. Todd and coworkers were not able to detect radical intermediates in the reaction between *N*‐phenyltetrahydroisoquinoline and nitromethane. Nevertheless, radicals are most likely involved and were postulated in the mechanism [13, 14].

While the use of (GFA1)^2+^ as CDC reagent led to clean, selective formation of the CDC products in high yields, the replacement of (GFA1)(PF_6_)_2_ by DDQ leads to unselective reactions resulting in complex product mixtures for some of the reactions studied herein. What is the reason for this reactivity difference between DDQ and (GFA1)^2+ ^? In the case of reaction with dimethylmalonate, Todd and coworkers suggested a possible direct reaction between dimethylmalonate and DDQ as an explanation for the unselective reaction. To check this possibility, we added DDQ to neat dimethylmalonate and recorded UV‐vis spectra with time (see Supporting Information). The absorption at 372 nm typical for DDQ [39] increased with time, due to the slow solvation of DDQ in dimethylmalonate, but no other bands appeared, indicating that DDQ does not react with dimethylmalonate. Therefore, the explanation offered by Todd and coworkers is not in accordance with the experimental data.

To explain the better performance of (GFA1)^2+^ compared with DDQ, we inspected in more detail the initial electron‐transfer step (Figure 3b). We first compared the redox properties of (GFA1)^2+^ and DDQ. A relatively high reduction potential for one‐electron reduction of *E*_1/2_ = +0.14 V was reported for DDQ/DDQ**^·^** ^−^ (in CH_3_CN solution with respect to Fc^+^/Fc) [40]. This potential is significantly higher than that of (GFA1)^2+^ (*E*_1/2_ = −0.21 V in CH_2_Cl_2_ solution). It is also higher than that of other important quinone reagents such as chloranil (CA) with −0.36 V (or −0.34 V) [41] for CA/A**^·^** ^−^. On the other hand, the reduction potential for the second electron transfer step is much lower; a value of *E*_1/2_ = −0.67 V was reported for the redox couple DDQ**^·^** ^−^/DDQ^2−^ [40]. In the case of (GFA1)^2+^, the first and the second one‐electron reduction exhibit similar potentials (*E*_1/2_ = −0.21 V for (GFA1)^2+^/GFA1). This has two important consequences.

1) In the case of two‐electron processes, like the reactions studied herein, the thermodynamics of electron transfer correlates with the sum of the two one‐electron potentials. The sum of the first and second one‐electron reduction potentials of DDQ (+0.14 + (−0.67) = −0.53 V) is much higher than the sum of the first and second one‐electron reduction potentials of CA (−0.36 +(−1.08) = −1.44 V) [42]. On the other hand, the sum of the first two electron steps is 2 x (−0.21) V = −0.42 V for (GFA1)^2+^, being slightly higher (less negative) than the −0.53 V for DDQ. This means that thermodynamically, (GFA1)^2+^ is a stronger two‐electron oxidant than DDQ. We recently showed that redox‐active guanidines effectively oxidize dihydroquinones to the corresponding (high‐potential) quinones [32].

2) Under the assumption that the redox potential is directly correlated with the rate for electron transfer (Marcus theory), the potentials also indicate that the rate constant *k*_1_, associated with the formation of the amine radical cation, is much larger for DDQ than for (GFA1)^2+^. On the other hand, the second electron transfer is much slower for DDQ, since there is a large potential difference between the first (*E*_1/2_ = +0.14 V) and the second (*E*_1/2_ = −0.67 V) electron transfer. Hence, *k*_2_ is significantly lower for DDQ than for (GFA1)^2+^ (see Figure 3b). Consequently, the lifetime of the intermediate amine radical cation should be longer for reactions with DDQ. This longer radical lifetime is the reason for the formation of complex reaction products for some of the reactions with DDQ. A relatively long lifetime of the radical intermediates in the case of DDQ leads to the unselective reactions. Todd and coworkers were able to isolate the crystallize the iminium ion (Scheme 1c) that was formed after the second electron transfer. However, the yield was low (50%) and the regeneration of the radical intermediate (rate constant k_−2_ in Figure 3b) was likely to be prevented by the precipitation of the iminium salt. In the case of reactions with (GFA1)^2+^, radicals are also involved, but due to the similar potentials for one‐ and two‐electron reduction, these radicals exhibit a much shorter lifetime.

## 3 Conclusions

Cross‐dehydrogenative coupling reactions (CDC) are of high importance in modern synthetic chemistry, as they are efficient, environmentally‐friendly ways to form new element‐element bonds. They require a CDC reagent that takes up the two protons and two electrons released in the bond forming process (see equation in Scheme 1a). Here, we report the first CDC reactions using a redox‐active guanidine as CDC reagent, namely the salt GFA1(PF_6_)_2_ of the oxidized, dicationic 1,4‐bis(tetramethylguanidino)benzene (GFA1). The molecules *N*‐phenyltetrahydroisoquinoline (PTQ), 2‐[4‐(methyl)phenyl]‐1,2,3,4‐tetrahydroisoquinoline (PTQ‐Me), 2‐(4‐methoxyphenyl)‐1,2,3,4‐tetrahydroisoquinoline (PTQ‐OMe), 2‐(4‐acetyl)‐1,2,3,4‐tetrahydroisoquinoline (PTQ‐COMe) and 2‐[4‐(trifluoromethyl)phenyl]‐1,2,3,4‐tetrahydroisoquinoline (PTQ‐CF_3_) react with nitromethane to give the CDC products in high yield. A Hammett analysis confirmed a large substituent effect on these CDC reactions. Moreover, redox‐active guanidine mediated CDC reactions of tetrahydroisoquinolines with dimethylmalonate and methylphenyl ketone to the corresponding CDC products are reported. By contrast, reactions with the high‐potential quinone 2,3‐dichloro‐5,6‐dicyano‐1,4‐benzoquinone (DDQ) in place of (GFA1)^2+^ lead in some cases to complex product mixtures, from which the CDC products cannot be isolated. Based on the likely mechanistic scenario for such reactions, an explanation is given for the better performance of redox‐active guanidines compared to DDQ. According to this explanation, the lifetime of radical intermediates formed upon the first one‐electron transfer step decides on the selectivity of the reactions. Since the first and second redox steps have very different potentials for high‐potential quinones such as DDQ, the lifetime of radical intermediates is generally relatively long. On the other hand, the similar redox potentials for the first and second one‐electron reduction of redox‐active guanidines such as (GFA1)^2+^ lead to a short lifetime of radical intermediates, thereby favoring the selective formation of the CDC product. The results of this work pave the way for a broad use of redox‐active guanidines in CDC reactions and other dehydrogenative coupling reactions.

## Conflicts of Interest

The authors do not have a conflict of interest.

## Supporting information




**Supporting File**: The authors have cited additional references within the Supporting Information [43, 44, 45, 46, 47, 48, 49, 50, 51, 52, 53, 54, 55, 56, 57, 58, 59].
