# Supplementary material for: Cross‐Dehydrogenative Coupling Reactions With Redox‐Active Guanidine Reagents
Source: Chemistry. 2026 Jul 3;32(38):e71329. doi: 10.1002/chem.71329 (PMC13660496; doi:10.1002/chem.71329)
Supplement: Supplementary file 1 [file CHEM-32-e71329-s001.pdf]

Content

1 General information 2
2 Synthesis of reagents 3
3 Analytical data of reagents 7
NMR spectra 7
Cyclic voltammetry (CV) measurements 12
4 Experimental details of the CDC reactions 15
5 Quantum-chemical calculations 62
6 References 77

1 General information

All synthetic work was carried out using standard Schlenk techniques under argon atmosphere. The solvents acetonitrile, dichloromethane and diethyl ether were dried with an MBraun MB-SPS-800 Solvent Purification System and stored over molecular sieves (3 Å or 4 Å). Other solvents were purchased from Acros Organics and stored similarly. The following chemicals were purchased and used as delivered: 1,4-diaminobenzene·2 HCl (>99%), copper(I)iodide (98%), nitromethane (>99%,) and iodobenzene (for synthesis) from Sigma-Aldrich. Dimethylmalonate (>99%,) and methyl phenyl ketone (>98%) from Acros, 1-iodo-4-(trifluoromethyl)benzene (98%) and 1-iodo-4-methoxybenzene (98.95%) from BLDpharm, 1,2,3,4-tetrahydroisoquinoline (97%) from alfa aesar. The synthesis of ferrocenium hexafluorophosphate [1] and chloroformamidinium chloride [2] followed the literature procedure. Elemental analyses were performed at the Microanalytical Laboratory of Heidelberg University using the vario EL and vario MICRO cube devices from Elementar Analysensysteme GmbH. NMR spectra were recorded on a Bruker Avance II 400, Bruker AVANCE III 600 or Bruker 600 Ultrashield system. Solvent resonances were taken as references for all ^1H NMR spectra. UV-Vis spectra were recorded with a Cary 5000 spectrophotometer. CV measurements were carried out with a Metrohm Autolab PGSTAT 204 potentiostate/galvanostate and an Ag/AgCl reference electrode, Pt rod counter electrode and glassy carbon working electrode. All voltammograms were recorded at room temperature. Dichloromethane (CH_2Cl_2) was used as solvent for the compounds (concentration around 10^{-3} M), whereas $n$$Bu_4N(PF_6)$ (electrochemical grade (≥99.0%), Fluka) was employed as supporting electrolyte (c = 0.1 M).

2 Synthesis of reagents

1,4-Bis(tetramethylguanidino)benzene (GFA1)

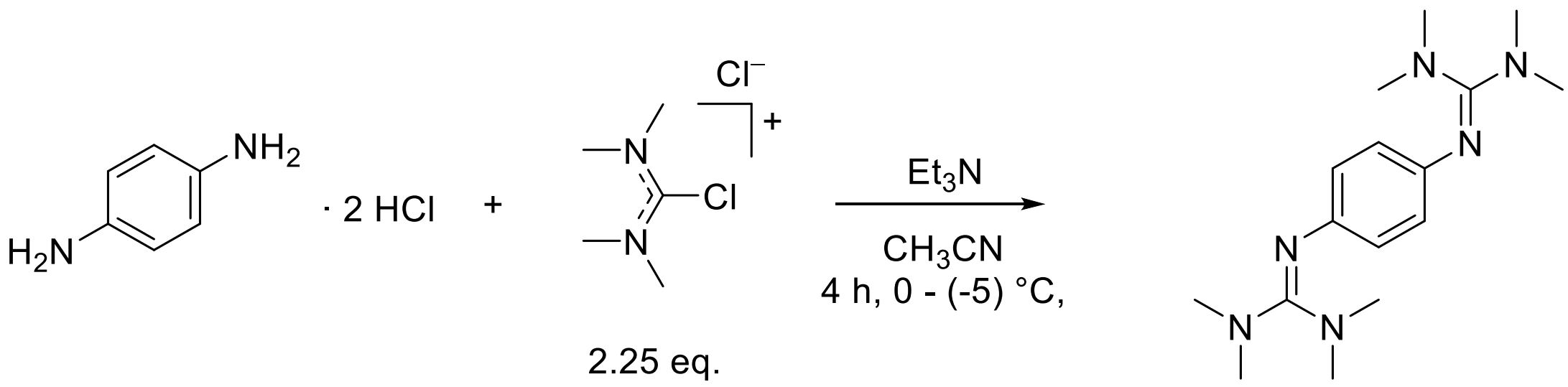

The previously published protocol [3] was varied (purification by crystallization from hot acetonitrile instead of sublimation to increase the product yield).

A suspension of 1,4-benzenediamine dihydrochloride (2.01 g, 11.1 mmol) and triethylamine (11.3 ml, 81.5 mmol) in acetonitrile (25 mL) was cooled to -5 °C. At this temperature a 1 M solution of chloroformamidinium chloride in acetonitrile (25 mL, 25 mmol) was slowly added. The reaction mixture was stirred at 0 - (-5) °C for 4 h. The solvent was removed under vacuum. The remaining residue was redissolved in aqueous HCl (5%) and deprotonated by the adding of a solution of sodium hydroxide in water (40%). The resulting mixture was extracted with dichloromethane. The organic phases were dried over K_2CO_3 and the solvent was removed under vacuum. The crude product was purified by recrystallization from hot acetonitrile to give GFA1 as light beige solid in a yield of 2.8 g (9.2 mmol, 83%). Elemental analysis for $C_{16}H_{28}N_6$ (304.44 g·mol^{-1}, %): calcd. C 63.12, H 9.27, N 27.61; found C 63.14, H 10.12, N 27.45. ^{1}H NMR (399.89 MHz, CD_3CN, 295.4 K): δ = 6.44 (s, 4 H, $CH_{arom.}$), 2.62 (s, 24 H, CH_3) ppm; ^{13}C NMR (100.56 MHz, CD_3CN, 295.9 K): δ = 159.5, 145.9, 122.5, 39.8 ppm.

Additional analytical data for this compound can be found in ref. 3.

GFA1(PF_6)$_2$

The previously published protocol [3] was varied (oxidation with ferrocenium instead of Ag^+). A solution of 1,4-bis(tetramethylguanidino)benzene (0.125g, 0.41 mmol) in dry dichloromethane (3 mL) was added to a suspension of ferrocenium hexafluorophosphate ($FcPF_6$) (0.250 g, 0.76 mmol) in dry dichloromethane (3 mL). Then the reaction mixture was stirred at room temperature for 2 h. The solvent was removed in vacuo and the residue washed three times with diethyl ether. The residue was re-dissolved in acetonitrile and layered with diethylether. One obtains GFA1(PF_6)$_2$ as clear yellow solid in a yield of 187 mg (0.32 mmol, 83%). Elemental analysis for $C_{16}H_{28}N_6F_{12}P_2$ (594.32g·mol^{-1}, %): calcd. C 32.33, H 4.75, N 14.14; found C 33.04, H 4.90, N 14.57. ^{1}H NMR (600.15 MHz, CD_3CN, 298 K): δ = 7.06 (s, 4 H, $CH_{arom.)}$, 3.08 (s, 24 H, CH_3) ppm.

Additional analytical data for this compound can be found in ref. 3.

2-Phenyl-1,2,3,4-tetrahydroisoquinoline (PTQ)

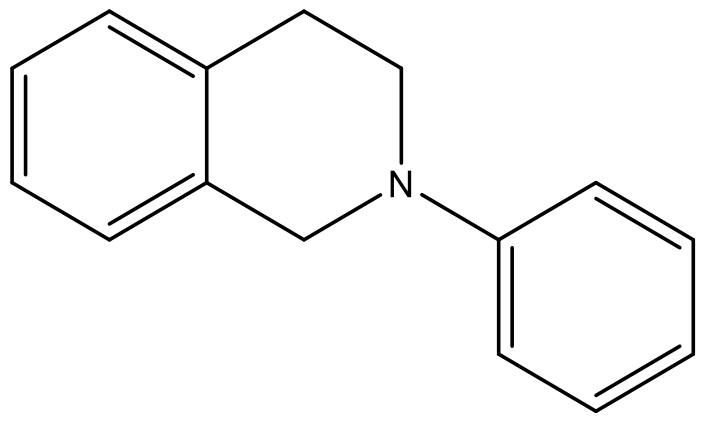

The synthesis of 2-phenyl-1,2,3,4-tetrahydroisoquinoline (PTQ) followed a published protocol.[4]

PTQ was obtained as off-white solid in a yield of 1.21 g (5.65 mol, 56%). Elemental analysis for $C_{15}H_{15}N$ (209.29 g·mol^{-1}, %): calcd. C 86.08, H 7.22, N 6.69; found C 85.43, H 7.13, N 6.81. ^{1}H NMR (600.18 MHz, CD_2Cl_2, 295 K): δ = 7.34 (dd, *J* = 8.69, 7.32 Hz, 2 H, $CH_{arom.}$), 7.23 (dd, *J* = 9.91, 5.97 Hz, 4 H, $CH_{arom.}$), 7.03 (d, *J* = 8.01 Hz, 2 H, $CH_{arom.}$), 6.88 (t, *J* = 7.26 Hz, 1 H, $CH_{arom.}$), 4.45 (s, 2 H, CH_2), 3.60 (t, *J* = 5.87 Hz, 2 H, CH_2), 3.03 ((t, *J* = 5.83 Hz, 2 H, CH_2) ppm; ^{13}C NMR (150.92 MHz, $CDCl_3$, 298 K): δ = 150.7, 135.0, 134.6, 129.4, 128.7, 126.7, 126.5, 126.2, 118.8, 115.3, 50.9, 46.7, 29.3 ppm.

2-[4-(Trifluoromethyl)phenyl]-1,2,3,4-tetrahydroisoquinoline (PTQ-CF_3)

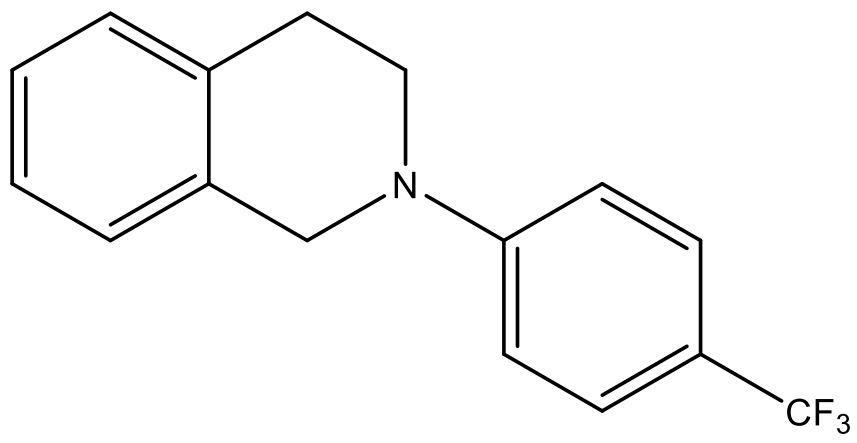

The synthesis of 2-[4-(trifluoromethyl)phenyl]-1,2,3,4-tetrahydroisoquinoline (PTQ-CF_3) followed a published protocol.[5]

PTQ-CF_3 was obtained as off-white solid in a yield of 1.59 g (6.7 mmol, 61%). Elemental analysis for $C_{16}H_{14}NF_3$ (277.29 g·mol^{-1}, %): calcd. C 69.31, H 5.09, N 5.05; found C 68.48, H 5.33, N 5.17. ^{1}H NMR (600.15 MHz, $CDCl_3$, 298 K): δ = 7.51 (d, *J* = 8.85 Hz, 2 H, $CH_{arom.}$), 7.21 (d, *J* = 7.18 Hz, 4 H, $CH_{arom.}$), 6.94 (d, *J* = 8.72 Hz, 2 H, $CH_{arom.}$), 4.49 (s, 2 H, CH_2), 3.63 (t, *J* = 5.88 Hz, 2 H), 3.00 (t, *J* = 5.82 Hz, 2 H) ppm; ^{13}C NMR (150.92 MHz, $CDCl_3$, 298 K): δ = 152.3, 135.0, 134.0, 128.5, 126.9, 126.7, 126.6, 126.5, 124.2, 119.3, 113.0, 49.6, 45.4, 29.1 ppm.

2-(4-Methoxyphenyl)-1,2,3,4-tetrahydroisoquinoline (PTQ-OMe)

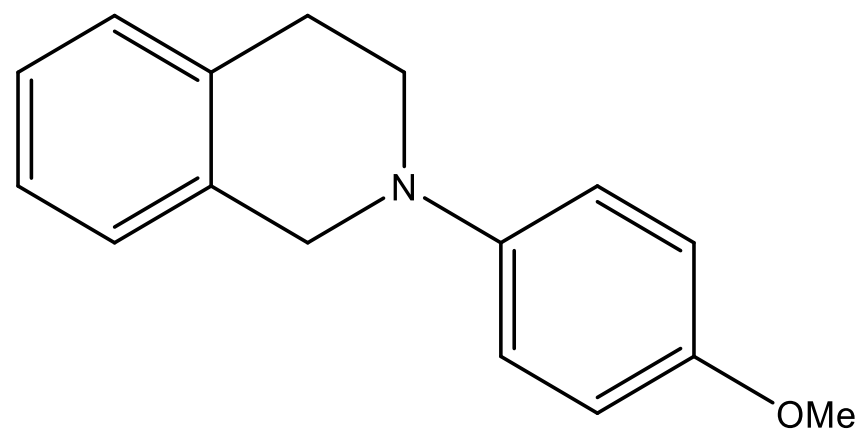

The synthesis of 2-(4-methoxyphenyl)-1,2,3,4-tetrahydroisoquinoline (PTQ-OMe) followed a published protocol.[5]

PTQ-OMe was obtained as off-white solid in a yield of 1.20 g (5.0 mmol, 50%). Elemental analysis for $C_{16}H_{17}NO$ (239.32 g·mol^{-1}, %): calcd. C 80.30, H 7.16, N 5.85; found C 80.48, H 6.90, N 6.11. ^{1}H NMR (600.15 MHz, $CDCl_3$, 298 K): δ = 7.17 (dd, *J* = 11.18, 4.70 Hz, 4 H, $CH_{arom.}$), 6.99 (d, *J* = 9.0 Hz, 2 H, $CH_{arom.}$), 6.87 (d, *J* = 9.1 Hz, 2 H, $CH_{arom.}$), 4.30 (s, 2 H, CH_2), 3.78 (s, 3 H, OCH_3), 3.45 (t, *J* = 5.9 Hz, 2 H, CH_2), 2.99 (t, *J* = 5.71 Hz, 2 H, CH_2) ppm; ^{13}C NMR (150.92 MHz, $CDCl_3$, 298 K): δ = 153.6, 145.5, 134.8, 134.7, 128.8, 126.7, 126.4, 126.1, 118.2, 114.8, 55.8, 52.8, 48.6, 29.3 ppm.

2-Phenyl-1,2,3,4-tetrahydroisoquinoline (PTQ-Me)

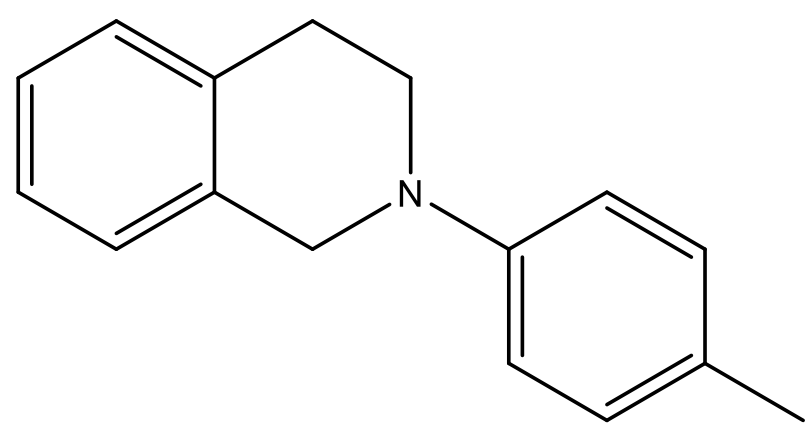

The synthesis of 2-(p-tolyl)-1,2,3,4-tetrahydroisoquinoline (PTQ-Me) followed a published protocol.[7]

PTQ-Me was obtained as yellow oil in a yield of 0.88 g (3.94 mmol, 39%). ^{1}H NMR (600.18 MHz, CD_2Cl_2, 295 K): δ = 7.17 (dd, *J* = 3.8, 2.4 Hz, 4H), 7.09 (s, 2H), 6.90 (d, *J* = 8.5 Hz, 2H), 4.34 (s, 2H), 3.50 (t, *J* = 5.9 Hz, 2H), 2.97 (t, *J* = 5.9 Hz, 2H), 2.27 (s, 3H), 2.27 (s, 3H) ppm; ^{13}C NMR (150.92 MHz, CD_2Cl_2, 298 K): δ = 149.1, 135.3, 135.1, 130.0, 128.9, 128.5, 126.9, 126.6, 126.3, 116.0, 51.7, 47.6, 29.4, 20.5 ppm.

1-(4-(3,4-dihydroisoquinolin-2(1H)-yl)phenyl)ethan-1-one (PTQ-COMe)

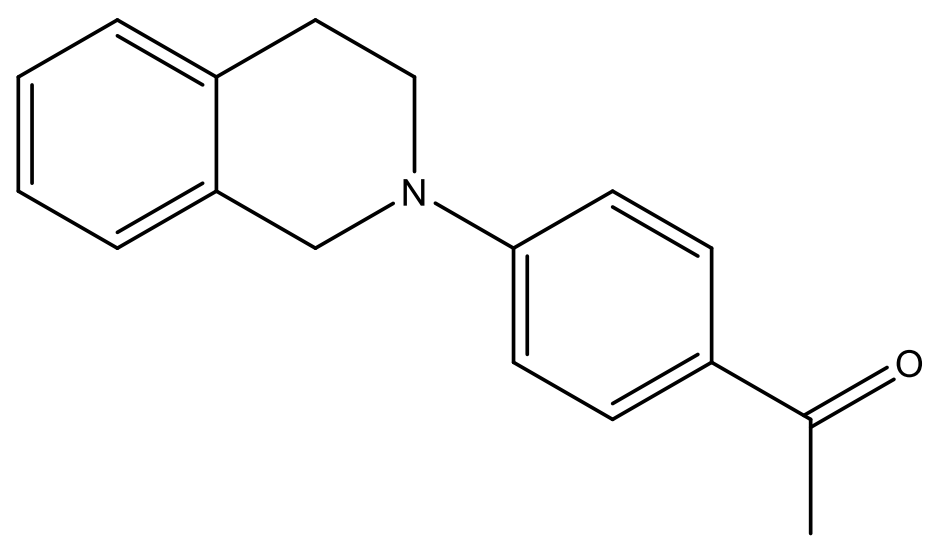

The synthesis of 1-(4-(3,4-dihydroisoquinolin-2(1H)-yl)phenyl)ethan-1-one (PTQ-COMe) followed a published protocol.[7]

PTQ-COMe was obtained as yellow solid in a yield of 0.51 g (2.03 mmol, 45%). ^{1}H NMR (600.18 MHz, CD_2Cl_2, 295 K): δ = 7.91 (d, *J* = 9.0 Hz, 2H), 7.25 – 7.18 (m, 4H), 6.88 (d, *J* = 9.0 Hz, 2H), 4.54 (s, 2H), 3.68 (t, *J* = 5.9 Hz, 2H), 3.00 (t, *J* = 5.9 Hz, 2H), 2.53 (s, 3H) ppm; ^{13}C NMR (150.92 MHz, $CDCl_3$, 298 K): δ = 196.6, 153.2, 135.2, 133.8, 130.7, 128.4, 127.0, 126.7, 126.6, 112.1, 49.1, 44.9, 29.2, 26.2 ppm.

3 Analytical data of reagents

NMR spectra

^{1}H NMR spectrum (600.18 MHz, CD_2Cl_2, 295 K) for PTQ

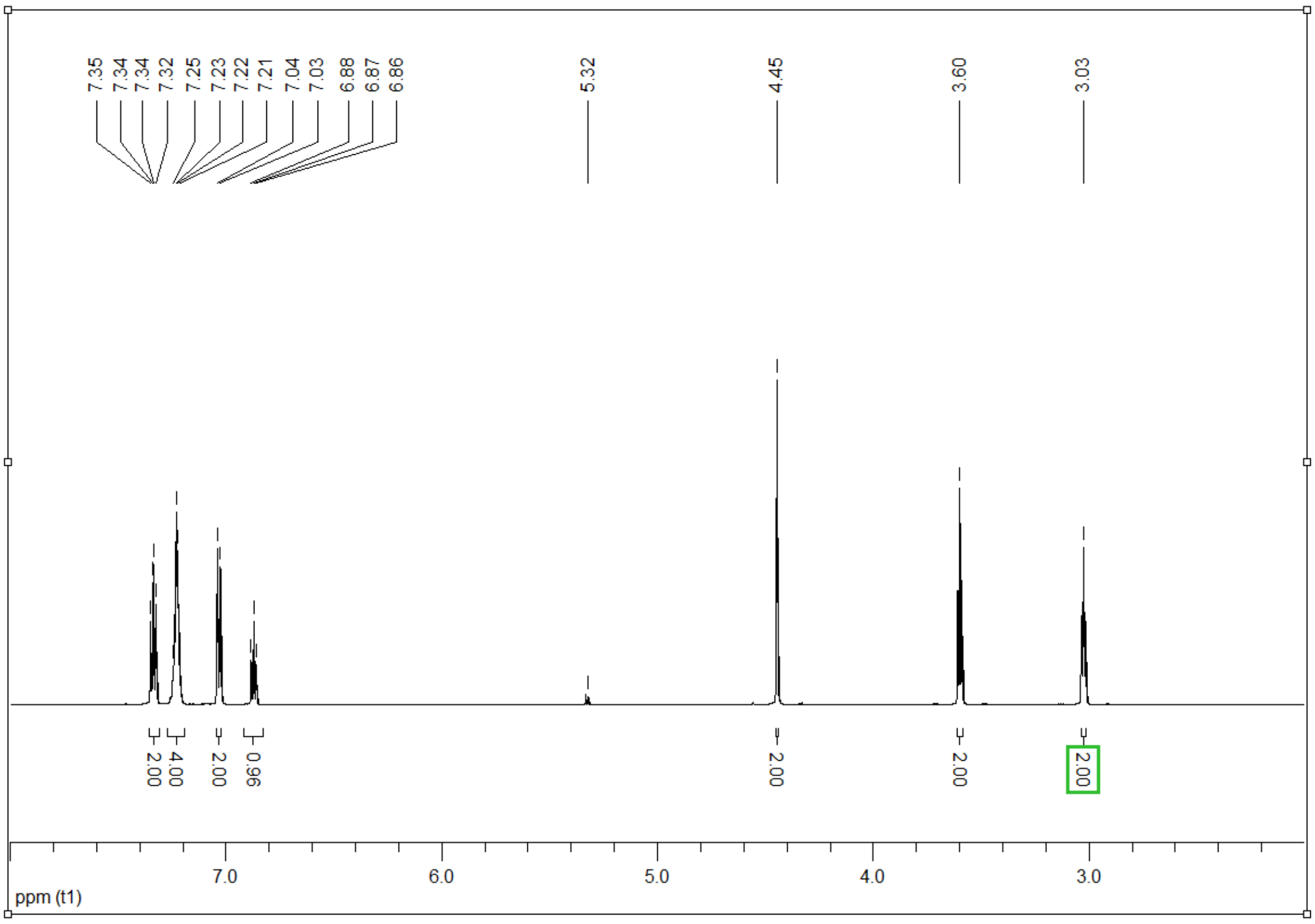

^{13}C NMR spectrum (150.92 MHz, $CDCl_3$, 298 K) for PTQ

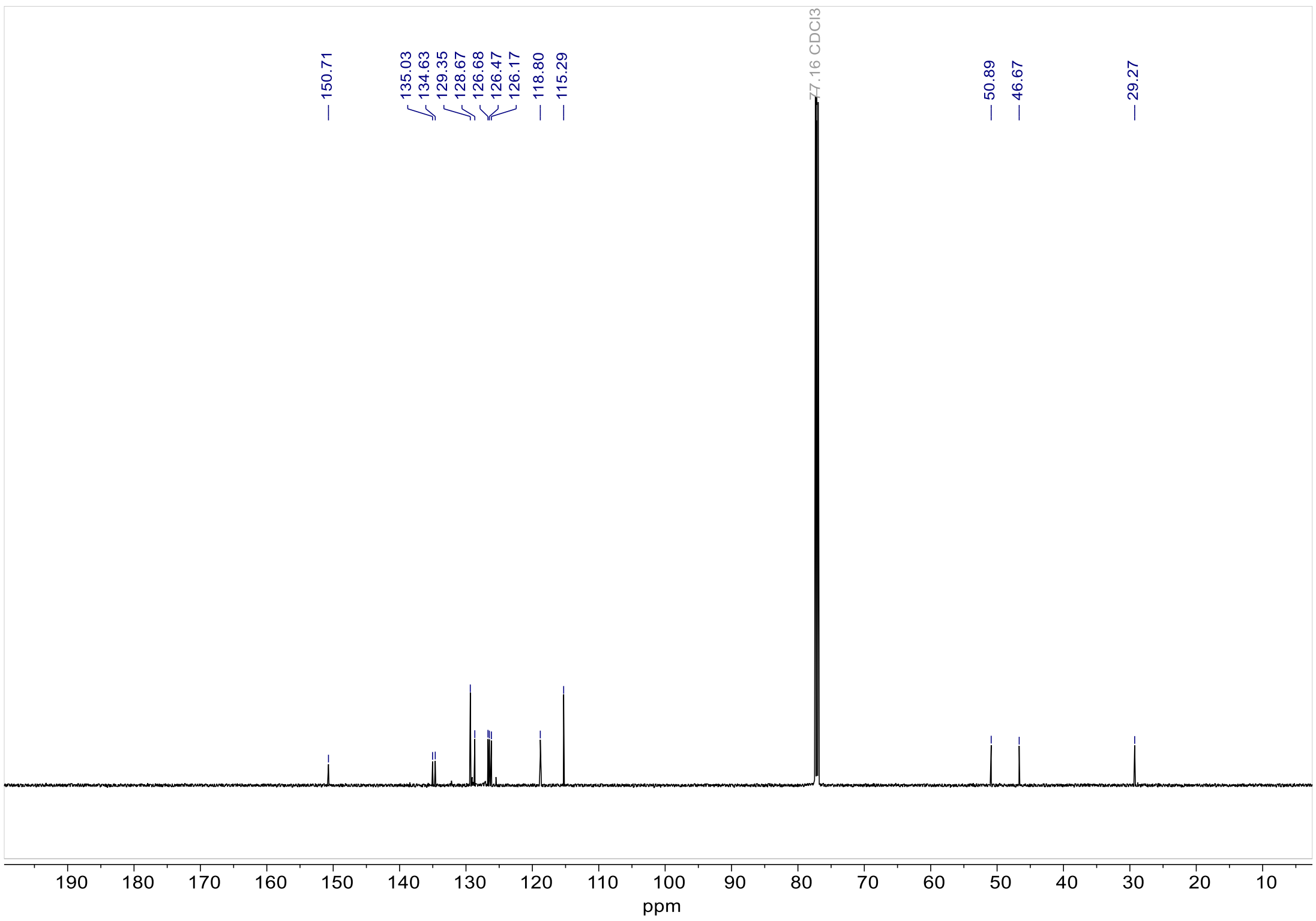

^{1}H NMR spectrum (600.15 MHz, $CDCl_3$, 298 K) for PTQ-CF$_3$

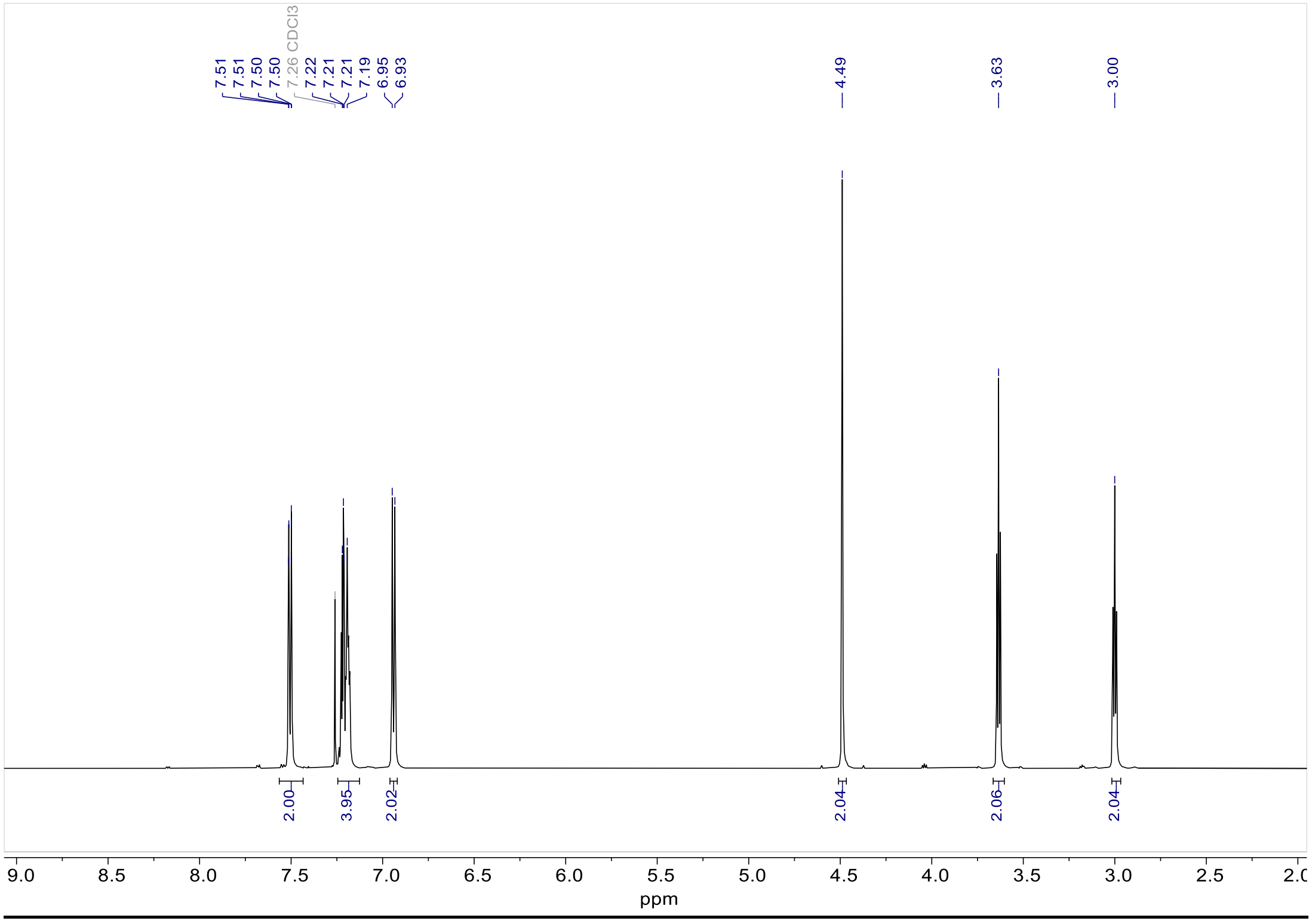

^{13}C NMR spectrum (150.92 MHz, $CDCl_3$, 298 K) for PTQ-CF$_3$

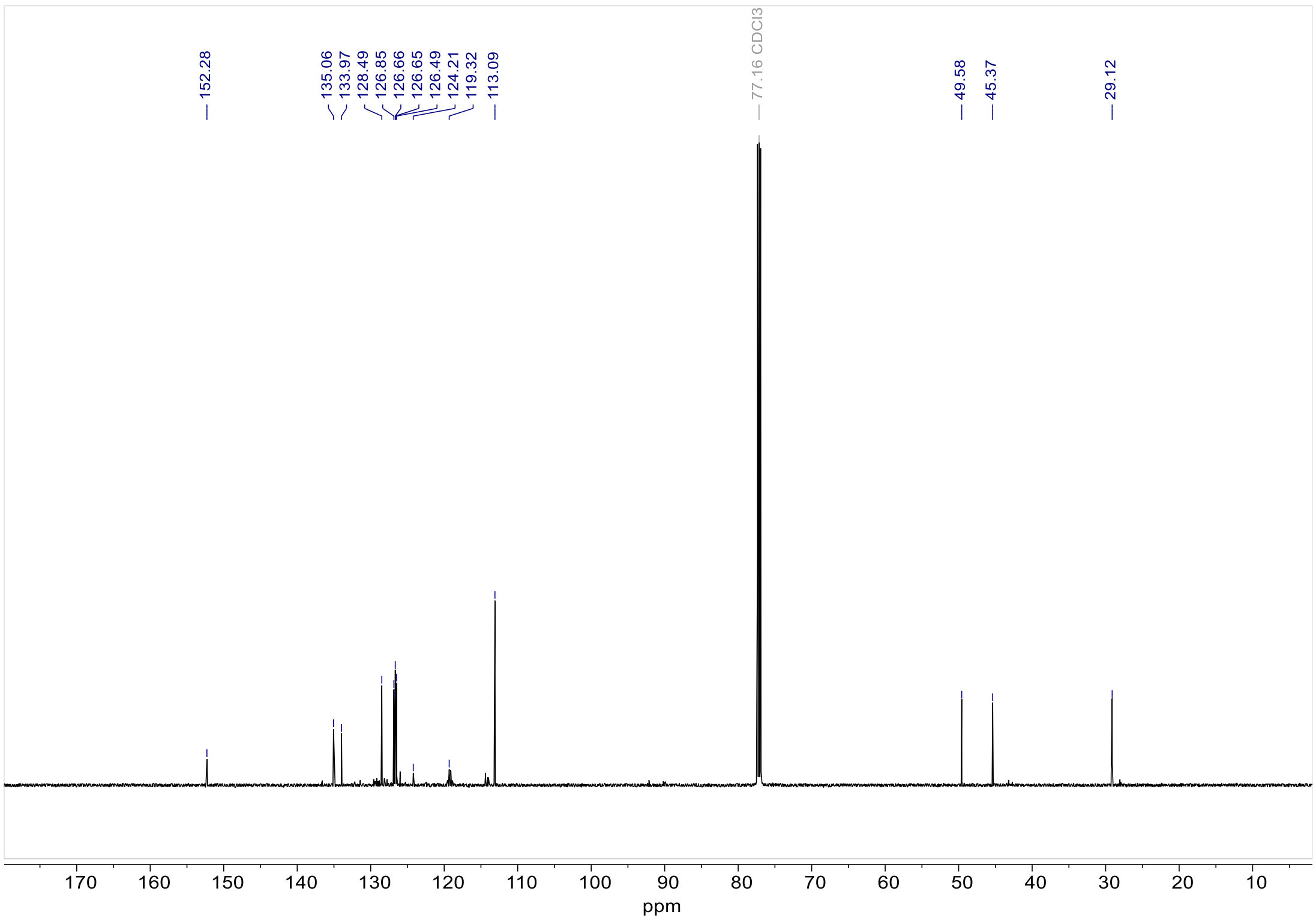

^1H NMR spectrum (600.15 MHz, $CDCl_3$, 298 K) for PTQ-OMe

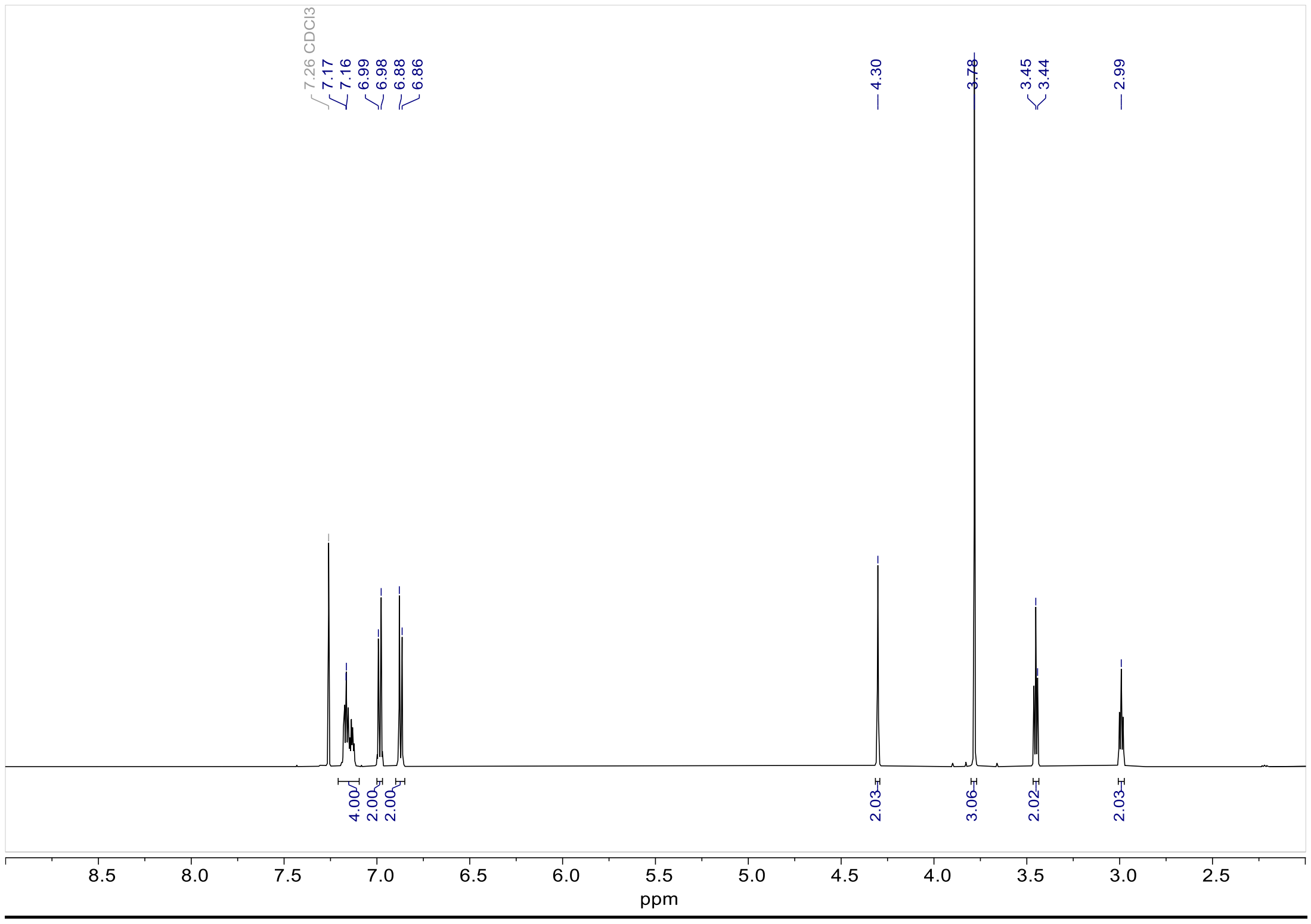

^{13}C NMR spectrum (150.92 MHz, $CDCl_3$, 298 K) for PTQ-OMe

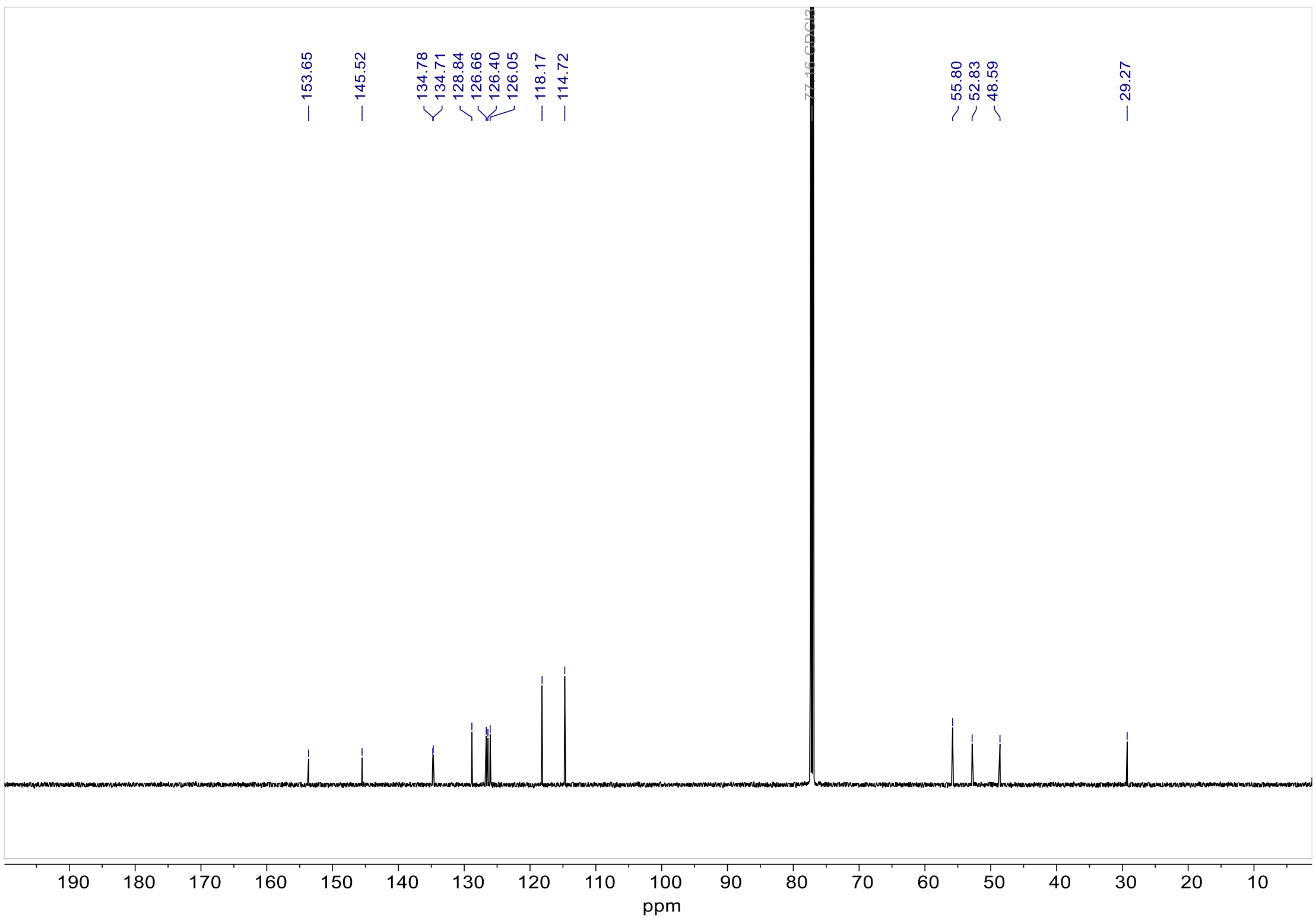

^1H NMR spectrum (600.18 MHz, CD_2Cl_2, 295 K) for PTQ-Me

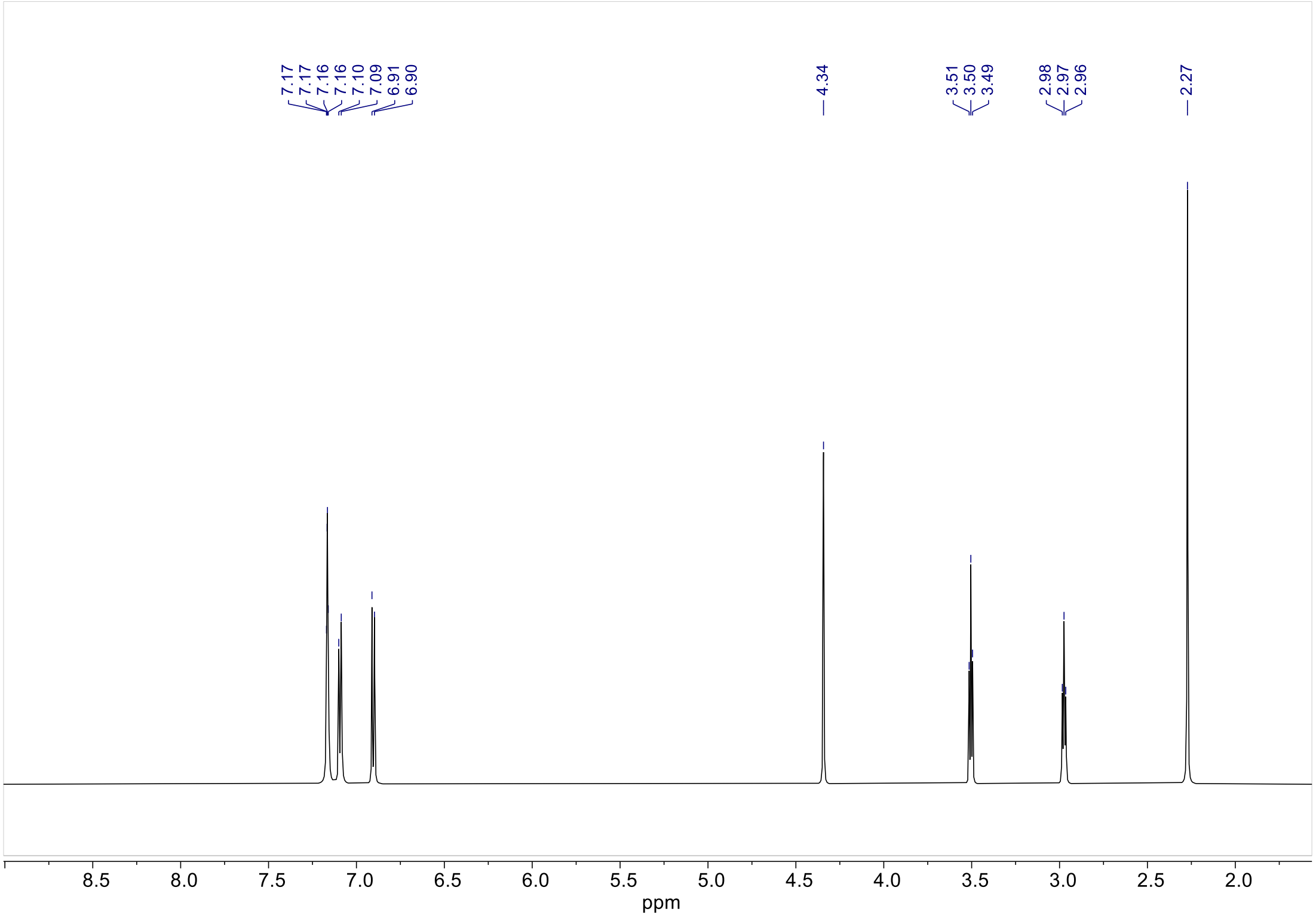

^{13}C NMR spectrum (150.92 MHz, CD_2Cl_2, 298 K) for PTQ-Me

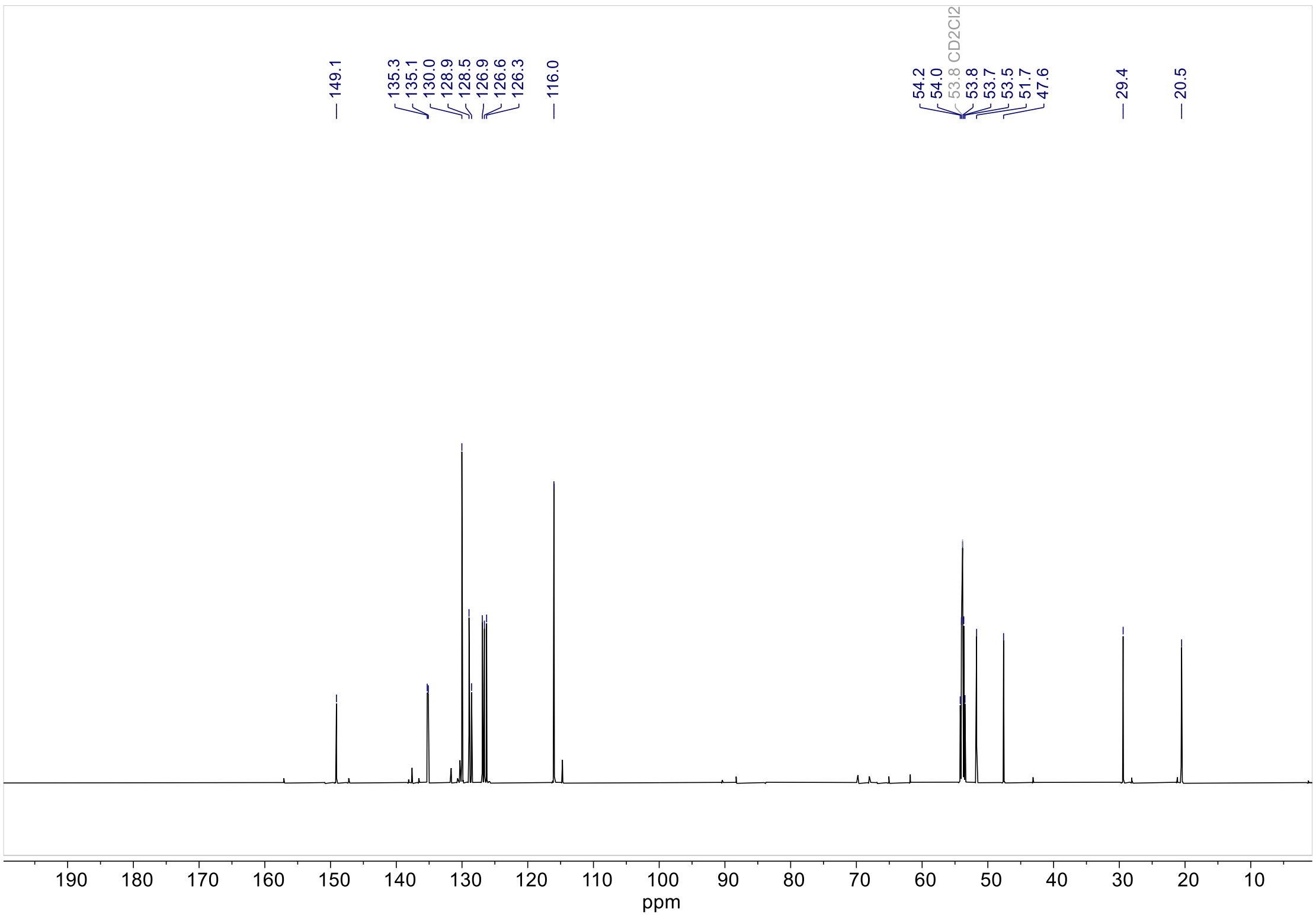

^{1}H NMR spectrum (600.18 MHz, $CDCl_3$, 295 K) for PTQ-COMe

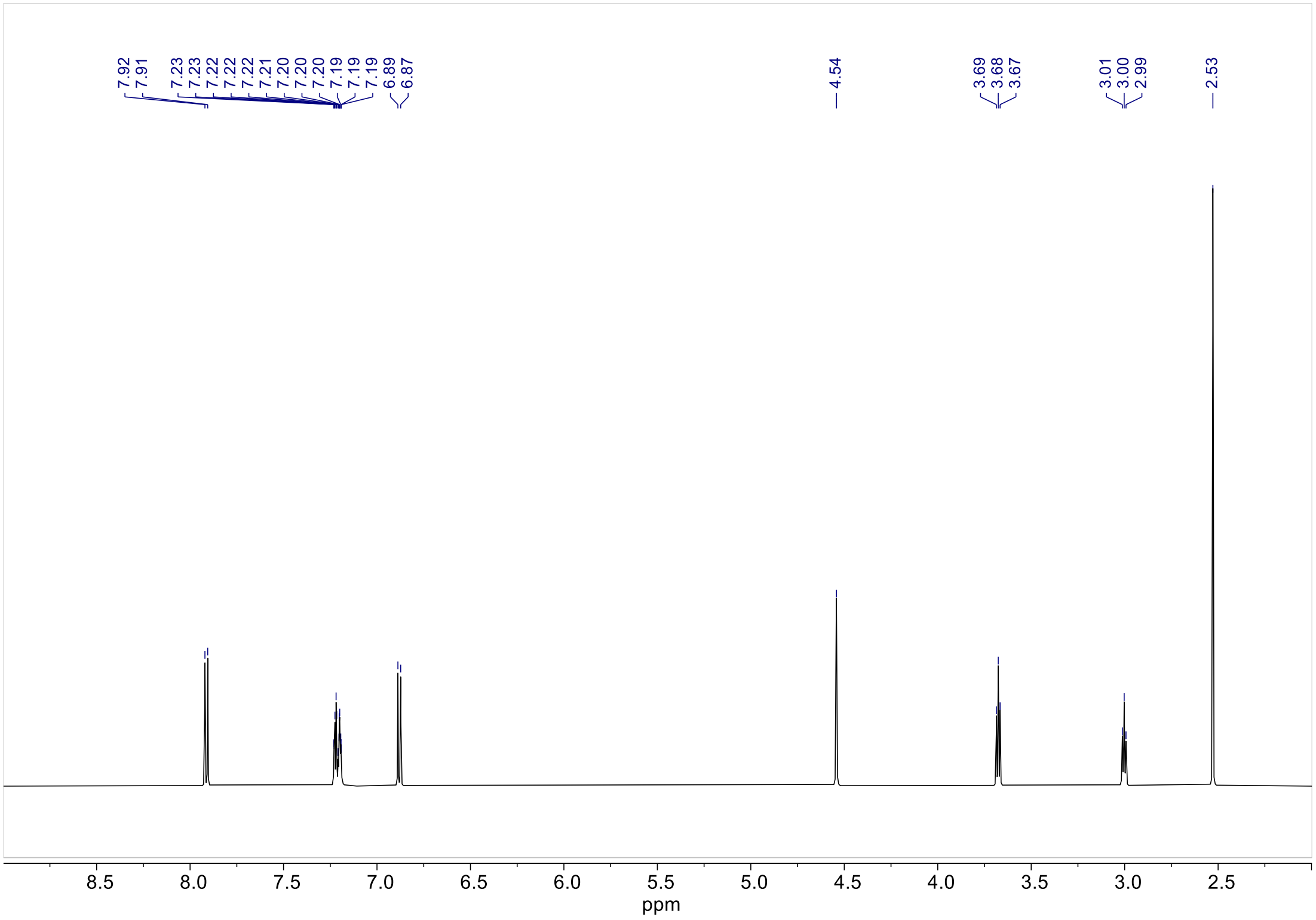

^{13}C NMR spectrum (150.92 MHz, $CDCl_3$, 298 K) for PTQ-COMe

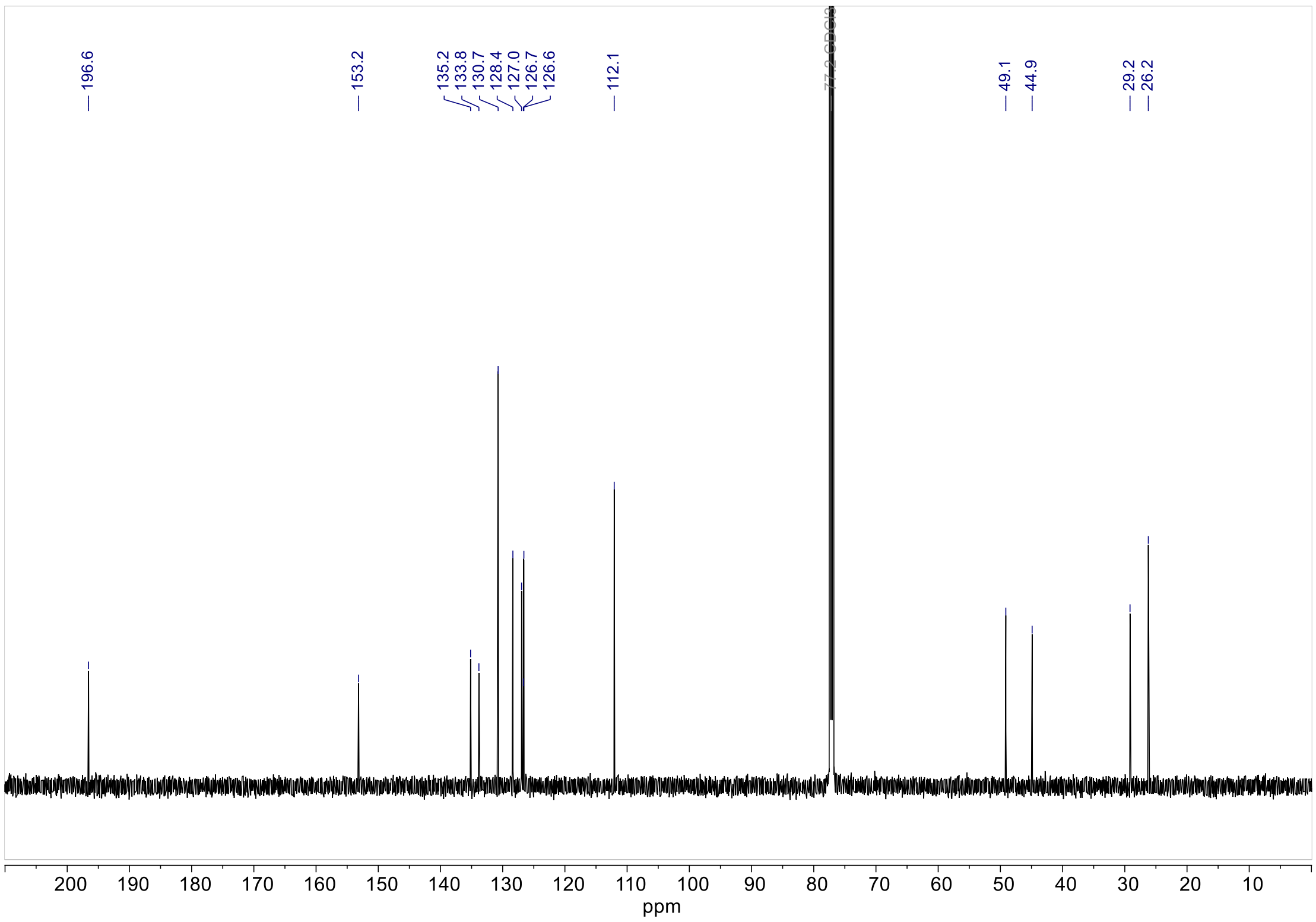

Cyclic voltammetry (CV) measurements

CV curve of 2-phenyl-1,2,3,4-tetrahydroisoquinoline (PTQ) and the derivates PTQ-COMe, PTQ-CF_3, PTQ-OMe and PTQ-Me, (CH_2Cl_2, Ag/AgCl reference electrode, 0.1 M $N(^nBu)_4(PF_6)$ as supporting electrolyte, scan speed 100 mV·s^{-1}) measured in oxidation direction. Potentials given vs. the (Fc^+/Fc) redox couple.

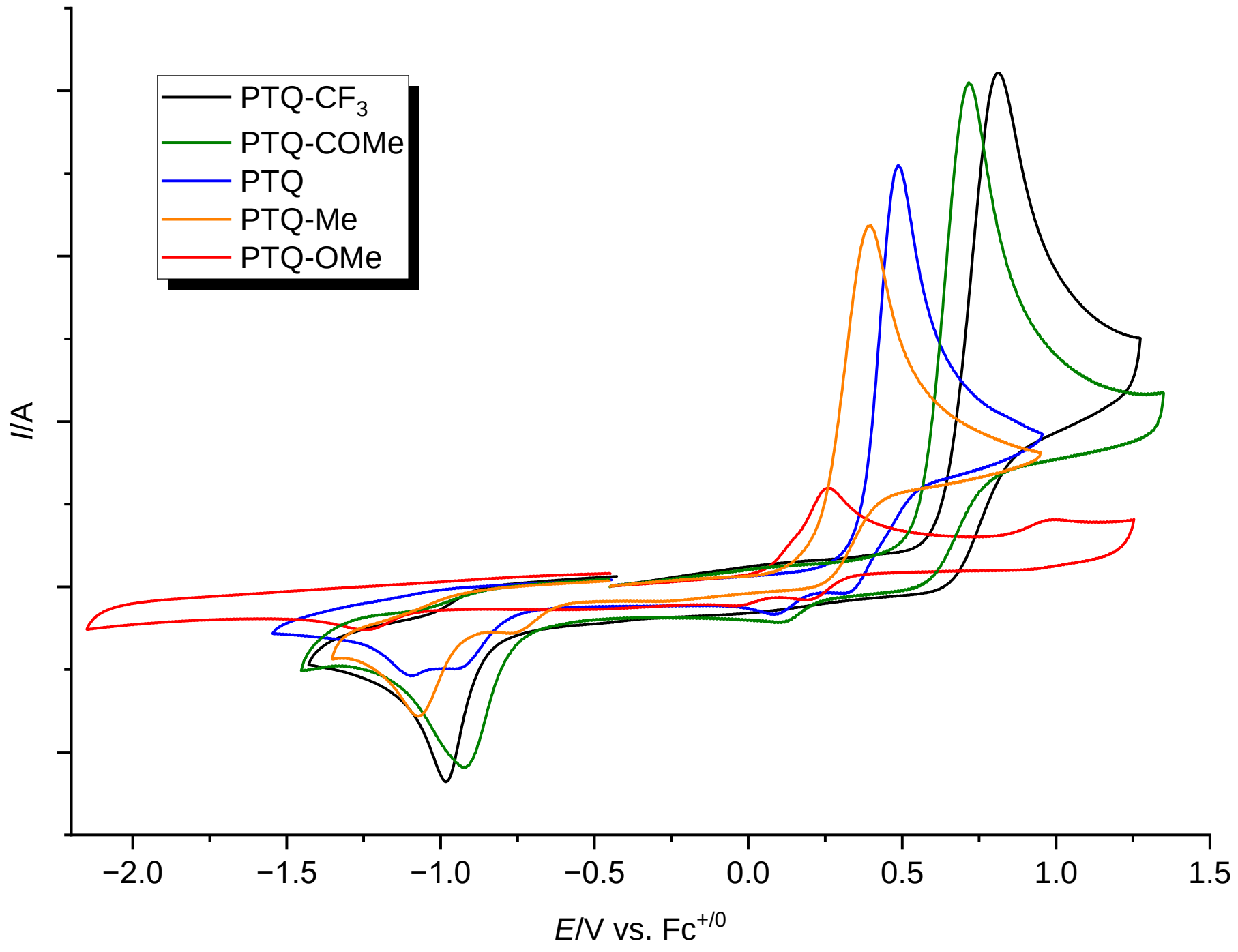

CV curve of PTQ (CH_2Cl_2, Ag/AgCl reference electrode, 0.1 M $N(^nBu)_4(PF_6)$ as supporting electrolyte, scan speed 100 mV·s^{-1}) measured in oxidation direction. Potentials given vs. the (Fc^+/Fc) redox couple. E_{ox} = 0.488 V

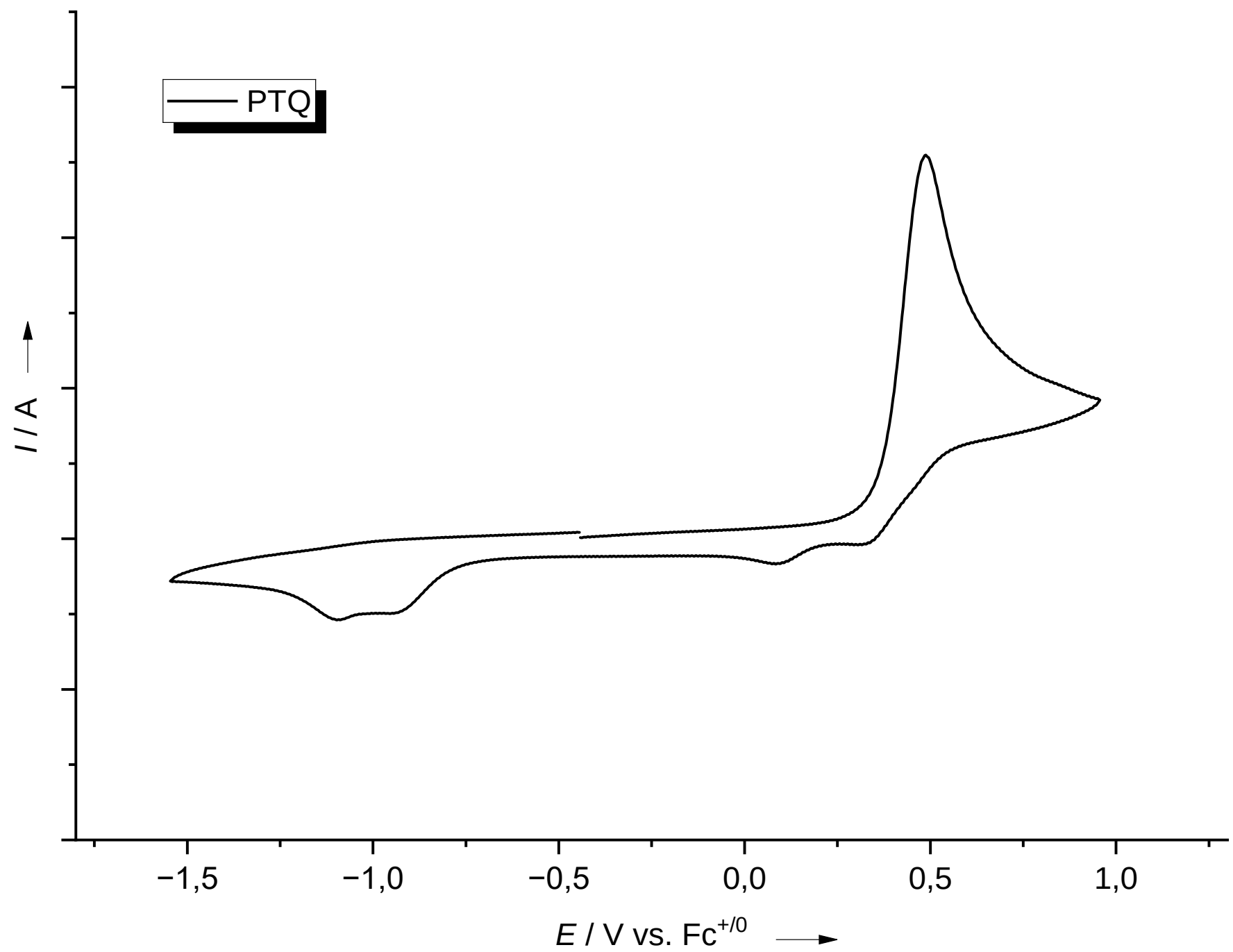

CV curve of PTQ-CF_3 (CH_2Cl_2, Ag/AgCl reference electrode, 0.1 M $N(^nBu)_4(PF_6)$ as supporting electrolyte, scan speed 100 mV·s^{-1}) measured in oxidation direction. Potentials given vs. the (Fc^+/Fc) redox couple. E_{ox} = 0.813 V

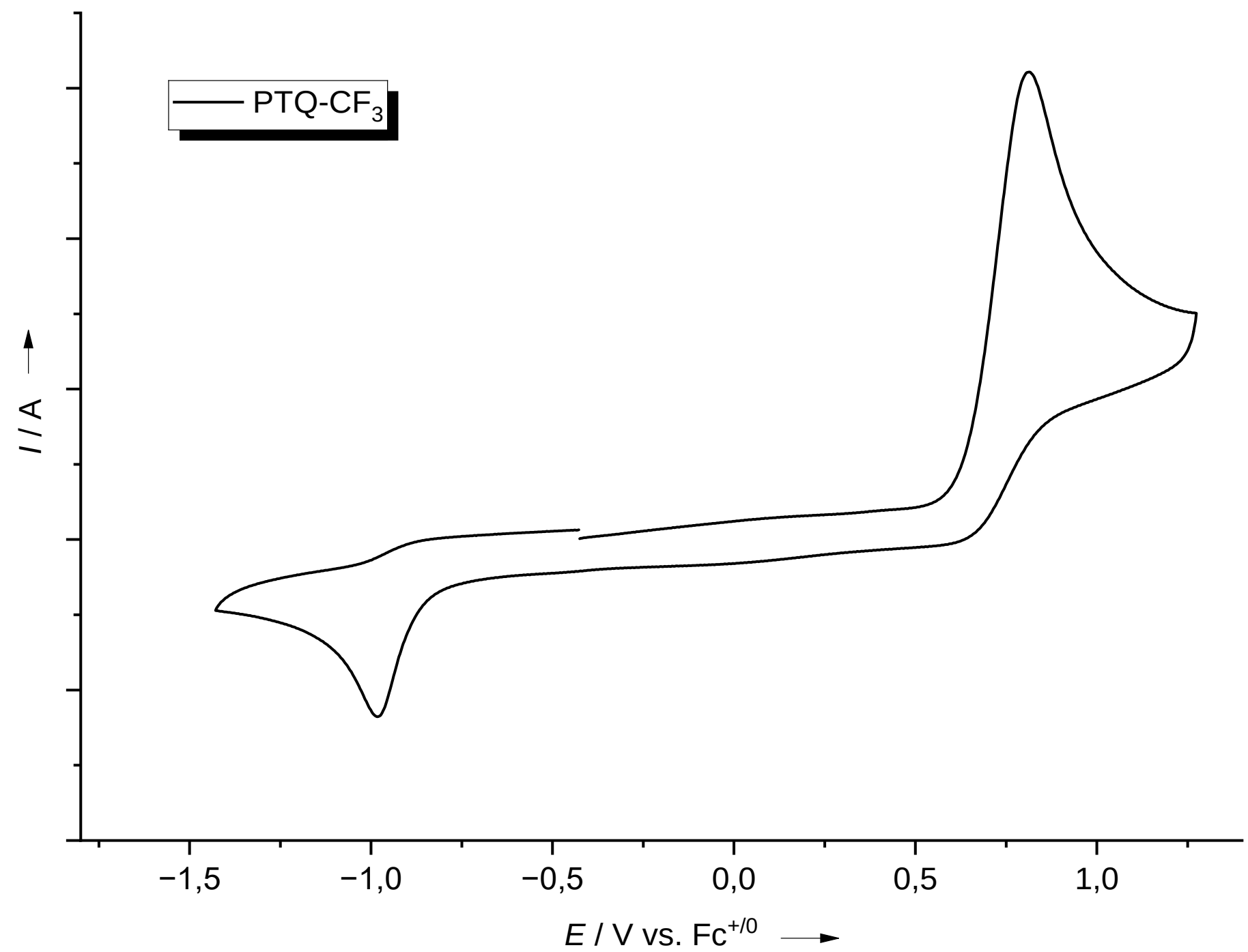

CV curve of PTQ-OMe (CH_2Cl_2, Ag/AgCl reference electrode, 0.1 M $N(^nBu)_4(PF_6)$ as supporting electrolyte, scan speed 100 mV·s^{-1}) measured in oxidation direction. Potentials given vs. the Fc^+/Fc redox couple. E_{ox} = 0.260 V

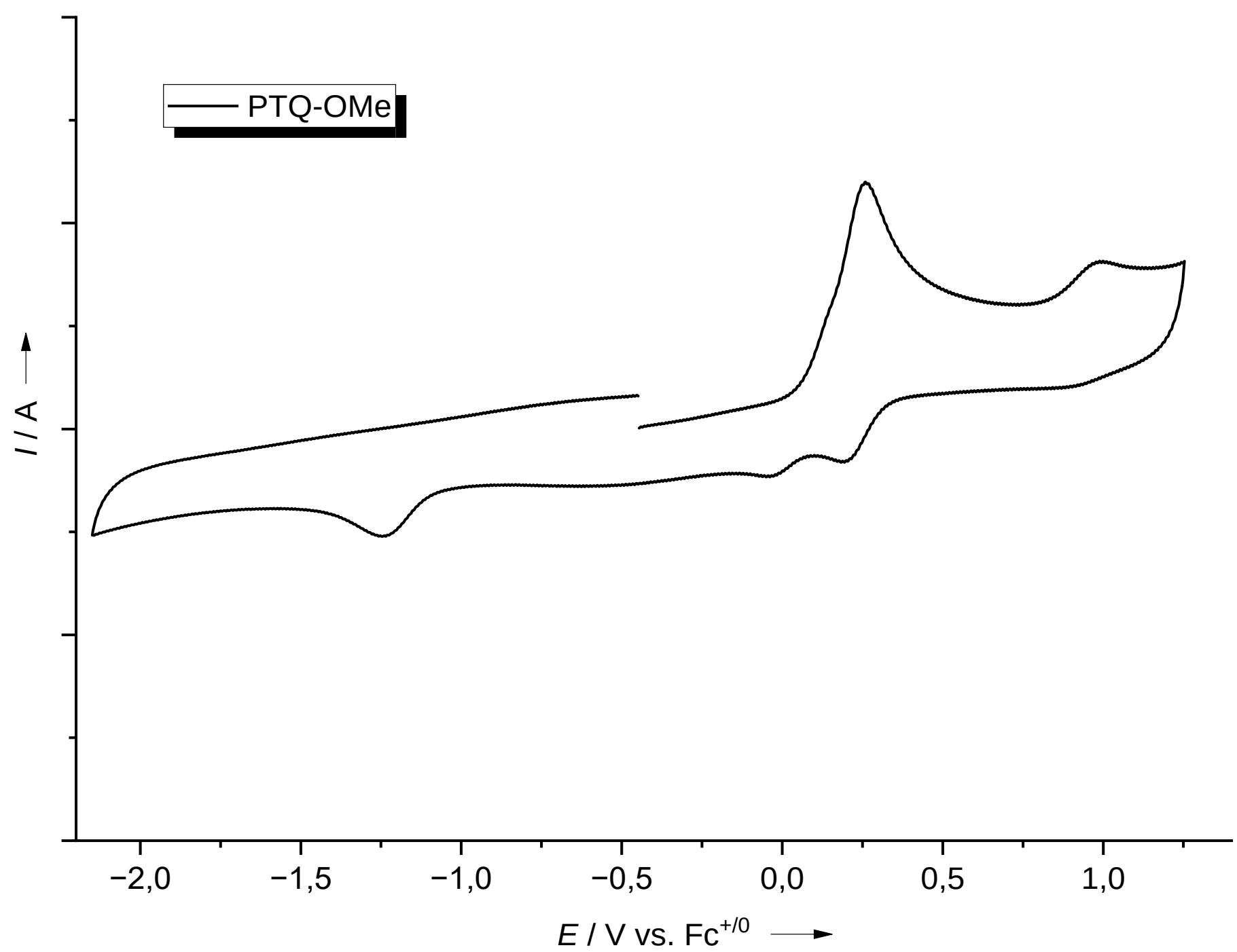

CV curve of PTQ-Me (CH_2Cl_2, Ag/AgCl reference electrode, 0.1 M $N(^{n}Bu)_4(PF_6)$ as supporting electrolyte, scan speed 100 mV·s^{-1}) measured in oxidation direction. Potentials given vs. the (Fc^+/Fc) redox couple. E_{ox} = 0.399 V

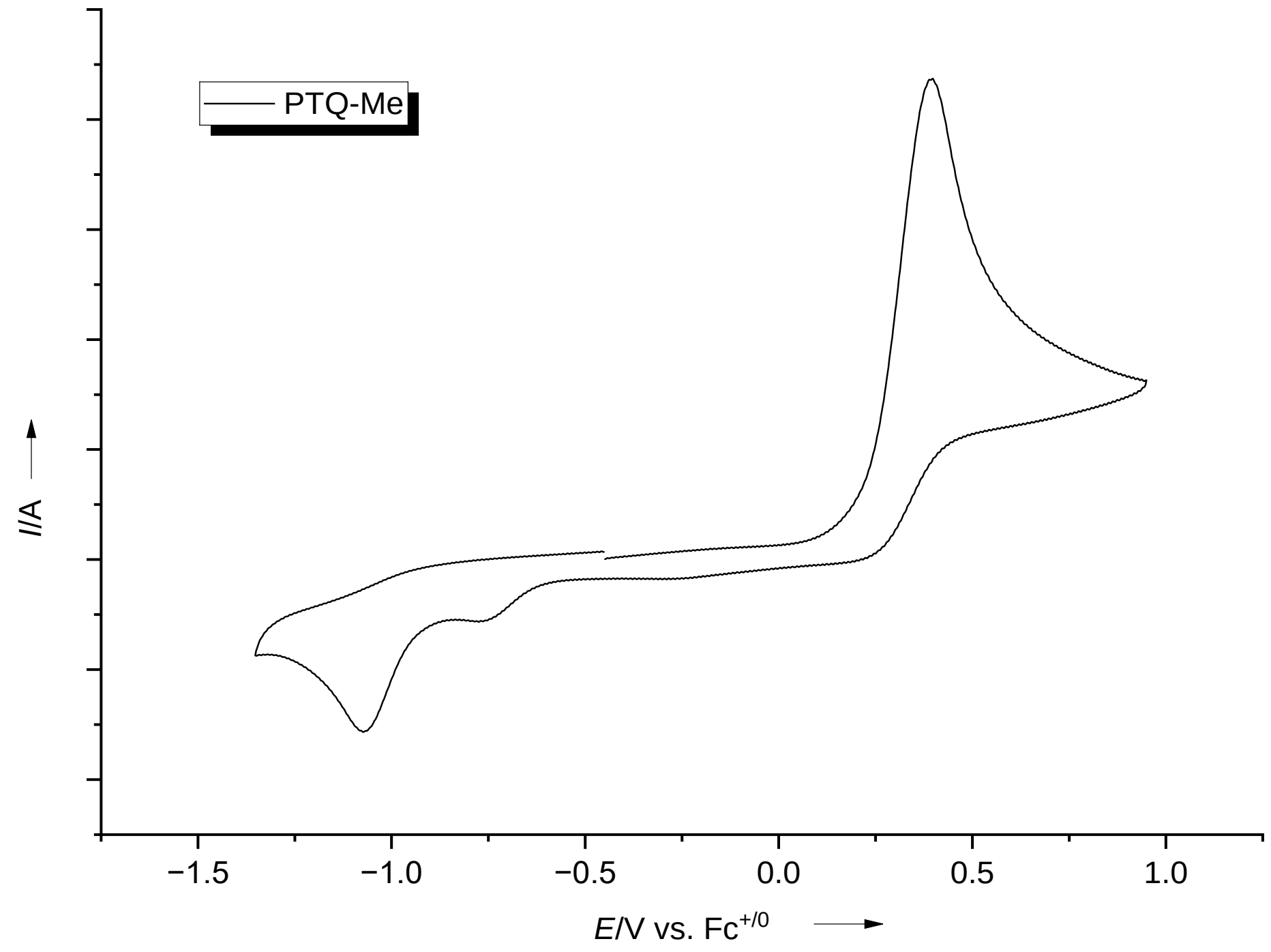

CV curve of PTQ-COMe (CH_2Cl_2, Ag/AgCl reference electrode, 0.1 M $N(^{n}Bu)_4(PF_6)$ as supporting electrolyte, scan speed 100 mV·s^{-1}) measured in oxidation direction. Potentials given vs. the (Fc^+/Fc) redox couple. E_{ox} = 0.718 V

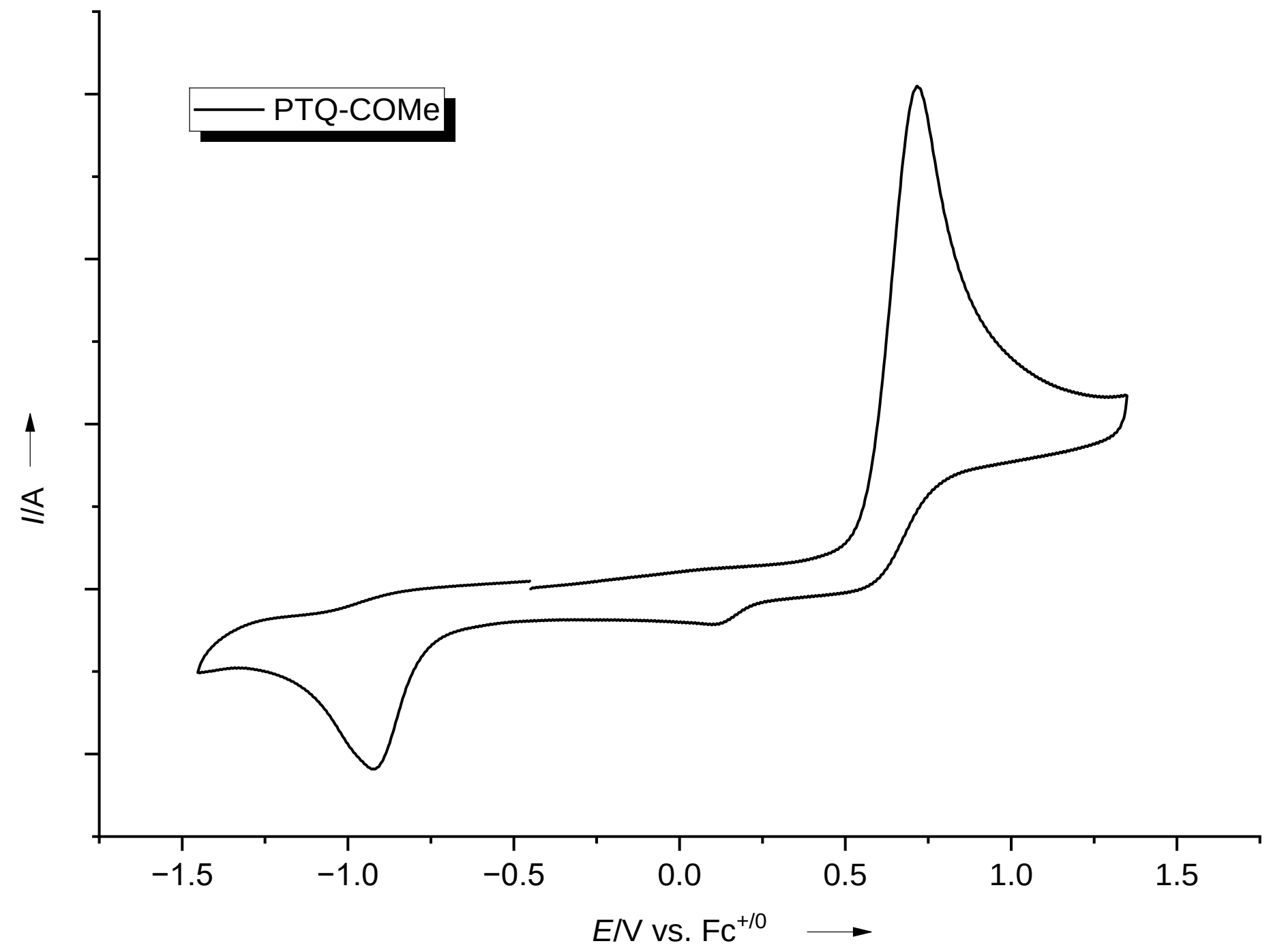

4 Experimental details of the CDC reactions

4.1 Reactions with nitromethane

Reaction between 2-phenyl-1,2,3,4-tetrahydroisoquinoline (PTQ) and nitromethane to give 1-(nitromethyl)-2-phenyl-1,2,3,4-tetrahydroisoquinoline

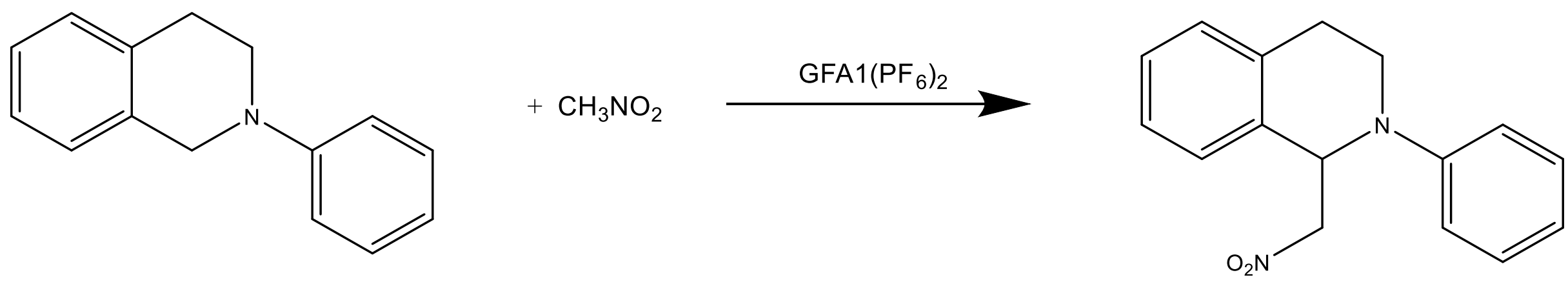

entry	A1	B1	C1	D1
$GFA1(PF_6)_2$ [mg / µmol]	-	-	15.98 / 27	65 / 110
DDQ [mg / µmol]	120 / 530	83.2 / 380	-	-
PTQ [mg / µmol]	95 / 460	71.2 / 340	5.23 / 25	20 / 96
nitromethane [ml]	3	3	0.5 (D3)	1
ratio PTQ / DDQ	1 / 1.1	1 / 1.1		
ratio PTQ / $GFA1^{2+}$			1 / 1	1 / 1.1
condition	Schlenk flask	Schlenk flask	NMR tube	Schlenk flask
reaction time (temperature)	30 min (r.t.)	3 h (r.t.)	3 h (r.t.)	3 h (r.t.)
Yield after workup [%]	65	89	93[a]	91

[a] without workup, ratio product / reactant from NMR spectroscopie

Protocol for entry A1: The synthesis of 1-(nitromethyl)-2-phenyl-1,2,3,4-tetrahydroisoquinoline followed the protocol given in the literature.[6] The product, 1-(nitromethyl)-2-phenyl-1,2,3,4-tetrahydroisoquinoline, was obtained as a pale-yellow oil (80 mg, 65%);

Protocol for entry B1: Reaction conditions similar to entry **D1** (81 mg, 89%);

Protocol for entry C1: In an NMR tube, the two reactants PTQ and $GFA1(PF_6)_2$ were dissolved in CD_3NO_2. Conversion was followed by ^1H NMR spectroscopy.

Protocol for entry D1: PTQ was added to a solution of $GFA1(PF_6)_2$ in nitromethane. The yellow-brown mixture was stirred at room temperature for 3 h. Then, nitromethane was removed under high vacuum. The residue was suspended in dichloromethane and filtrated over Celite.

The clear yellow solution was concentrated and purified by flash column chromatography (5:1, hexane: ethyl acetate) to give 1-(nitromethyl)-2-phenyl-1,2,3,4-tetrahydroisoquinoline (23 mg, 91%) as a pale-yellow solid. ^{1}H NMR (600.18 MHz, CD_2Cl_2, 295 K): δ = 7.30 (dd, *J* = 15.76, 8.45 Hz, 3 H, $CH_{arom.}$), 7.25 (dd, *J* = 11.65, 7.42 Hz, 2 H, $CH_{arom.}$), 7.18 (d, *J* = 7.58 Hz, 1 H, $CH_{arom.}$), 7.03 (d, *J* = 8.77 Hz, 2 H), 6.88 (t, *J* = 7.28, 7.28 Hz, 1 H), 5.59 (t, *J* = 7.28, 7.28 Hz, 1 H), 4.90 (dd, *J* = 11.96, 8.17 Hz, 1 H), 4.62 (dd, *J* = 11.96, 6.43 Hz, 1 H), 3.66 (ddt, *J* = 13.30, 13.30, 11.20, 4.91 Hz, 2 H), 3.20 - 3.00 (m, 1 H), 2.81 (td, *J* = 16.41, 4.79, 4.79 Hz, 1 H) ppm; ^{13}C NMR (150.94 MHz, CD_2Cl_2, 295.1 K): δ = 148.9, 135.9, 133.2, 129.8, 129.6, 128.4, 127.4, 126.9, 119.6, 115.5, 79.1, 58.4, 42.3, 26.5 ppm. The analytical data matched those in the literature.[7]

Separation of GFA1 for entry D1: The residue on celite containing (GFA1+2 H)$(PF_6)_2$ was solved in acetonitrile, then acetonitrile was removed under high vacuum. The residue was suspended in water, deprotonated with 25% NaOH and extracted several times with dichloromethane. The combined organic phases were dried over K_2CO_3, filtrated and condensed to give 1,4-bis(dimethylamino)benzene (GFA1) as an offwhite solid (21.5 mg, 64%).

^{1}H NMR spectrum (600.18 MHz, CD_3NO_2, 298 K) for entry **C1** first measurement (30 min, r.t.)

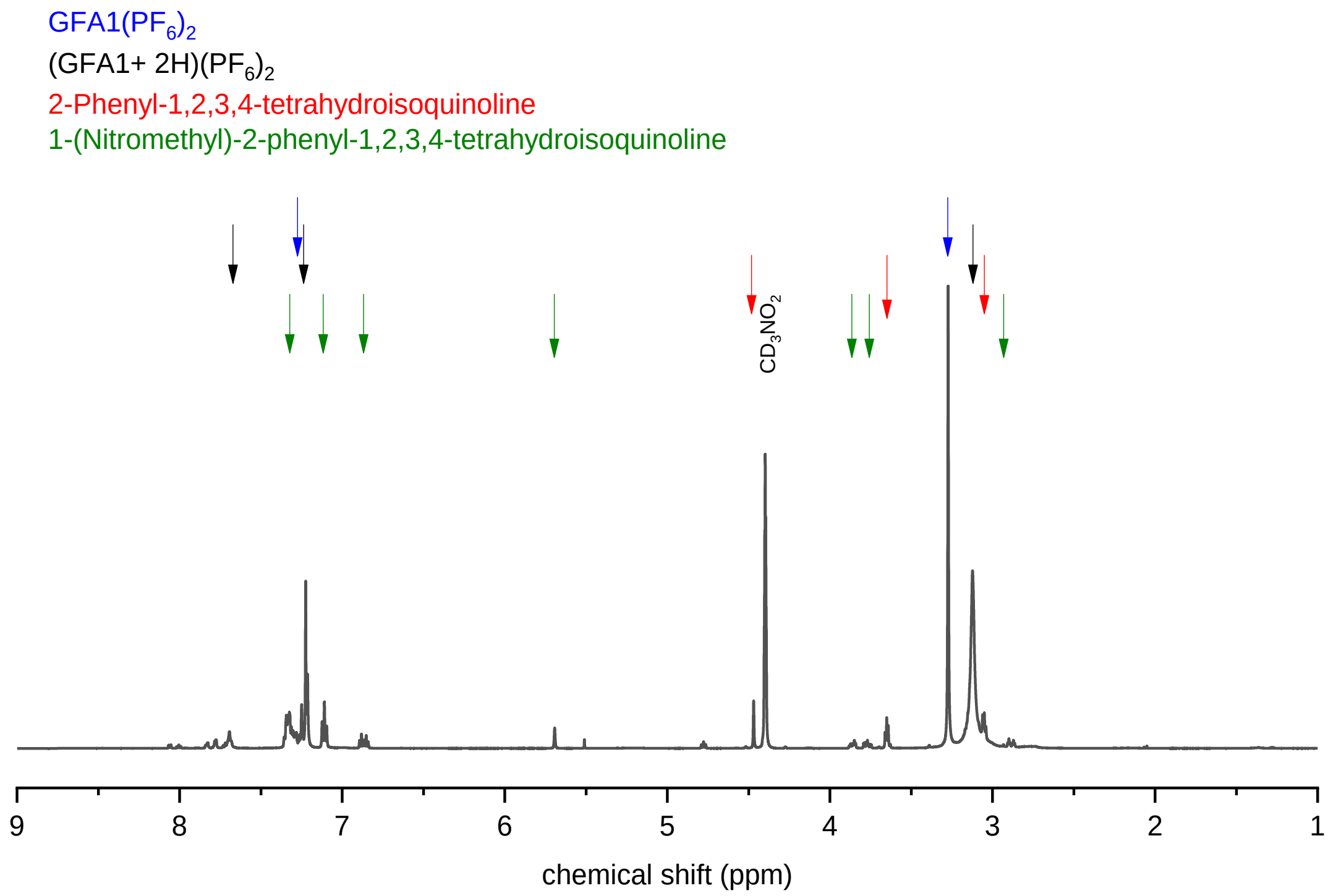

^1H NMR spectrum (600.18 MHz, CD_3NO_2, 298 K) for entry **C1** last measurement (3 h, r.t.)

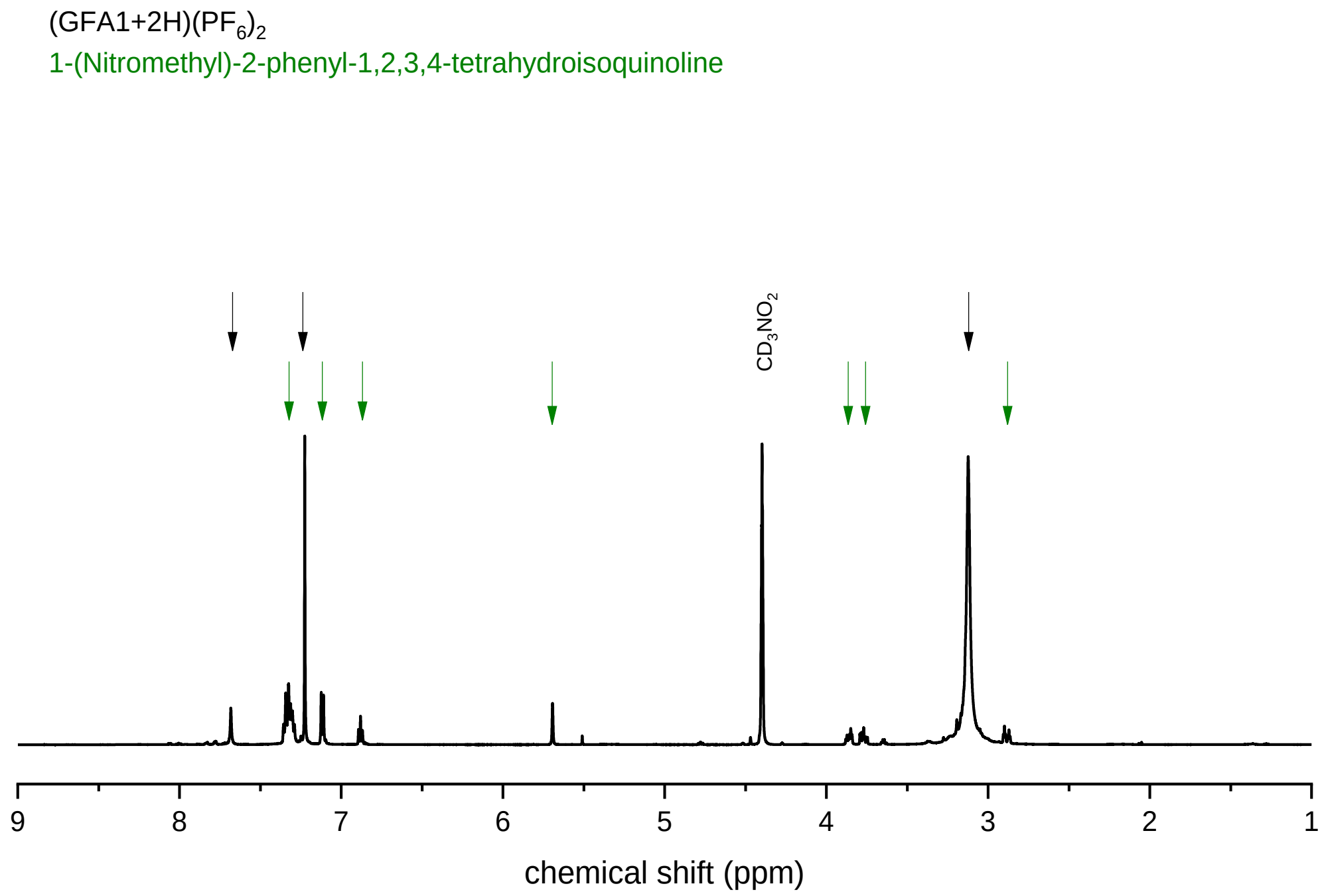

^1H NMR spectrum (600.18 MHz, CD_2Cl_2, 298.1 K) for entry **D1** after workup

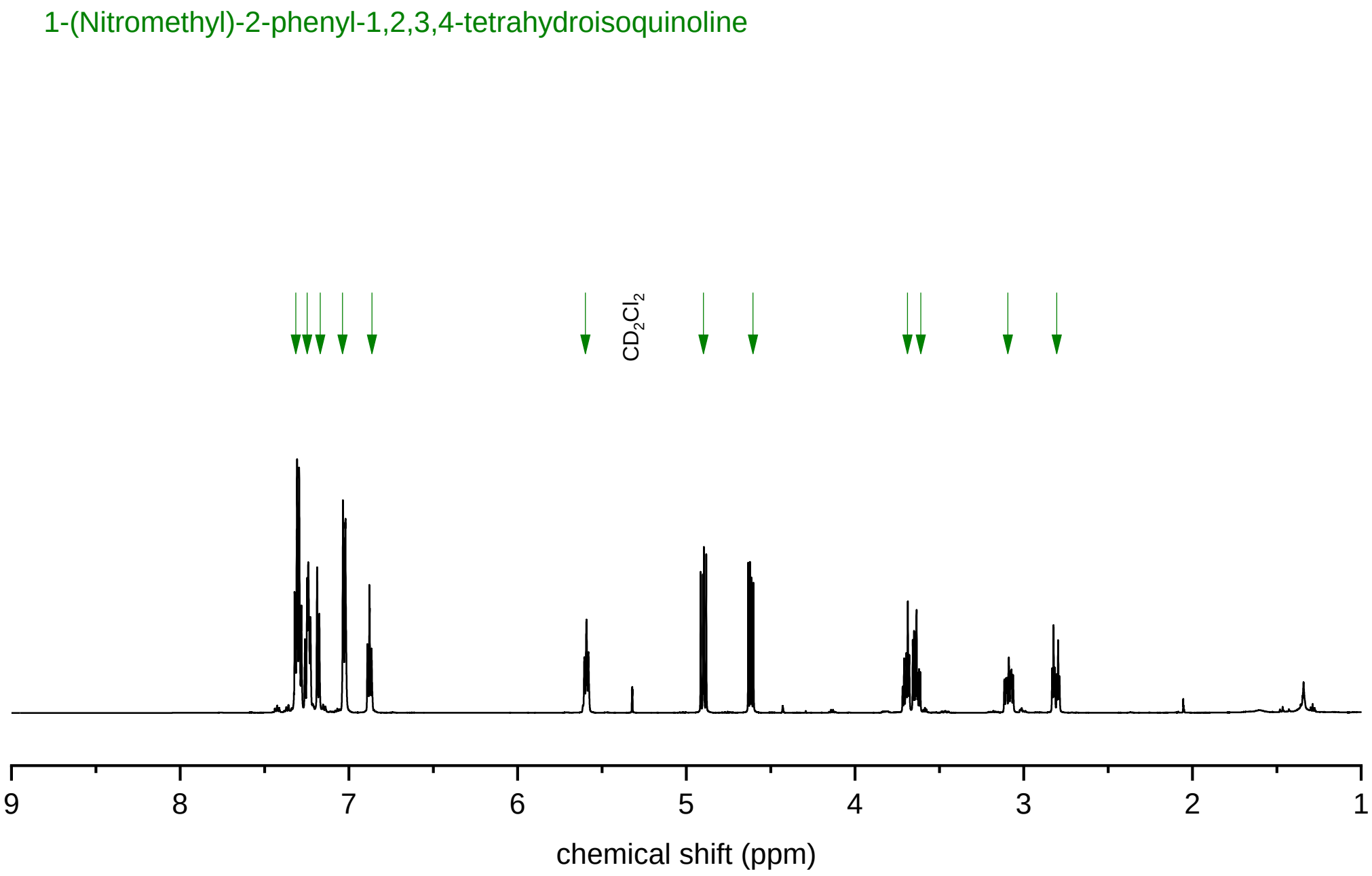

^{13}C NMR spectrum (150.95 MHz, CD_2Cl_2, 295 K) for entry **D1** after workup

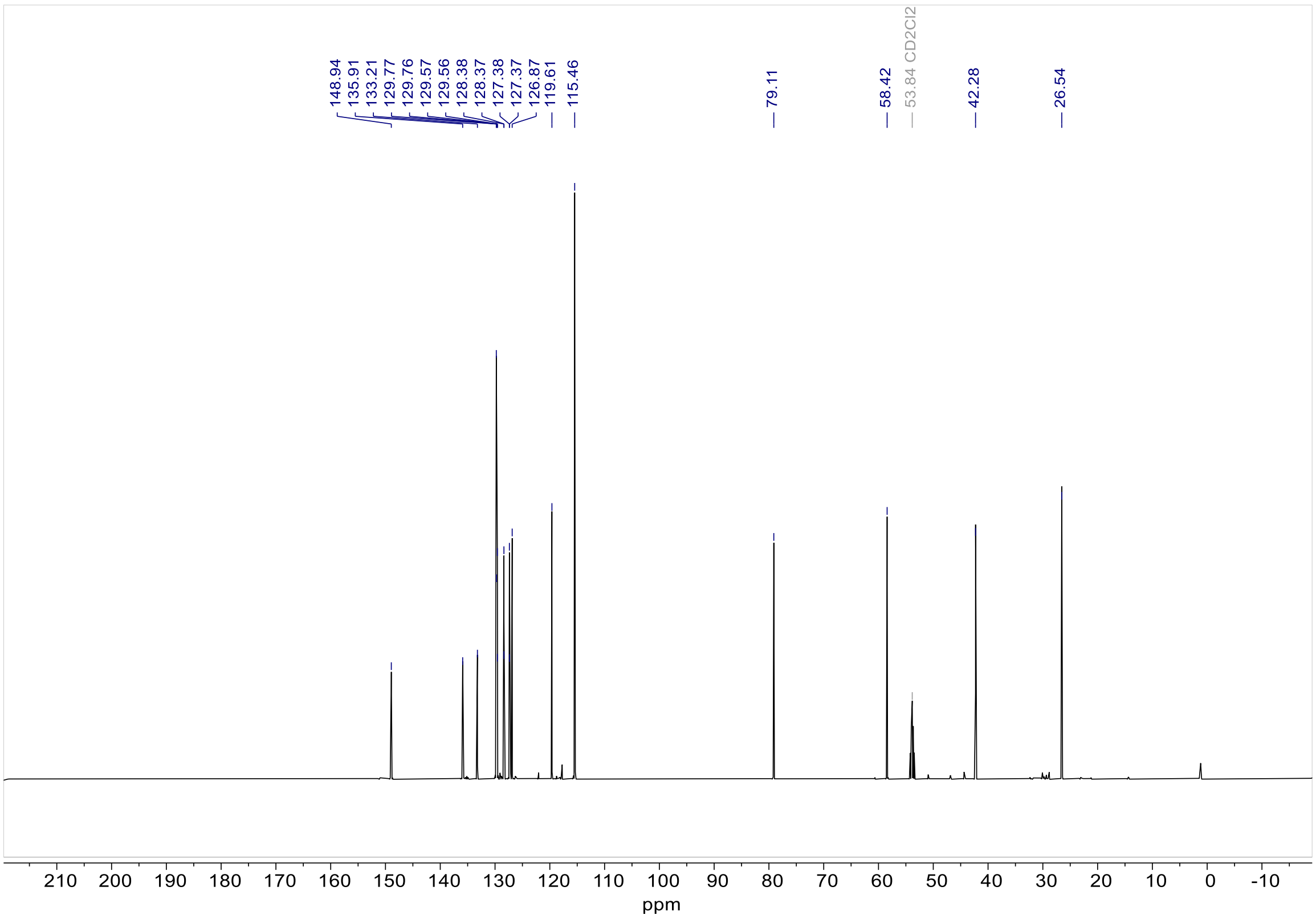

^1H NMR spectrum (600.15 MHz, CD_3CN, 298 K), for entry **D1**, separation of GFA1

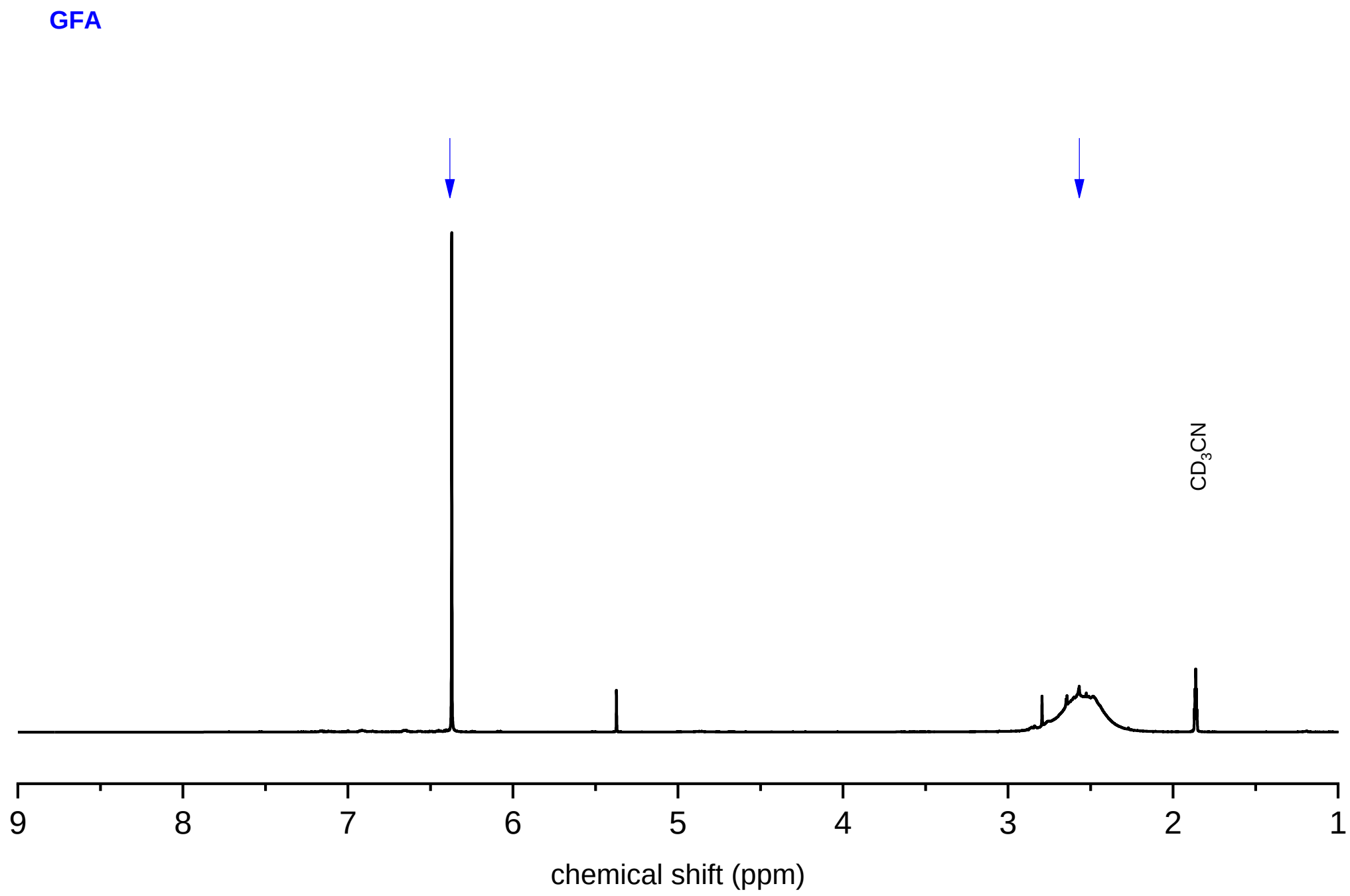

^{13}C NMR spectrum (150.91 MHz, CD_3CN, 295 K) for entry **D1**, separation of GFA1

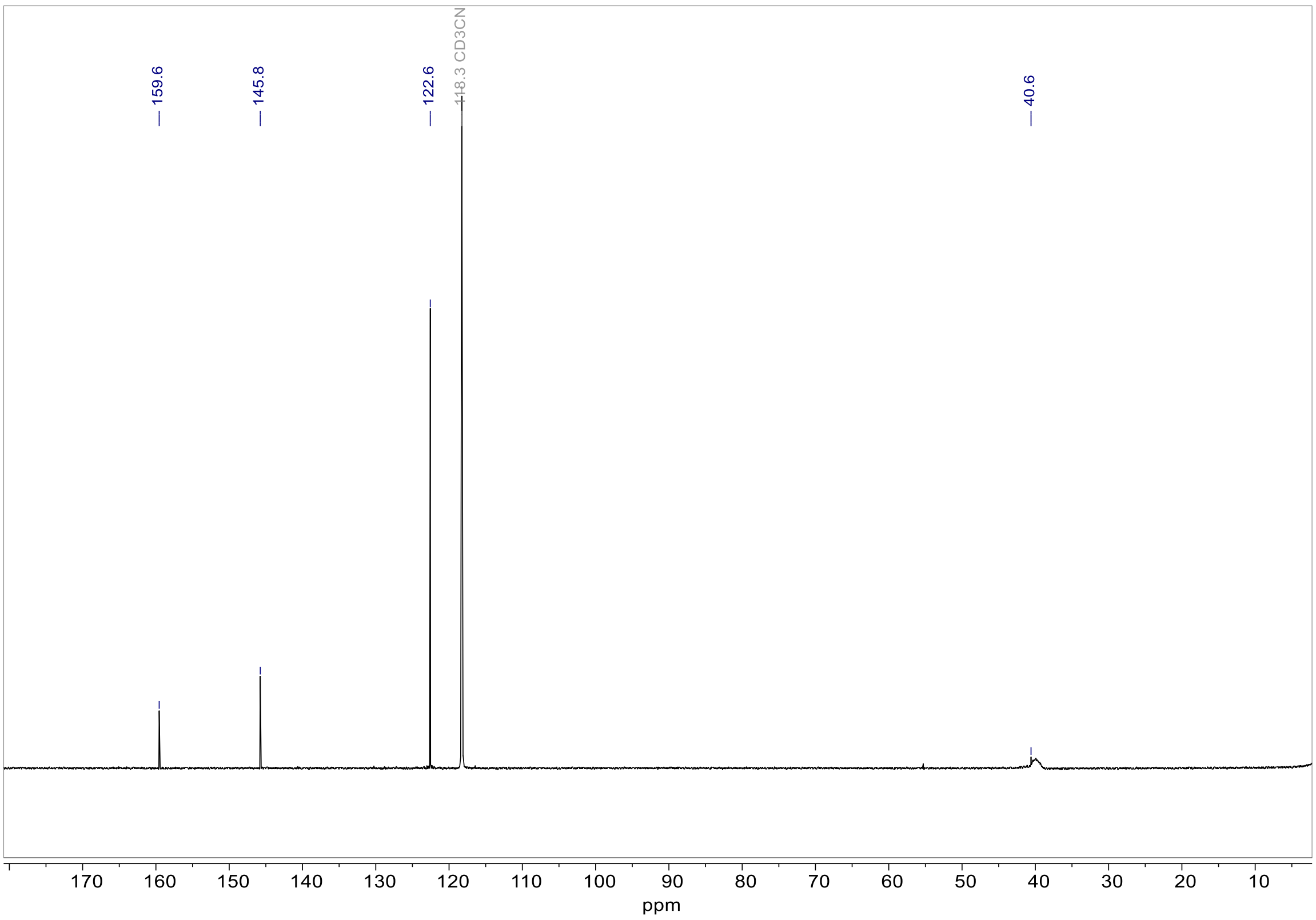

Reaction between 2-[4-(trifluoromethyl)phenyl]-1,2,3,4-tetrahydroisoquinoline (PTQ-CF_3) and nitromethane to give 1-(nitromethyl)-2-[4-(trifluoromethyl)phenyl-1,2,3,4-tetrahydro-isoquinoline

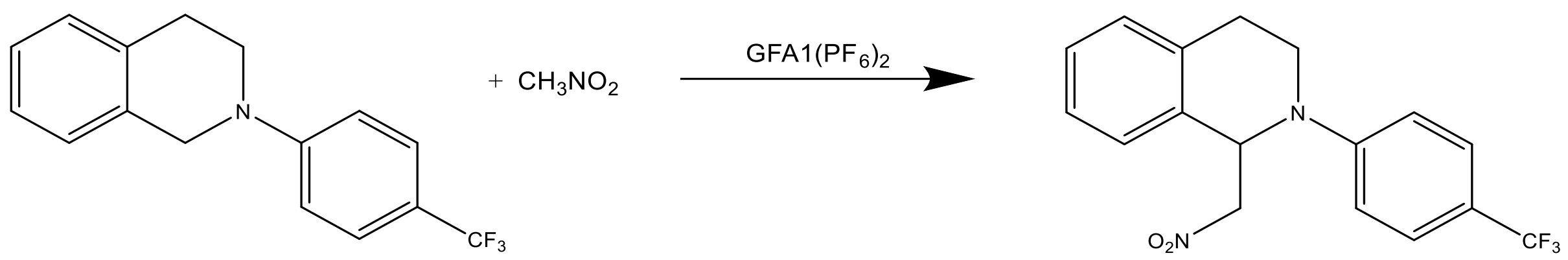

entry	E1a	E1b	F1
GFA1(PF_6)$_2$ [mg / µmol]	14.028 / 23.7	16.69 / 28.1	55.4 / 93
PTQ-CF_3 [mg / µmol]	5.192 / 18.7	5.07 / 18.3	18.9 / 68
nitromethane [ml]	0.5 (D3)	0.5 (D3)	1.0
ratio PTQ-CF_3 / $GFA1^{2+}$	1 / 1.3	1 / 1.5	1 / 1.5
condition	NMR tube	NMR tube	Schlenk flask
reaction time (temperature)	2 h (r.t.)	12 h (50 °C)	20 h (50 °C)
Yield after workup [%]	no conversion	93	78

Protocol for entry E1a: In an NMR tube, the two reactants PTQ-CF_3 and GFA1(PF_6)$_2$ were dissolved in CD_3NO_2. Conversion was followed by ^{1}H NMR spectroscopy.
Protocol for entry E1b: In an NMR tube, the two reactants PTQ-CF_3 and GFA1(PF_6)$_2$ were dissolved in CD_3NO_2. Conversion was followed by ^{1}H NMR spectroscopy. Then the excess of CD_3NO_2 was removed under high vacuum. The residue was suspended in CH_2Cl_2, filtered over syringe filter and concentrated to give 1-(nitromethyl)-2-[4-(trifluormethyl) phenyl]-1,2,3,4-tetrahydro-isoquinoline (5.63 mg, 93%) in high purity.
Protocol for entry F1: To a solution of GFA1(PF_6)$_2$ in half the amount of nitromethane, PTQ-CF_3 and the remaining solvent was added. The yellow-brown mixture was stirred at 50 °C for 20 h. Then nitromethane was removed under high vacuum. The residue was suspended in dichloromethane, filtered over syringe filter and concentrated to give 1-(nitromethyl)-2-[4-(trifluormethyl) phenyl]-1,2,3,4-tetrahydroisoquinoline (18.5 mg, mixture product / reactant = 95 / 5, 78%) as a pale-yellow oil. ^{1}H NMR (399.89 MHz, CD_2Cl_2, 295 K): δ = 7.52 (d, *J* = 8.7 Hz, 2 H, $CH_{arom.}$), 7.34 - 7.27 (m, *J* = 7.58 Hz, 1 H, $CH_{arom.}$), 7,24 (dd, *J* = 8.5, 7.1 Hz, 2 H, $CH_{arom.}$), 7.16 (d, *J* = 7.3 Hz, 1 H, $CH_{arom.}$), 7.05 (d, *J* = 8.8 Hz, 2 H, $CH_{arom.}$), 5.64 (s, 1 H), 4.90 (dd, *J* = 12.1, 7.8 Hz, 1 H), 4.63 (dd, *J* = 12.1, 6.8 Hz, 1 H), 3.7 (dd, *J* = 11.9, 6.71 Hz, 2 H), 3.11 (dd, *J* = 15.0, 7.2 Hz, 1 H), 2.89 (dt, *J* = 16.3, 5.5, 5.5 Hz, 1 H) ppm; ^{13}C NMR (100.56 MHz, CD_2Cl_2, 295 K): δ = 151.1, 135.6, 132.8, 129.5, 128.8, 127.4, 127.1, 127.1, 120.4, 120.0, 113.6, 78.8, 58.0, 42.2, 26.9 ppm. The analytical data matched those in the literature.[7]

Separation of (GFA1+2H)(PF_6)$_2$: The insoluble components consisted of (GFA1+2H)(PF_6)$_2$ in high purity (for entry **F1**, 22.6 mg, 56%; for entry **E1b**, 11.04 mg, 66%) as an off-white solid.

^{1}H NMR spectrum (600.18 MHz, CD_3NO_2, 298 K) for entry **E1** first measurement (10 min, r.t.)

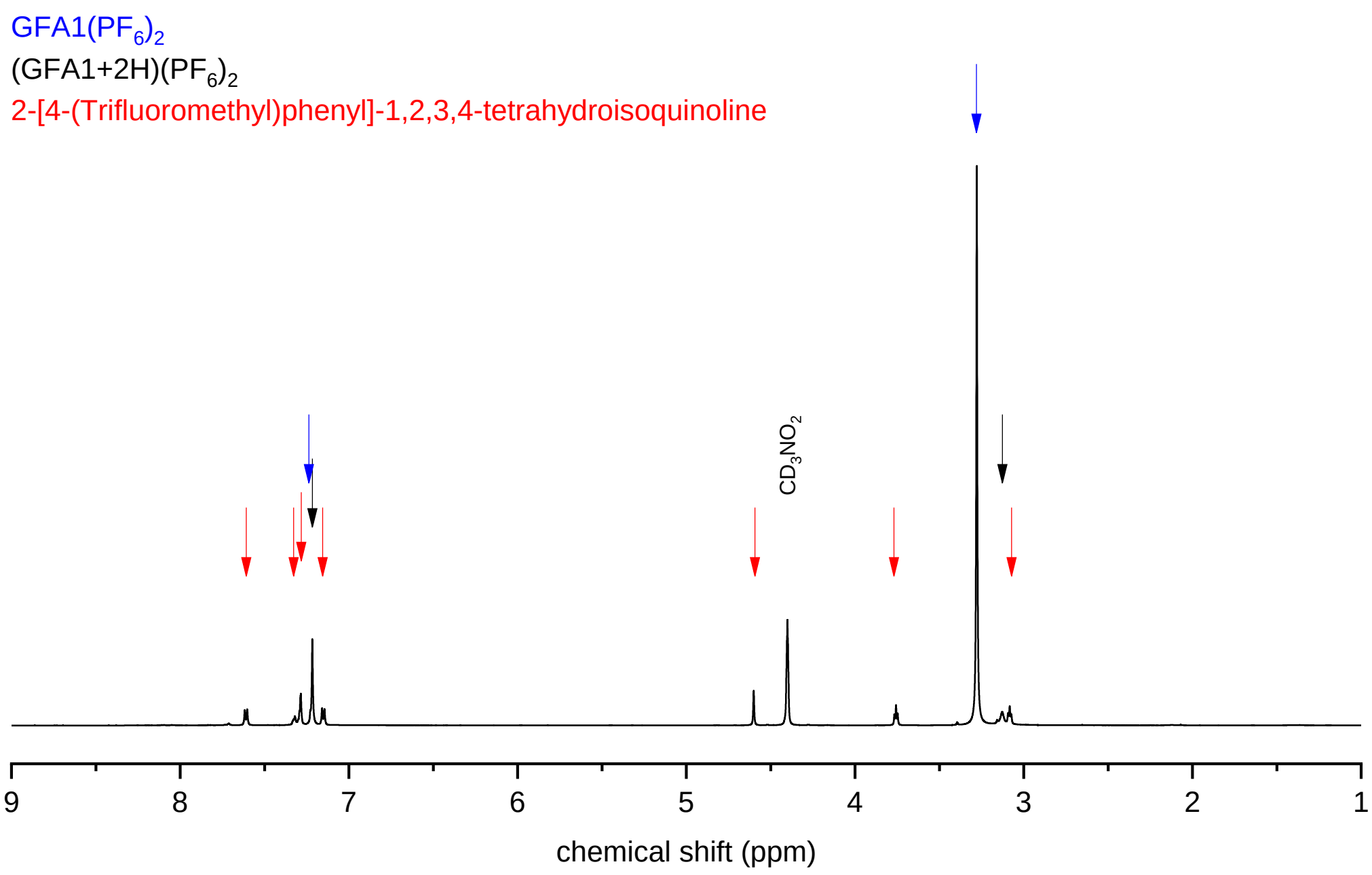

^{1}H NMR spectrum (600.18 MHz, CD_3NO_2, 298 K) for entry **E1** last measurement (12 h, 50 °C)

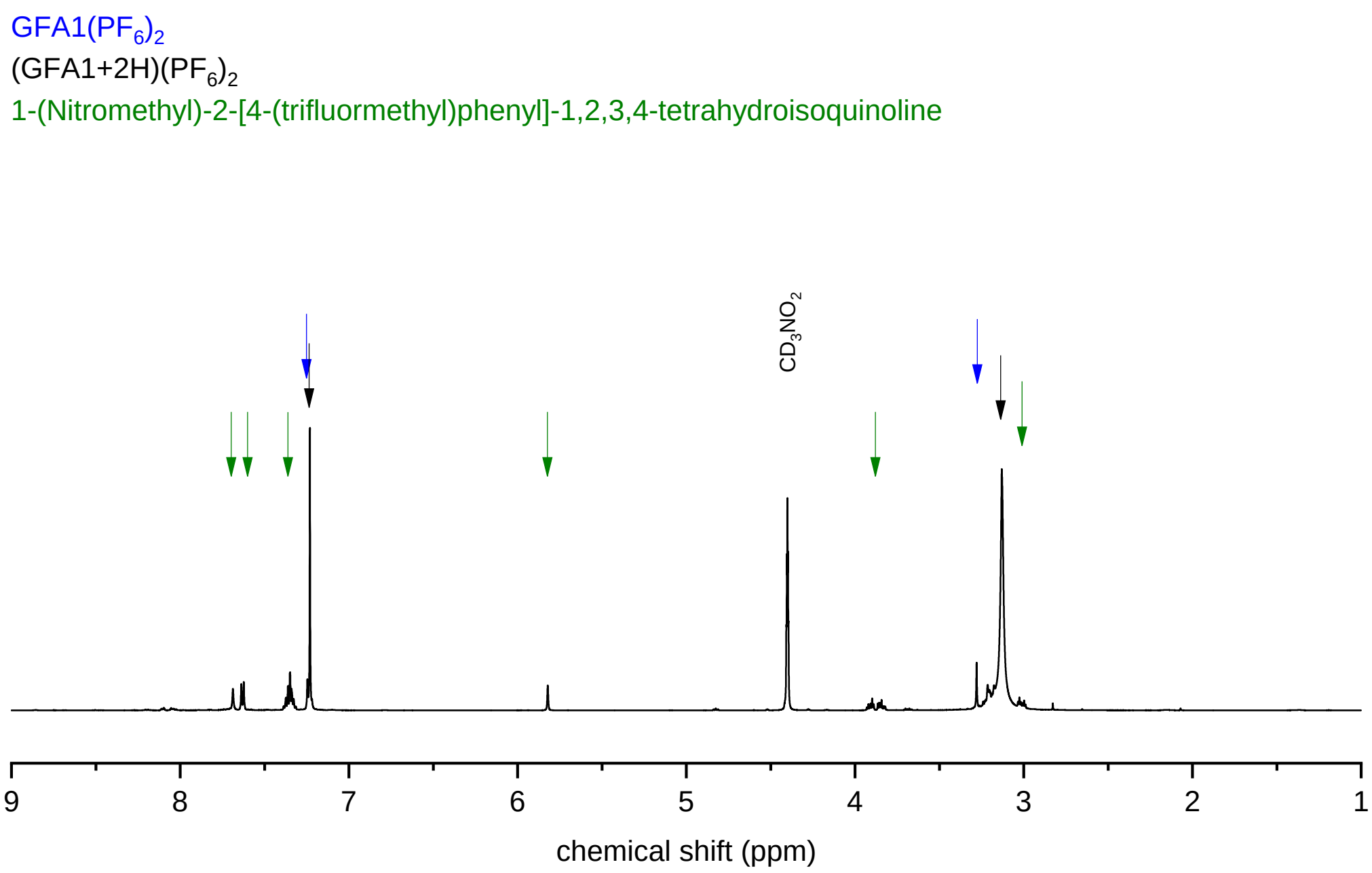

^{1}H NMR spectrum (600.18 MHz, $CDCl_3$, 298 K) for entry **E1** after workup

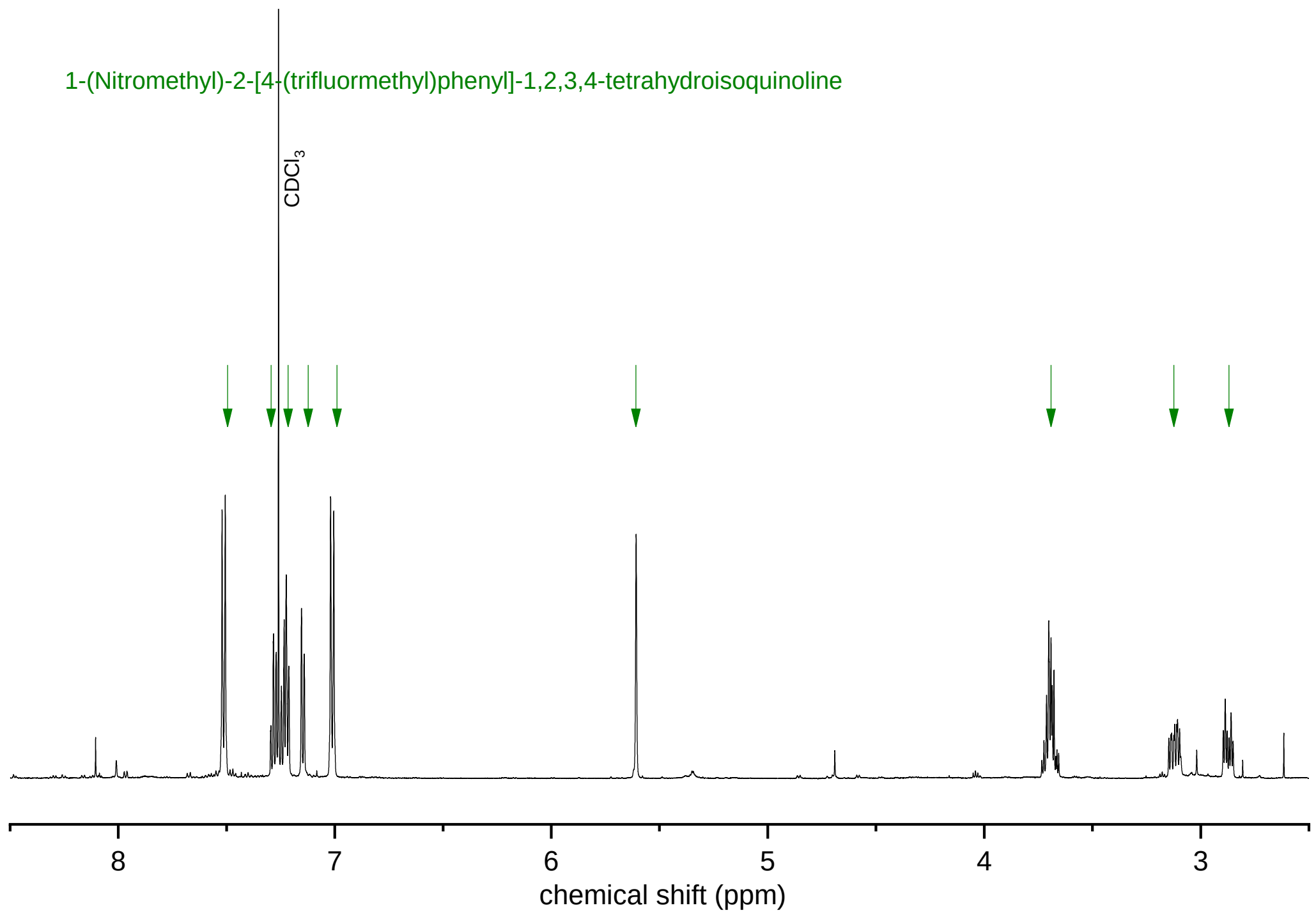

^{1}H NMR spectrum (399.89 MHz, CD_2Cl_2, 295 K) for entry **F1** after workup

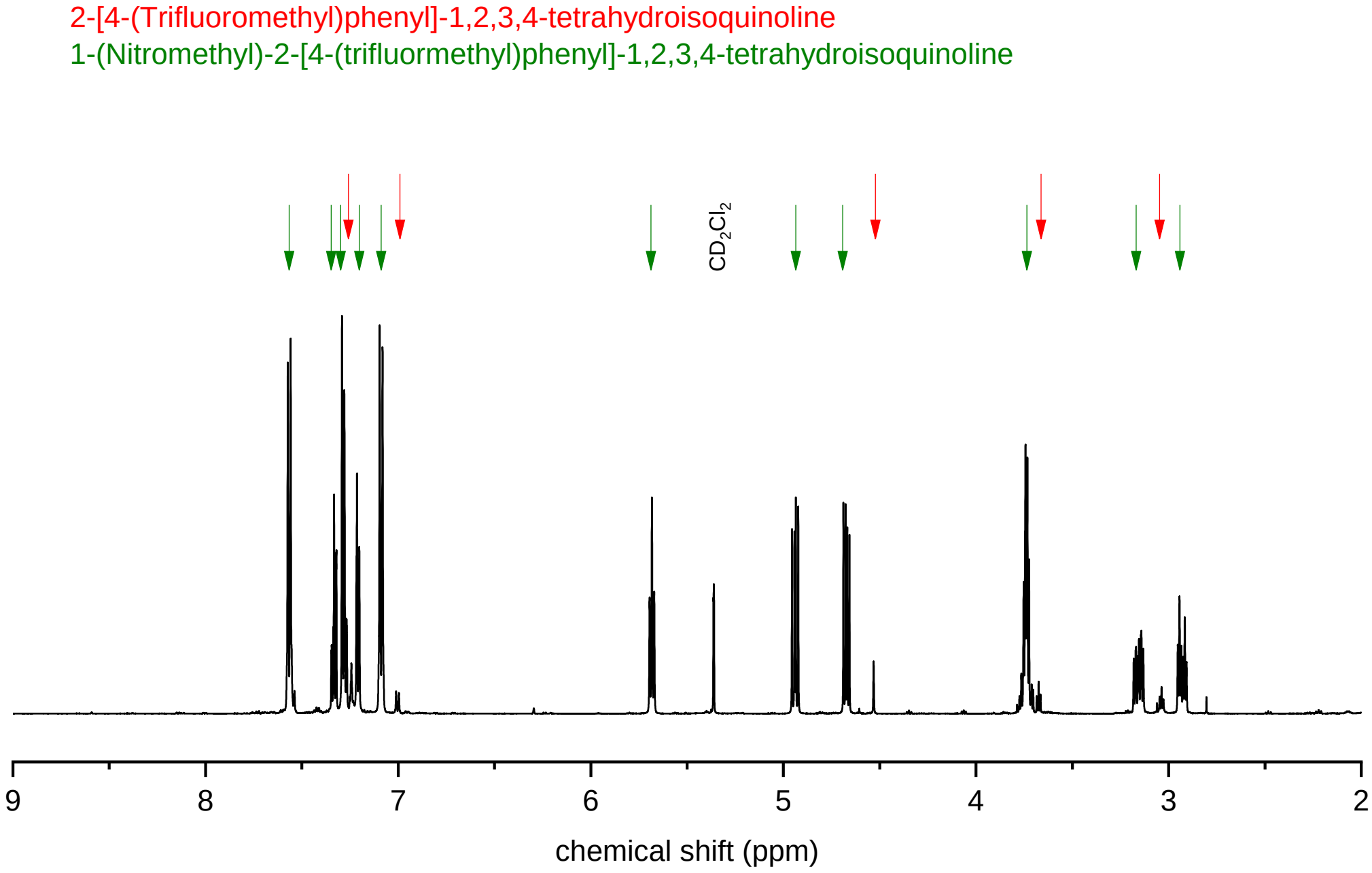

^{13}C NMR spectrum (100.56 MHz, CD_2Cl_2, 295 K) for entry **F1** after workup

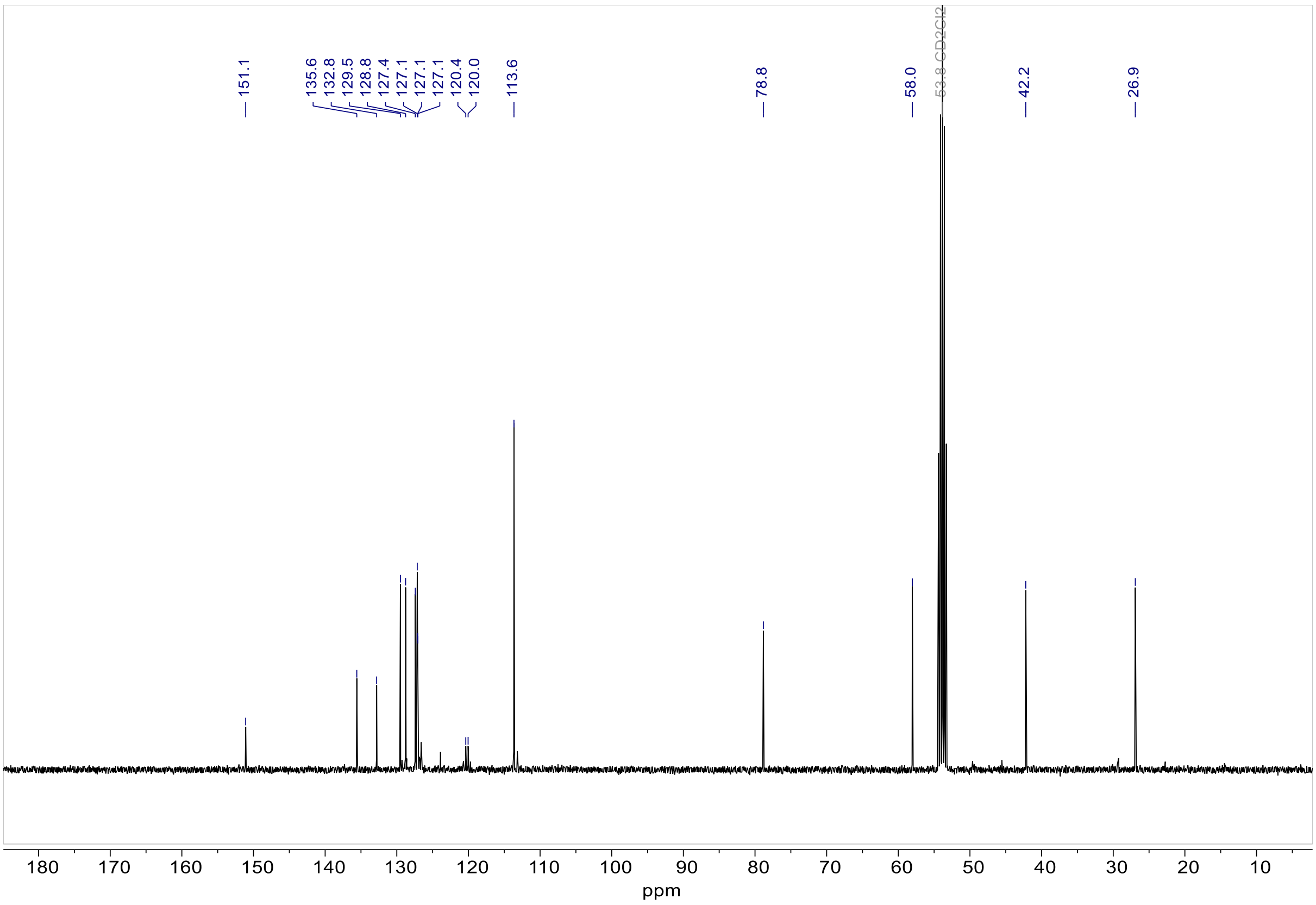

^{1}H NMR spectra for entry **F1** after workup

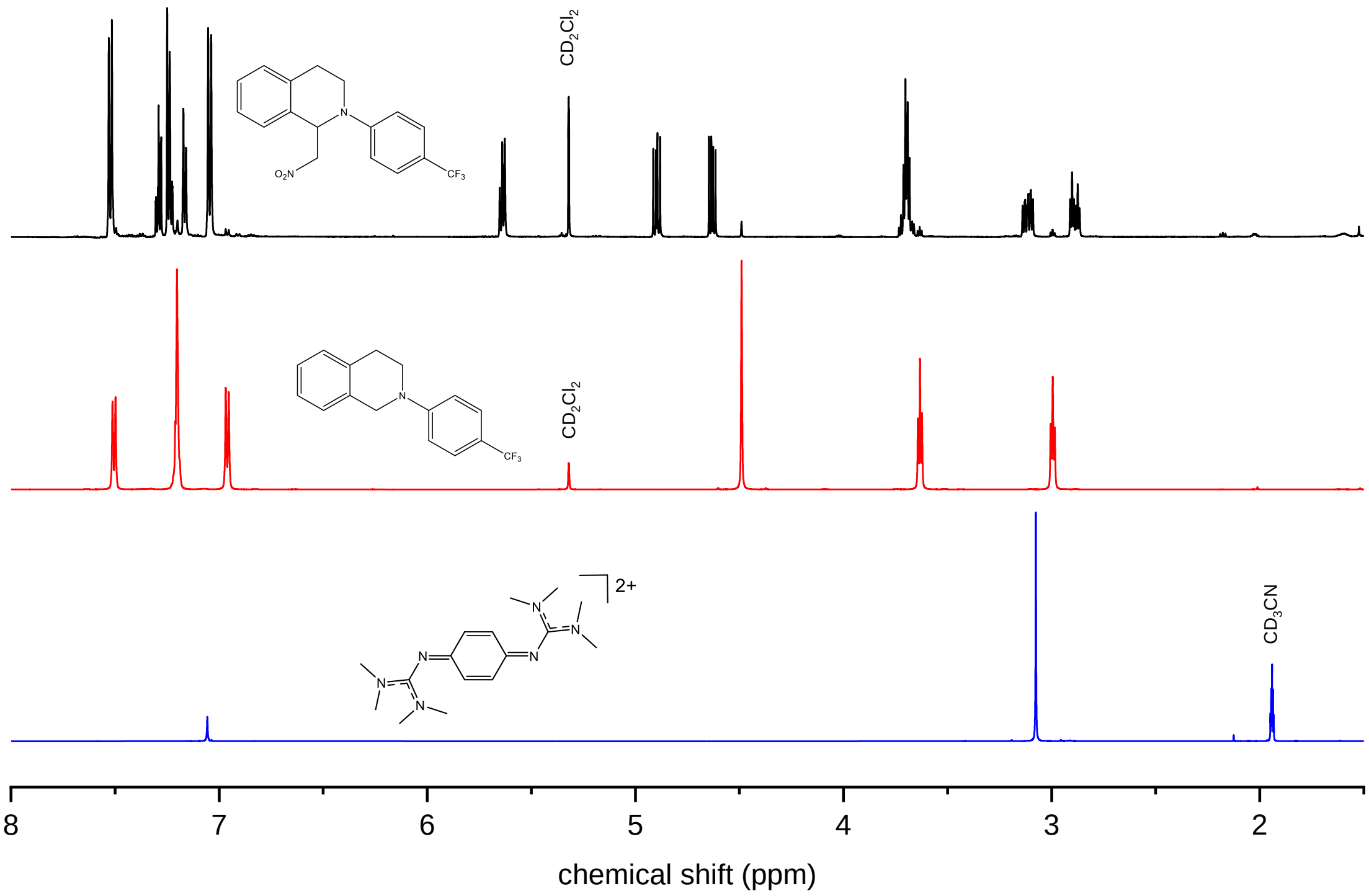

Reaction with DDQ as CDC reagent for comparison

entry	G1
DDQ [mg / µmol]	5.899 / 26.0
PTQ-CF_3 [mg / µmol]	5.091 / 18.4
nitromethane [ml]	0.5
ratio PTQ-CF_3 / DDQ	1 / 1.4
condition	Schlenk flask
reaction time (temperature)	50 °C (20 h)
conversion to 1(nitromethyl)-2-[4-(trifluoromethyl) phenyl-1,2,3,4-tetrahydroisoquinoline	no conversion

Protocol for entry G1: In a Schlenk flask, the two reactants PTQ-CF_3 and DDQ were dissolved in nitromethane. The reaction was stirred for 20 h at 50 °C. Then the reaction was stopped by the addition of an aqueous saturated $NaHCO_3$ solution (2 ml). Subsequently the excess nitromethane was removed under vacuum. The obtained solution was extracted several times with CH_2Cl_2. The combined organic phases were dried over $MgSO_4$, filtered and concentrated.

^{1}H NMR spectra (600.18 MHz, 298.1 K) for entry **G1**

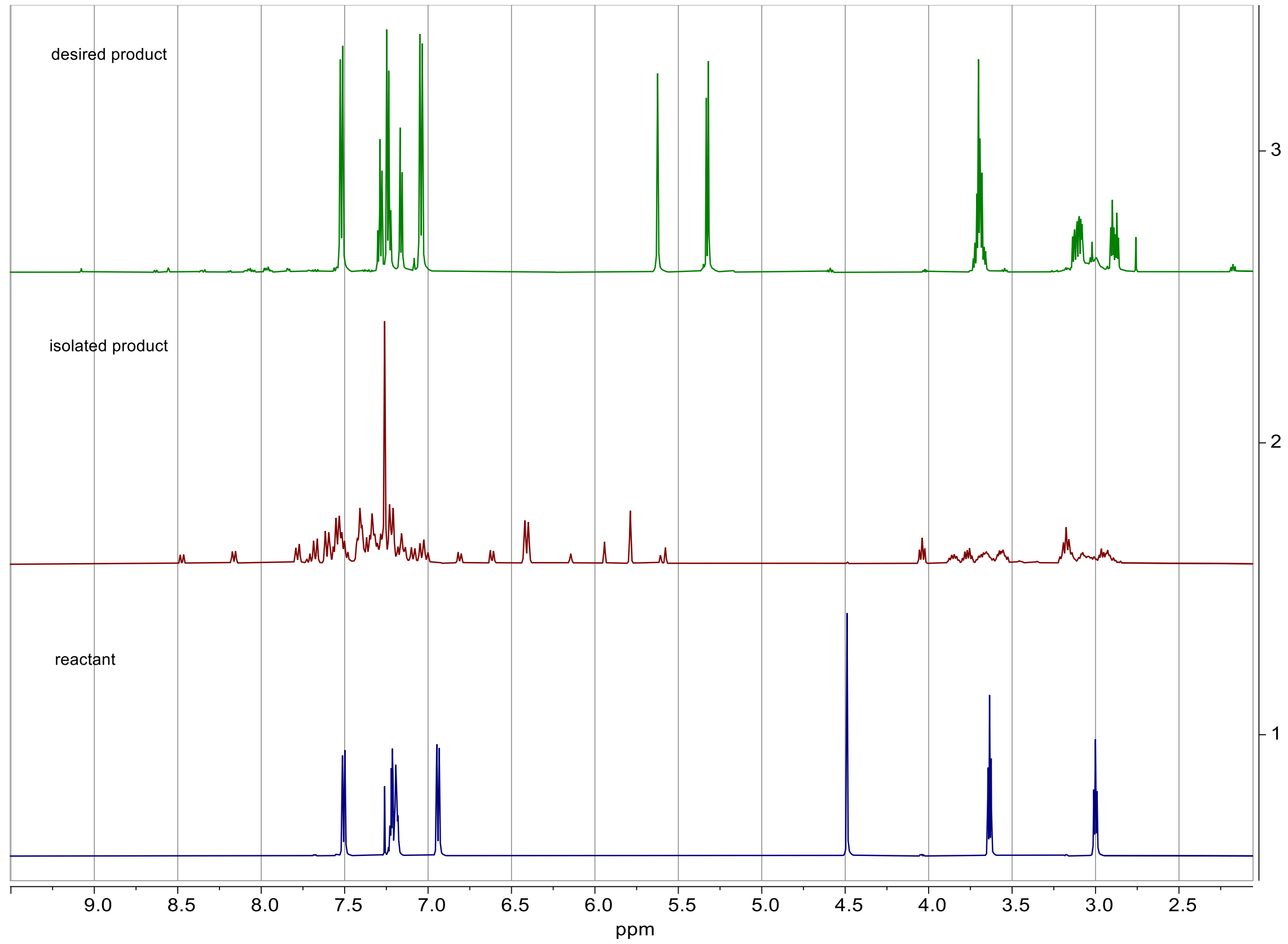

Reaction between 2-(4-methoxyphenyl)-1,2,3,4-tetrahydroisoquinoline (PTQ-OMe) and nitromethane to give 1-(nitromethyl)-2-(4-methoxyphenyl) -1,2,3,4-tetrahydroisoquinoline

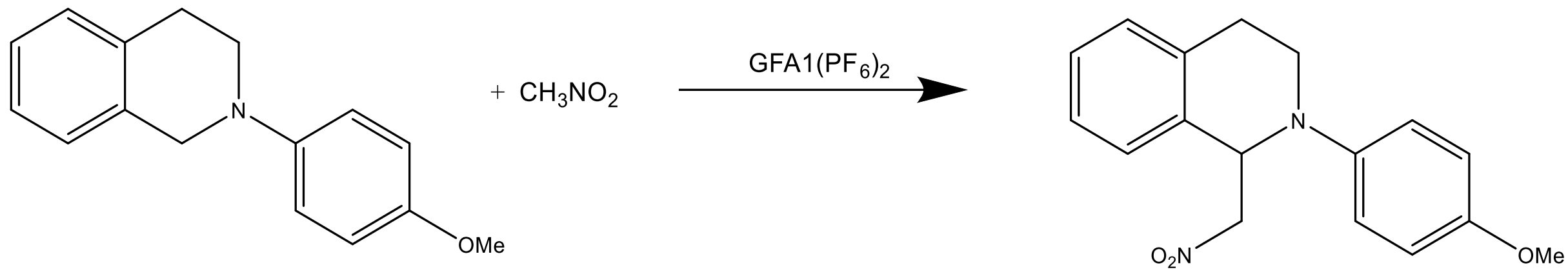

entry	H1	J1
$GFA1(PF_6)_2$ [mg / µmol]	14.60 / 24.5	64.0 / 108
PTQ-OMe [mg / µmol]	4.66 / 19.5	22.0 / 92
nitromethane [ml]	0.5 (D3)	1.0
ratio PTQ-OMe / $GFA1^{2+}$	1 / 1.3	1 / 1.2
condition	NMR tube	Schlenk flask
reaction time (temperature)	10 min (r.t.)	2 h (r.t.)
Yield after workup [%]	96 [a]	85

[a] Without workup, ratio product / reactant from NMR spectroscopie

Protocol for entry H1: In an NMR tube, the two reactants PTQ-OMe and $GFA1(PF_6)_2$ were dissolved in CD_3NO_2. Conversion was followed by ^1H NMR spectroscopy. Then, the excess CD_3NO_2 was removed under high vacuum. The residue was suspended in CH_2Cl_2, filtered over syringe filter and concentrated.

Protocol for entry J1: To a solution of $GFA1(PF_6)_2$ in half the amount of nitromethane, PTQ-OMe and the remaining solvent was added. The yellow-brown mixture was stirred at room temperature for 2 h. Then, nitromethane was removed under high vacuum. The residue was suspended in dichloromethane and filtered through a syringe filter. The solution was concentrated to 1 ml and layered with petroleum ether. The precipitated yellow oil was decanted and dried in vacuo to obtain the desired product (23.5 mg, mixture product / reactant = 94 / 6, 85%). ^1H NMR (399.89 MHz, CD_2Cl_2, 295 K): δ = 7.21 (m, *J* = 29.13, 10.23 Hz, 4 H, $CH_{arom.}$), 6.92 (dd, *J* = 9.11 Hz, 2 H, $CH_{arom.}$), 6.81 (dd, *J* = 9.11 Hz, 2 H, $CH_{arom.}$), 5.39 (dd, J = 8.82, 5.71 Hz, 1 H), 4.84 (dd, *J* = 12.06, 8.89 Hz, 1 H), 4.60 (dd, *J* = 12.07, 5.67 Hz, 1 H), 3.73 (s, 3 H), 3.58 (m, *J* = 10.09, 4.27 Hz, 2 H), 2.98 (ddd, *J* = 5.48, 5.48 Hz, 1 H), 2.69 (dt, *J* = 16.59, 3.90, 3.90 Hz, 1 H) ppm; ^{13}C NMR (100.56 MHz, CD_2Cl_2, 295 K): δ = 154.3, 143.5, 136.0, 133.2, 129.8, 128.2, 127.3, 126.8, 119.1, 114.9, 79.3, 59.1, 55.8, 43.4, 25.9 ppm. The analytical data matched those in the literature.[7]

Separation of (GFA1+2H)(PF_6)$_2$ for entry J1: the insoluble components consisted of (GFA1+2H)(PF_6)$_2$ in high purity (54 mg, 98%).

^{1}H NMR spectrum (600.18 MHz, CD_3NO_2, 298 K) for entry **H1** (10 min, r.t.)

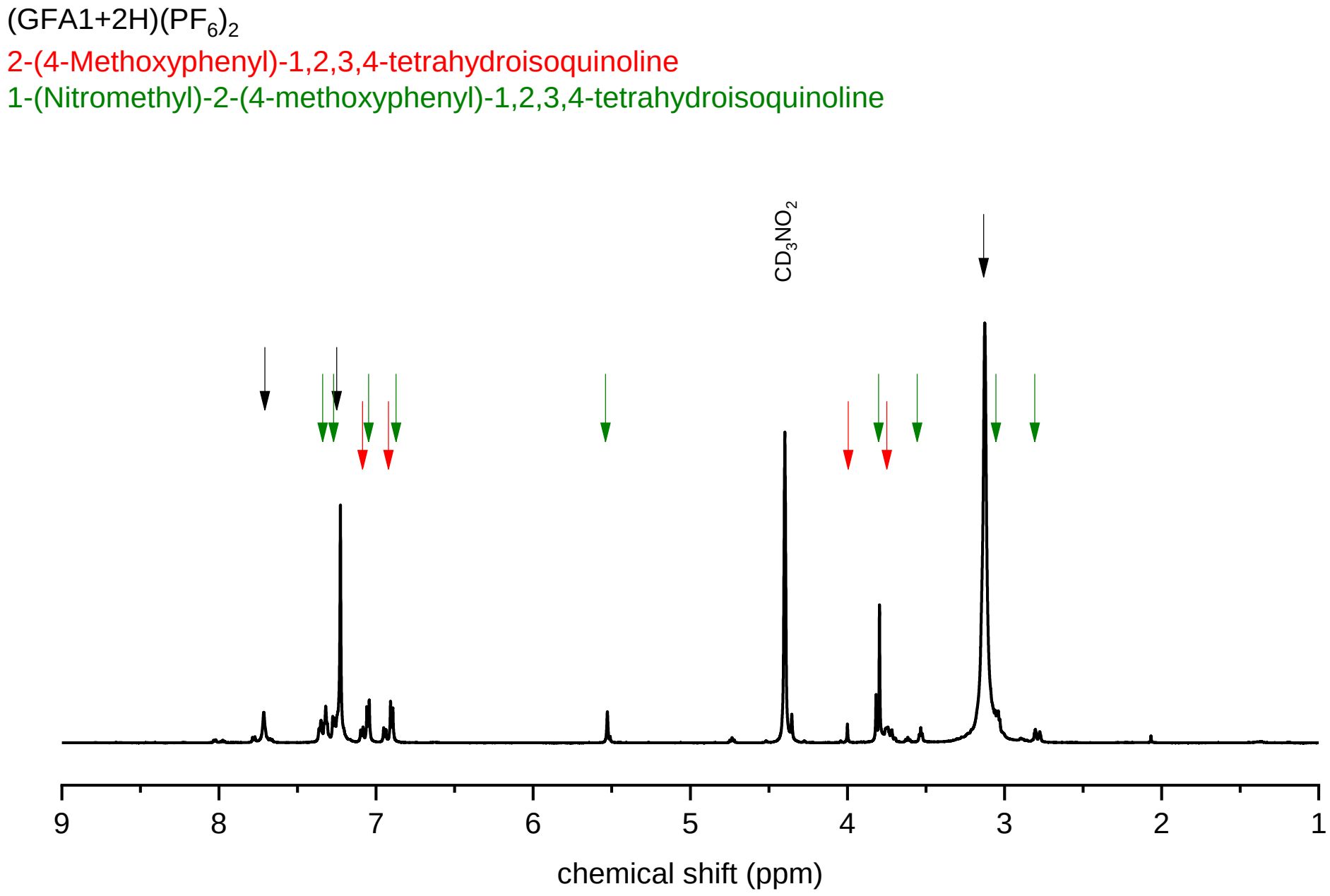

^{1}H NMR spectrum (600.18 MHz, CD_2Cl_2, 298 K) for entry **H1** after workup

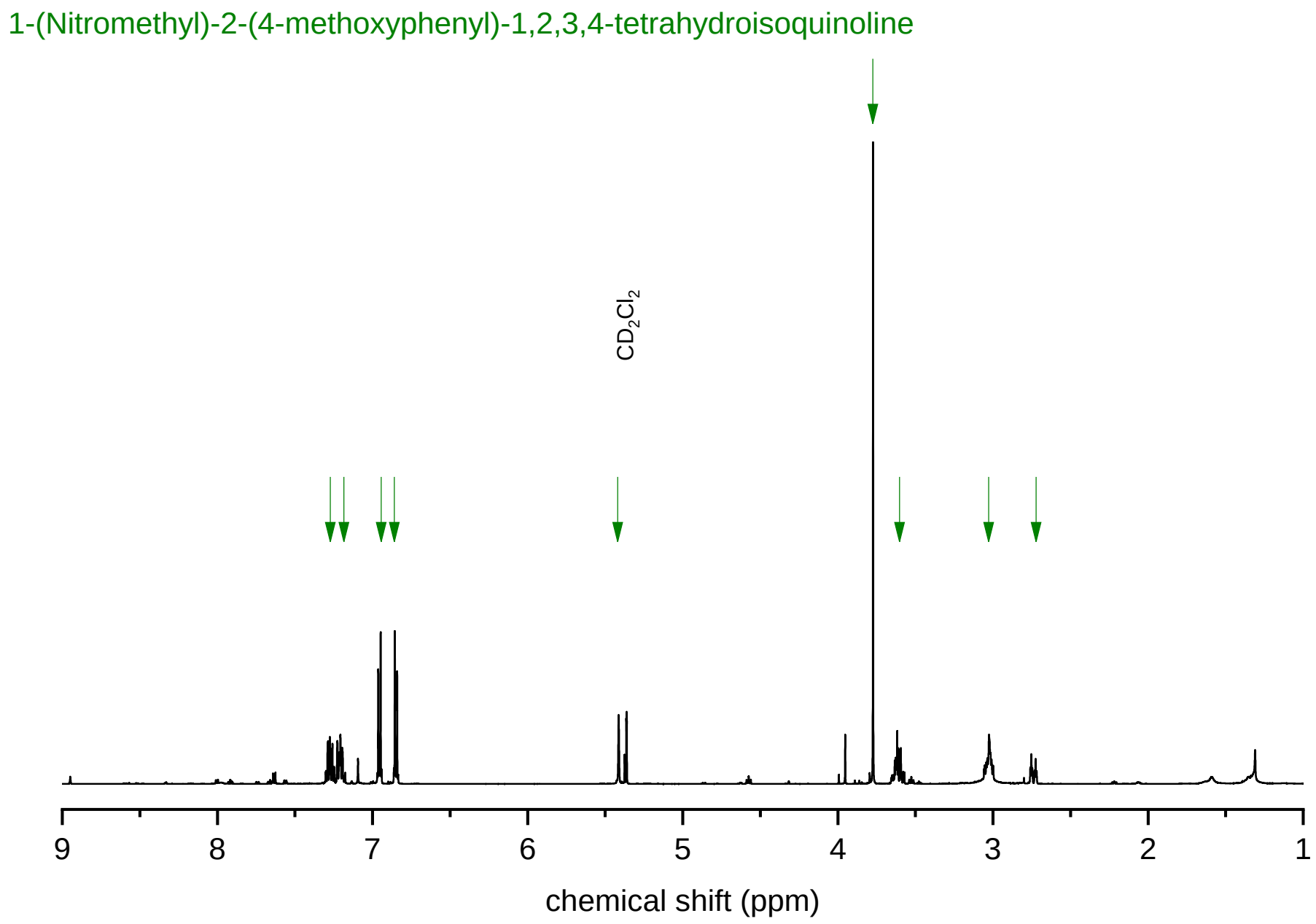

^1H NMR spectrum (399.89 MHz, CD_2Cl_2, 295 K) for entry **J1** after workup

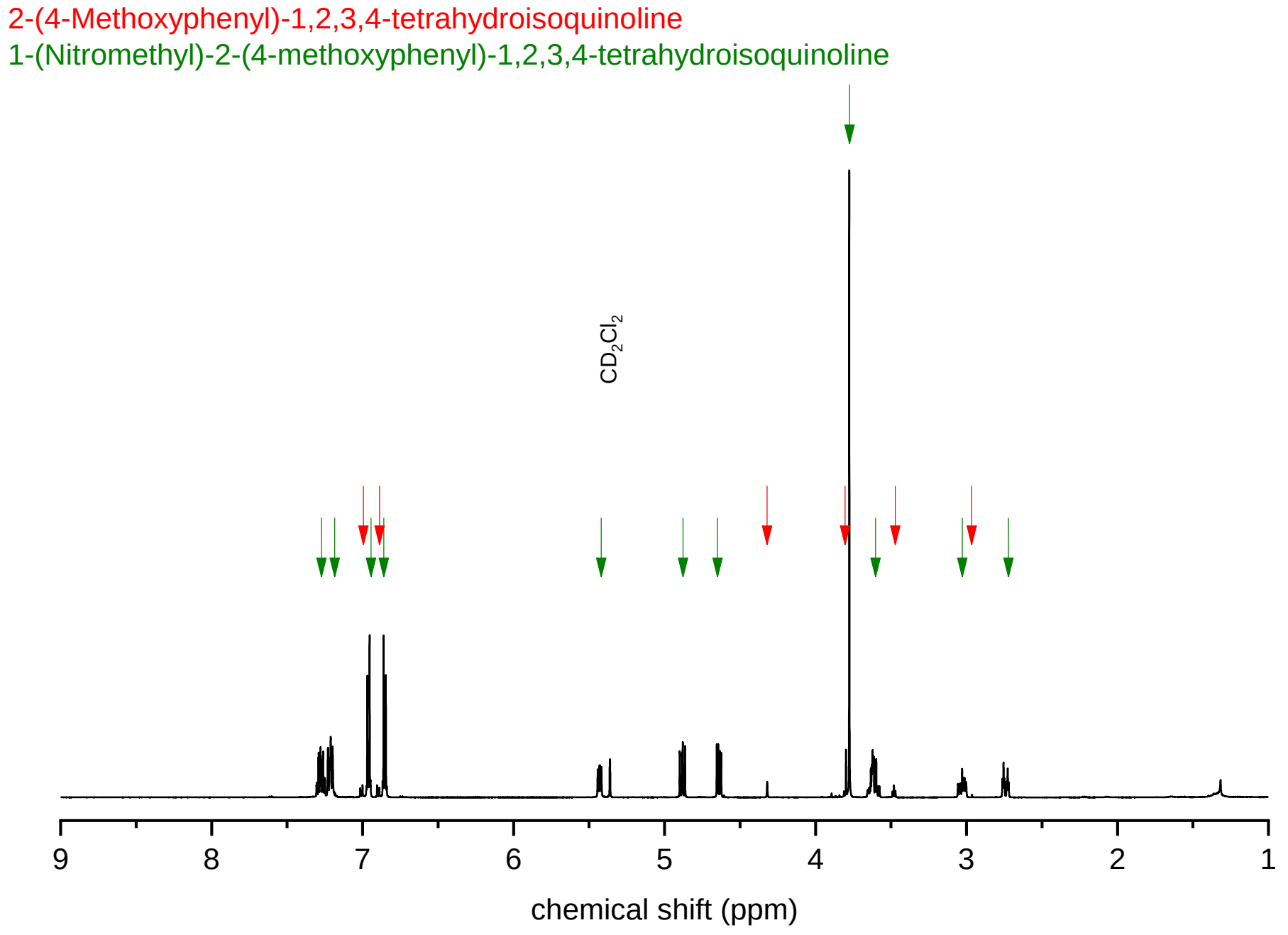

^{13}C NMR spectrum (100.55 MHz, CD_2Cl_2, 295 K) for entry **J1** after workup

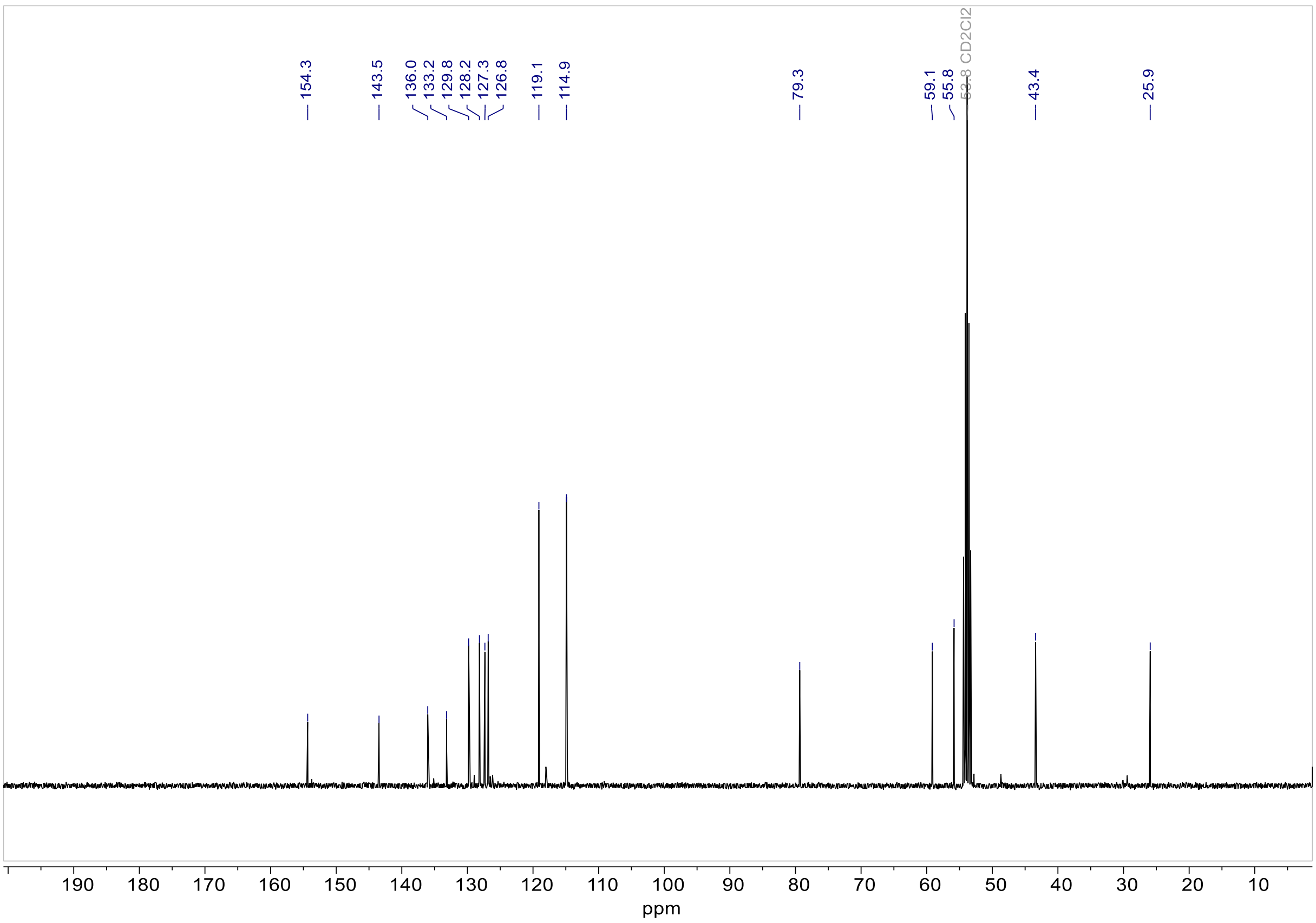

^{1}H NMR spectra for entry **J1** after workup

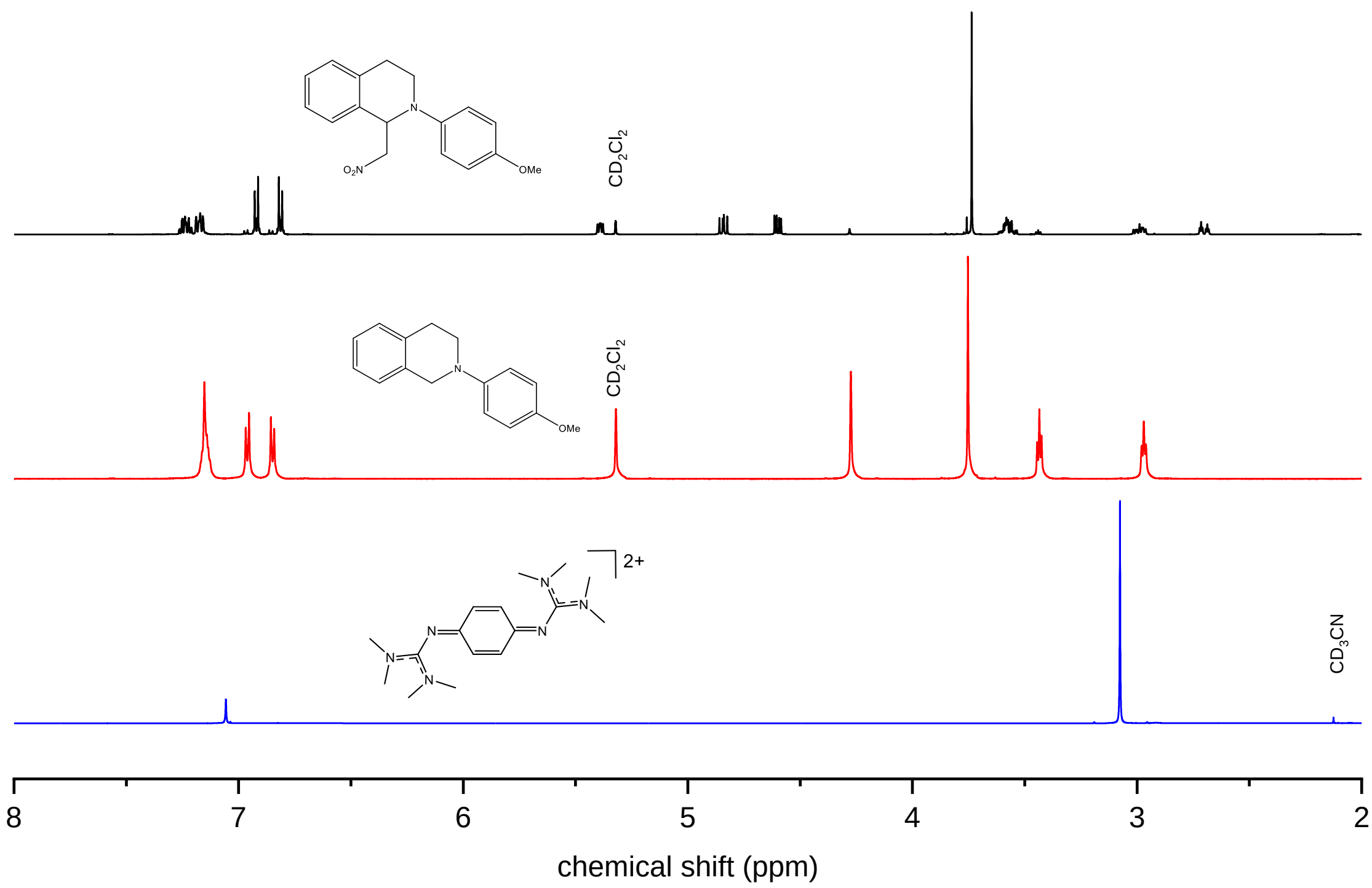

^{1}H NMR spectrum (600.15 MHz, CD_3CN, 298 K) for entry **J1**, separation of (GFA1+2H)$(PF_6)_2$

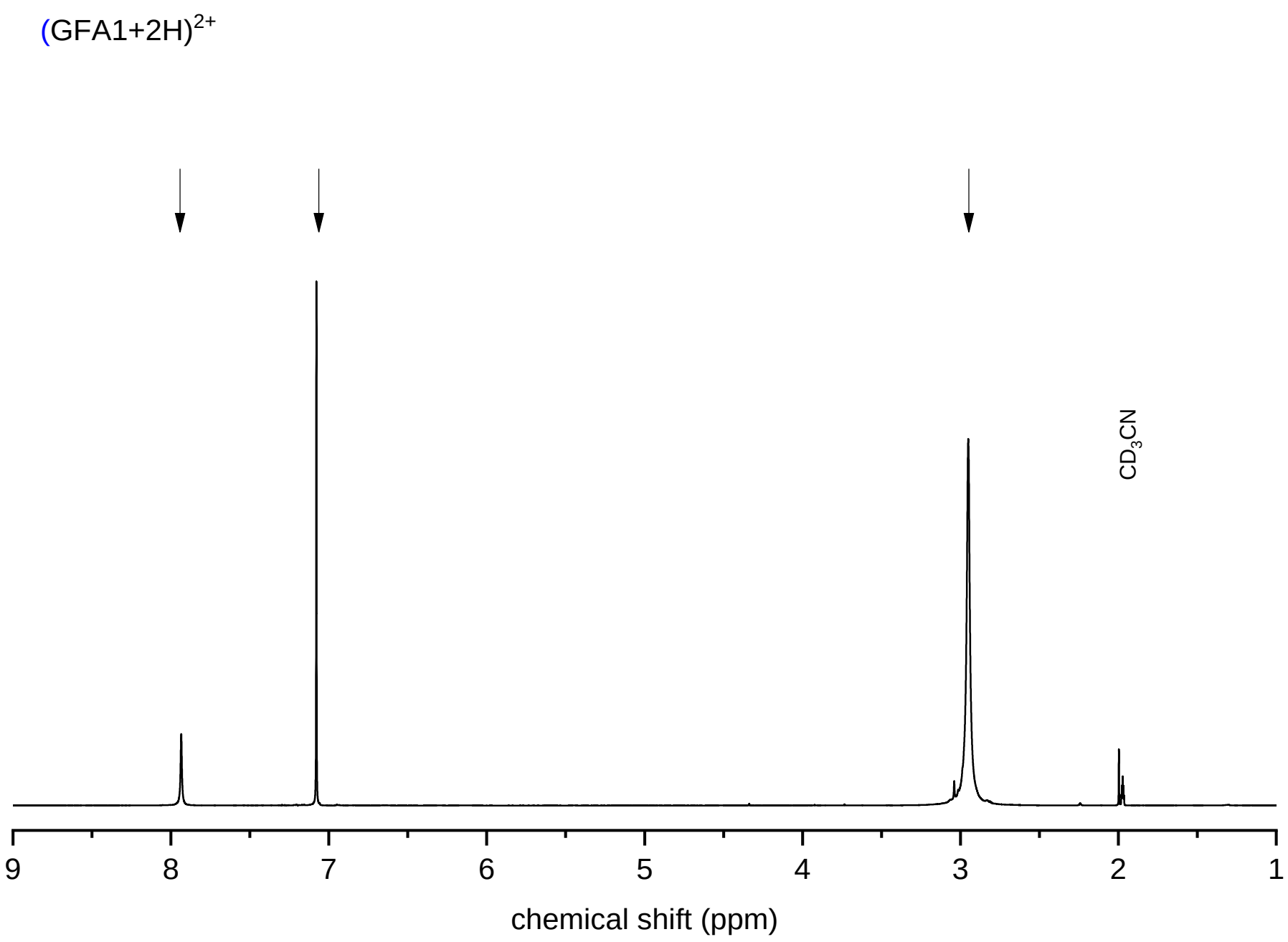

Reaction between 2-(p-tolyl)-1,2,3,4-tetrahydroisoquinoline (PTQ-Me) and nitromethane to give 1-(nitromethyl)-2-(p-tolyl) -1,2,3,4-tetrahydro-isoquinoline

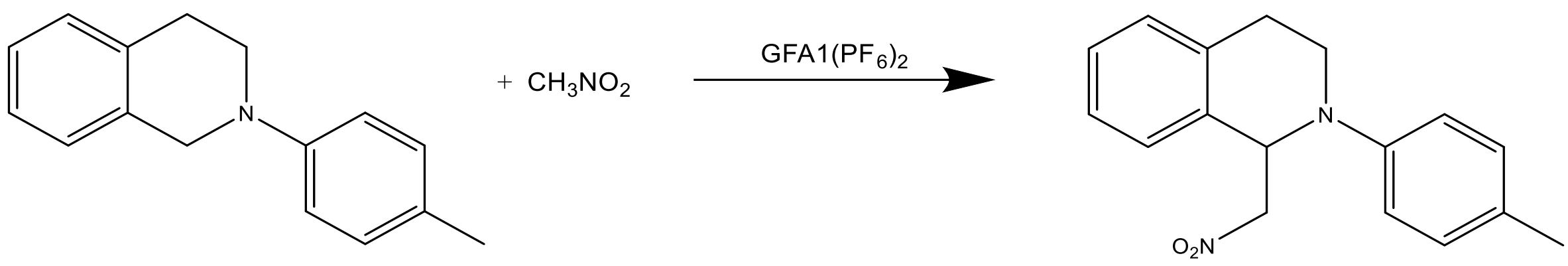

entry	K1	L1
GFA1(PF_6)$_2$ [mg / µmol]	15.524 / 26	48.896 / 82
PTQ-Me [mg / µmol]	5.786 / 23	16.144 / 72
nitromethane [ml]	0.5 (D3)	0.8 (D3)
ratio PTQ-Me / GFA1^{2+}	1 / 1.1	1 / 1.1
condition	NMR tube	Schlenk flask
reaction time (temperature)	1.5 h (r.t.)	2 h (r.t.)
Yield after workup [%]	85	95

Protocol for entry K1: In an NMR tube, the two reactants PTQ-Me and GFA1(PF_6)$_2$ were dissolved in CD_3NO_2. Conversion was followed by ^{1}H NMR spectroscopy. Then the excess of nitromethane (D3) was removed under high vacuum. The residue was suspended in CH_2Cl_2, filtered over syringe filter and concentrated to give 1-(nitromethyl)-2-(p-tolyl)-1,2,3,4-tetrahydro-isoquinoline (5.522 mg, 85%) as a yellow oil.

Protocol for entry L1: To a solution of GFA1(PF_6)$_2$ in half the amount of nitromethane, PTQ-Me and the remaining solvent was added. The yellow-brown mixture was stirred at room temperature for 2 h. Then, nitromethane (D3) was removed under high vacuum. The residue was suspended in dichloromethane, filtered over syringe filter and concentrated to give 1-(nitromethyl)-2-(p-tolyl)-1,2,3,4-tetrahydro-isoquinoline (19.26 mg, 95 %) as a yellow oil.

^{1}H NMR (400 MHz, $CDCl_3$, 298 K): δ = 7.29 – 7.11 (m, 4H), 7.09 – 7.02 (d, 2H), 6.88 (d, *J* = 8.6 Hz, 2H), 5.48 (s, 1H), 3.70 – 3.53 (m, 2H), 3.09 – 3.00 (m, 1H), 2.75 (dt, *J* = 16.4, 4.6 Hz, 1H), 2.26 (s, 3H) ppm. ^{13}C NMR (100.64 MHz, $CDCl_3$, 298 K): δ = 146.6, 135.5, 133.2, 130.1, 129.4, 129.3, 128.2, 127.1, 126.8, 116.1, 58.5, 42.6, 26.5, 20.5 ppm.

The analytical data matched those in the literature.[7]

Separation of (GFA1+2H)(PF_6)$_2$: The insoluble components consisted of (GFA1+2H)(PF_6)$_2$ in high purity (for entry **K1**, 10.824 mg, 69%; for entry **L1**, 40.04 mg, 85 %) as an off-white solid.

^{1}H NMR spectrum (400 MHz, CD_3NO_2, 298 K) for entry **K1** first measurement (5 min, r.t.)

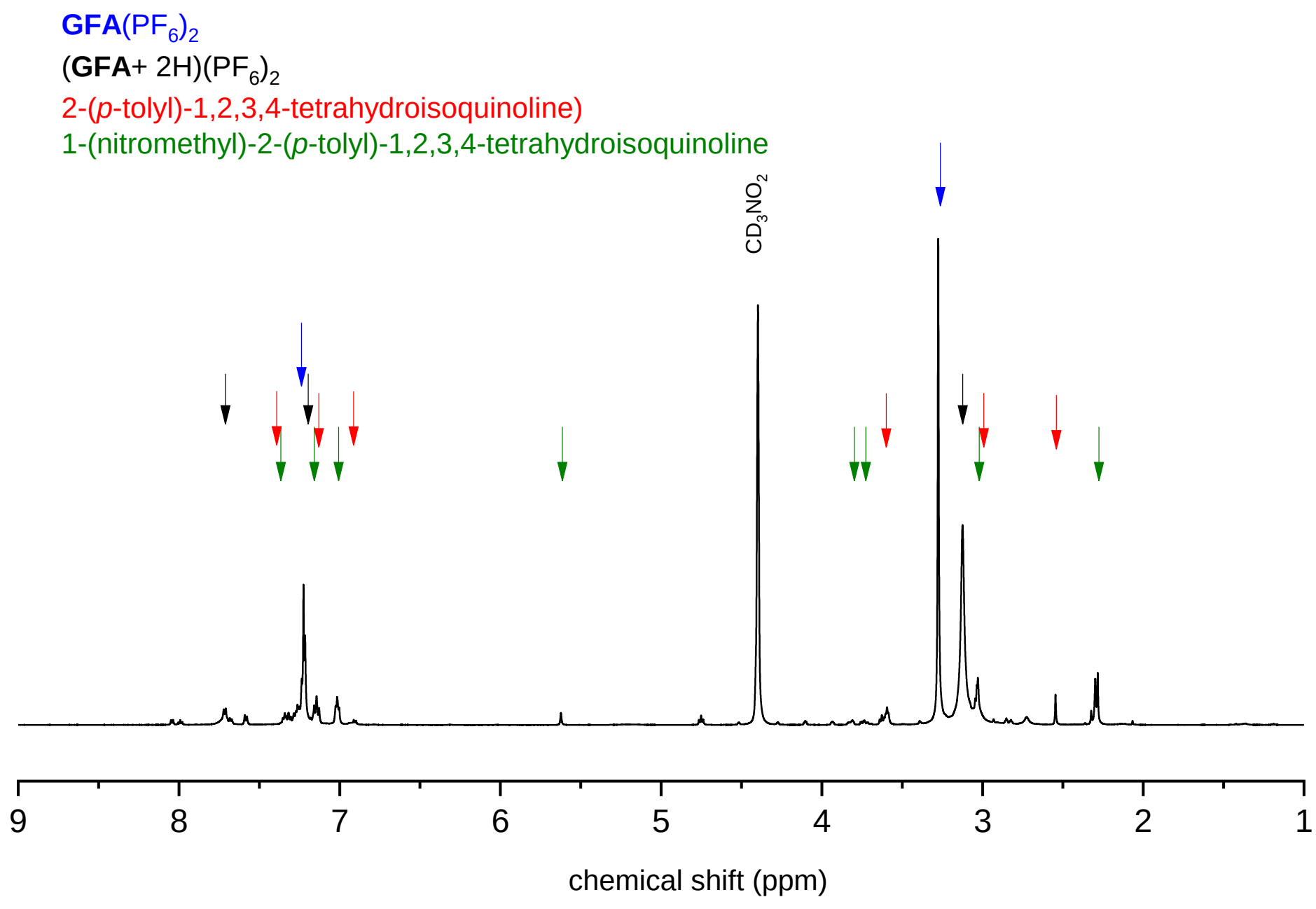

^{1}H NMR spectrum (400 MHz, CD_3NO_2, 298 K) for entry **K1** last measurement (1.5 h, r.t.)

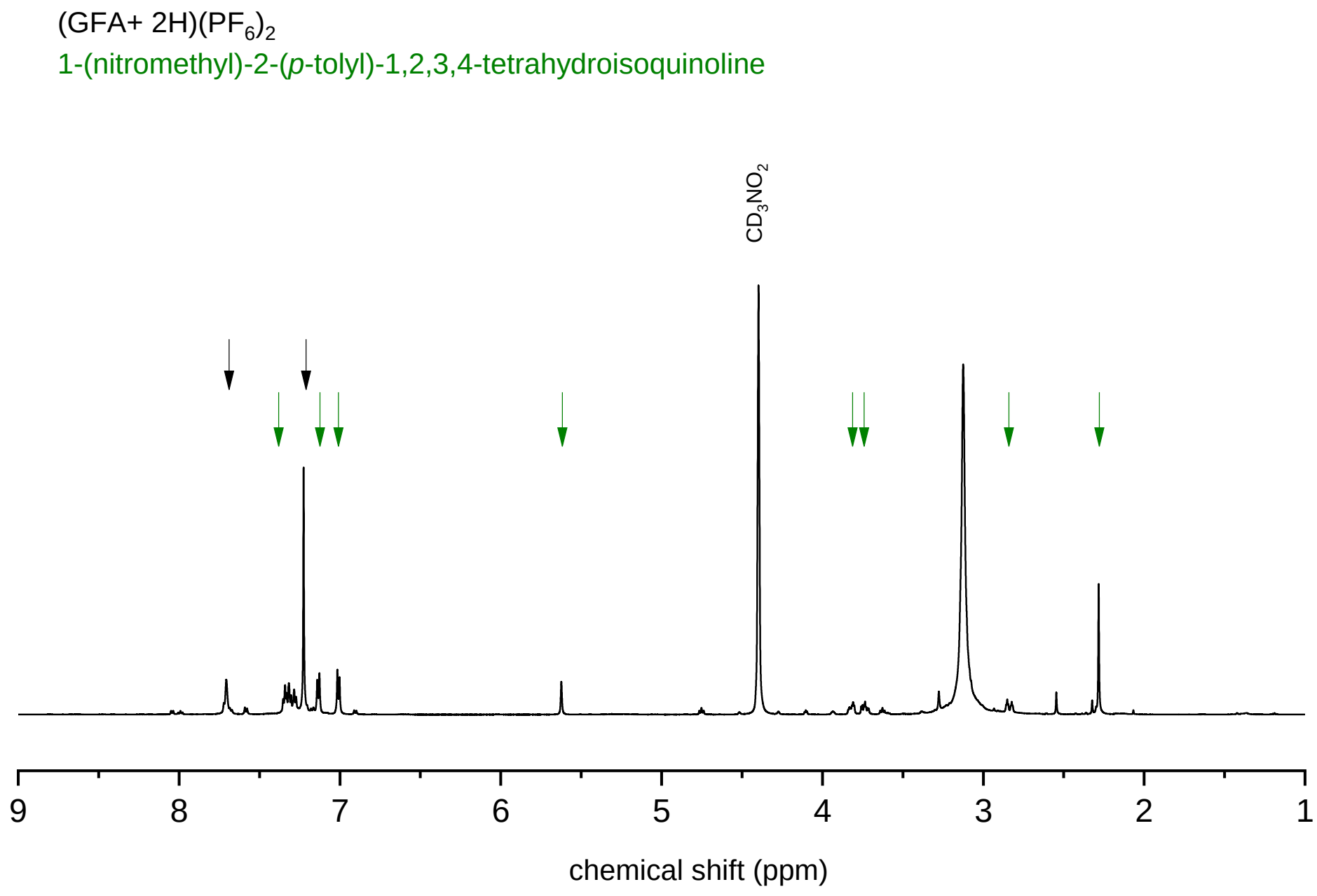

^{1}H NMR spectrum (400 MHz, $CDCl_3$, 298 K) for entry **L1** after workup

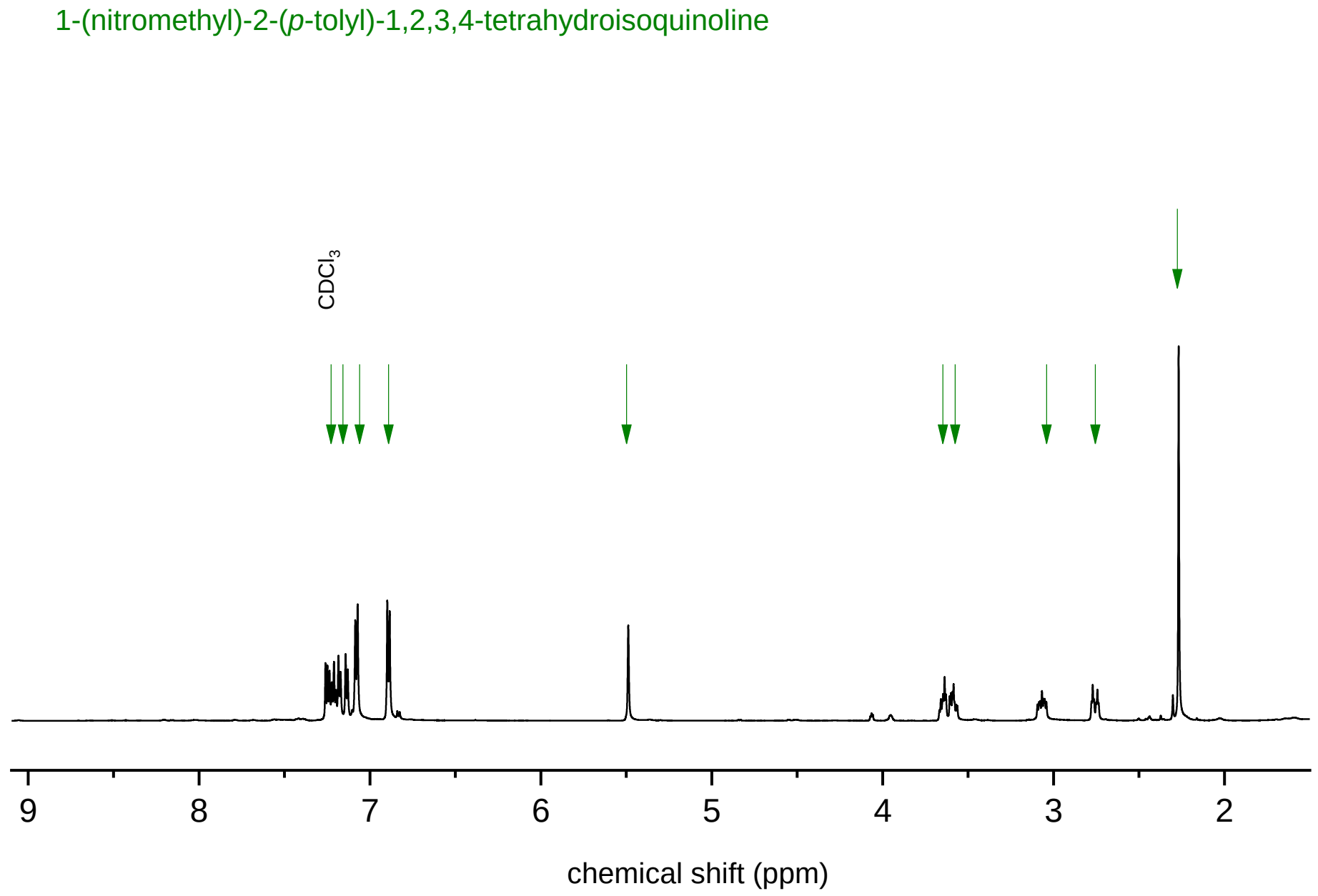

^{13}C NMR spectrum (100.64 MHz, $CDCl_3$, 298 K) for entry **L1** after workup

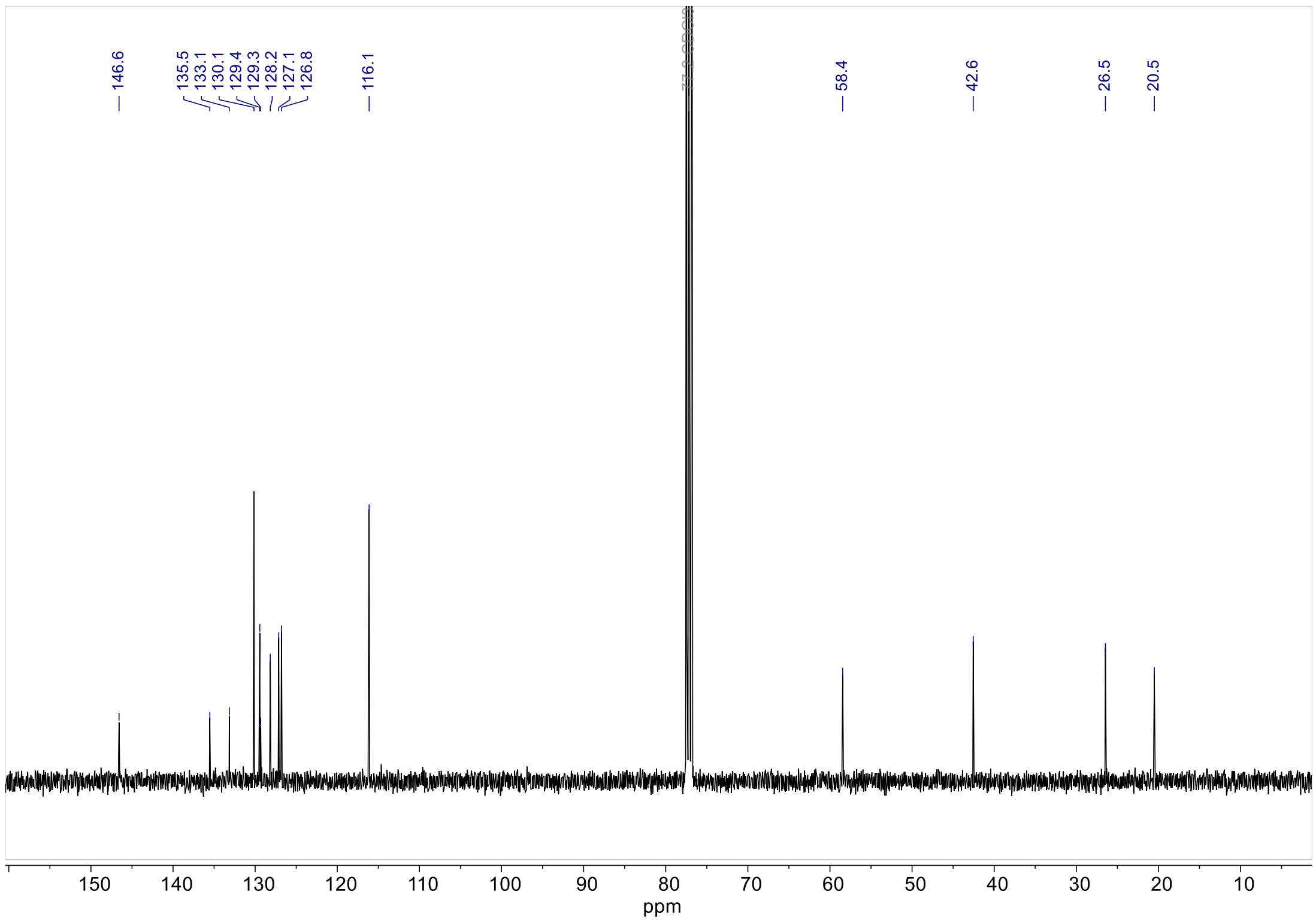

Reaction between 1-(4-(3,4-dihydroisoquinoline-2(1H)-yl)phenyl)ethan-1-one (PTQ-COMe) and nitromethane to give 1-(4-(1-(nitromethyl)-3,4-dihydroisoquinoline-2(1H)-yl)phenyl) ethan-1-one

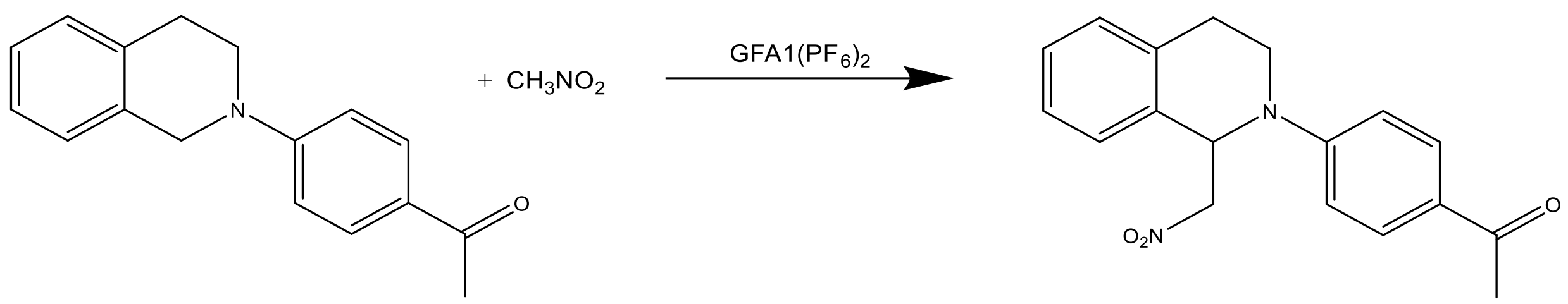

entry	M1a	M1b	N1
GFA1(PF$_6$)$_2$ [mg / µmol]	16.120 / 27	17.830 / 30	48.744 / 82
PTQ-COMe [mg / µmol]	5.618 / 25	5.092 / 20	14.070 / 56
nitromethane [ml]	0.5 (D3)	0.5 (D3)	1.0 (D3)
ratio PTQ-COMe / GFA1^{2+}	1 / 1.1	1 / 1.5	1 / 1.5
condition	NMR tube	NMR tube	Schlenk flask
reaction time (temperature)	20 h (r.t.)	5 h (50 °C)	5 h (50 °C)
Yield after workup [%]	66[a]	95	98

[a] without workup, ratio product / reactant from NMR spectroscopie

Protocol for entry M1a: In an NMR tube, the two reactants PTQ-COMe and GFA1(PF$_6$)$_2$ were dissolved in CD_3NO_2. Conversion was followed by ^{1}H NMR spectroscopy.
Protocol for entry M1b: In an NMR tube, the two reactants PTQ-COMe and GFA1(PF$_6$)$_2$ were dissolved in CD_3NO_2. Conversion was followed by ^{1}H NMR spectroscopy. Then the excess of nitromethane (D3) was removed under high vacuum. The residue was suspended in CH_2Cl_2, filtered over syringe filter and concentrated to give 1-(4-(1-(nitromethyl)-3,4-dihydroisoquinolin-2(1H)-yl)phenyl) ethan-1-one (6.00 mg, 95%) as a yellow oil.
Protocol for N1: To a solution of GFA1(PF$_6$)$_2$ in half the amount of nitromethane, PTQ-COMe and the remaining solvent was added. The yellow-brown mixture was stirred at 50 °C for 5 h. Then, nitromethane (D3) was removed under high vacuum. The residue was suspended in dichloromethane, filtered over syringe filter and concentrated to give 1-(4-(1-(nitromethyl)-3,4-dihydroisoquinolin-2(1H)-yl)phenyl) ethan-1-one (17.11 mg, 98%) as a yellow oil. ^{1}H NMR (600 MHz, $CDCl_3$): δ 7.91 (d, *J* = 8.9 Hz, 2H), 7.33 – 7.21 (m, 3H), 7.19 – 7.06 (m, 1H), 6.97 (d, *J* = 9.0 Hz, 2H), 5.67 (s, 1H), 3.75 – 3.65 (m, 2H), 3.13 (dt, *J* = 16.2, 6.4 Hz, 1H), 2.91 (dt, *J* = 16.2, 5.9 Hz, 1H), 2.52 (s, 3H) ppm. ^{13}C NMR (101 MHz, $CDCl_3$): δ = 196.5, 151.7, 134.9, 132.6, 130.9, 129.2, 128.8, 128.1, 127.2, 112.5, 57.5, 42.1, 27.2, 26.2 ppm.
The analytical data matched those in the literature.[7]

Separation of (GFA1+2H)(PF_6)$_2$: The insoluble components consisted of (GFA1+2H)(PF_6)$_2$ in high purity (for entry **M1b**, 11.75 mg, 63%; for entry **L1**, 39.53 mg, 81 %) as an off-white solid.

^{1}H NMR spectrum (400 MHz, CD_3NO_2, 298 K) for entry **M1b** first measurement (5 min, r.t.)

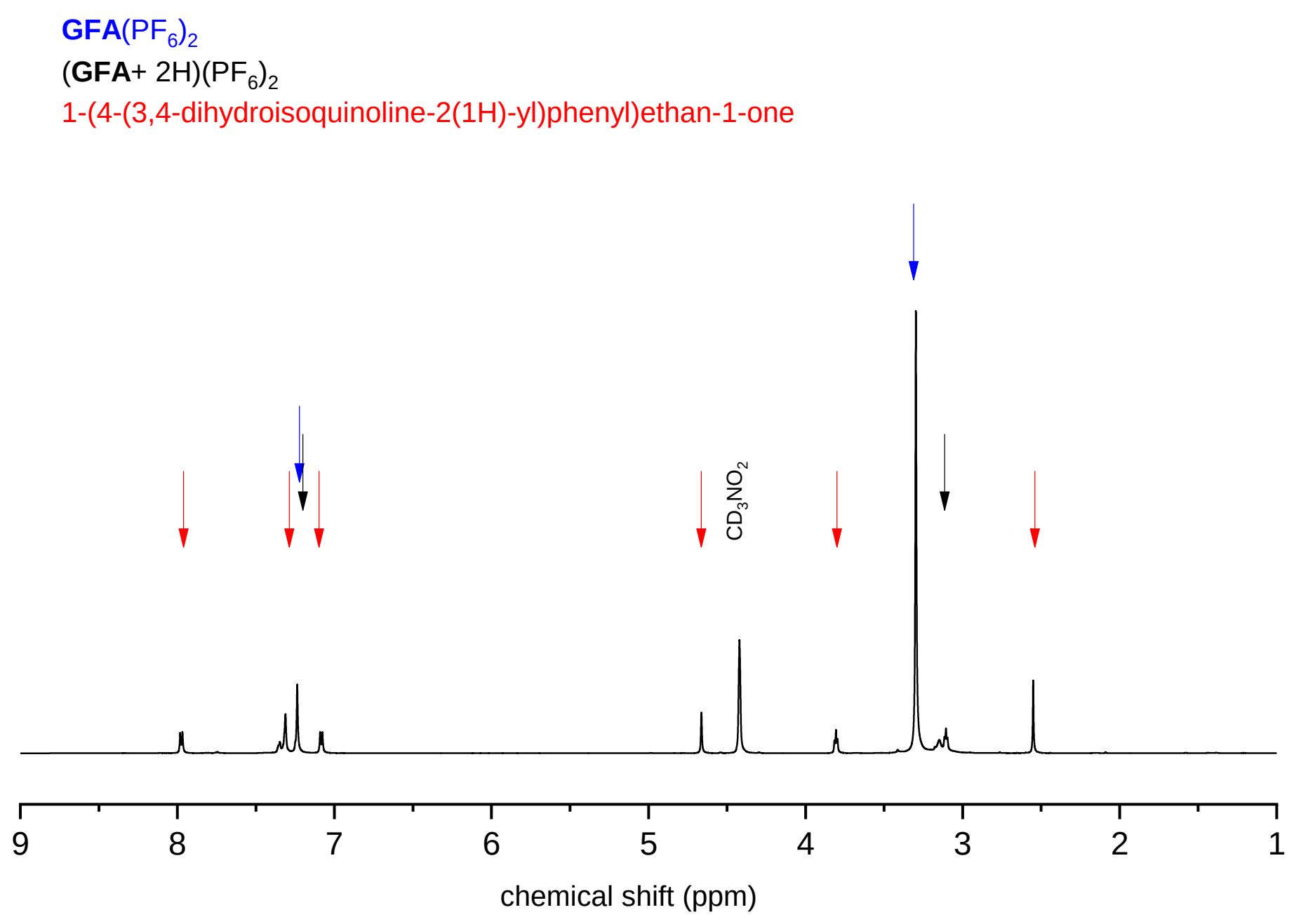

^{1}H NMR spectrum (400 MHz, CD_3NO_2, 298 K) for entry **M1b** last measurement (5 h, 50 °C)

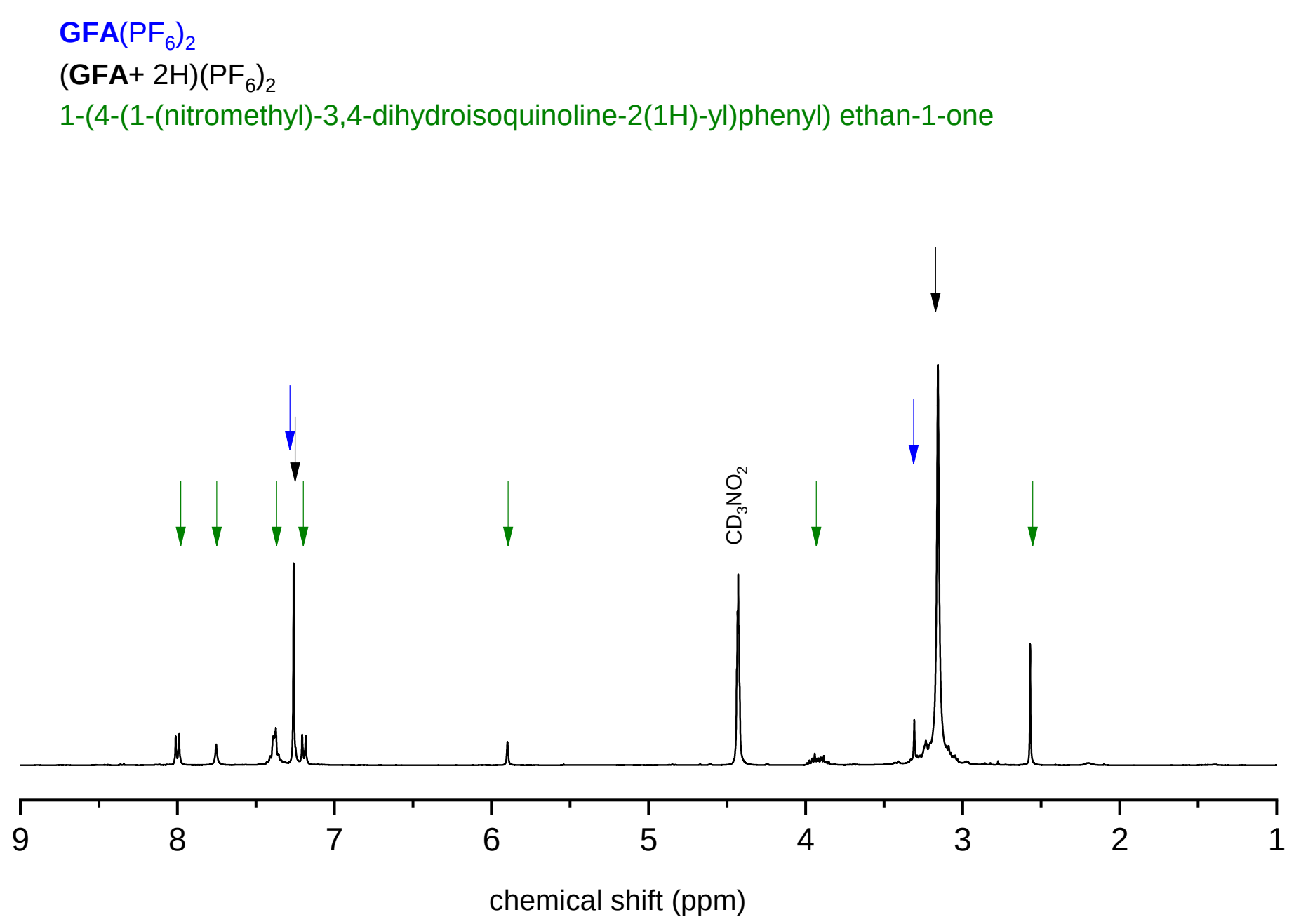

^{1}H NMR spectrum (400 MHz, $CDCl_3$, 298 K) for entry **N1** after workup

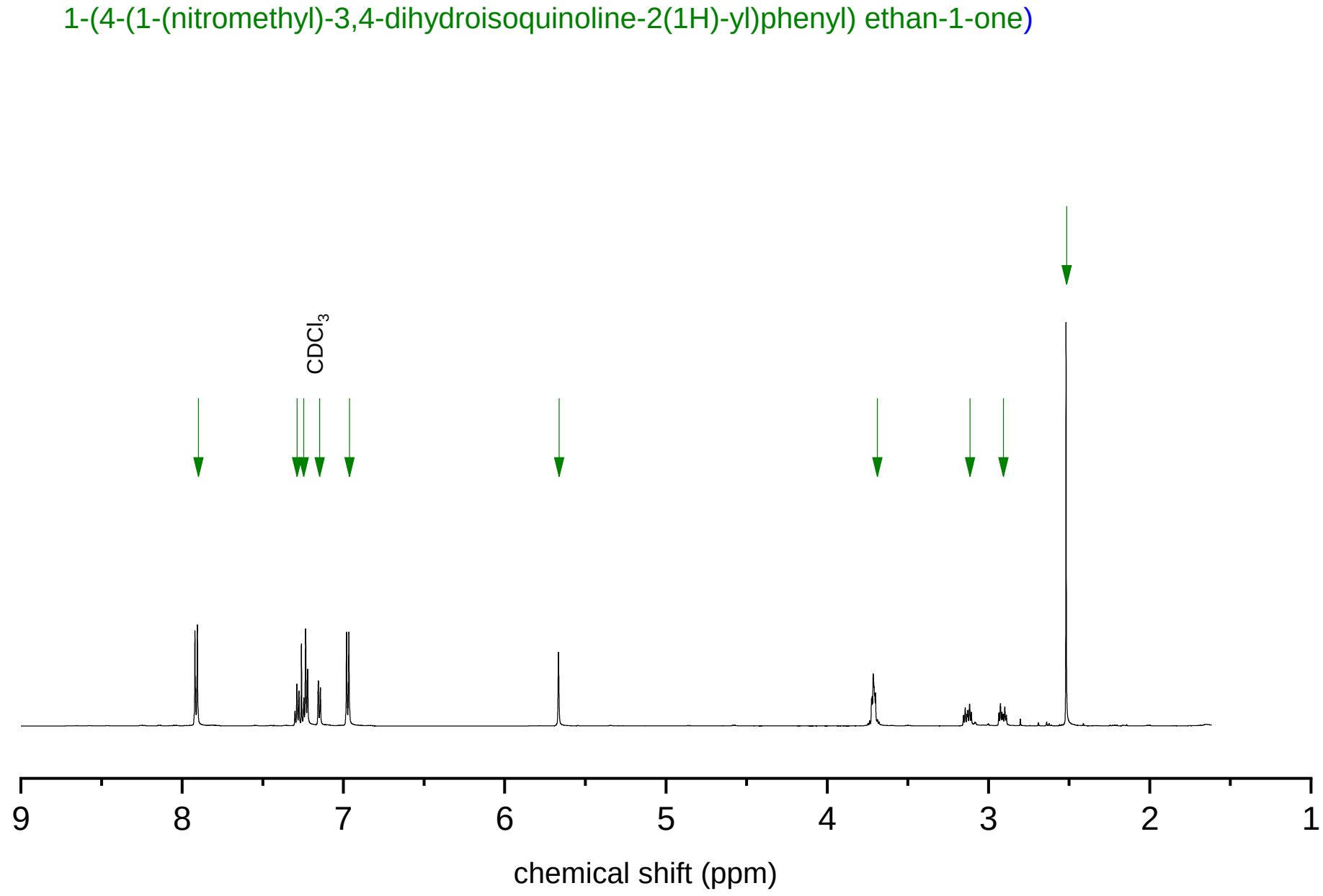

^{13}C NMR spectrum (100.64 MHz, $CDCl_3$, 298 K) for entry **N1** after workup

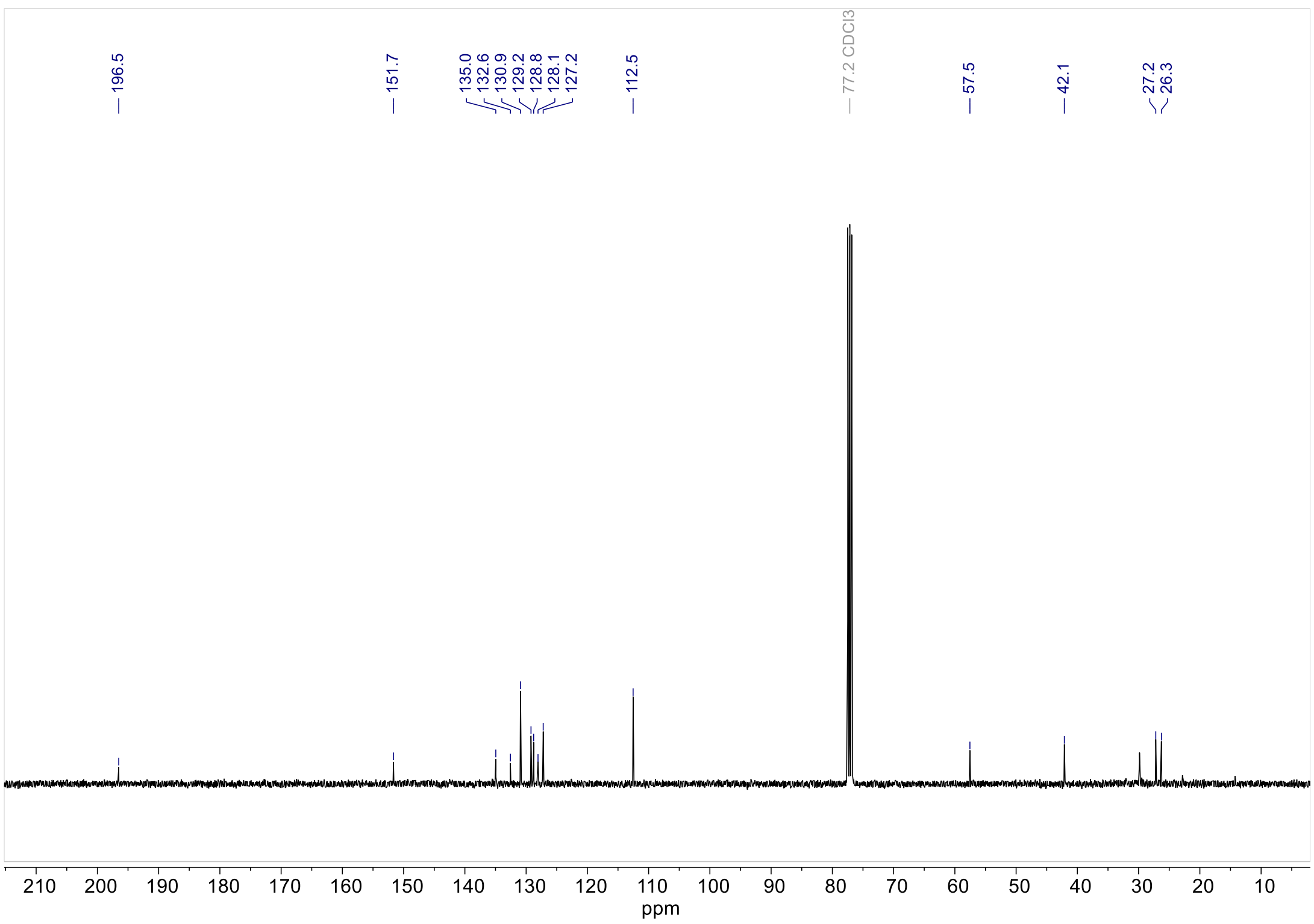

Reactions with nitromethane

Conversion vs. time plot for entry **C1, E1a/b, H1, K1, M1a/b**

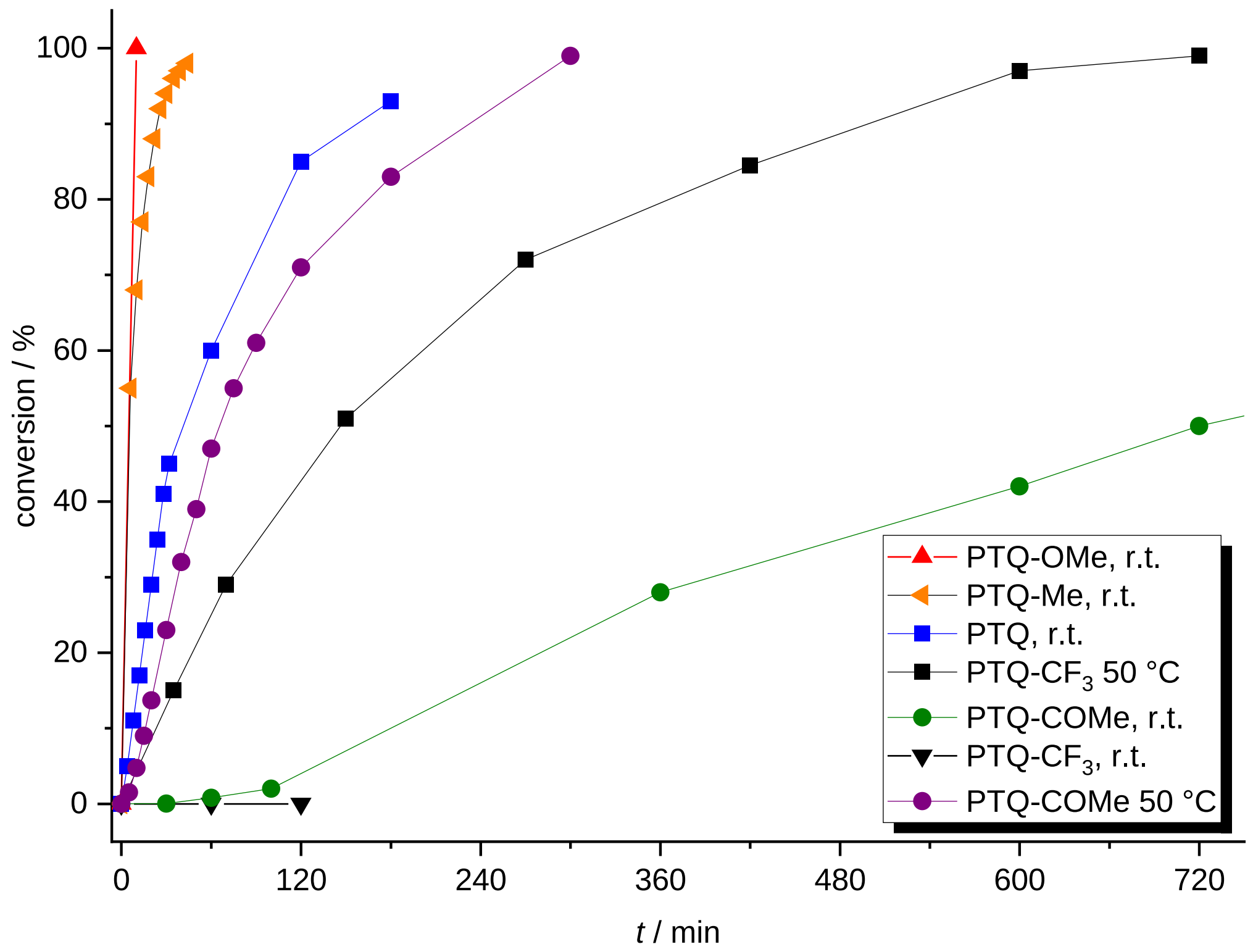

4.2. Reactions with dimethylmalonate

4.2.1 Dimethylmalonate as solvent

Reaction between 2-phenyl-1,2,3,4-tetrahydroisoquinoline (PTQ) and dimethylmalonate to give 2-(2-phenyl-1,2,3,4-tetrahydroisoquinoline-1-yl) malonic acid dimethylester

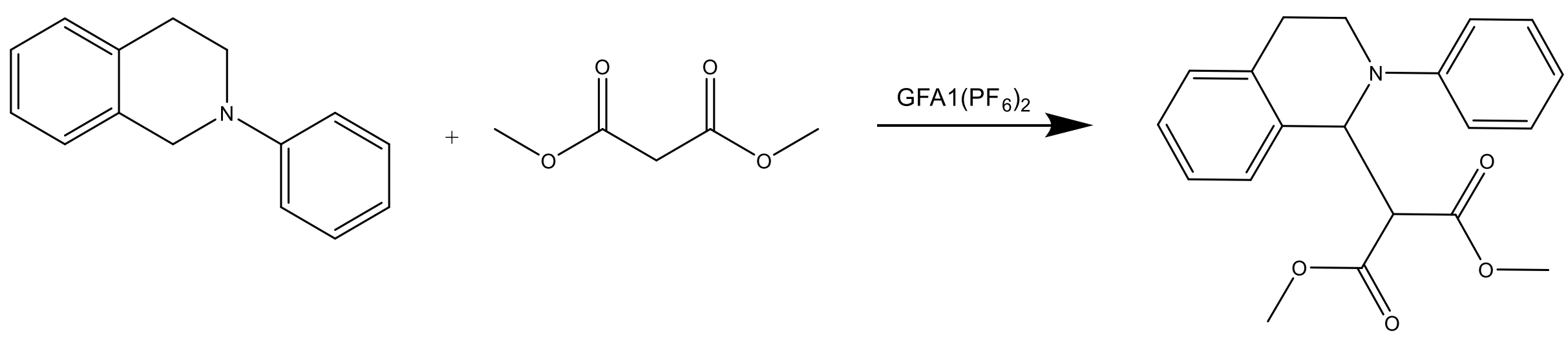

entry	A2
GFA1(PF_6)$_2$ [mg / µmol]	21.940 / 36.9
PTQ [mg / µmol]	5.170 / 24.7
dimethylmalonate [µl / µmol]	150 / 1300
ratio PTQ / GFA1^{2+} / dimethylmalonate	1 / 1.5 / 50
condition	Schlenk flask
reaction time (temperature)	20 h (50 °C)
Yield after workup [%]	81

Protocol for entry A2: The reactants were poorly soluble in dimethylmalonate even at 50 °C. Therefore, the reaction mixture was stirred overnight. The next day, the solvent was removed in vacuum. The residue was suspended in CH_2Cl_2, filtered over syringe filter and concentrated to give 2-(2-phenyl-1,2,3,4-tetrahydroisoquinoline-1-yl)malonic acid dimethylester (7.125 mg, 81%) as a yellow oil in high purity. ^{1}H NMR (600.15 MHz, CD_2Cl_2, 298 K): δ = 7.24 - 7.16 (m, 4 H), 7.17 - 7.08 (m, 2 H), 7.01 - 6.96 (m, 2 H), 6.76 (tt, *J* = 7.3, 1.0 Hz, 1 H), 5.70 (d, *J* = 9.4 Hz, 1 H), 3.95 (d, *J* = 9.4 Hz, 1 H), 3.70 - 3.67 (m, 2 H), 3.66 (s, 3 H), 3.55 (s, 3 H), 3.07 (ddd, *J* = 15.7, 9.1, 6.2 Hz, 1 H), 2.90 - 2.85 (m, 1 H) ppm; ^{13}C NMR (150.92 MHz, CD_2Cl_2, 298 K): δ = 168.4, 167.6, 148.9, 135.8, 134.9, 129.3, 129.1, 127.8, 127.2, 126.2, 118.8, 115.4, 59.3, 58.3, 52.7, 42.3, 26.2 ppm. The analytical data matched those in the literature.[8]

The insoluble components consisted of (GFA1+2H)(PF_6)$_2$ in high purity (16.37 mg, 74%).

^{1}H NMR spectrum (600.15 MHz, $CDCl_3$, 298 K) for entry **A2** after workup

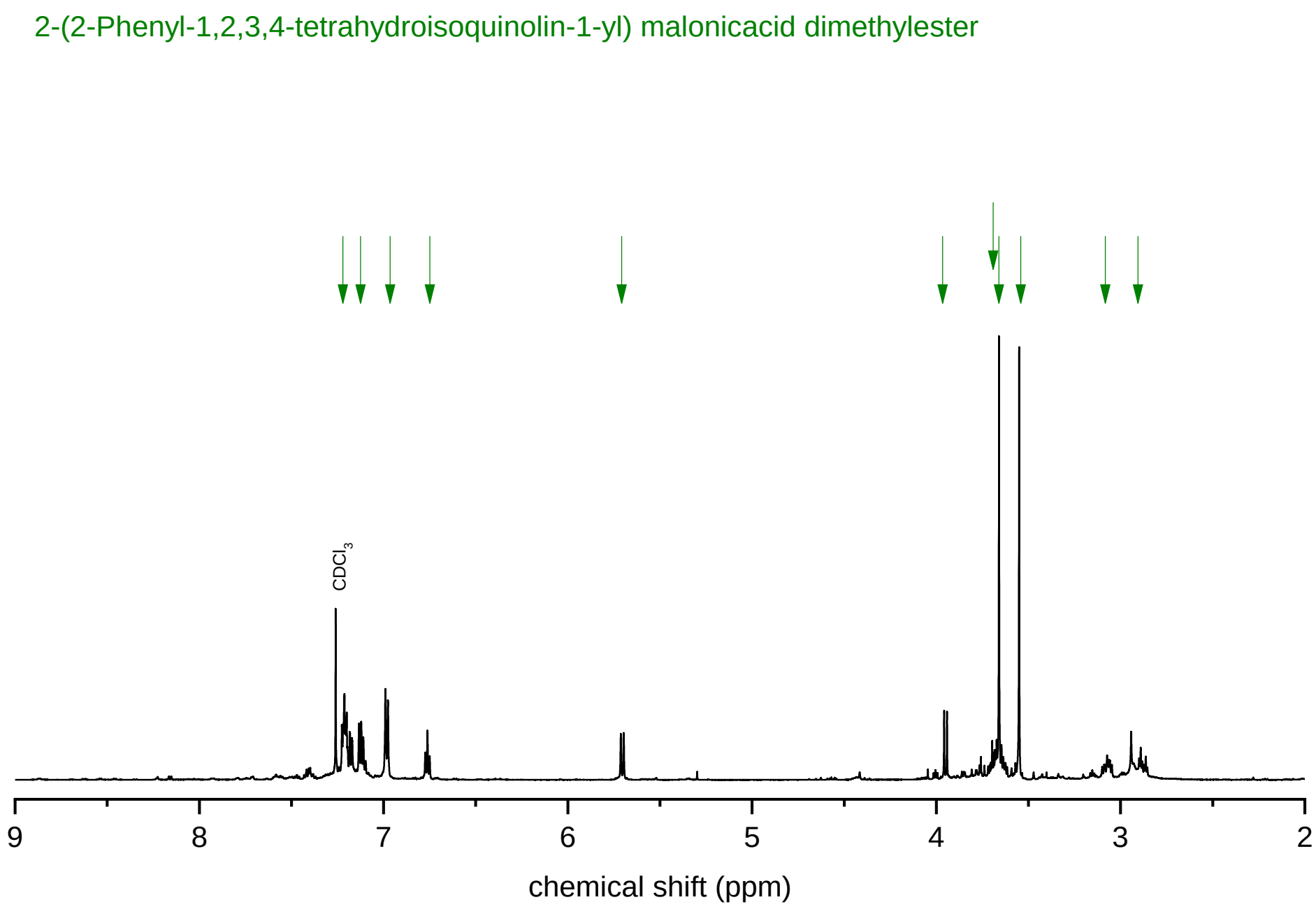

^{13}C NMR spectrum (150.92 MHz, $CDCl_3$, 298 K) for entry **A2** after workup

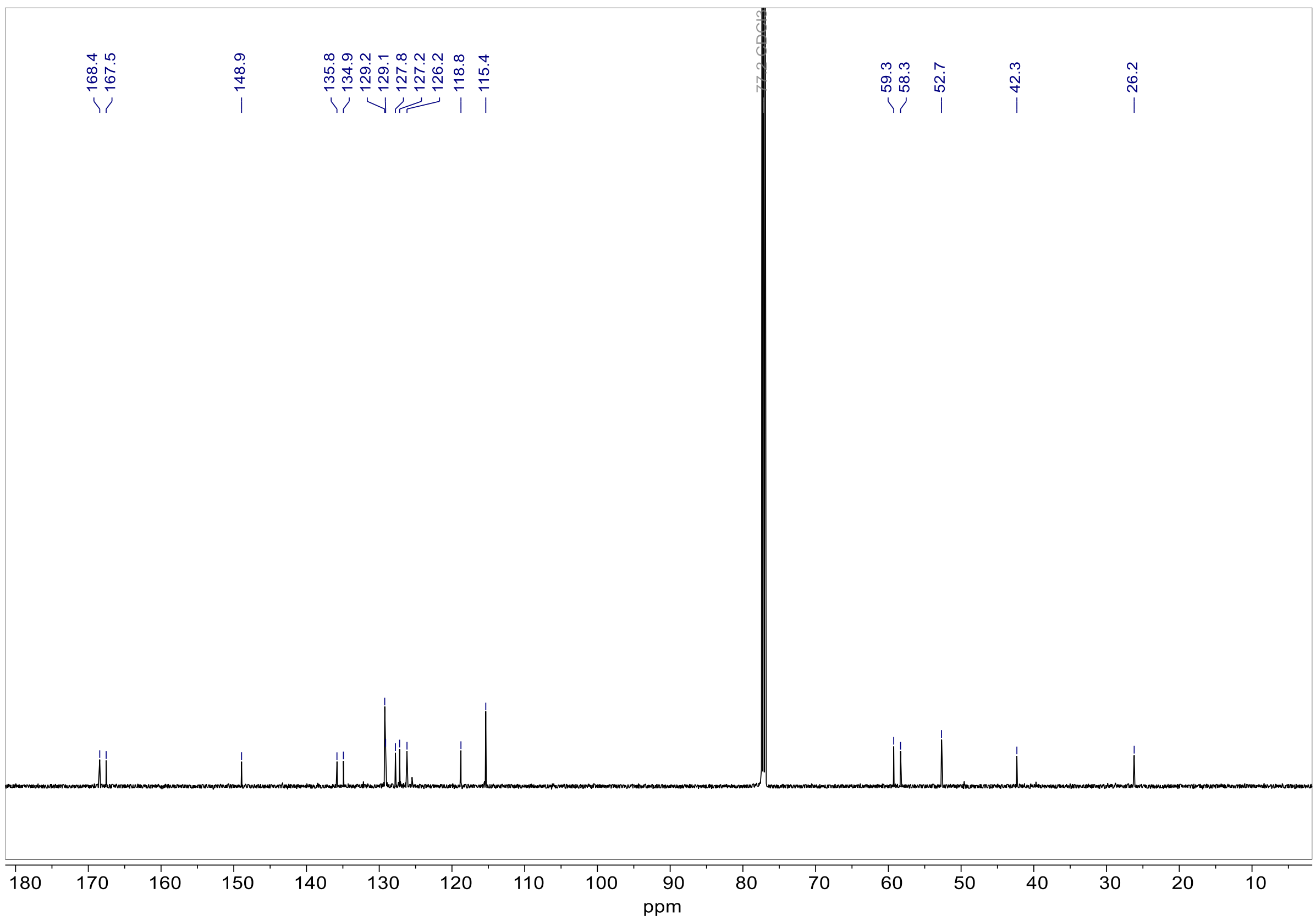

Reaction between 2-[4-(trifluoromethyl)phenyl-1,2,3,4-tetrahydroisoquinoline (PTQ-CF_3) and dimethylmalonate to give 2-(2-(4-trifluoromethyl)-1,2,3,4-tetrahydroisoquinoline-1-yl)-malonic acid dimethylester

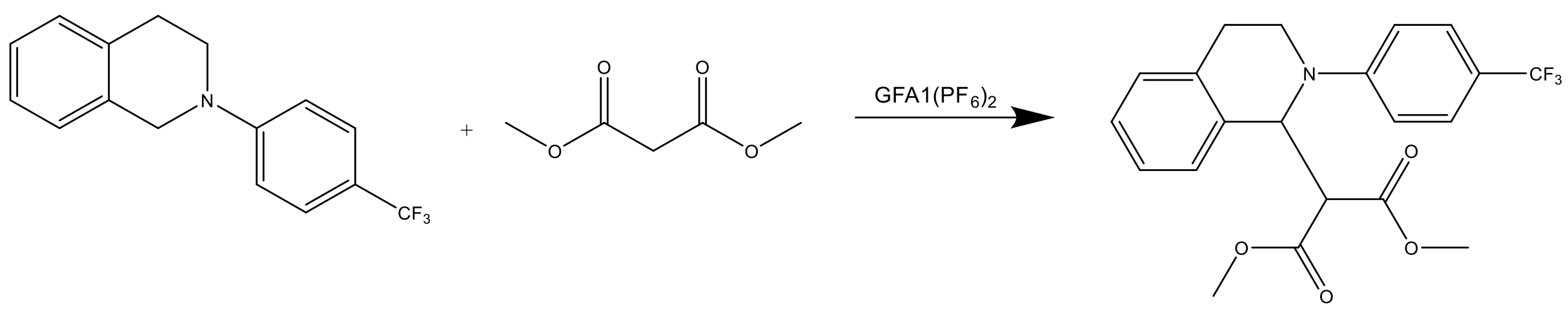

entry	B2	C2
GFA1(PF_6)$_2$ [mg / µmol]	49.9 / 84	21.402 / 36
PTQ-CF_3 [mg / µmol]	17.4 / 63	6.566 / 24
dimethylmalonate [µl / µmol]	500 / 4350	150 / 1300
ratio PTQ-CF_3 / GFA1^{2+} / dimethylmalonate	1 / 1.3 / 55	1 / 1.5 / 50
condition	Schlenk flask	Schlenk flask
reaction time (temperature)	20 h (50 °C)	45 h (50 °C)
Yield after workup [%]	60[a]	88

[a]ratio product / reactant from NMR spectroscopie

General protocol for entry B2 / C2: The reactants were poorly soluble in dimethylmalonate even at 50 °C. Therefore, the reaction mixture was stirred overnight at this temperature. Then, the solvent was removed in vacuum. The residue was suspended in CH_2Cl_2, filtrated over syringe filter and concentrated. **For entry C2:** One obtains 9.5 mg of 2-(2-(4-trifluoromethyl)-1,2,3,4-tetrahydroisoquinoline-1-yl)malonic acid dimethylester (mixture product / reactant = 87 / 13, 21.1 µmol, 88%) as a pale-yellow oil. ^{1}H NMR (600 MHz, $CDCl_3$): δ = 7.45 (d, *J* = 8.7 Hz, 2 H), 7.22 (m, 2 H), 7.20 - 7.12 (m, 2 H), 7.01 (d, *J* = 8.7 Hz, 2 H), 5.81 (d, *J* = 9.4 Hz, 1 H), 3.90 (d, *J* = 9.4 Hz, 1 H), 3.76 - 3.68 (m, 1 H), 3.69 (s, 3 H), 3.61 (m, 1 H), 3.51 (s, 3 H), 3.13 - 2.98 (m, 2 H) ppm; ^{13}C NMR (150.92 MHz, $CDCl_3$, 298 K): δ = 168.2, 167.3, 150.8, 135.7, 134.6, 128.9, 128.2, 127.1, 126.6, 125.9, 124.1, 119.5, 113.3, 59.1, 57.1, 52.8, 42.6, 26.7 ppm. The analytical data matched those in the literature.[9]

Separation of (GFA1+2H)$^{2+}$ for entry B2: The insoluble component consisted of (GFA1+2H)(PF_6)$_2$ and GFA1(PF_6)$_2$ in a ratio of 62:38. **For entry C2:** The insoluble component consisted of (GFA1+2H)(PF_6)$_2$ and GFA1(PF_6)$_2$ in a ratio of 80:20 (32.5 mg, 90%).

^{1}H NMR spectrum (600.15 MHz, $CDCl_3$, 298 K) for entry **B2** after workup

2-[4-(Trifluoromethyl)phenyl]-1,2,3,4-tetrahydroisoquinoline
2-(2-(4-Trifluoromethyl)-1,2,3,4-tetrahydroisoquinoline-1-yl)malonic acid dimethylester

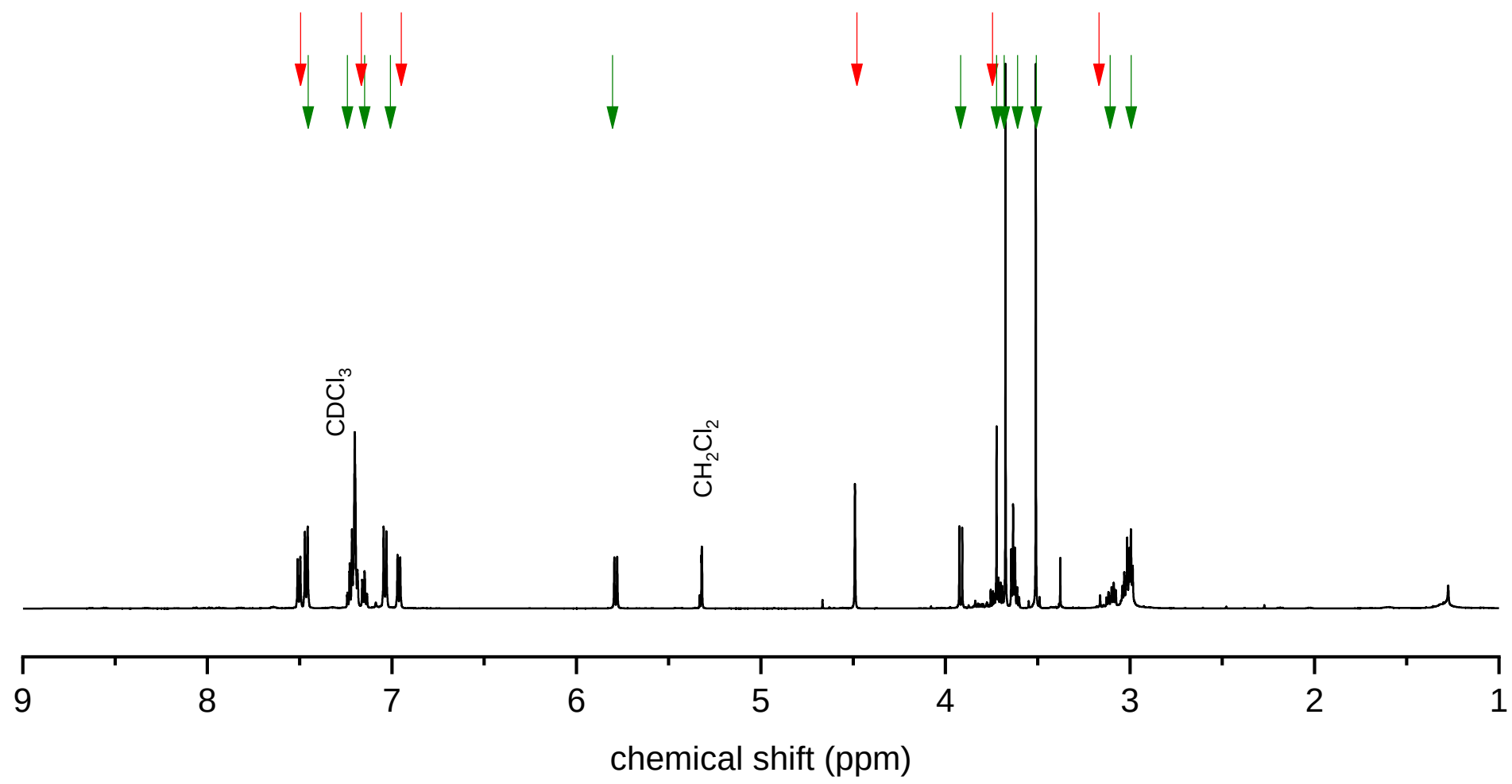

^{1}H NMR spectrum (600.15 MHz, $CDCl_3$, 298 K) for entry **C2** after workup

2-[4-(Trifluoromethyl)phenyl]-1,2,3,4-tetrahydroisoquinoline
2-(2-(4-Trifluoromethyl)-1,2,3,4-tetrahydroisoquinoline-1-yl)malonic acid dimethylester

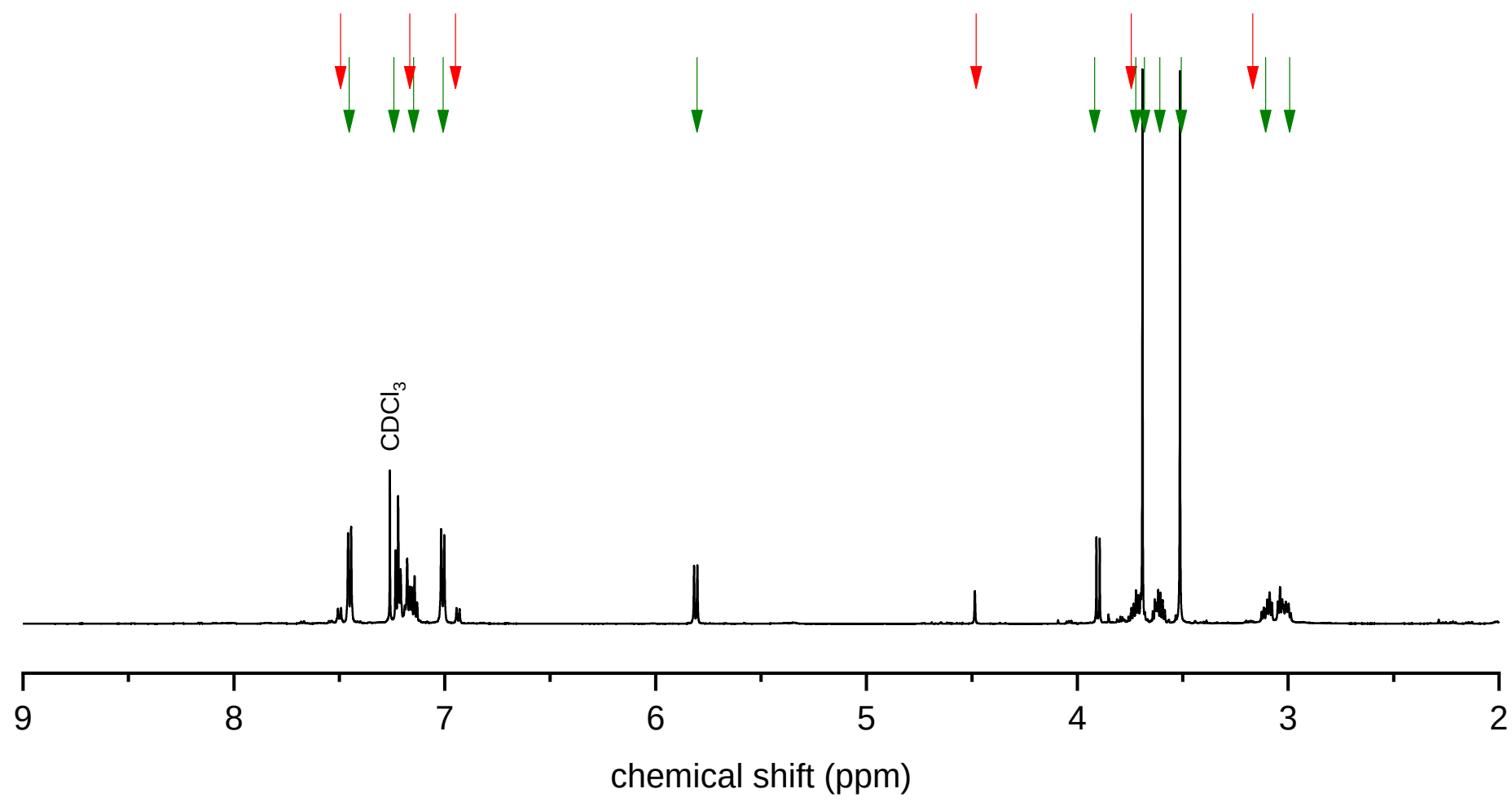

^{13}C NMR spectrum (150.92 MHz, $CDCl_3$, 298 K) for entry **C2** after workup

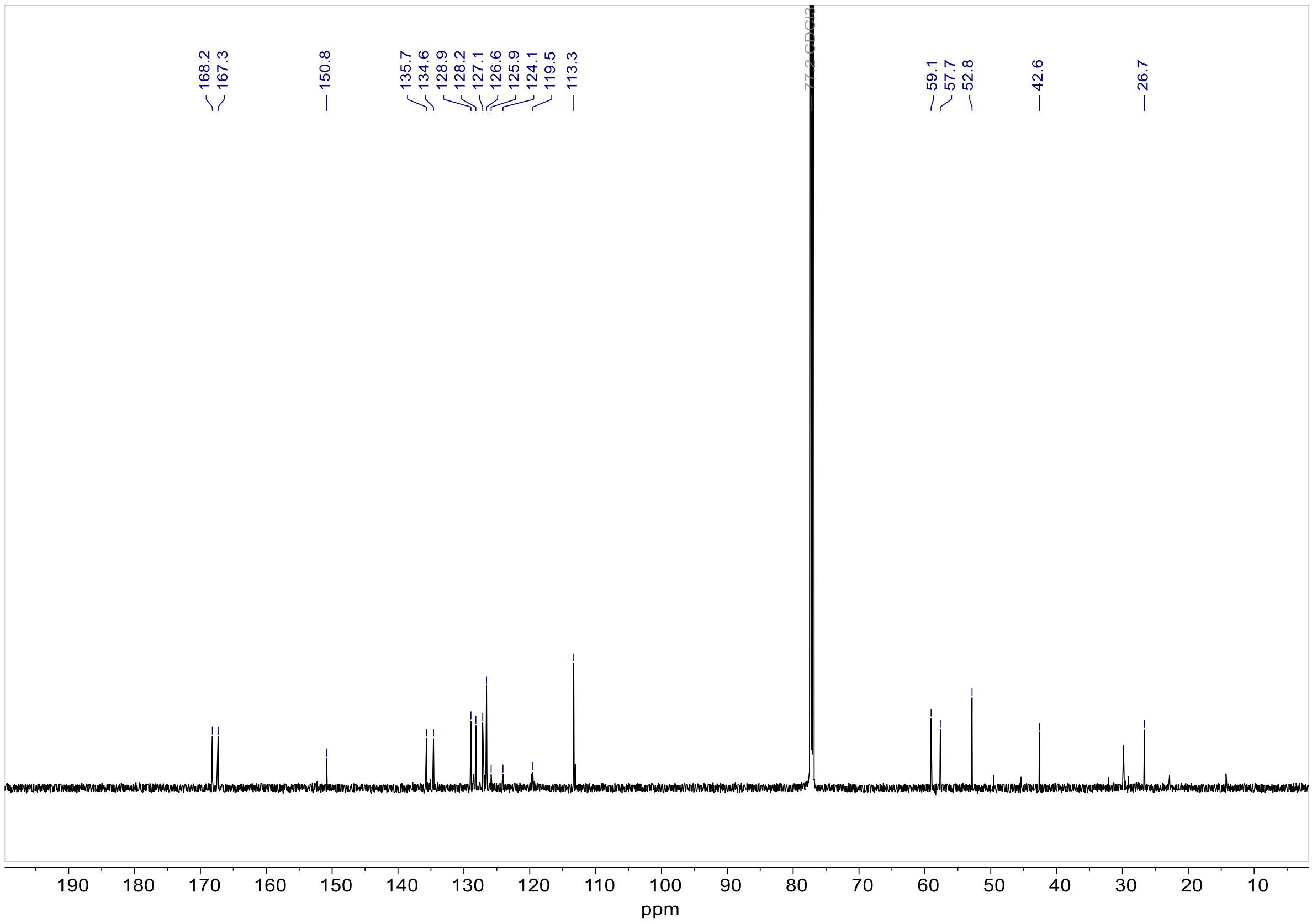

^{1}H NMR spectra for entry **C2** after workup

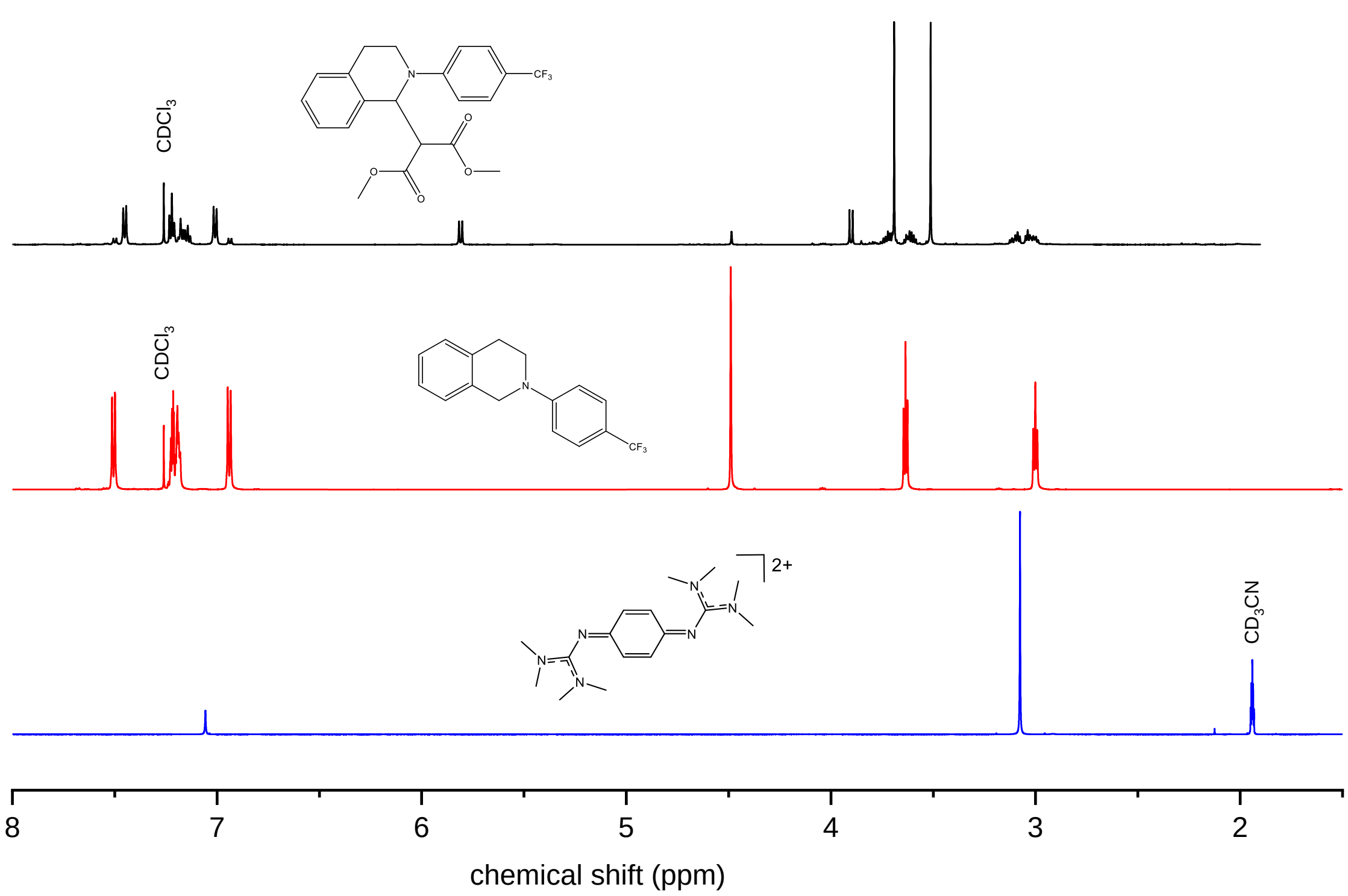

^1H NMR spectrum (600.15 MHz, CD_3CN, 298 K) for entry **C2,** separation of $(GFA1+2H)(PF_6)_2$ and $GFA(PF_6)_2$ in a ratio of 80:20

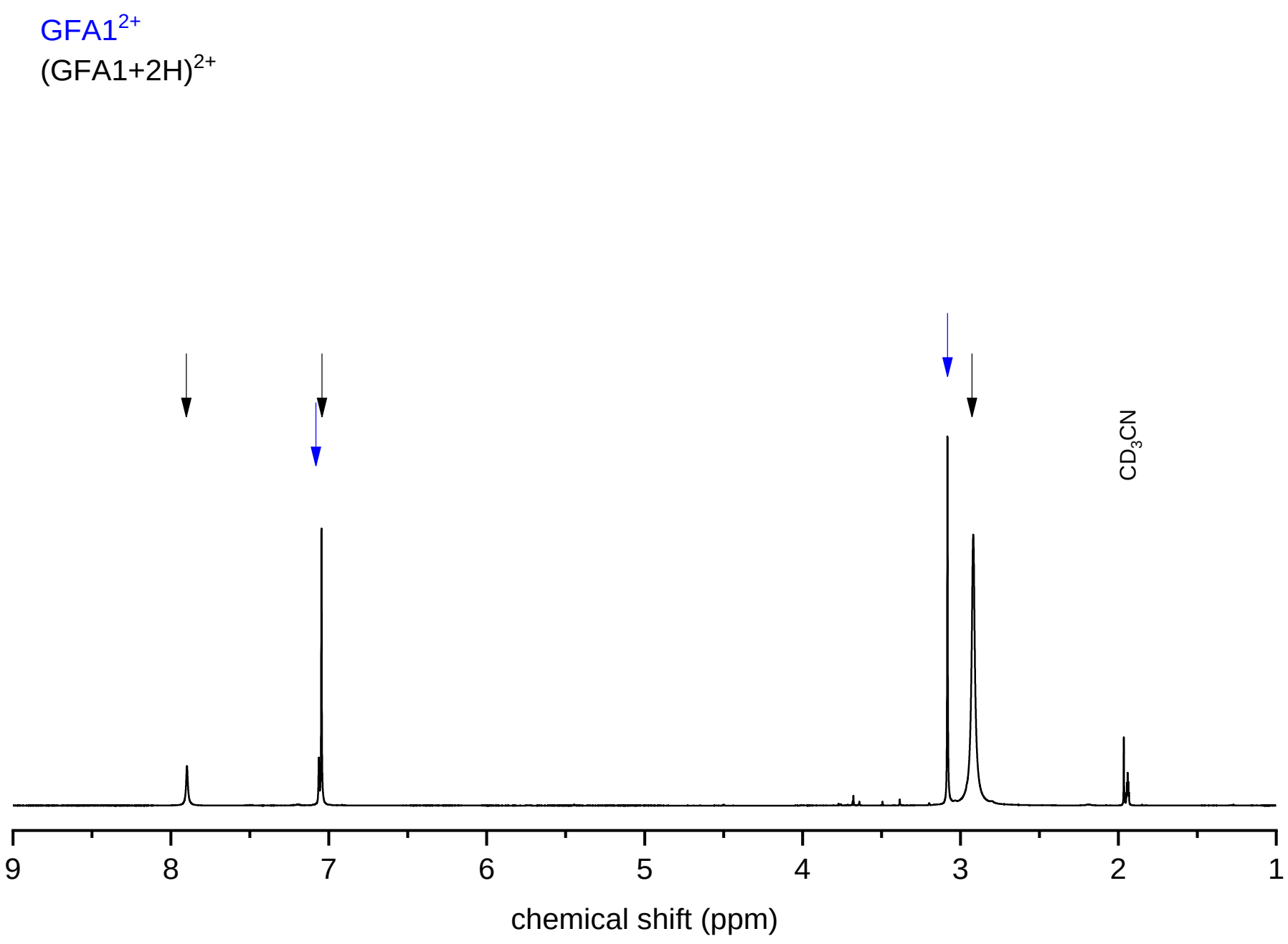

Reaction between 2-(4-methoxyphenyl)-1,2,3,4-tetrahydroisoquinoline (PTQ-OMe) and dimethylmalonate to give 2-(2-(4-methoxyphenyl)-1,2,3,4-tetrahydroisoquinoline-1-yl)-malonic acid dimethylester

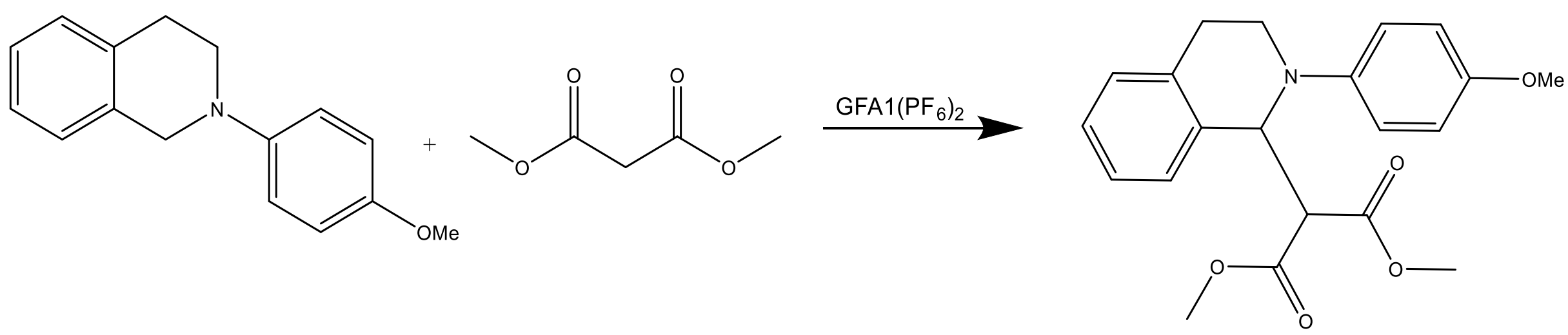

entry	D2
$GFA1(PF_6)_2$ [mg / µmol]	22.868 / 39
PTQ-OMe [mg / µmol]	6.588 / 27.5
dimethylmalonate [µl / µmol]	150 / 1300
ratio PTQ-OMe / $GFA1^{2+}$ / dimethylmalonate	1 / 1.4 / 50
condition	Schlenk flask
reaction time (temperature)	20 h (50 °C)
Yield after workup [%]	82

Protocol for entry D2: The reactants were poorly dissolved in dimethylmalonate, even at 50 °C. Therefore, The reaction mixture was stirred overnight. The next day, the solvent was removed in vacuum. The residue was suspended in CH_2Cl_2, filtrated over syringe filter and concentrated to give 2-(2-(4-methoxyphenyl)-1,2,3,4-tetrahydroisoquinoline-1-yl)malonic acid dimethylester (8.28 mg, 82%) as a brownish-yellow oil in high purity. ^{1}H NMR (600.15 MHz, $CDCl_3$, 298 K): δ = 7.21 -7.15 (m, 2 H), 7.11 (ddd, *J* = 6.2, 1.7, 0.9 Hz, 2 H), 6.91 (d, *J* = 9.1 Hz, 2 H), 6.77 (d, *J* = 9.1 Hz, 2 H), 5.49 (d, *J* = 9.4 Hz, 1 H), 3.95 (d, *J* = 9.4 Hz, 1 H), 3.73 (s, 3 H), 3.69 - 3.64 (m, 1 H), 3.63 (s, 3 H), 3.61 (s, 3 H), 3.57 - 3.52 (m, 1 H), 3.00 (d, *J* = 10.6 Hz, 1 H), 2.76 (d, *J* = 16.5 Hz, 1 H) ppm; ^{13}C NMR (150.92 MHz, $CDCl_3$, 298 K): δ = 168.5, 167.7, 153.4, 143.6, 135.5, 134.9, 129.3, 127.6, 127.2, 126.1, 118.5, 114.6, 59.3, 55.7, 52.7, 52.6, 43.2, 25.7 ppm. The analytical data matched those in the literature.[8]

The insoluble components consisted of $(GFA1+2H)(PF_6)_2$ in high purity (19.34 mg, 83%).

^{1}H NMR spectrum (600.15 MHz, $CDCl_3$, 298 K) for entry **D2** after workup

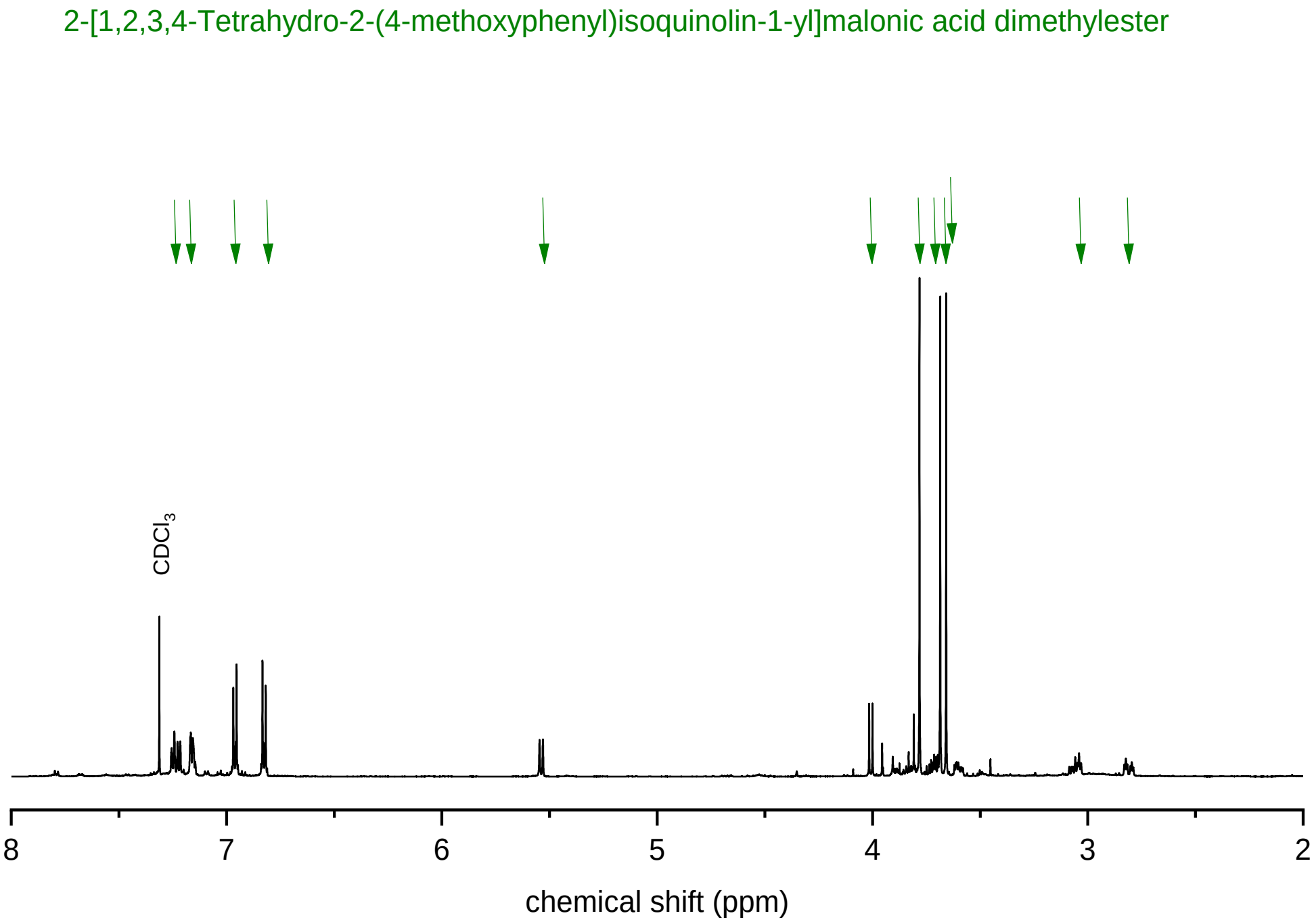

^{13}C NMR spectrum (150.92 MHz, $CDCl_3$, 298 K) for entry **D2** after workup

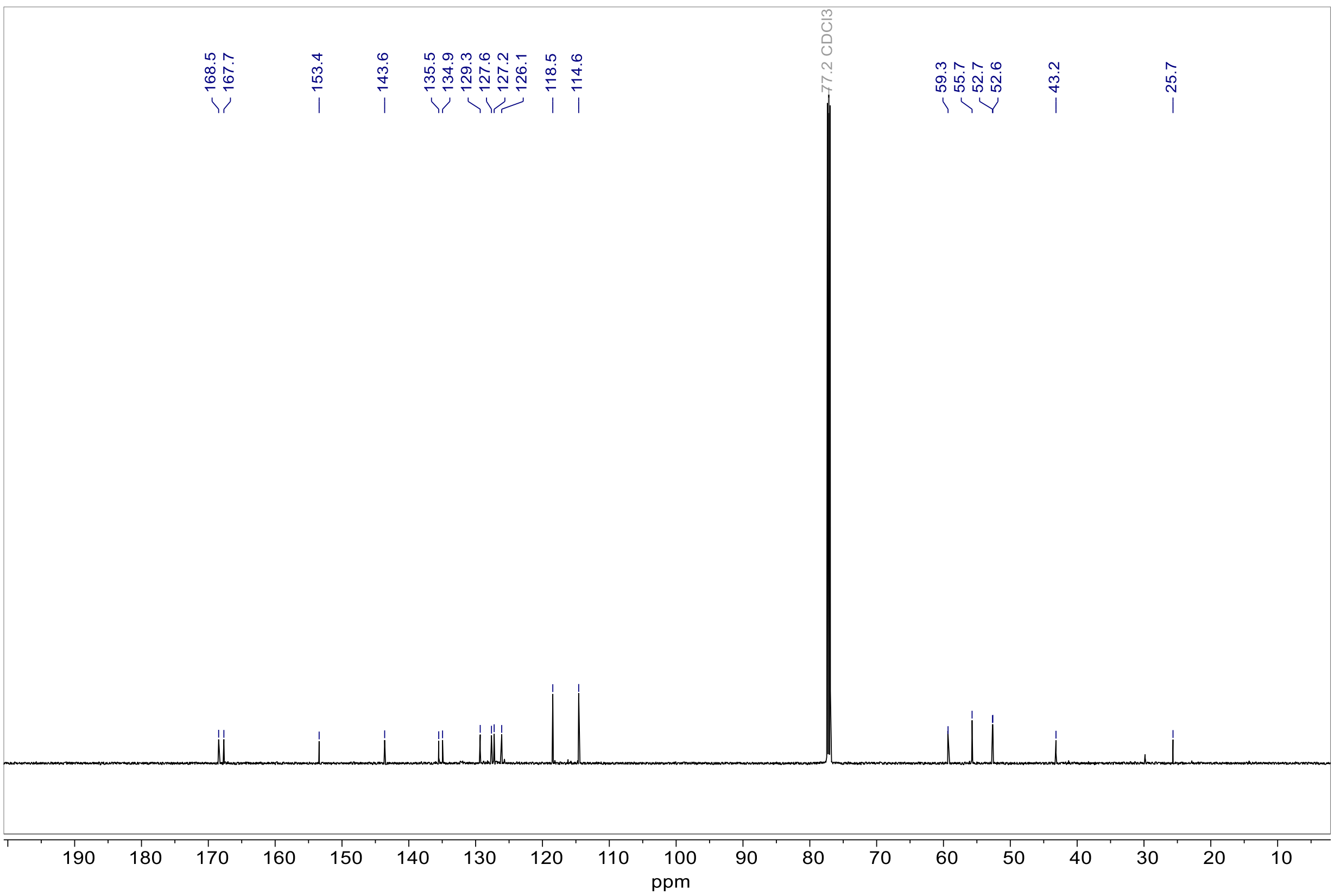

^{1}H NMR spectra for entry **D2** after workup

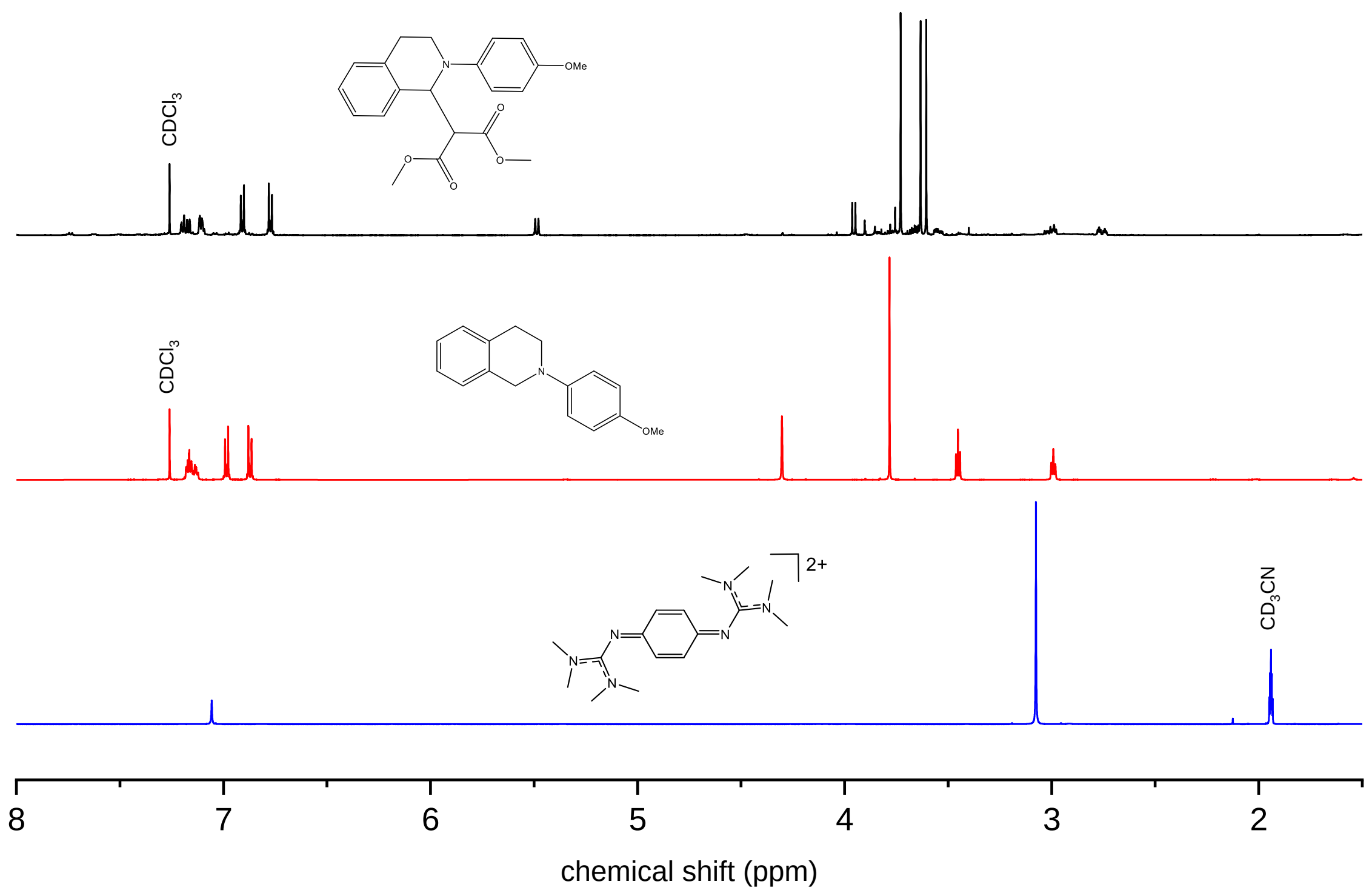

4.2.2 Acetonitrile as solvent

Reaction between 2-phenyl-1,2,3,4-tetrahydroisoquinoline (PTQ) and dimethylmalonate

entry	E2
PTQ [mg / µmol]	4.346 / 21
dimethylmalonate [µl / µmol]	4.8 / 42
CD_3CN [ml]	0.42
solution I [ml]	0.21
GFA1$(PF_6)_2$ [mg / µmol]	25 / 42
ratio PTQ / GFA1^{2+} / dimethylmalonate	1 / 2 / 2
condition	NMR tube
reaction time (temperature)	9 h (r.t.)
Yield after workup [%]	55

Protocol for entry E2: First a solution of 25.0 mg (42 µmol) GFA1$(PF_6)_2$ in 0.21 ml CD_3CN ($c = 2.0\cdot10^{-1}$ mol·l^{-1}) was prepared (solution I). In an NMR tube, the two reactants PTQ and dimethylmalonate were dissolved in CD_3CN. After addition of solution I, conversion was followed by ^{1}H NMR spectroscopy. Then, the solvent and excess dimethylmalonate were removed under high vacuum. The residue was suspended in CH_2Cl_2, filtrated over syringe filter and concentrated to give 4.2 mg of 2-(2-phenyl-1,2,3,4-tetrahydroisoquinoline-1-yl)malonic acid dimethylester (mixture product / reactant = 91 / 9, 11.7 µmol, 55%) as a yellow oil.
^{1}H NMR (600.15 MHz, $CDCl_3$) δ= 7.20 (dd, *J* = 16.5, 9.3 Hz, 4 H), 7.15 - 7.09 (m, 2 H), 6.98 (d, *J* = 8.04 Hz, 2 H), 6.76 (t, *J* = 7.26, 7.26 Hz, 1 H), 5.70 (d, *J* = 9.4 Hz, 1 H), 3.95 (d, *J* = 9.4 Hz, 1 H), 3.69 (d, *J* = 5.3 Hz, 2 H), 3.66 (s, 3 H), 3.55 (s, 3 H), 3.06 (m, *J* = 3.0 Hz, 1 H), 2.88 (m, *J* = 6.0 Hz, 1 H). The analytical data matched those in the literature.[8]

The insoluble components consisted of (GFA1+2H)$(PF_6)_2$ in high purity (18.3 mg, 73%).

^1H NMR spectrum (400.20 MHz, CD_3CN, 298 K) for entry **E2** first measurement (10 min, r.t.)

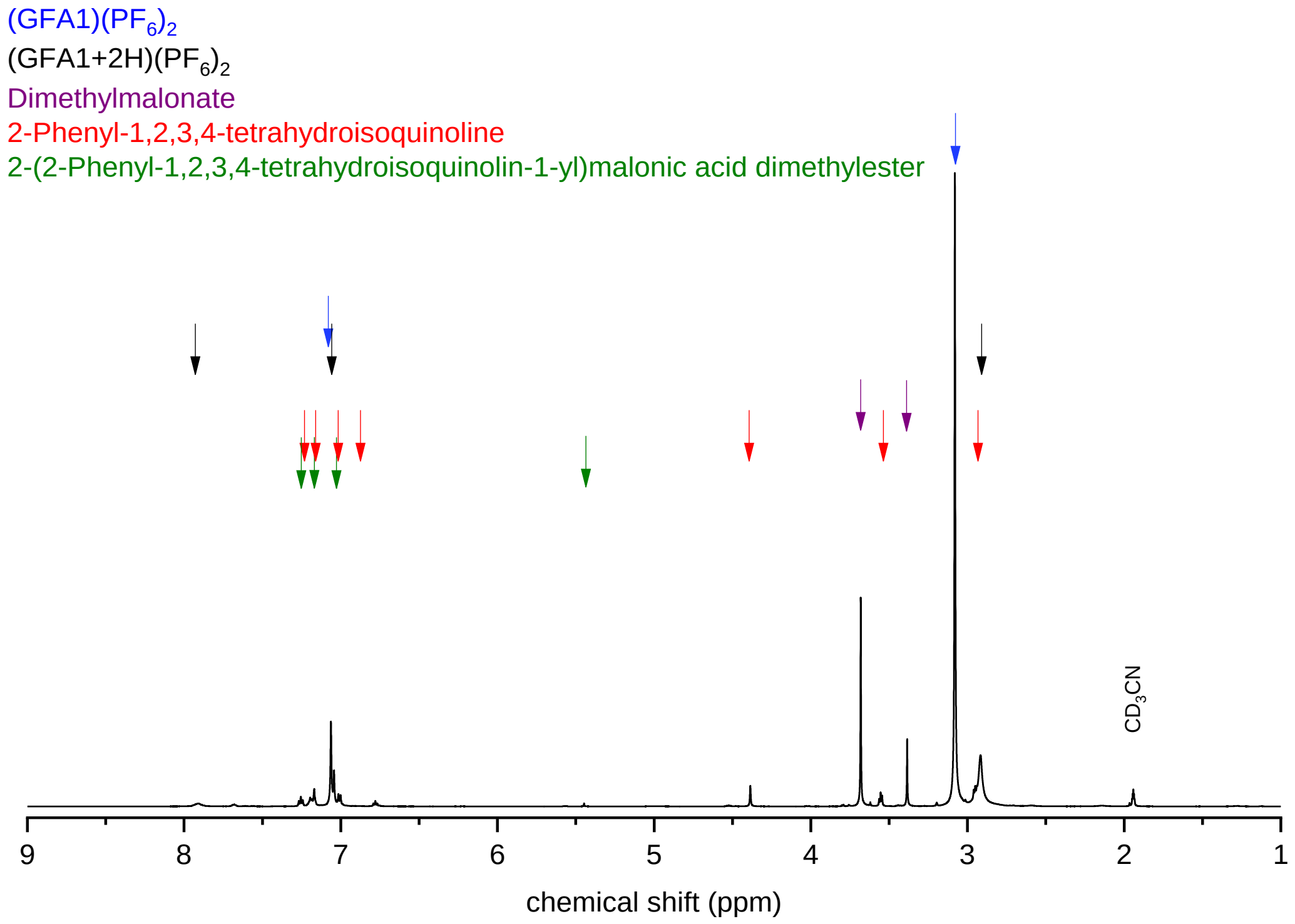

^1H NMR spectrum (400.20 MHz, CD_3CN, 298 K) for entry **E2** last measurement (9 h, r.t.)

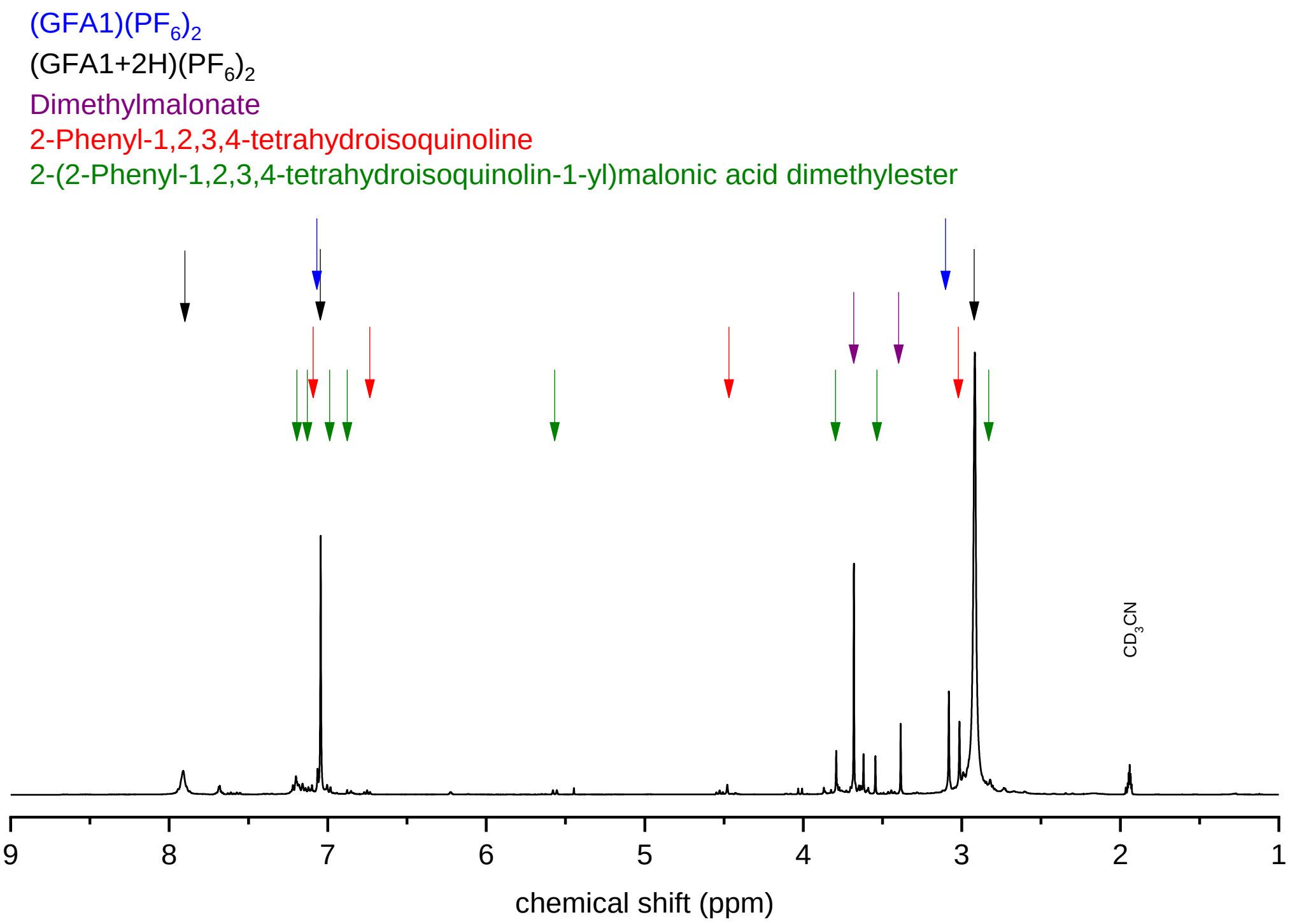

^{1}H NMR spectrum (600.15 MHz, $CDCl_3$, 298 K) for entry **E2** after workup

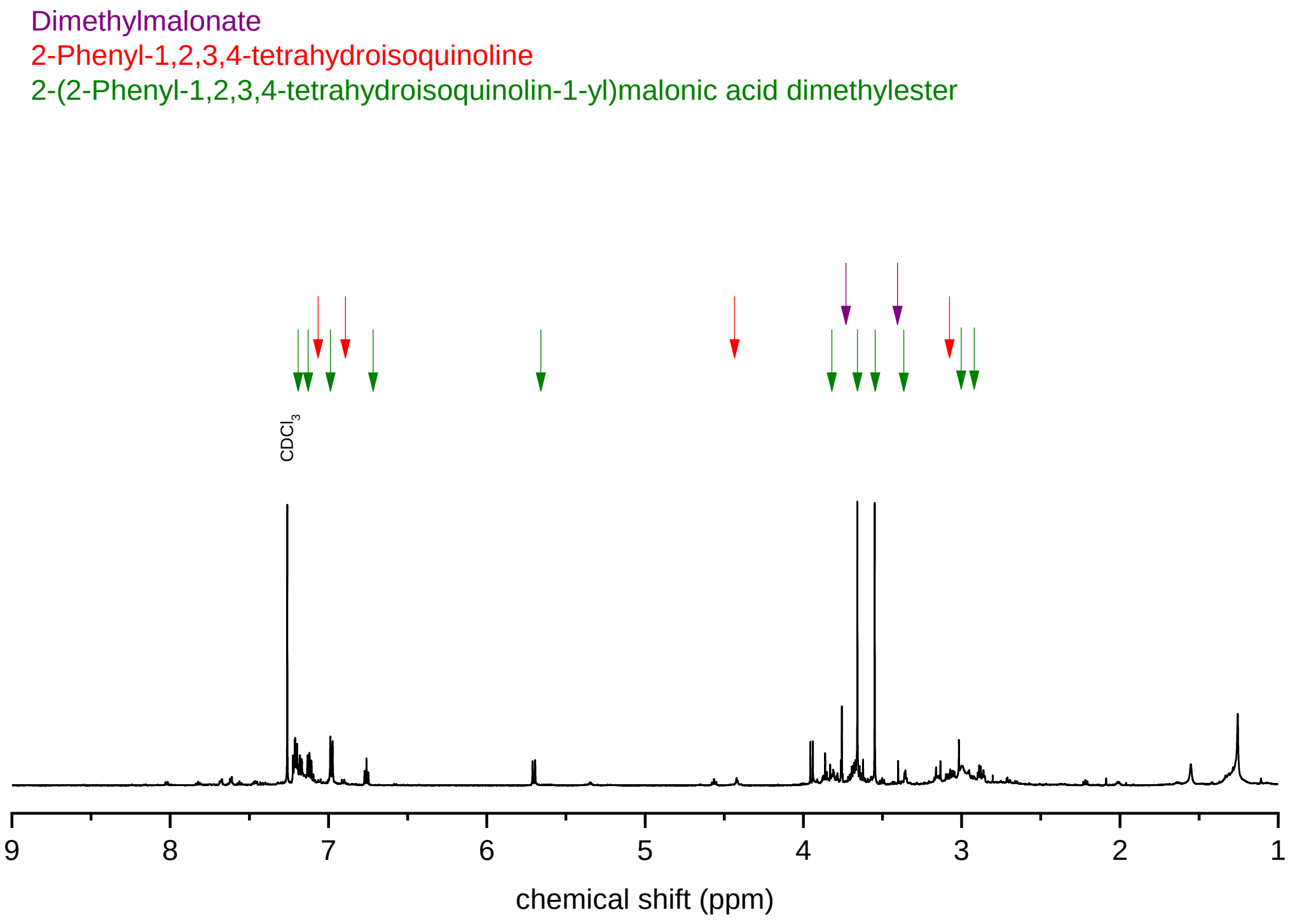

Reaction between 2-(4-trifluoromethyl)phenyl]-1,2,3,4-tetrahydroisoquinoline (PTQ-CF_3) and dimethylmalonate

entry	F2	G2
PTQ-CF_3 [mg / µmol]	6.994 / 25	6.994 / 25
dimethylmalonate [µl / µmol]	45.7 / 50	45.7 / 50
CD_3CN [ml]	0.5	0.5
solution I [ml]	0.25	0.25
GFA1(PF_6)$_2$ [mg / µmol]	29.7 / 50	29.7 / 50
ratio PTQ- CF_3 / GFA1^{2+} / dimethylmalonate	1 / 2 / 2	1 / 2 / 2
condition	NMR tube	NMR tube
reaction time (temperature)	20 h (r.t.)	20 h (50 °C)
Yield after workup [%]	17[a]	67

[a] Ratio product / reactant determined from NMR spectroscopy.

General protocol for entry F2 / G2: First, a solution of 29.7 mg (50 µmol) GFA1(PF_6)$_2$ in 0.25 ml CD_3CN ($c = 2.0\cdot10^{-1}$ mol·l^{-1}) was prepared (solution I). In an NMR tube, the two reactants PTQ-CF_3 and dimethylmalonate were dissolved in CD_3CN. After addition of solution I, conversion was followed by ^{1}H NMR spectroscopy. Then, the solvent and excess dimethylmalonate were removed under high vacuum. The residue was suspended in CH_2Cl_2, filtered over syringe filter and concentrated.

For entry G2: One obtains 7.62 mg of 2-(2-(4-trifluoromethyl)-1,2,3,4-tetrahydro-isoquinoline-1-yl)malonic acid dimethylester (mixture product / reactant = 85 / 15, 16.7 µmol, 67%) as pale-yellow oil. ^{1}H NMR (600.15 MHz, $CDCl_3$): δ= 7.45 (d, *J* = 8.77 Hz, 2 H), 7.22 (t, *J* = 7.03, 7.03 Hz, 2 H), 7.16 (dd, *J* = 13.52, 7.76 Hz, 2 H), 7.01 (d, *J* = 8.85 Hz, 2 H), 5.81 (d, *J* = 9.42 Hz, 1 H), 3.90 (d, *J* = 9.42 Hz, 1 H), 3,72 (m, 1 H), 3.69 (s, 3 H), 3.61 (td, *J* = 12.63, 6.05, 6.05 Hz, 1 H) 3.51 (s, 3 H), 3.14 - 3.06 (m, 1 H), 3.02 (td, *J* = 13.46, 6.18, 6.18 Hz, 1 H) ppm. The analytical data matched those in the literature.[9]

The insoluble components consisted of (GFA1+2H)(PF_6)$_2$ in high purity (22.4 mg, 75%).

^{1}H NMR spectrum (400.20 MHz, CD_3CN, 298 K) for entry **G2** first measurement (20 min, r.t.)

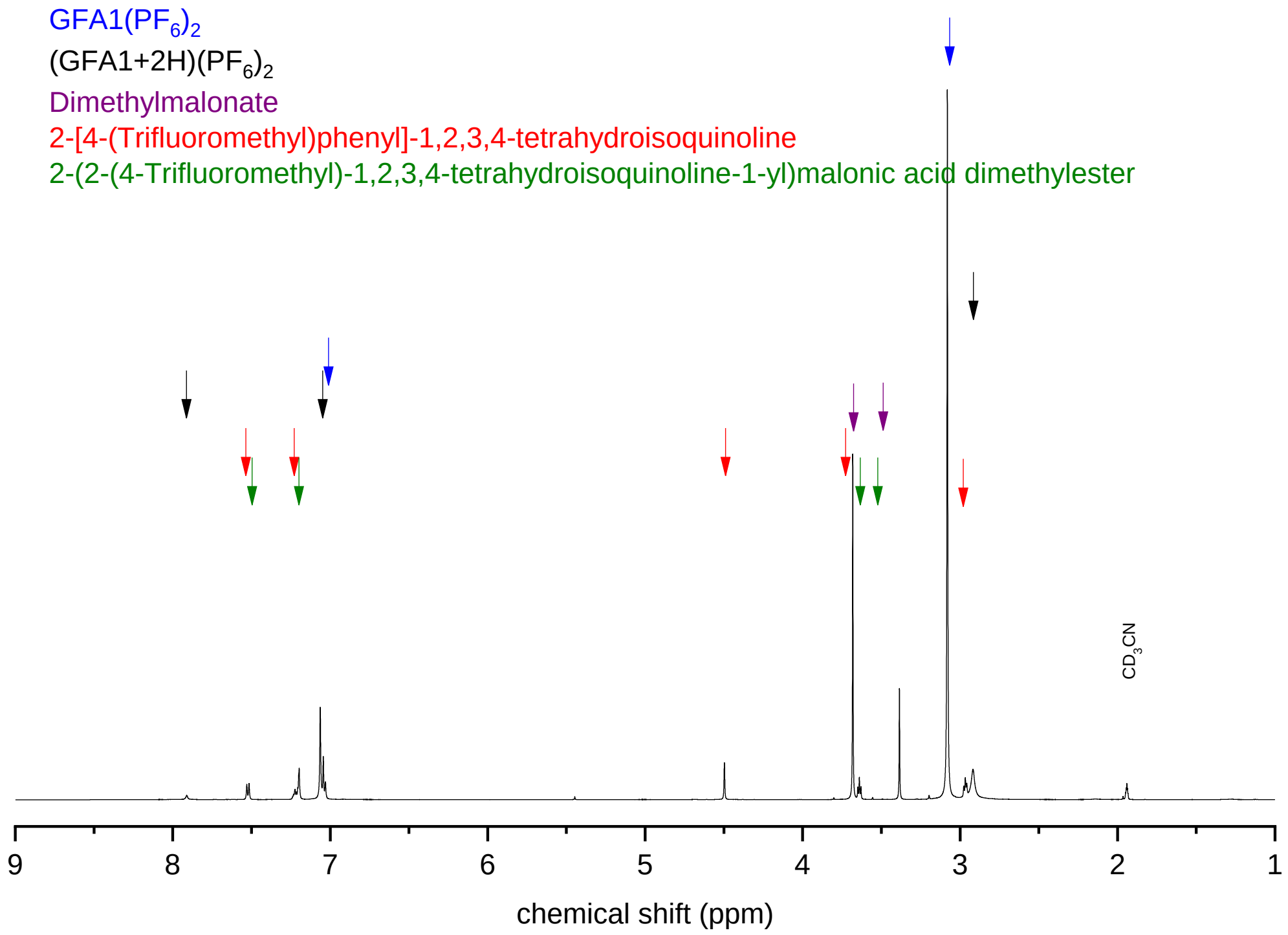

^{1}H NMR spectrum (400.20 MHz, CD_3CN, 298 K) for entry **G2** last measurement (20 h, 50 °C)

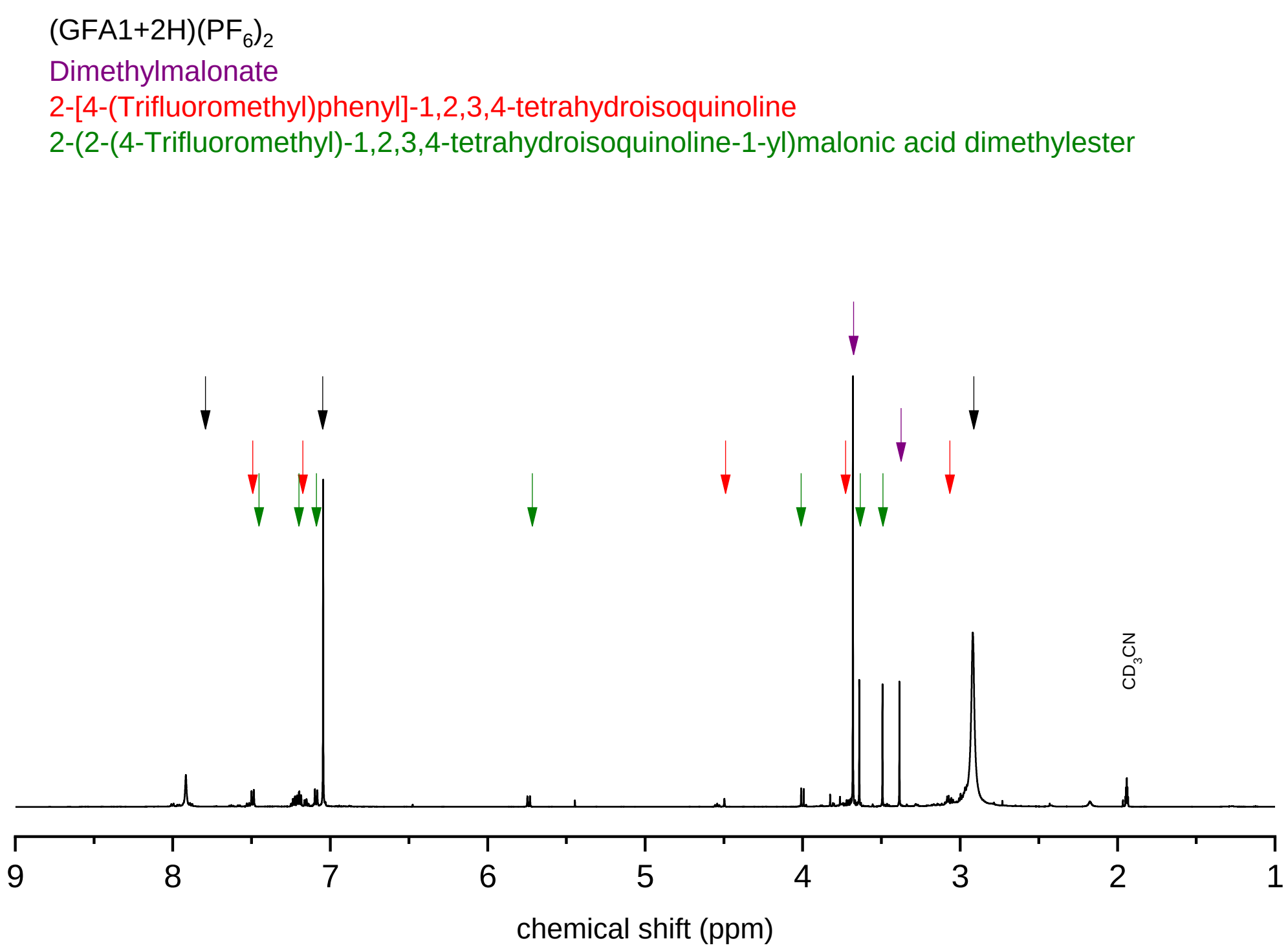

^{1}H NMR spectrum (600.15 MHz, $CDCl_3$, 298 K) for entry **G2** after workup

2-[4-(Trifluoromethyl)phenyl]-1,2,3,4-tetrahydroisoquinoline
2-(2-(4-Trifluoromethyl)-1,2,3,4-tetrahydroisoquinoline-1-yl)malonic acid dimethylester

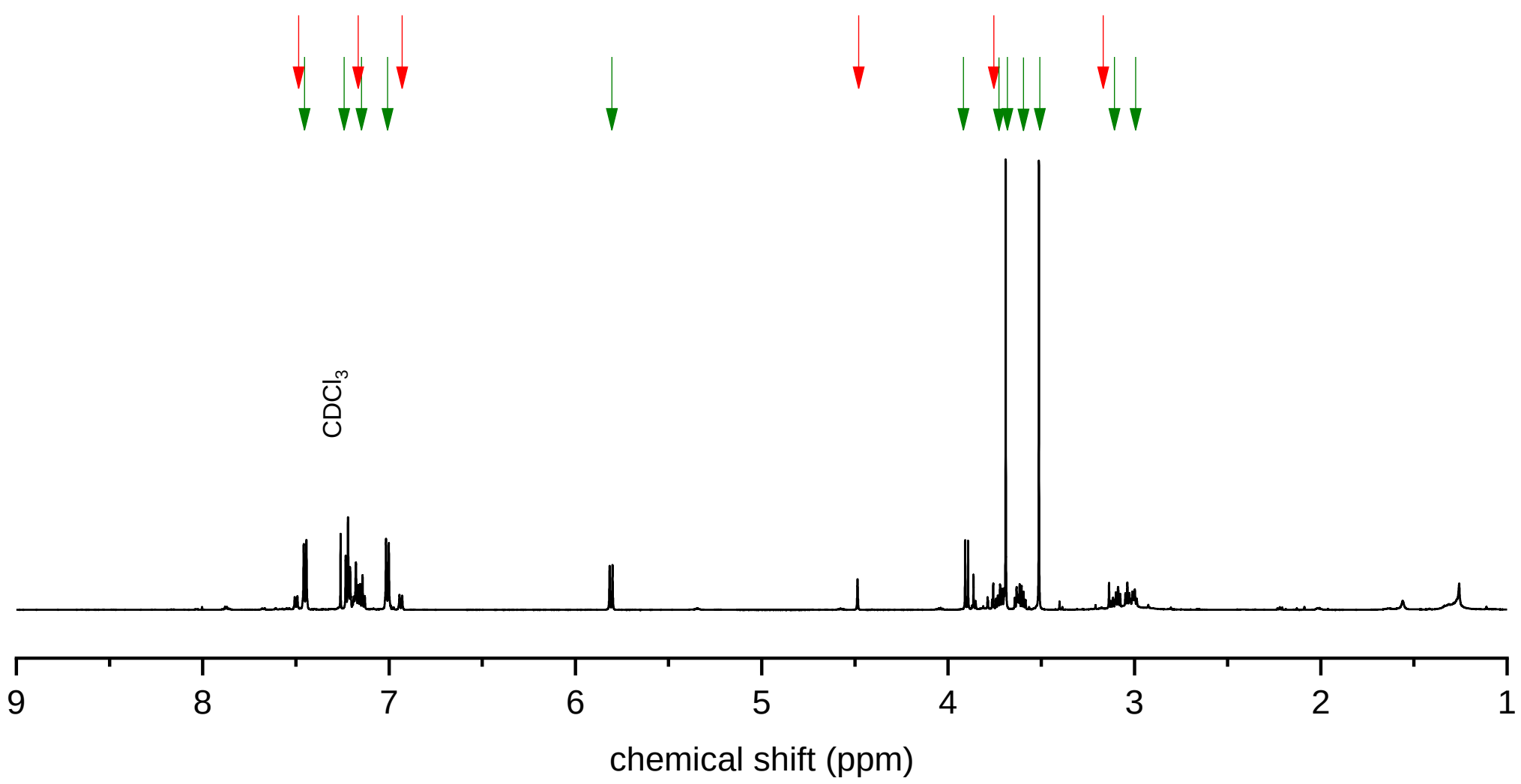

Reaction between 2-(4-methoxyphenyl)-1,2,3,4-tetrahydroisoquinoline (PTQ-OMe) and dimethylmalonate

entry	H2
PTQ-OMe [mg / µmol]	5.414 / 22.6
dimethylmalonate [µl / µmol]	5.2 / 45
CD_3CN [ml]	0.5
solution I [ml]	0.24
GFA1$(PF_6)_2$ [mg / µmol]	28.8 / 48
ratio PTQ-OMe / GFA1^{2+} / dimethylmalonate	1 / 2 / 2
condition	NMR tube
reaction time (temperature)	1 h (r.t.)
Yield after workup [%]	79

Protocol for entry H2: First, a solution of 28.8 mg (48 µmol) GFA1$(PF_6)_2$ in 0.24 ml CD_3CN ($c = 2.0 \cdot 10^{-1}$ mol·l^{-1}) was prepared (solution I). In an NMR tube, the two reactants PTQ-OMe and dimethylmalonate were dissolved in CD_3CN. After addition of solution I, conversion was followed by ^{1}H NMR spectroscopy. Then, the solvent and excess dimethylmalonate were removed under high vacuum. The residue was suspended in CH_2Cl_2, filtrated over syringe filter and concentrated to give 6.81 mg of 2-[1,2,3,4-tetrahydro-2-(4-methoxyphenyl) isoquinoline-1-yl] malonic acid dimethylester (mixture product / reactant = 95 / 5, 17.83 µmol, 79%) as a yellow oil. ^{1}H NMR (600.15 MHz, $CDCl_3$): δ = 7.18 (dd, *J* = 16.00, 7.47 Hz, 2 H), 7.11 (d, *J* = 5.94 Hz, 2 H), 6.95 - 6.88 (m, 2 H), 6.81 - 6.74 (m, 2 H), 5.49 (d, *J* = 9.40 Hz, 1 H), 3.95 (d, *J* = 9.40 Hz, 1 H), 3.73 (s, 3 H), 3.67 (dd, *J* = 8.65, 3.39 Hz, 1 H), 3.63 (s, 3 H), 3.60 (s, 3 H), 3.55 (ddd, *J* = 9.98, 5.67, 3.76 Hz, 1 H), 3.07 - 2.92 (m, 1 H), 2.76 (td, *J* = 16.62, 4.36, 4.36 Hz, 1 H) ppm. The analytical data matched those in the literature.[8]

The insoluble components consisted of (GFA1+2H)$(PF_6)_2$ in high purity (21.2 mg, 74%).

^1H NMR spectrum (400.20 MHz, CD_3CN, 298 K) for entry **H2** first measurement (10 min, r.t.)

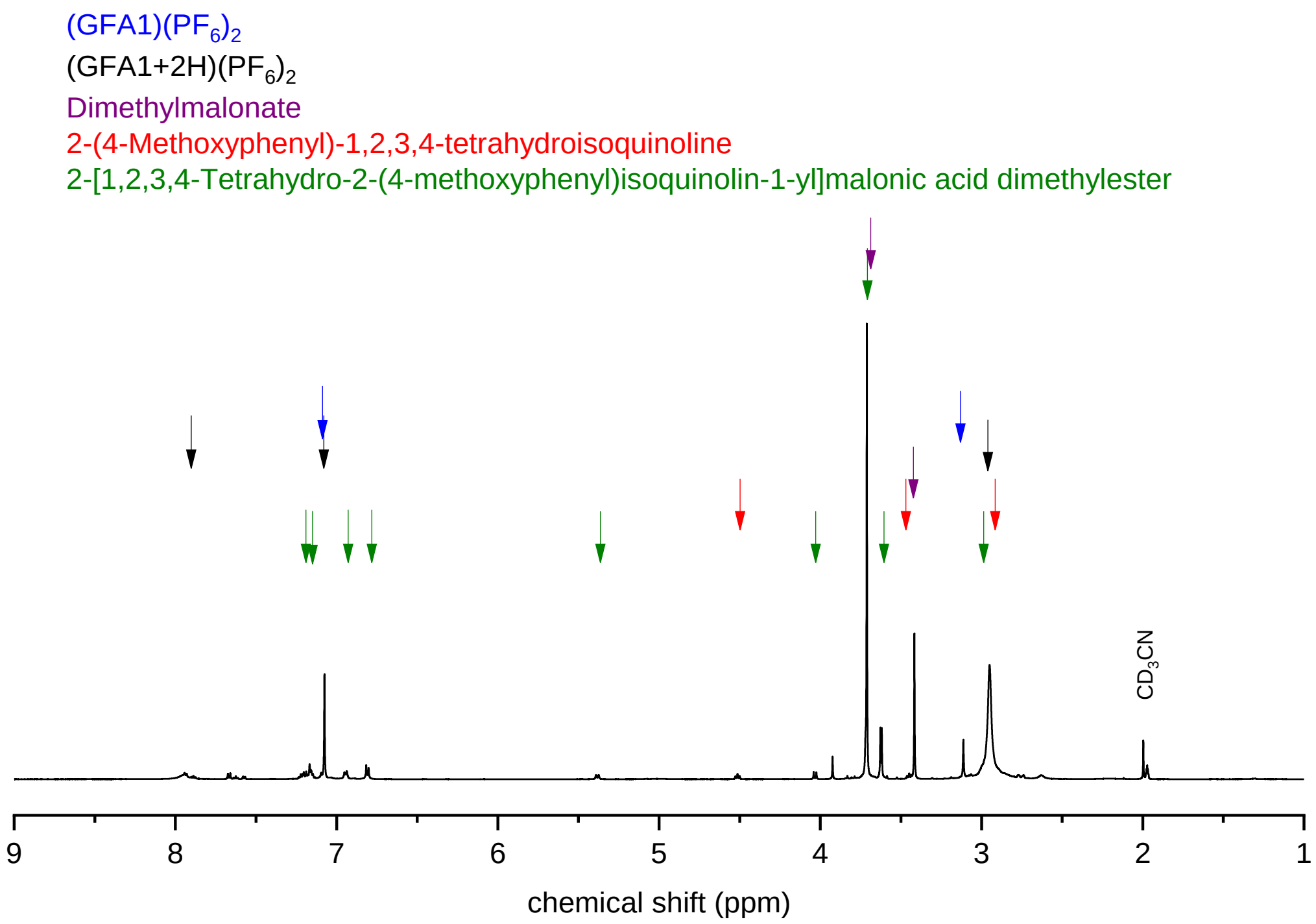

^1H NMR spectrum (400.20 MHz, CD_3CN, 298 K) for entry **H2** last measurement (1 h, r.t.)

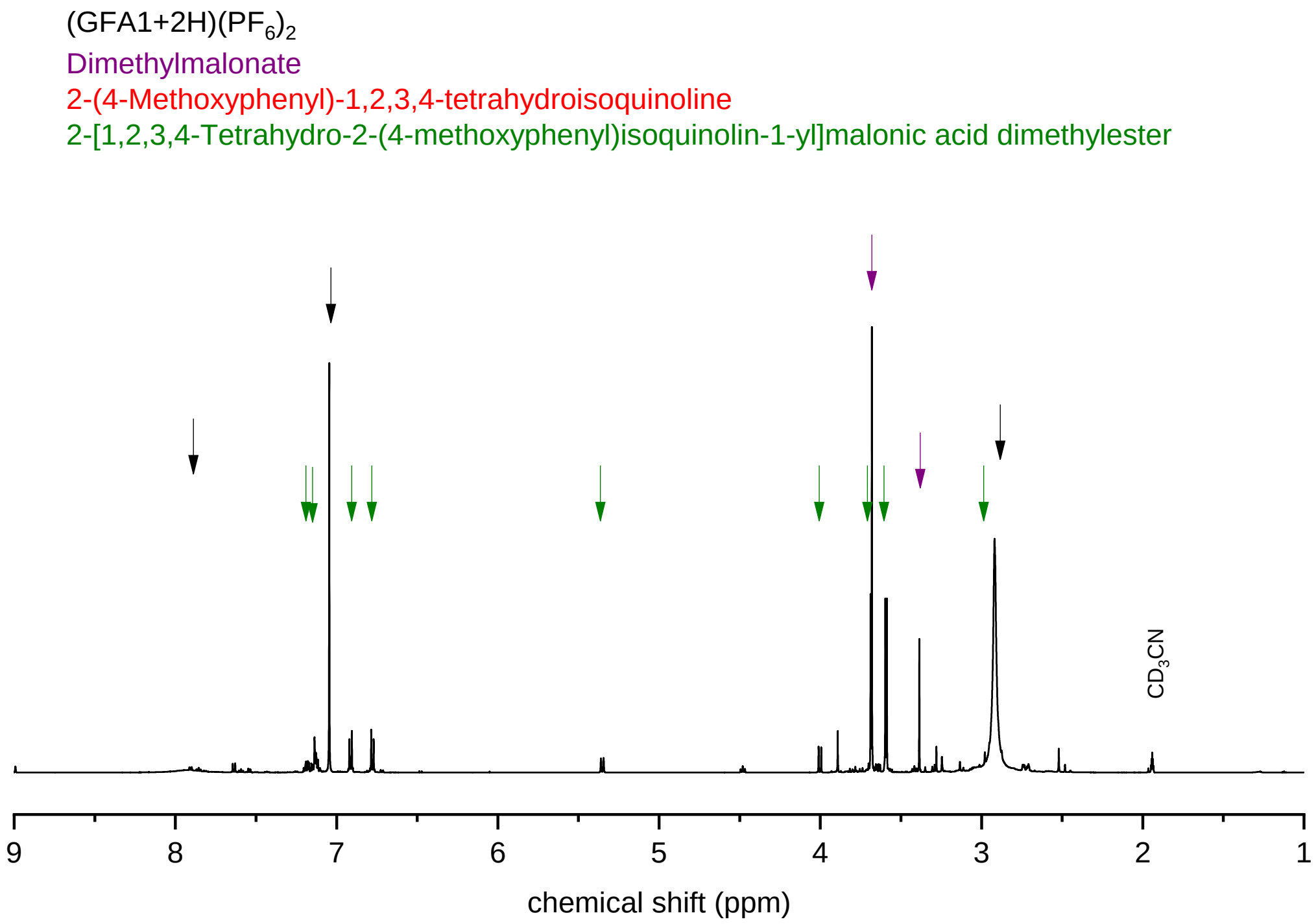

^{1}H NMR spectrum (600.15 MHz, $CDCl_3$, 298 K) for entry **H2** after workup

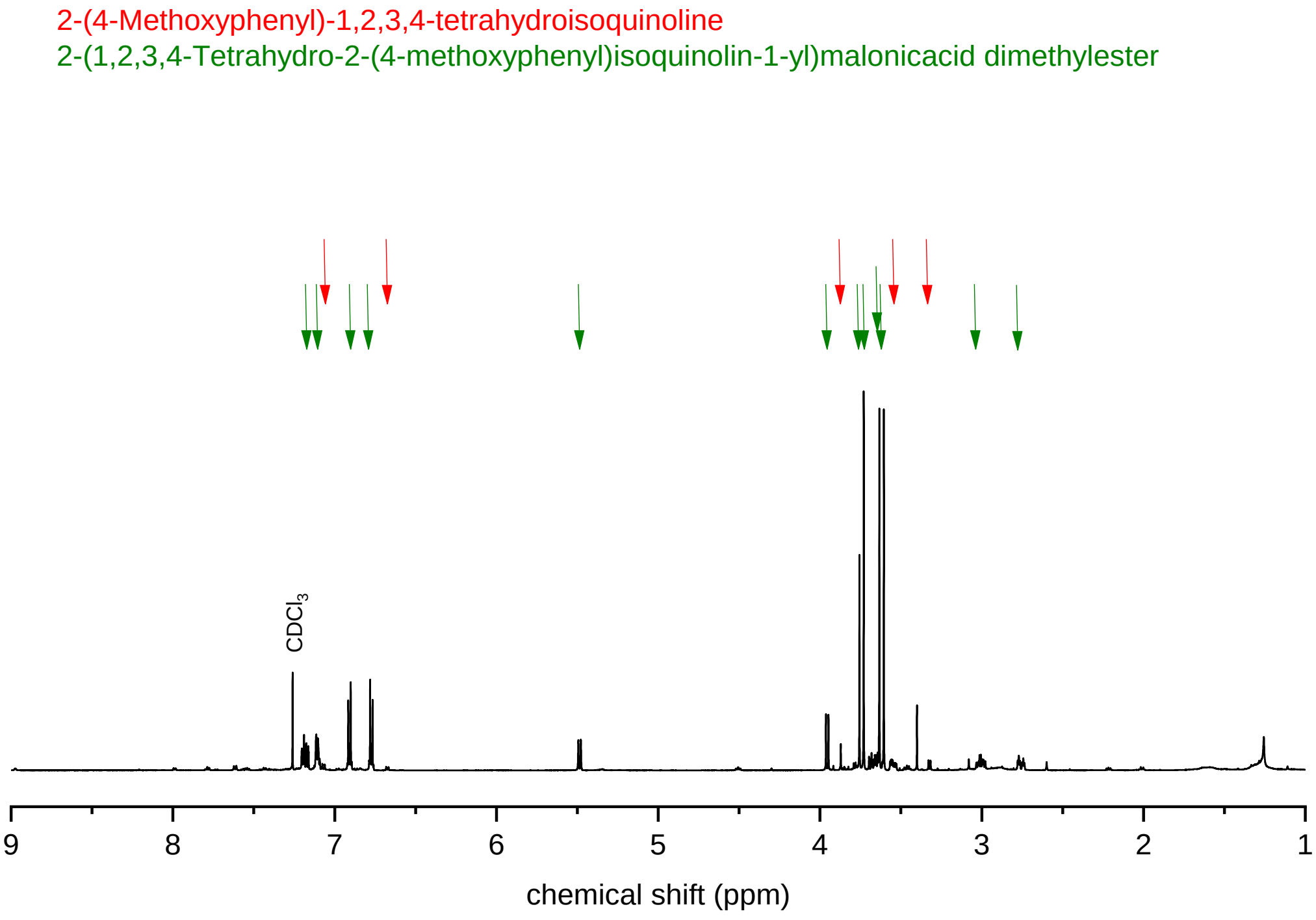

^{1}H NMR spectrum (600.15 MHz, CD_3CN, 298 K) for entry **H2,** separation of $(GFA1+2H)(PF_6)_2$

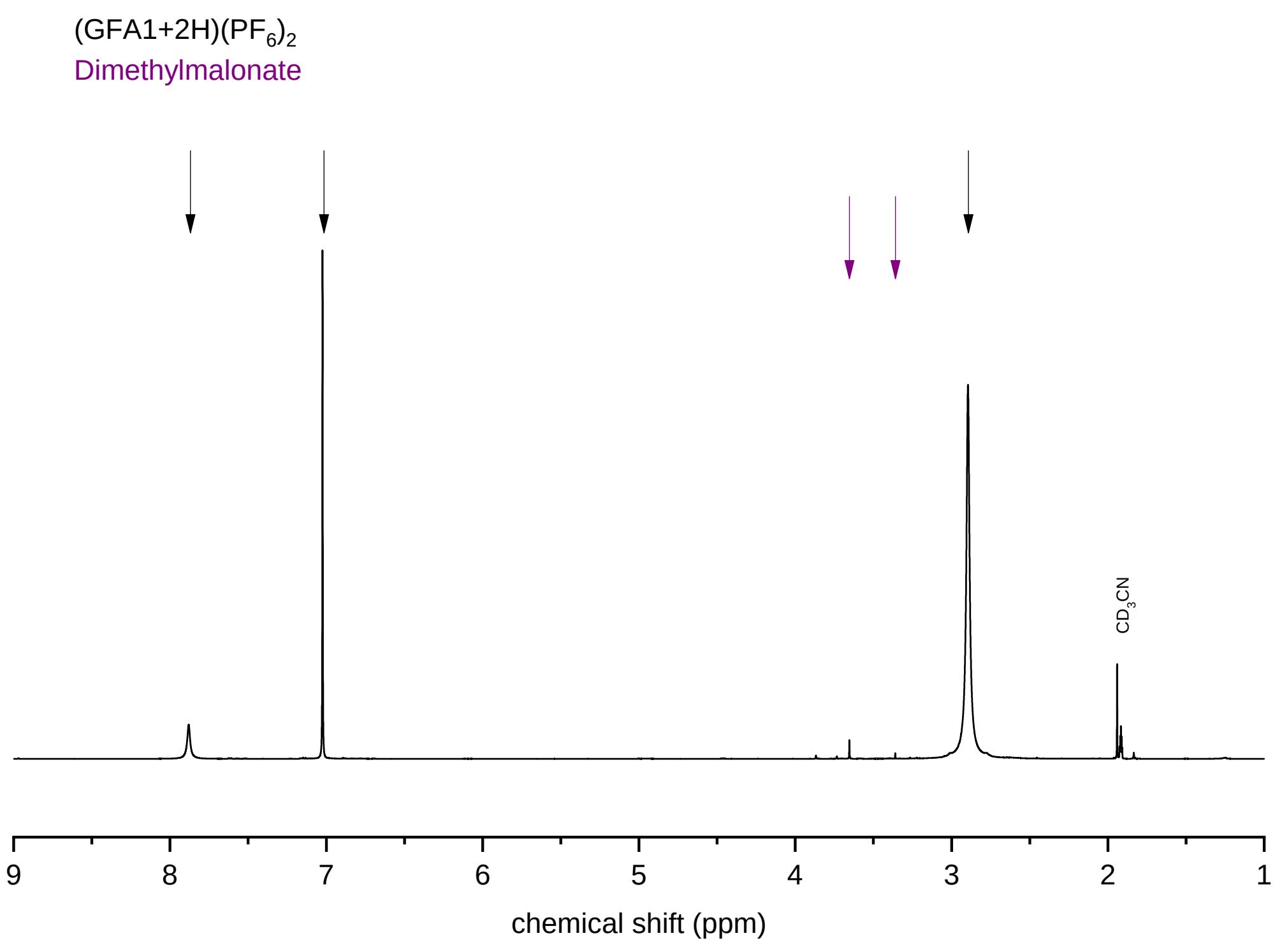

Reaction with DDQ as CDC reagent for comparison

entry	J2
PTQ-OMe [mg / µmol]	5.406 / 22.5
dimethylmalonate [µl / µmol]	5.2 / 45
CH_3CN [ml]	0.5
solution I [ml]	0.22
DDQ [mg / µmol]	10.038 / 44.2
ratio PTQ-OMe / DDQ / dimethylmalonate	1 / 2 / 2
condition	Schlenk flask
reaction time (temperature)	1 h (r.t.)
Conversion after workup	no conversion

General protocol: First a solution of 10.038 mg (44.2 µmol) DDQ in 0.22 ml CH_3CN ($c = 2.0 \cdot 10^{-1}$ mol·l^{-1}) was prepared (solution I). In an Schlenk flask, the two reactants PTQ-OMe and dimethylmalonate were dissolved in CH_3CN. After addition of solution I the reaction was stirred for 1 h at room temperature. The reaction was stopped by the addition of an aqueous saturated $NaHCO_3$ solution. The small amount of CH_3CN was removed under high vacuum. The obtained solution was extracted several times with CH_2Cl_2. The combined organic phases were dried over $MgSO_4$, filtered and concentrated.

^{1}H NMR spectra (600.18 MHz, 298 K) for entry **J2**

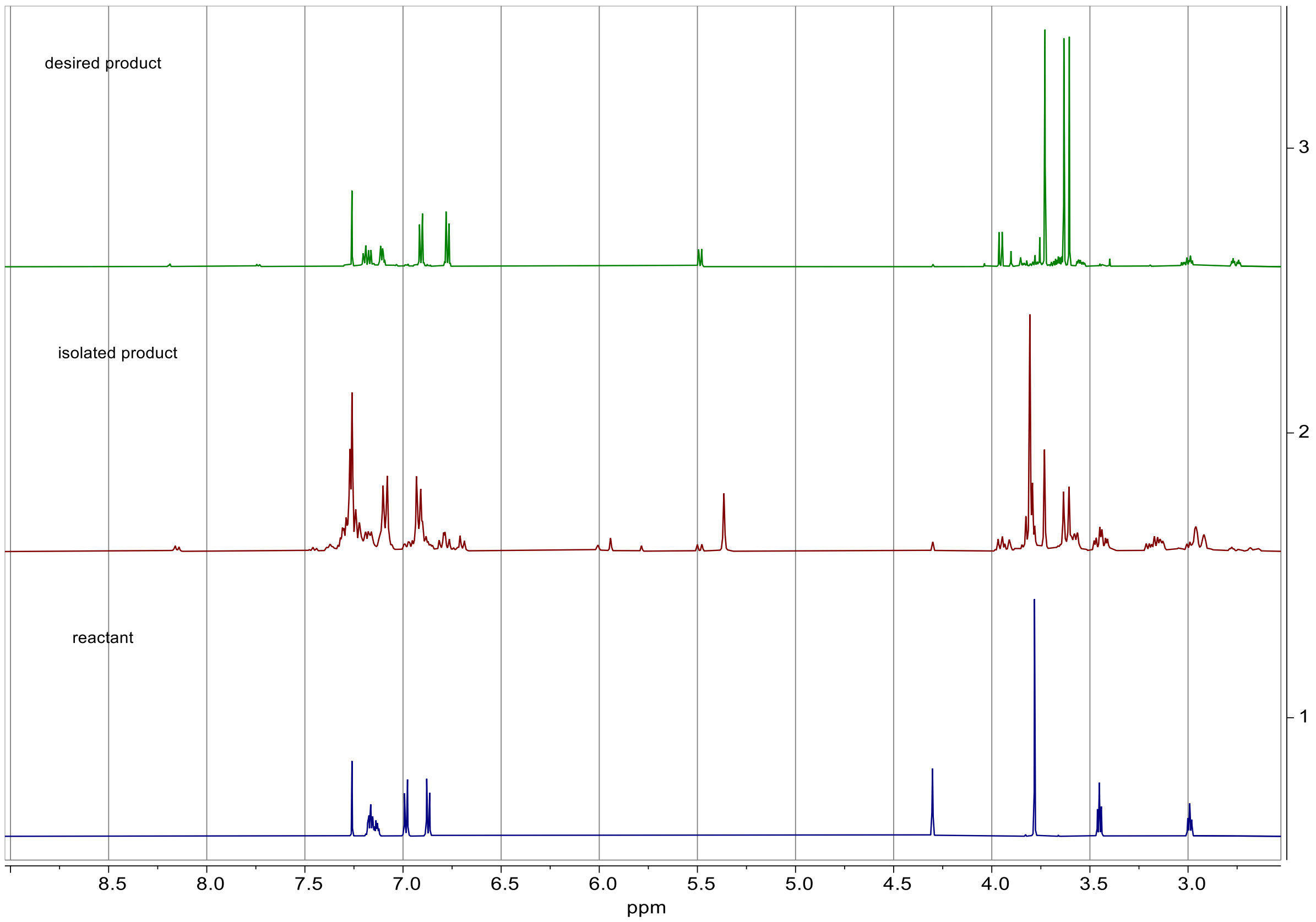

4.3 Reactions with methylphenyl ketone

4.3.1 Methylphenyl ketone as solvent

Reaction between 2-phenyl-1,2,3,4-tetrahydroisoquinoline (PTQ) and methylphenyl ketone to give 1-phenyl-2-(2-phenyl-1,2,3,4-tetrahydroisoquinoline-1-yl)ethenone

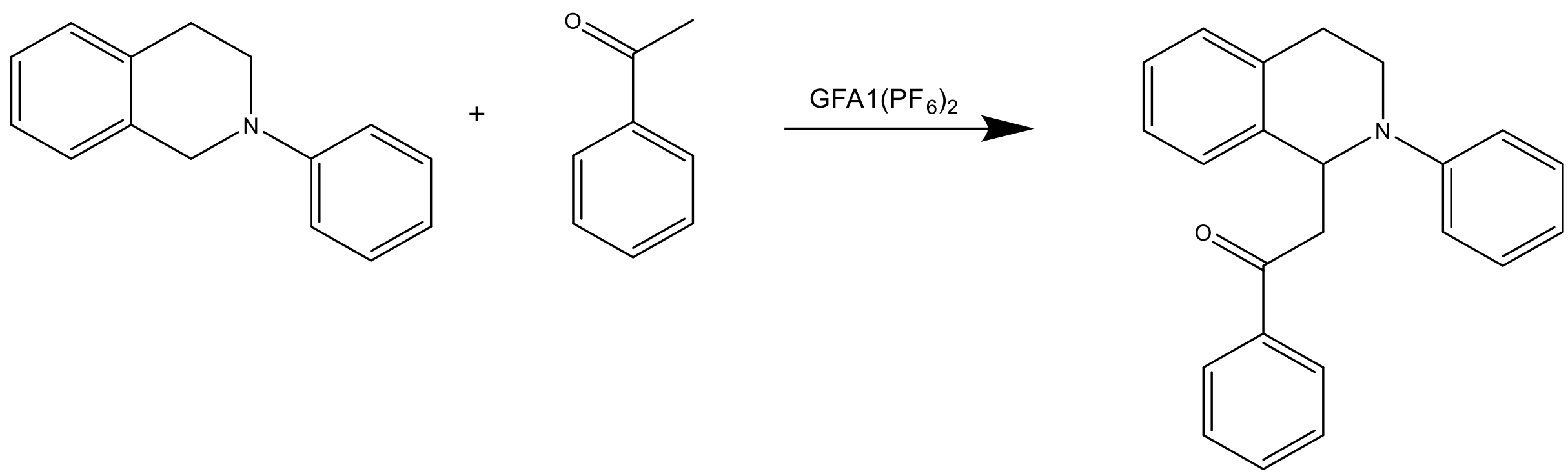

entry	A3	B3
$GFA1(PF_6)_2$ [mg / µmol]	13.271 / 22	21.942 / 37
PTQ [mg / µmol]	2.692 / 13	3.864 / 18.5
methyl phenyl ketone [µl / µmol]	500 / 4285	400 / 3428
ratio PTQ / $GFA1^{2+}$ / methyl phenyl ketone	1 / 1.7 / 330	1 / 2 / 185
condition	Schlenk flask	Schlenk flask
reaction time (temperature)	2 h (r.t.)	4 h (50 °C)
Yield after workup [%]	no conversion	68

Protocol for entry A3: The reactants were dissolved in methylphenyl ketone at 50 °C and stirred 2 h at this temperature. Subsequently the excess of methyl phenyl ketone was removed under high vacuum. The residue was suspended in chloroform, filtered through a syringe filter and condensed. The insoluble component consisted of $(GFA1{+}2H)(PF_6)_2$ in high purity.

Protocol for entry B3: The reactants were dissolved in methylphenyl ketone at 50 °C and stirred 4 h at this temperature. Afterwards, excess methylphenyl ketone was removed in vacuum. The residue was suspended in chloroform and water (1:1) and the aqueous phase was extracted three times with chloroform. The combined organic phases were dried over $MgSO_4$, filtrated and condensed to give 4.45 mg of 1-phenyl-2-(2-phenyl-1,2,3,4-tetrahydro-isoquinoline-1-yl)ethenone (mixture: product / reactant = 89 / 11, 13.9 µmol, 68%) as a brownish-yellow oil. ^1H NMR (600 MHz, $CDCl_3$): δ = 7.85 (dd, J = 8.4, 1.3 Hz, 2 H), 7.54 - 7.50

(m, 1 H), 7.42 - 7.38 (m, 2 H), 7.15 (dd, *J* = 4.0, 1.0 Hz, 2 H), 6.98 - 6.95 (m, 2 H), 6.77 - 6.73 (m, 1 H), 5.67 (dd, *J* = 7.3, 5.0 Hz, 1 H), 3.69 - 3.60 (m, 2 H), 3.57 (dd, *J* = 16.6, 5.0 Hz, 1 H), 3.40 (dd, *J* = 16.6, 7.3 Hz, 1 H), 3.15 - 3.08 (m, 1 H), 2.93 (dt, *J* = 16.0, 5.2 Hz, 1 H) ppm; ^{13}C NMR (150.92 MHz, $CDCl_3$, 298 K): δ = 198.8, 148.9, 138.7, 137.4, 134.6, 133.2, 129.5, 128.7, 128.3, 127.3, 127.0, 126.4, 118.1, 114.5, 55.2, 45.5, 42.3, 27.7 ppm. The analytical data matched those in the literature.[10]

Separation of GFA1 for entry B3: The aqueous phase contained (GFA1+2H)$(PF_6)_2$ that was deprotonated with 25% NaOH and extracted with chloroform. The combined organic phases were dried over K_2CO_3, filtrated and condensed to give (10.3 mg, 33.8 µmol, 91%) of 1,4-bis(tetramethylguanidino)benzene (GFA1) as an off-white solid.

^{1}H NMR spectrum (600.15 MHz, $CDCl_3$, 298 K) for entry **B3**

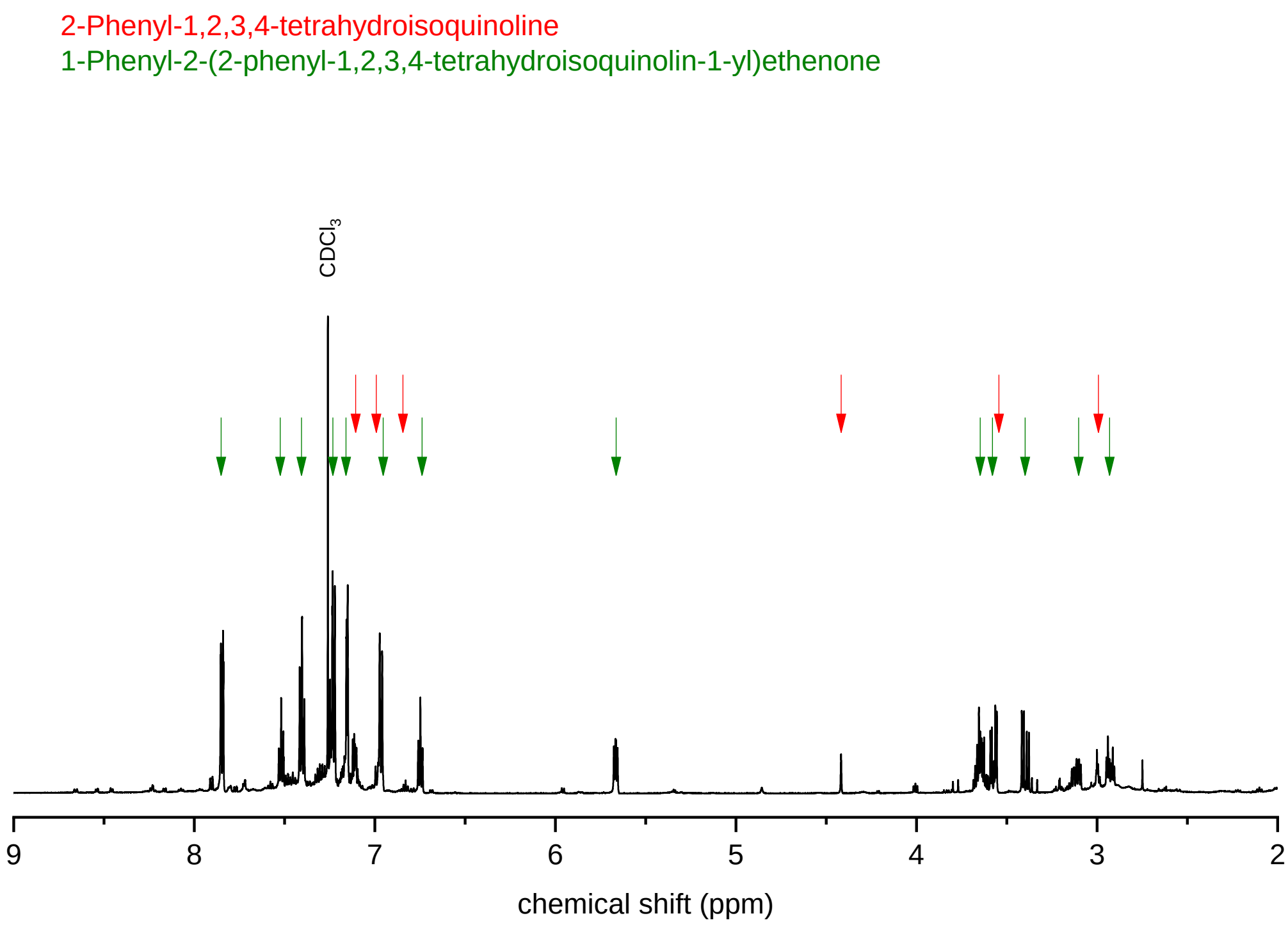

^{13}C NMR spectrum (150.92 MHz, $CDCl_3$, 298 K) for entry **B3** after workup

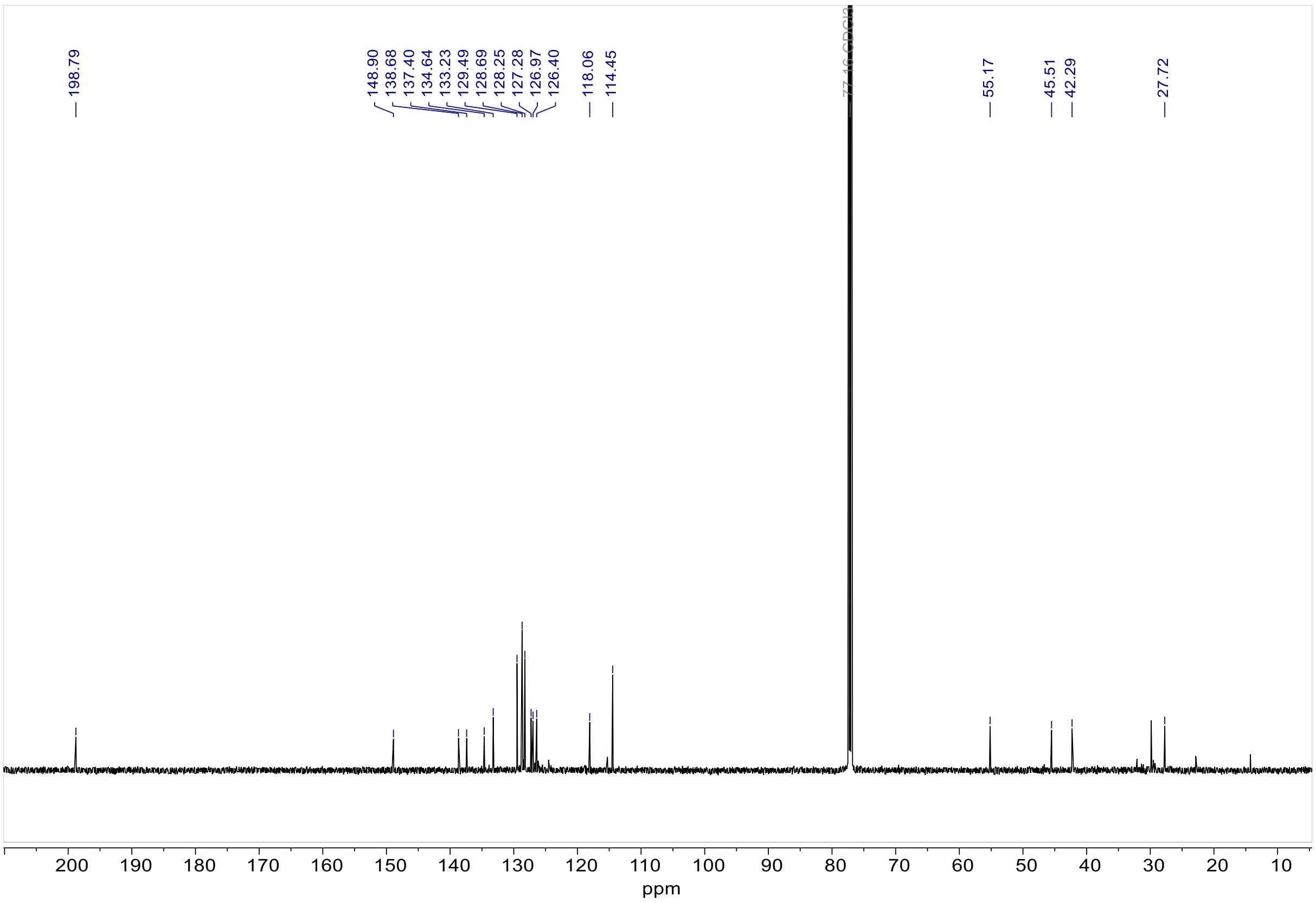

Reaction between 2-(4-methoxyphenyl)-1,2,3,4-tetrahydroisoquinoline (PTQ-OMe) and methylphenyl ketone to give 1-(4-methoxyphenyl)-2-(2-phenyl-1,2,3,4-tetrahydroisoquinoline-1-yl)ethenone

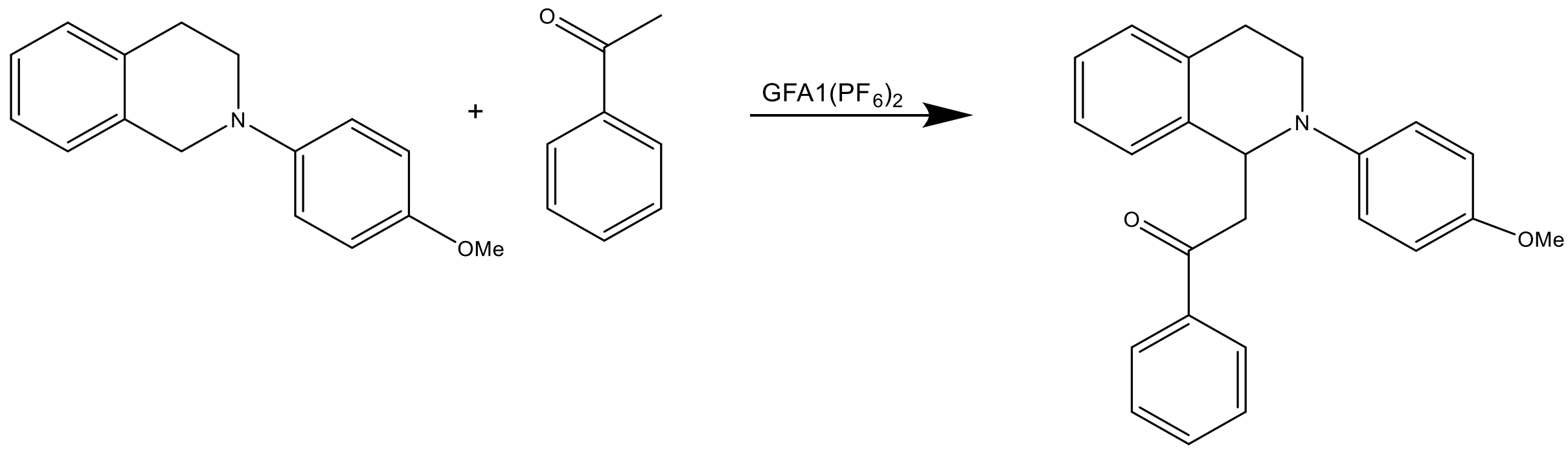

entry	C3
$GFA1(PF_6)_2$ [mg / µmol]	19.216 / 32.2
PTQ-OMe [mg / µmol]	4.354 / 18.2
methyl phenyl ketone [µl / µmol]	500 / 4285
ratio PTQ-OMe / $GFA1^{2+}$ / methyl phenyl ketone	1 / 1.8 / 240
condition	Schlenk flask
reaction time (temperature)	6 h (r.t.)
Yield after workup [%]	69

Protocol for entry C3: The reactants were dissolved in methylphenyl ketone at room temperature and stirred for 6 h at this temperature. Subsequently, excess methylphenyl ketone was removed under high vacuum. The residue was suspended in chloroform and water (1:1) and the aqueous phase was extracted three times with chloroform. The combined organic phases were dried over $MgSO_4$, filtrated and condensed to give 4.62 mg of 1-phenyl-2-(2-phenyl-1,2,3,4-tetrahydroisoquinoline-1-yl)ethenone (mixture product / reactant = 95 / 5, 12.5 µmol, 69%) as a brownish-yellow oil. ^1H NMR (600 MHz, $CDCl_3$) δ = 7.86 - 7.80 (m, 2 H), 7.51 (m, 1 H), 7.40 (t, *J* = 7.8 Hz, 2 H), 7.19 (d, *J* = 7.5 Hz, 1 H), 7.16 - 7.09 (m, 3 H), 6.93 (d, *J* = 8.9 Hz, 2 H), 6.79 (d, *J* = 9.0 Hz, 2 H), 5.52 (t, *J* = 6.1 Hz, 1 H), 3.73 (s, 3 H), 3.56 (d, *J* = 10.5 Hz, 3 H), 3.30 (dd, *J* = 16.3, 6.6 Hz, 1 H), 3.08 (m, 1 H), 2.83 (dt, *J* = 16.3, 4.2 Hz, 1 H) ppm; ^{13}C NMR (150.92 MHz, $CDCl_3$, 298 K): δ = 198.9, 153.2, 143.7, 138.7, 137.5, 134.4, 133.1, 129.0, 128.6, 128.2, 127.2, 126.8, 126.3, 118.0, 114.8, 56.4, 55.8, 45.1, 42.9, 27.4 ppm. The analytical data matched those in the literature.[10]

The insoluble components consisted of $(GFA1+2H)(PF_6)_2$ in high purity.

^{1}H NMR spectrum (600.15 MHz, $CDCl_3$, 298 K) for entry **C3** after workup

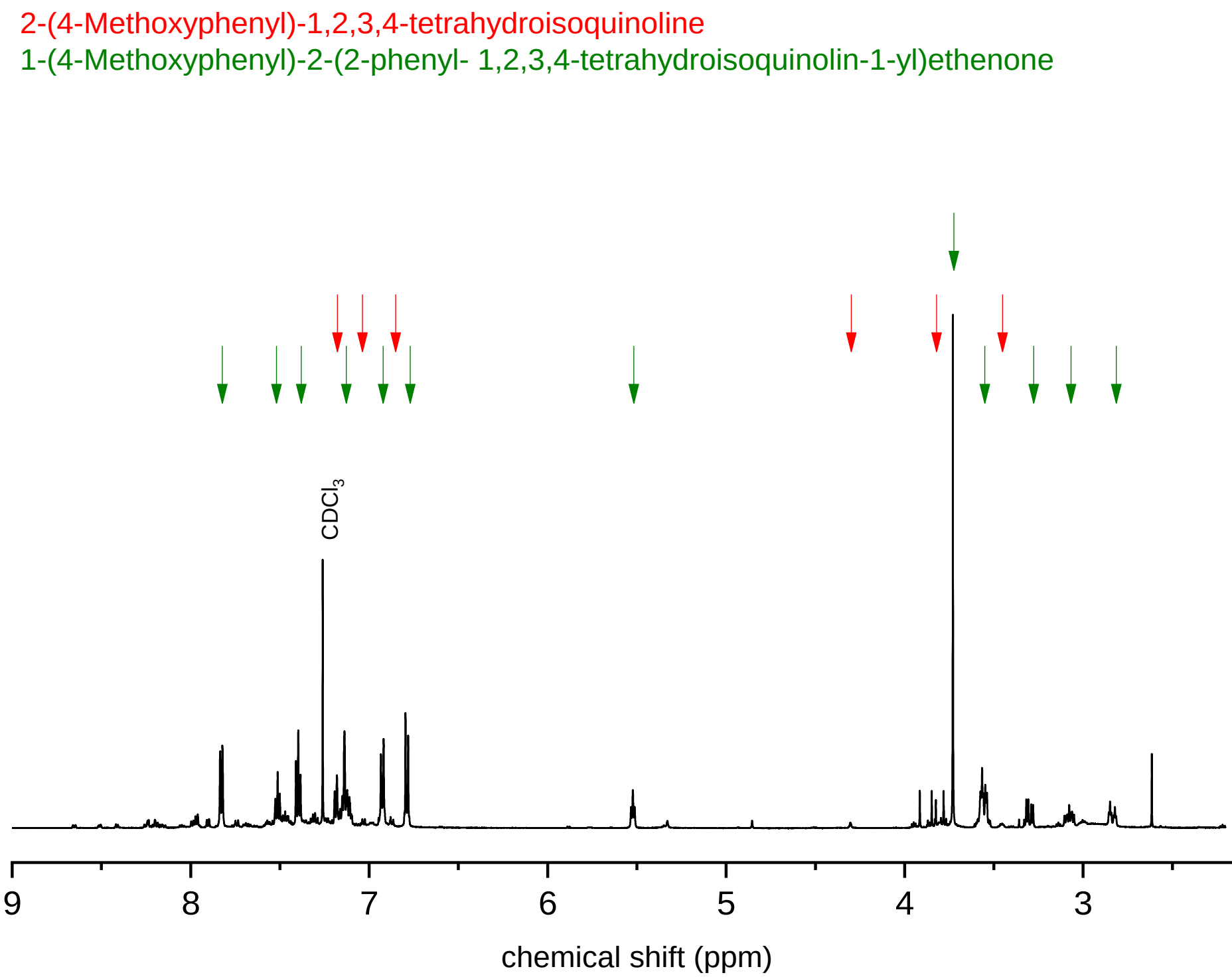

^{13}C NMR spectrum (150.92 MHz, $CDCl_3$, 298 K) for entry **C3** after workup

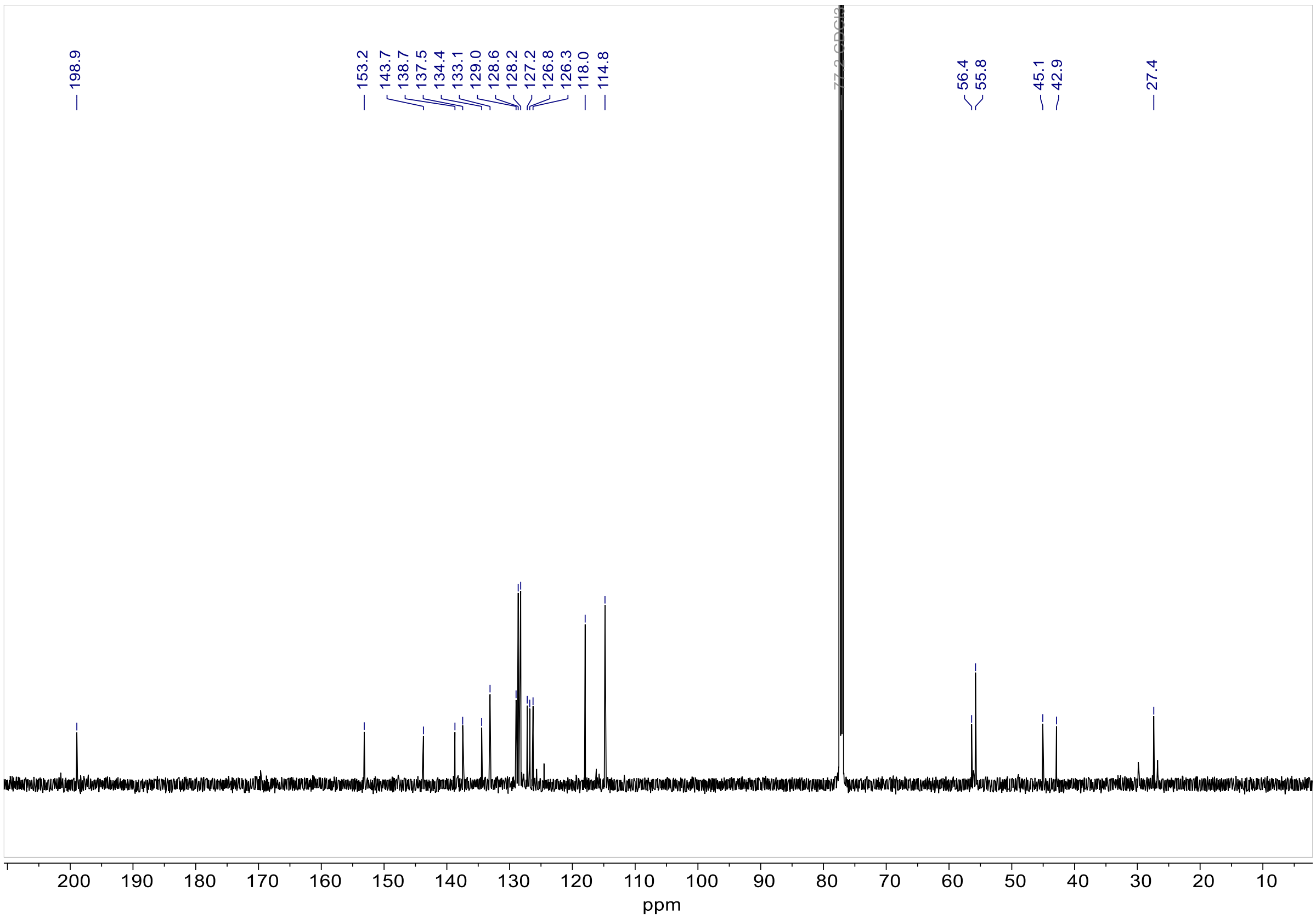

UV-Vis experiment

Reaction between DDQ and malonic acid dimethylester

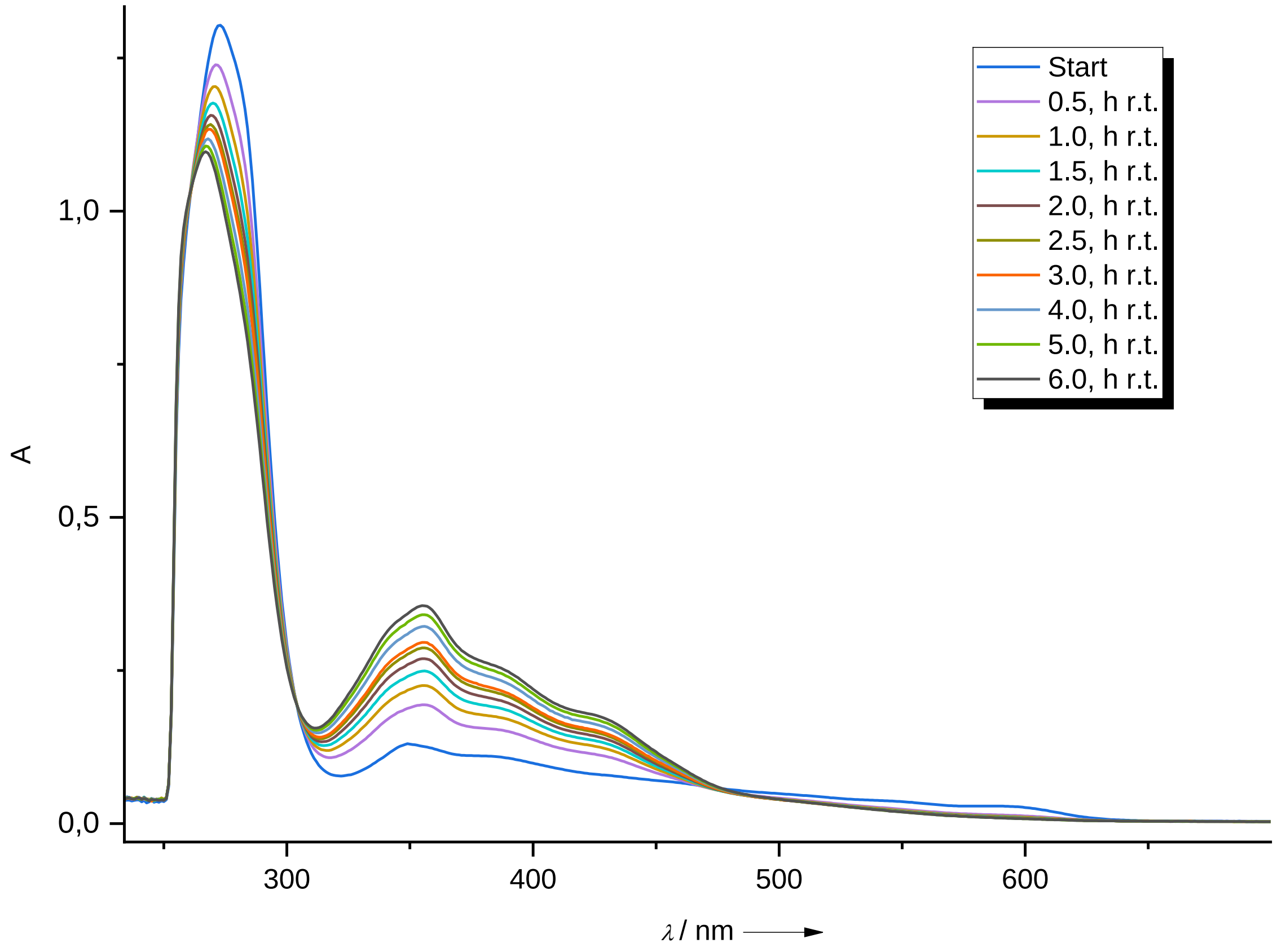

First a malonic acid dimethylester baseline was recorded

Protocol: 3 mL malonic acid dimethylester and a small amount of DDQ were mixed under stirring in a 1 cm quartz cuvette equipped with a silicon septum. UV-vis spectra were taken after constant time intervals.

5 Quantum-chemical calculations

Computational details

Density functional calculations are performed with the program TURBOMOLE [11,12,13,14]. The B3LYP functional [15,16] is used in connection with the def2-SV(P) and def2-TZVP basis sets [17]. The two-electron integrals are calculated using the resolution-of-the-identity (RI) approximation [18] in connection with the appropriate def2-SV(P) and def2-TZVP auxiliary basis sets [19]. The calculations include the D3 dispersion correction with Becke-Johnson damping [20,21]. Harmonic vibrational frequencies [22] are calculated.

Results

The calculations considered two conformations of PTQ, denoted K1 and K2, that differ by the rotation of the phenyl group. The conformation with the plane of the phenyl ring being nearly coplanar with the N-C bond closer to the aromatic ring of the isoquinoline moiety (K1) is lower in energy. In the other conformation the plane of the phenyl ring is nearly coplanar with the other N-C bond. (Only the former lower-energy conformation is used in the calculations on the PTQ-CF_3 and PTQ-OMe derivatives.)

For the coupling product 1-(nitromethyl)-2-phenyl-1,2,3,4-tetrahydroisoquinoline (denoted PTQ-CH_2NO_2 in the following discussion) of the reaction with CH_3NO_2, the calculations considered the two configurations with the CH_2NO_2 unit being located either above or below the plane of the isoquinoline, when the isoquinoline is placed in such a way that the N atom takes a lower right position, and then, for each of the two configurations three conformations are considered that differ by rotation of the CH_2NO_2 group around the C-C axis. These isomers are denoted by the letter I followed by two digits, the first indicating the position of the CH_2NO_2 unit above (1) or below (2) the tetrahydro isoquinoline plane, the second (1, 2, 3) indicating rotations of CH_2NO_2 around the C-C axis. The lowest-energy isomer of PTQ-CH_2NO_2 (I22) has a structure where the NO_2 group takes a position trans with respect to the framework N atom. In the isomer next-higher in energy (I13), the NO_2 group takes a position trans to the aromatic ring of the tetrahydro isoquinoline moiety.

Concerning the orientation of the phenyl ring in PTQ-CF_3 and PTQ-OMe, only the conformation corresponding to the lowest-lying one of PTQ has been considered. Furthermore, for PTQ-CF_3, there are two conformations (K1, K2) very close in energy that differ by rotation of the CF_3 group, and likewise, for PTQ-OMe, there are two conformations (K1, K2) that differ in the rotation of the OCH_3 group.

The structures of the products 1-(nitromethyl)-2-[4-(trifluoromethyl)phenyl-1,2,3,4-tetrahydro-isoquinoline (PTQ-CF_3-CH_2NO_2) and 1-(nitromethyl)-2-(4-methoxyphenyl) -1,2,3,4-tetrahydroisoquinoline (PTQ-OMe-CH_2NO_2) have been calculated starting from the two lowest-energy structures of PTQ-CH_2NO_2 by addition of the CF_3 or OCH_3 group. Accordingly, the notation of the isomers uses a combination of the notations used for PTQ-CH_2NO_2 and PTQ-CF_3 or PTQ-OMe.

The electronic reaction energies, the reaction enthalpies at 0 and 298 K, and the reaction free enthalpies at 298 K obtained by the calculations are shown in Table 1. The total electronic energies are collected in Table 2, and the thermodynamic contributions are shown in Table 3.

Table 1. Electronic reaction energies, reaction enthalpies at 0 and 298 K, and reaction free enthalpies at 298 K (E / kJ mol^{-1})

Reaction	Basis set	$\Delta_r E$	$\Delta_r H_0$	$\Delta_r H_{298}$	$\Delta_r G_{298}$
GFA1$(PF_6)_2$ + PTQ + CH_3NO_2 → (GFA1+2H)$(PF_6)_2$ + PTQ-CH_2NO_2	SV(P)	-244.7	-222.3	-227.9	-160.0
	TZVP	-225.0	-204.2	-209.3	-139.9
GFA1$(PF_6)_2$ + PTQ-CF_3 + CH_3NO_2 → (GFA1+2H)(PF_6)2 + PTQ-CF_3-CH_2NO_2	SV(P)	-243.3	-221.1	-226.7	-158.5
	TZVP	-223.7	-202.9	-208.2	-137.3
GFA1$(PF_6)_2$ + PTQ-OMe + CH_3NO_2 → (GFA1+2H)$(PF_6)_2$ + PTQ-OMe-CH_2NO_2	SV(P)	-244.0	-221.9	-227.2	-160.1
	TZVP	-223.7	-203.2	-208.1	-139.5

Table 2. Calculated electronic energies by density functional calculations with the B3LYP functional including the D3 dispersion correction.

System	Isom.	Sym.	Term	*E*(SV(P))/Hartree	*E*(TZVP)/Hartree
GFA1$(PF_6)_2$		C_i	^1A_g	-2833.317908	-2836.223219
(GFA1+2H)$(PF_6)_2$		C_i	^1A_g	-2834.584580	-2837.498600
CH_3NO_2		C_1	^1A	-244.705458	-245.007489
PTQ	K1	C_1	^1A	-634.549917	-635.265675
	K2	C_1	^1A	-634.548473	-635.264492
PTQ-CH_2NO_2	I11	C_1	^1A	-878.079110	-879.080948
	I12	C_1	^1A	-878.075317	-879.077465
	I13	C_1	^1A	-878.079608	-879.082218
	I21	C_1	^1A	-878.078844	-879.079935
	I22	C_1	^1A	-878.081904	-879.083483
	I23	C_1	^1A	-878.078048	-879.080159
PTQ-CF_3	K1	C_1	^1A	-971.227454	-972.347054
	K2	C_1	^1A	-971.227452	-972.347046
PTQ-CF_3-CH_2NO_2	I13K1	C_1	^1A	-1214.756665	-1216.163039
	I13K2	C_1	^1A	-1214.756667	-1216.163170
	I22K1	C_1	^1A	-1214.758924	-1216.164369
	I22K2	C_1	^1A	-1214.758893	-1216.164364
PTQ-OMe	K1	C_1	^1A	-748.928243	-749.780725
	K2	C_1	^1A	-748.927876	-749.780314
PTQ-OMe-CH_2NO_2	I13K1	C_1	^1A	-992.458032	-993.597171
	I13K2	C_1	^1A	-992.457977	-993.597154
	I22K1	C_1	^1A	-992.459949	-993.598054
	I22K2	C_1	^1A	-992.459751	-993.597881

Table3. Calculated thermodynamic contributions by density functional calculations with the B3LYP functional including the D3 dispersion correction.

System	Isom.	Sym.	Term	$E_{vib,0}$/	dH_{298}/	μ_{298}/	$E_{vib,0}$/	dH_{298}/	μ_{298}/
				Hartree	kJ mol^{-1}	kJ mol^{-1}	Hartree	kJ mol^{-1}	kJ mol^{-1}
GFA1$(PF_6)_2$		C_i	^1A_g	0.481163	1373.16	1065.91	0.478764	1369.17	1049.06
(GFA1+2H)$(PF_6)_2$		C_i	^1A_g	0.506889	1439.37	1133.05	0.504373	1435.24	1119.91
CH_3NO_2		C_1	^1A	0.049840	144.84	54.09	0.049633	144.33	54.58
PTQ	K1	C_1	^1A	0.263055	725.44	587.15	0.262225	723.42	585.18
PTQ-CH_2NO_2	I22	C_1	^1A	0.295689	820.86	658.75	0.294178	817.39	653.97
PTQ-CF_3	K1	C_1	^1A	0.268073	748.34	582.30	0.266925	745.56	578.95
PTQ-CF_3-CH_2NO_2	I22K1	C_1	^1A	0.300673	843.58	654.05	0.298865	839.35	649.10
PTQ-OMe	K1	C_1	^1A	0.295489	817.29	663.71	0.294546	815.09	661.13
PTQ-OMe-CH_2NO_2	I22K1	C_1	^1A	0.328015	912.64	734.51	0.326395	909.02	729.10

Cartesian coordinates of the structures optimized with the def2-TZVP basis set

GFA1(PF_6)$_2$

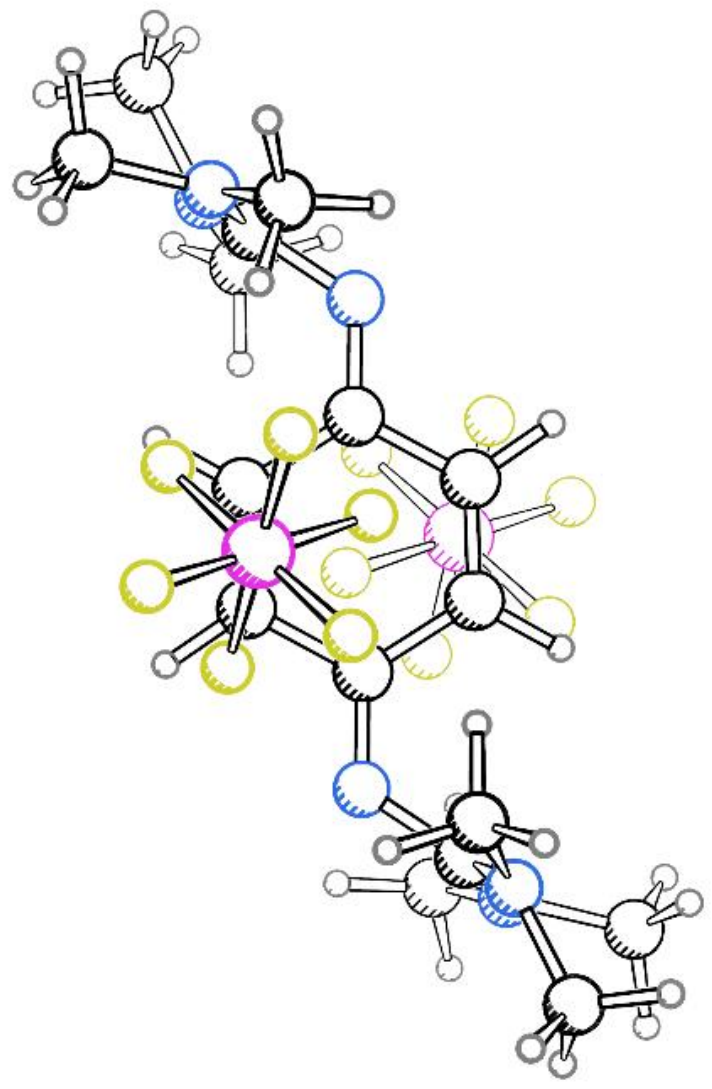

Energy = -2836.223219437

N	-3.9415455	-1.8971503	-0.1822909
C	-2.7793302	-2.3297761	0.3140800
C	-4.0267174	-1.3794891	-1.5561569
C	-5.0997750	-1.5978480	0.6550352
N	-1.6477856	-2.0894186	-0.4075927
N	-2.6831183	-3.0660758	1.4260790
H	-3.9406278	-0.2948294	-1.5682149
H	-4.9946774	-1.6753785	-1.9612446
H	-3.2358050	-1.8110183	-2.1608258
H	-5.9326645	-2.2733165	0.4502627
H	-5.4103880	-0.5764900	0.4320258
H	-4.8365923	-1.6533137	1.7068151
C	-0.8873889	-1.0709722	-0.2030409
C	-1.3635631	-3.3010741	2.0421458
C	-3.7383686	-3.9868591	1.8471363
F	1.4973643	-3.0218955	0.5131083
C	-1.1454737	-0.0775125	0.8318308
C	0.3016907	-0.9334935	-1.0298893
H	-0.7835969	-4.0261258	1.4709978
H	-1.5317850	-3.6775808	3.0480304
H	-0.7846808	-2.3871768	2.1144689
H	-4.1852081	-3.6810803	2.7945163
H	-3.2947019	-4.9749109	1.9756696
H	-4.5109482	-4.0553700	1.0877796
P	2.5760148	-2.4618535	1.6035298

H	-2.0541521	-0.1616109	1.4101040
C	-0.3016907	0.9334935	1.0298893
C	1.1454737	0.0775125	-0.8318308
H	0.4807043	-1.7034850	-1.7648331
F	1.8616769	-3.3366387	2.7594036
F	3.2567131	-1.5382100	0.4228143
F	3.5772032	-3.6626454	1.2527782
F	1.5518407	-1.2193430	1.9348464
F	3.6283832	-1.8495083	2.6688636
C	0.8873889	1.0709722	0.2030409
H	-0.4807043	1.7034850	1.7648331
H	2.0541521	0.1616109	-1.4101040
N	1.6477856	2.0894186	0.4075927
C	2.7793302	2.3297761	-0.3140800
F	-1.4973643	3.0218955	-0.5131083
N	3.9415455	1.8971503	0.1822909
N	2.6831183	3.0660758	-1.4260790
P	-2.5760148	2.4618535	-1.6035298
C	4.0267174	1.3794891	1.5561569
C	5.0997750	1.5978480	-0.6550352
C	1.3635631	3.3010741	-2.0421458
C	3.7383686	3.9868591	-1.8471363
F	-1.8616769	3.3366387	-2.7594036
F	-3.2567131	1.5382100	-0.4228143
F	-3.5772032	3.6626454	-1.2527782
F	-1.5518407	1.2193430	-1.9348464
F	-3.6283832	1.8495083	-2.6688636
H	3.9406278	0.2948294	1.5682149
H	4.9946774	1.6753785	1.9612446
H	3.2358050	1.8110183	2.1608258
H	5.9326645	2.2733165	-0.4502627
H	5.4103880	0.5764900	-0.4320258
H	4.8365923	1.6533137	-1.7068151
H	0.7835969	4.0261258	-1.4709978
H	1.5317850	3.6775808	-3.0480304
H	0.7846808	2.3871768	-2.1144689
H	4.1852081	3.6810803	-2.7945163
H	3.2947019	4.9749109	-1.9756696
H	4.5109482	4.0553700	-1.0877796

(GFA1+2H)$(PF_6)_2$

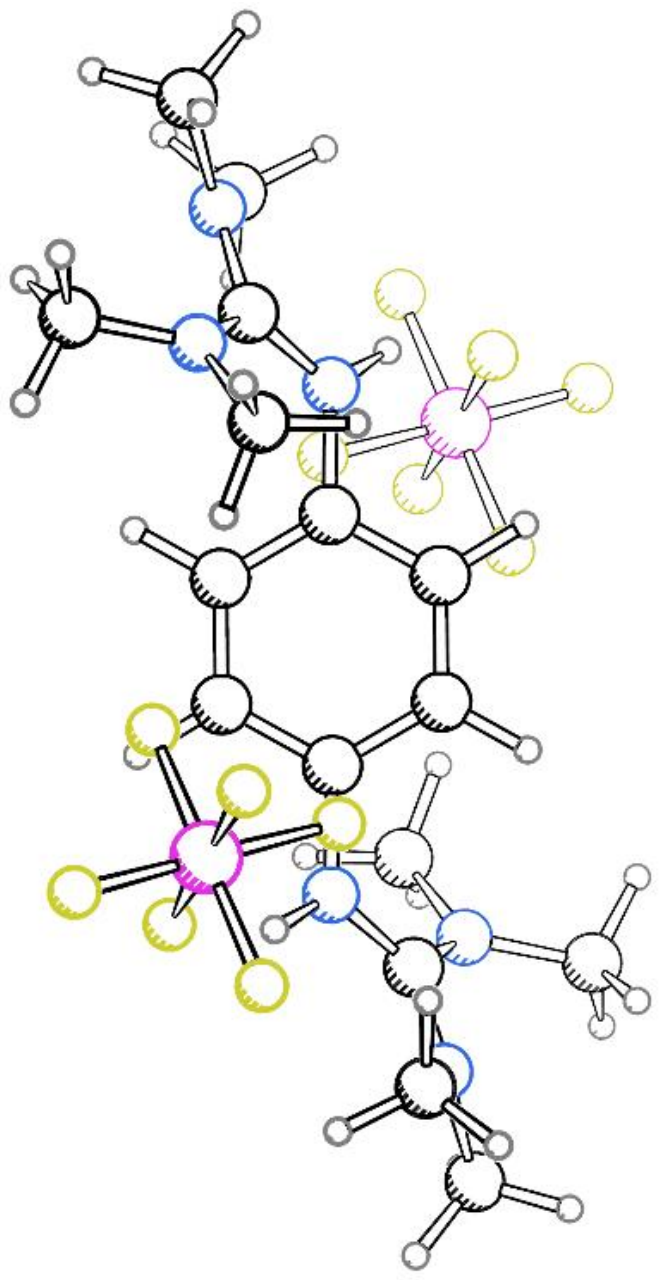

Energy = -2837.498600278

N	-3.4176326	-2.0071943	0.2756583
C	-2.4271704	-2.7205361	0.8465692
C	-3.2704220	-1.3713210	-1.0354259
C	-4.6037888	-1.6082993	1.0236422
N	-1.1514611	-2.4892637	0.5314687
N	-2.7090256	-3.6905934	1.7337132
H	-3.0637673	-0.3027729	-0.9509707
H	-4.2048408	-1.5080000	-1.5815503
H	-2.4666657	-1.8454321	-1.5897968
H	-5.5097535	-2.0503098	0.6033601
H	-4.6967890	-0.5211207	0.9741982
H	-4.5106046	-1.9033686	2.0648851
H	-0.4493248	-3.2044607	0.7305473
C	-0.5981808	-1.2151259	0.2485751
C	-1.7053842	-4.1586414	2.6951390
C	-3.9121041	-4.5058323	1.6271422
F	1.1710381	-3.8583717	0.9533365
C	-1.0386137	-0.0810860	0.9176556
C	0.4436687	-1.1390976	-0.6642253
H	-1.1626522	-5.0278991	2.3181196
H	-2.2205397	-4.4372443	3.6143437
H	-0.9869729	-3.3746451	2.9141206
H	-4.5757306	-4.3506834	2.4805456
H	-3.6222205	-5.5582159	1.6034737
H	-4.4462582	-4.2723235	0.7108359
P	2.1817553	-3.2504131	2.1481641
H	-1.8304632	-0.1515820	1.6503335
C	-0.4436687	1.1390976	0.6642253
C	1.0386137	0.0810860	-0.9176556

H	0.7940320	-2.0347992	-1.1559605
F	1.5421137	-4.3222730	3.1755384
F	2.7399525	-2.1681580	1.0671960
F	3.3018726	-4.3007600	1.6985732
F	0.9881639	-2.1973004	2.5426730
F	3.1214659	-2.6384986	3.2911454
C	0.5981808	1.2151259	-0.2485751
H	-0.7940320	2.0347992	1.1559605
H	1.8304632	0.1515820	-1.6503335
N	1.1514611	2.4892637	-0.5314687
H	0.4493248	3.2044607	-0.7305473
C	2.4271704	2.7205361	-0.8465692
F	-1.1710381	3.8583717	-0.9533365
N	3.4176326	2.0071943	-0.2756583
N	2.7090256	3.6905934	-1.7337132
P	-2.1817553	3.2504131	-2.1481641
C	3.2704220	1.3713210	1.0354259
C	4.6037888	1.6082993	-1.0236422
C	1.7053842	4.1586414	-2.6951390
C	3.9121041	4.5058323	-1.6271422
F	-1.5421137	4.3222730	-3.1755384
F	-2.7399525	2.1681580	-1.0671960
F	-3.3018726	4.3007600	-1.6985732
F	-0.9881639	2.1973004	-2.5426730
F	-3.1214659	2.6384986	-3.2911454
H	3.0637673	0.3027729	0.9509707
H	4.2048408	1.5080000	1.5815503
H	2.4666657	1.8454321	1.5897968
H	5.5097535	2.0503098	-0.6033601
H	4.6967890	0.5211207	-0.9741982
H	4.5106046	1.9033686	-2.0648851
H	1.1626522	5.0278991	-2.3181196
H	2.2205397	4.4372443	-3.6143437
H	0.9869729	3.3746451	-2.9141206
H	4.5757306	4.3506834	-2.4805456
H	3.6222205	5.5582159	-1.6034737
H	4.4462582	4.2723235	-0.7108359

CH_3NO_2

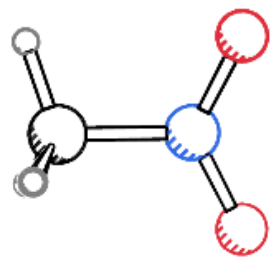

Energy = -245.0074886870

C	-0.9083527	0.8204078	-0.0840318
N	0.4016000	1.1744772	0.5512942

H	-1.3858042	0.0898018	0.5654068
H	-1.5016117	1.7232544	-0.1727104
H	-0.6914686	0.3682731	-1.0477036
O	0.5221583	2.2961497	1.0095450
O	1.2506048	0.3015828	0.5740758

PTQ (K1)

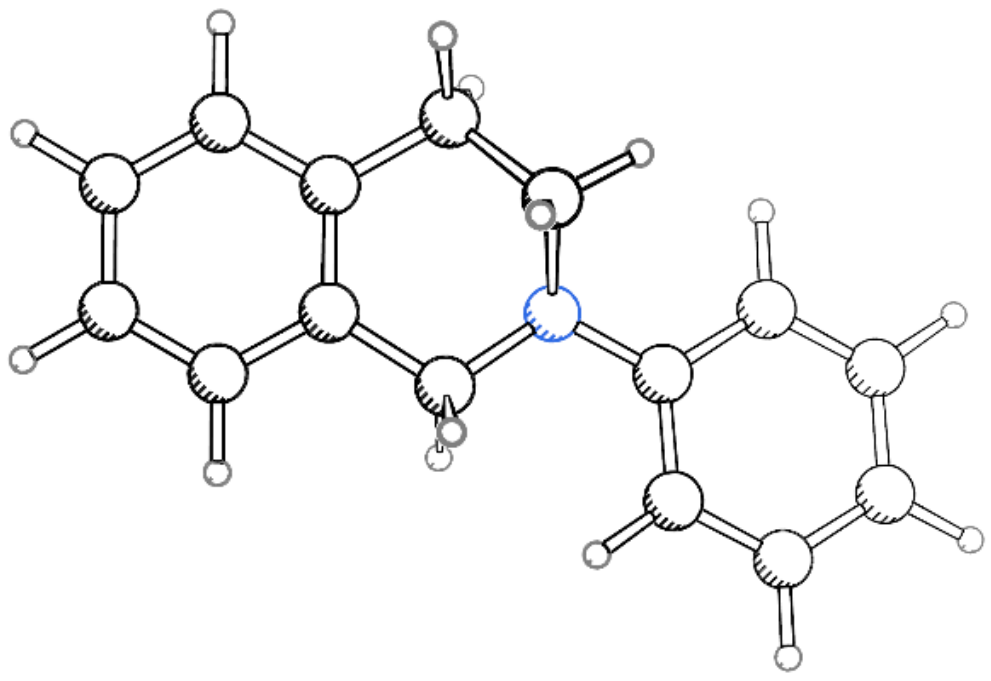

Energy = -635.2656746969

H	-3.9842474	0.8397014	-2.0420554
C	-3.2870808	0.9970809	-1.2290050
C	-3.4975133	0.3868516	0.0042214
C	-2.5971313	0.6002159	1.0361183
C	-1.4790003	1.4155514	0.8582917
C	-1.2676733	2.0303656	-0.3764353
C	-2.1801893	1.8117250	-1.4097757
H	-4.3578067	-0.2517865	0.1583670
H	-2.7584061	0.1248493	1.9974575
H	-2.0160102	2.2936741	-2.3669818
C	-0.0665328	2.9169958	-0.5793628
C	0.4896255	3.3994463	0.7504752
C	-0.5375726	1.6180420	2.0230545
N	0.7019141	2.2622941	1.6391984
H	-0.3352500	3.7728867	-1.2040317
H	0.7147835	2.3637093	-1.1104846
H	1.4251113	3.9288672	0.6023087
H	-0.2135362	4.1110208	1.2131424
H	-1.0637852	2.1918051	2.8072788
H	-0.3023718	0.6434838	2.4542456
H	0.6232587	1.4688244	4.2235727
H	2.4853736	1.4801779	5.7897484
C	1.7396956	2.3098146	2.5761772
C	3.0069764	2.7953201	2.2086834
C	4.0546408	2.8130584	3.1130400
C	3.8858849	2.3408824	4.4121782
C	2.6432843	1.8507774	4.7843332
C	1.5802419	1.8381068	3.8876272

H	3.1817772	3.1311044	1.1960030
H	5.0194483	3.1878471	2.7941593
H	4.7089987	2.3518215	5.1140176

1-(nitromethyl)-2-phenyl-1,2,3,4-tetrahydroisoquinoline (PTQ-CH_2NO_2) (I22)

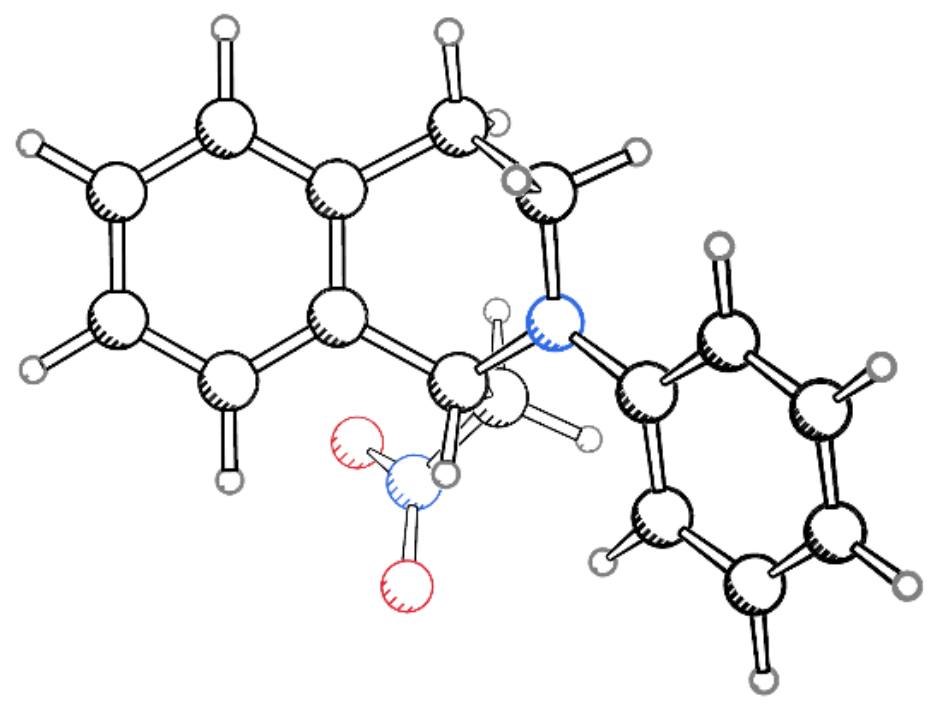

Energy = -879.0834826302

H	-4.7601992	0.3419407	-2.8884012
C	-3.9216207	0.1839456	-2.2224755
C	-4.1412906	-0.1799903	-0.8993071
C	-3.0618899	-0.3731710	-0.0458591
C	-1.7620156	-0.2134285	-0.5157571
C	-1.5389924	0.1494086	-1.8441185
C	-2.6235725	0.3541383	-2.6896973
H	-5.1502136	-0.3081315	-0.5298018
H	-3.2294500	-0.6535225	0.9867883
H	-2.4508461	0.6496416	-3.7178797
C	-0.1124407	0.3322901	-2.2692455
C	0.6784646	1.0535105	-1.1832078
C	-0.5777497	-0.4762847	0.3802726
N	0.5503793	0.4005609	0.1123940
H	-0.0532746	0.9090558	-3.1930721
H	0.3528965	-0.6380144	-2.4745549
H	1.7337618	1.0729149	-1.4628890
H	0.3368078	2.0961283	-1.1258861
H	-0.8982062	-0.3287015	1.4104395
C	-0.1077871	-1.9505803	0.2601914
H	0.6409460	-0.6555268	2.6081382
H	2.1187470	0.0226725	4.3920305
C	1.3860610	0.7806831	1.1571254
C	2.3231265	1.8176310	0.9915286
C	3.1668910	2.1891759	2.0282137
C	3.1121295	1.5544956	3.2622126
C	2.1861464	0.5345846	3.4401438
C	1.3366211	0.1468491	2.4142913
H	2.3871242	2.3537608	0.0576561

H	3.8714699	2.9950858	1.8637498
H	3.7720015	1.8495928	4.0666914
H	0.0565273	-2.2425580	-0.7713461
H	0.7884924	-2.1033308	0.8572800
N	-1.1438434	-2.8794763	0.8145383
O	-1.4319881	-2.7308794	1.9938036
O	-1.6463070	-3.6955436	0.0644791

PTQ-CF_3 (K1)

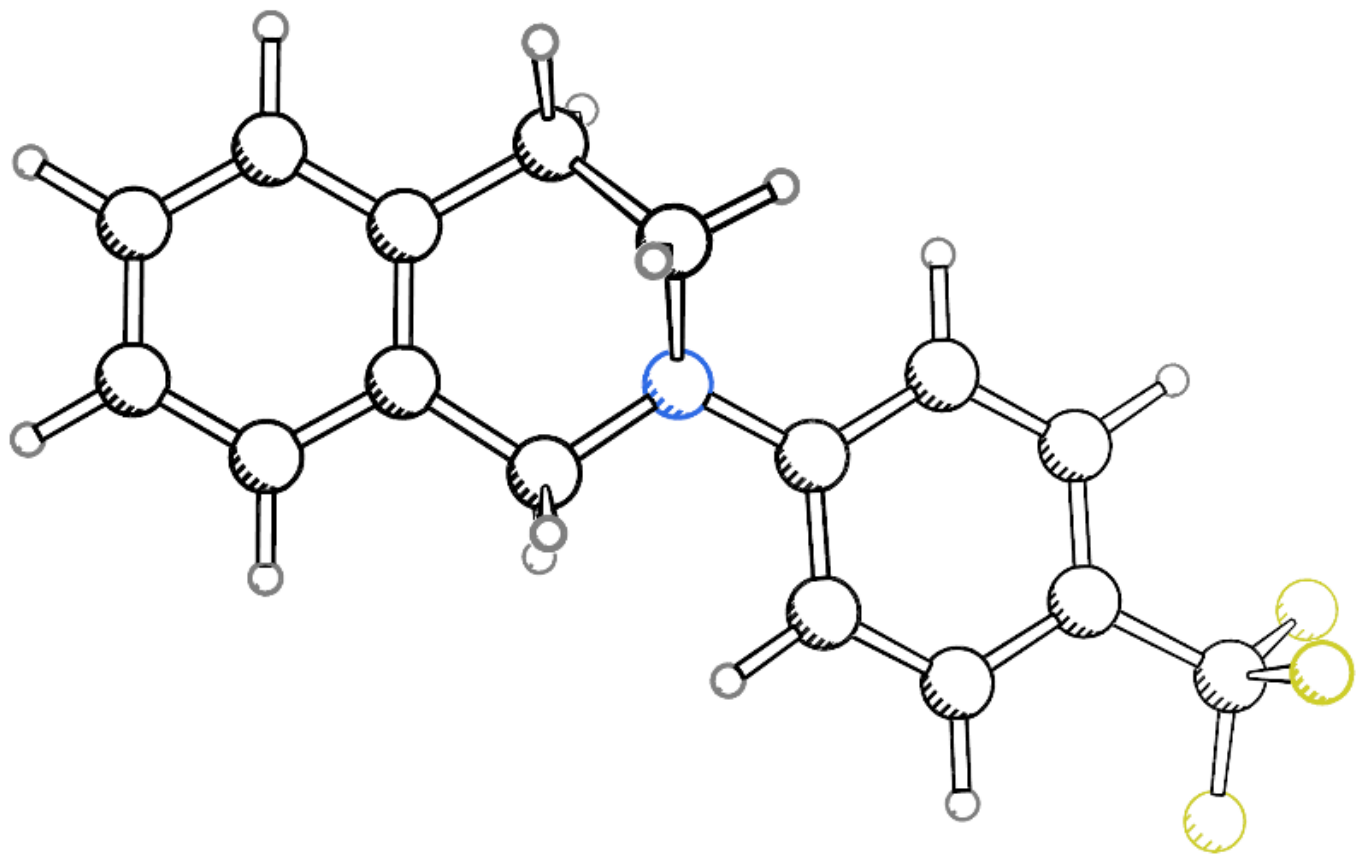

Energy = -972.3470539194

H	-7.1709044	-0.5879849	0.0901478
C	-6.1143835	-0.4024010	0.2349280
C	-5.6015983	-0.2286075	1.5173754
C	-4.2473101	0.0110458	1.6866871
C	-3.3867522	0.0777041	0.5903507
C	-3.8991176	-0.0923511	-0.6959898
C	-5.2647964	-0.3314858	-0.8577581
H	-6.2547776	-0.2804795	2.3788555
H	-3.8458039	0.1456237	2.6850685
H	-5.6613542	-0.4587632	-1.8586253
C	-2.9807457	-0.0102333	-1.8871389
C	-1.7127172	0.7534527	-1.5424677
C	-1.9195863	0.3399966	0.8430546
N	-1.1019505	0.1876115	-0.3450117
H	-3.4925352	0.4765739	-2.7211047
H	-2.7102296	-1.0176400	-2.2196165
H	-1.0098108	0.7192375	-2.3675525
H	-1.9527513	1.8135001	-1.3683460
H	-1.8059975	1.3476040	1.2787357
H	-1.5694455	-0.3679512	1.5959298
H	0.3205836	0.5211888	1.9204454
H	2.7311141	0.3338128	2.1436624

C	0.2776802	0.1397109	-0.2084679
C	1.1068910	-0.1234088	-1.3176410
C	2.4765023	-0.2142818	-1.1807483
C	3.0812629	-0.0543171	0.0659141
C	2.2804636	0.2022802	1.1696214
C	0.9033588	0.3011994	1.0398754
H	0.6728805	-0.2946755	-2.2915831
H	3.0838481	-0.4314231	-2.0494290
C	4.5703030	-0.1148920	0.1849542
F	4.9796972	-0.3291358	1.4527112
F	5.1040247	-1.0978019	-0.5766410
F	5.1651980	1.0366992	-0.2208837

PTQ-CF_3-CH_2NO_2 (I22K1)

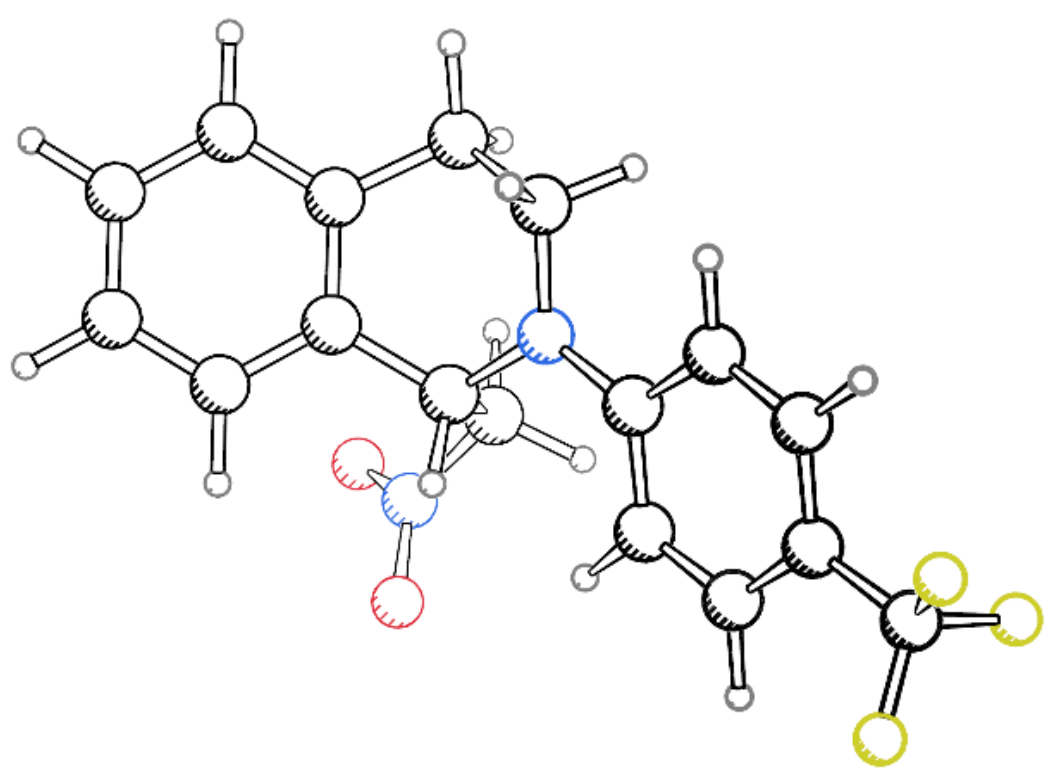

Energy = -1216.164369274

H	2.5604824	5.0209929	-3.0670167
C	2.0576385	4.1572083	-2.6517016
C	0.6918777	4.2012064	-2.3981492
C	0.0518536	3.0930799	-1.8567472
C	0.7740300	1.9368677	-1.5786270
C	2.1443834	1.8892553	-1.8343914
C	2.7814042	3.0059578	-2.3636786
H	0.1260534	5.0974811	-2.6155051
H	-1.0116422	3.1251329	-1.6543404
H	3.8484050	2.9744090	-2.5498590
C	2.8588837	0.6187007	-1.4814411
C	2.3613050	0.0798582	-0.1446224
C	0.0858595	0.7048074	-1.0474230
N	0.9082589	-0.0491113	-0.1110002
H	3.9350908	0.7826566	-1.4167141
H	2.7035541	-0.1369947	-2.2590959
H	2.8040253	-0.9021571	0.0322568
H	2.7040231	0.7445897	0.6589885

H	-0.8201562	1.0230601	-0.5350417
C	-0.3517321	-0.2309421	-2.2032971
H	-1.7365717	-0.5612275	0.1960869
H	-2.7439102	-1.7972675	2.0060995
C	0.3065594	-0.7488649	0.9189174
C	1.0734367	-1.2912859	1.9698256
C	0.4779152	-2.0011048	2.9965603
C	-0.8986923	-2.1954947	3.0257900
C	-1.6709971	-1.6616773	2.0000088
C	-1.0886423	-0.9517003	0.9654949
H	2.1408149	-1.1425903	2.0028419
H	1.0946766	-2.3957351	3.7926324
C	-1.5284345	-3.0091011	4.1112623
H	0.4614392	-0.4414808	-2.8893964
H	-0.7708121	-1.1513474	-1.8022099
N	-1.4437999	0.4083722	-3.0070137
O	-1.2406148	0.6195987	-4.1873059
O	-2.4669376	0.6935856	-2.4011849
F	-2.8044648	-2.6407187	4.3542666
F	-1.5663788	-4.3316182	3.8046599
F	-0.8559691	-2.9125608	5.2792093

PTQ-OMe (K1)

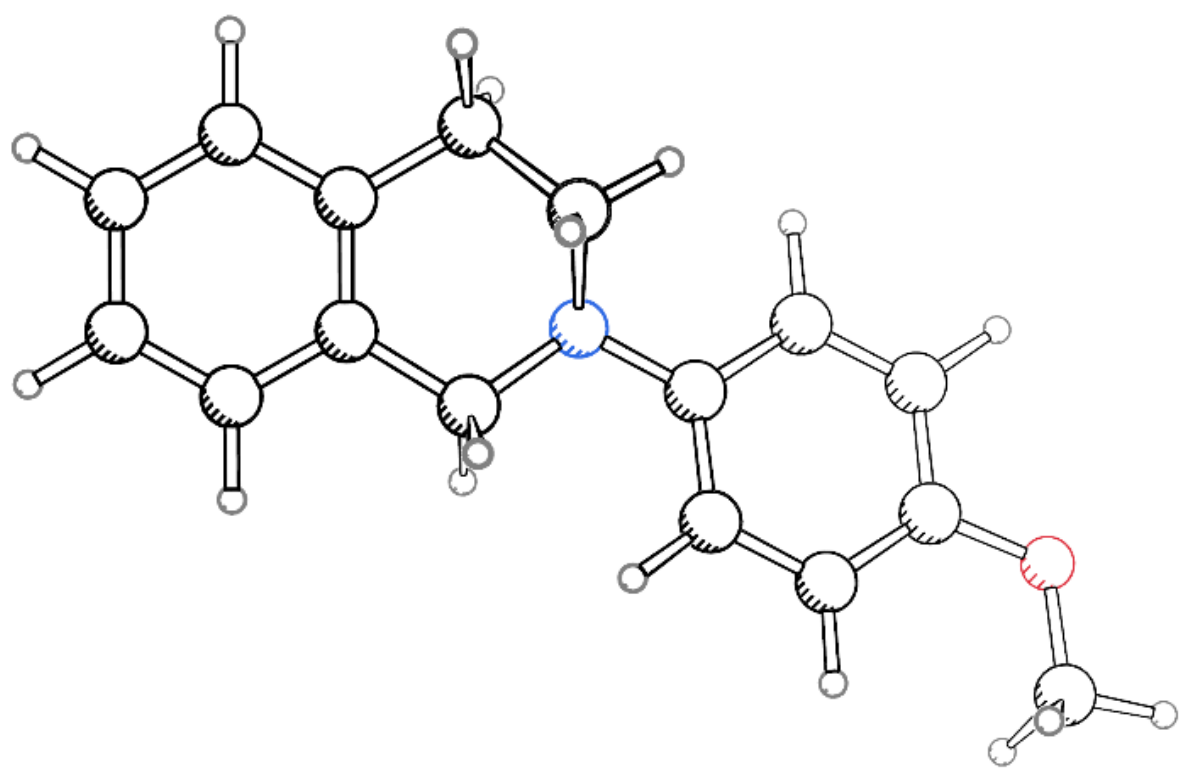

Energy = -749.7807248910

H	-4.9686567	-1.0275057	-3.9564175
C	-4.2524520	-0.9149925	-3.1524695
C	-4.4521622	-1.5655389	-1.9383122
C	-3.5269361	-1.4095896	-0.9179260
C	-2.3949063	-0.6124193	-1.0887618
C	-2.1935634	0.0420391	-2.3048255
C	-3.1308896	-0.1188579	-3.3263951
H	-5.3231771	-2.1909817	-1.7900925
H	-3.6794782	-1.9165054	0.0286105
H	-2.9742679	0.3931255	-4.2692203

C	-0.9736371	0.9040894	-2.5037273
C	-0.3586735	1.3089969	-1.1736771
C	-1.4286210	-0.4621001	0.0625981
N	-0.1678487	0.1257734	-0.3397256
H	-1.2357610	1.7950047	-3.0805324
H	-0.2272504	0.3530646	-3.0850301
H	0.5968196	1.8005705	-1.3313428
H	-1.0138307	2.0337366	-0.6614502
H	-1.9187731	0.1346972	0.8544835
H	-1.2311552	-1.4466679	0.4910137
H	-0.3003184	-0.5208380	2.2980393
H	1.5364551	-0.5460306	3.8748708
C	0.8645348	0.1551412	0.6169476
C	2.1633692	0.5332766	0.2330281
C	3.2067826	0.5412611	1.1344514
C	3.0039804	0.1542739	2.4620760
C	1.7331883	-0.2376532	2.8585323
C	0.6769291	-0.2274653	1.9450411
H	2.3611877	0.7983348	-0.7968512
H	4.2028003	0.8289330	0.8241183
O	4.1026356	0.1882226	3.2736493
C	3.9390820	-0.1978019	4.6246906
H	4.9183429	-0.1004535	5.0881403
H	3.2268037	0.4520134	5.1435464
H	3.6014198	-1.2361491	4.7065176

PTQ-OMe-CH_2NO_2 (I22K1)

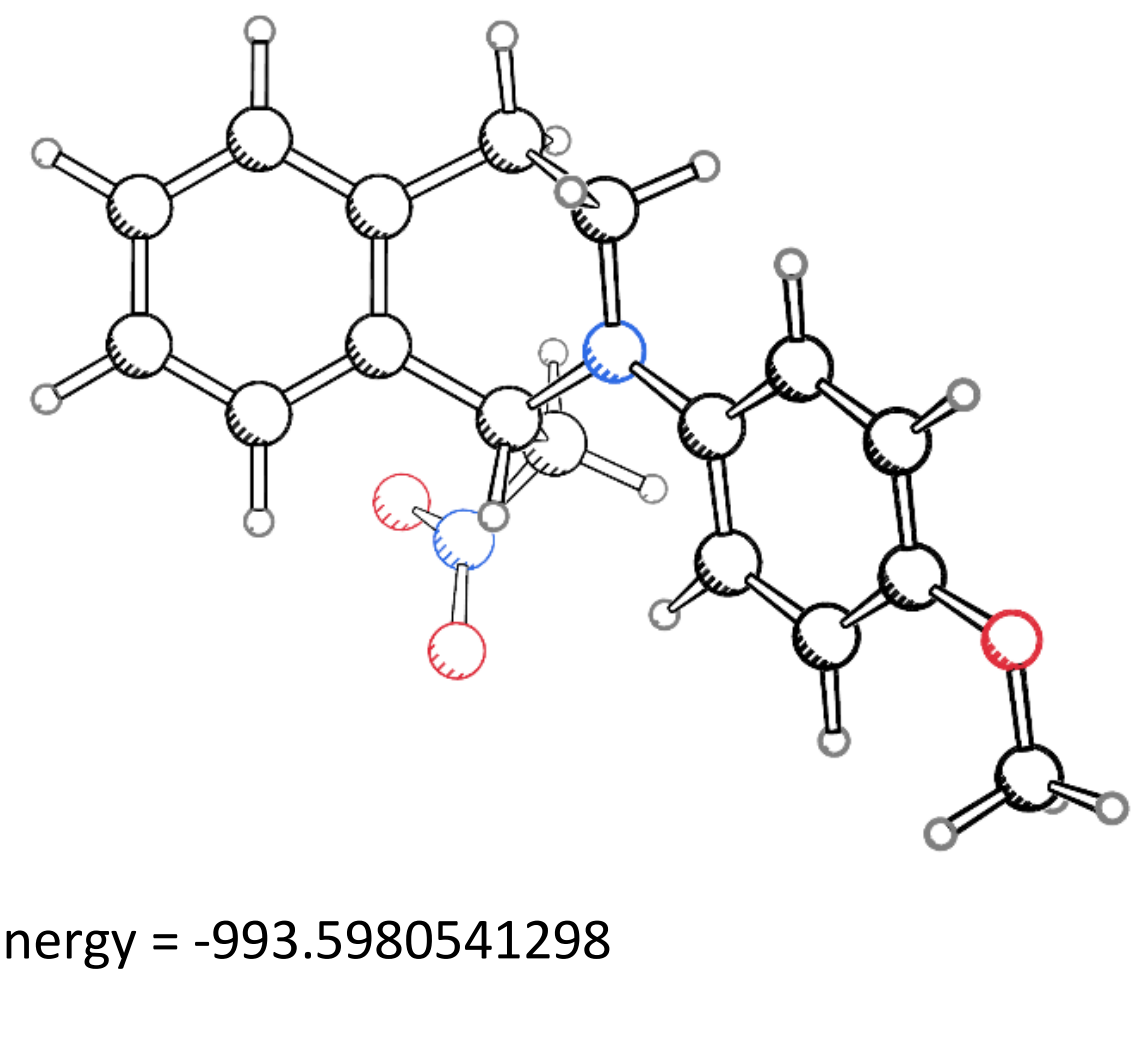

Energy = -993.5980541298

H	3.6434159	0.3238072	-5.1690026
C	3.3453339	0.1911162	-4.1369139
C	4.0921697	-0.6242167	-3.2949057
C	3.7003885	-0.7982485	-1.9731983
C	2.5686583	-0.1502452	-1.4879036
C	1.8186954	0.6704429	-2.3306424

C	2.2086205	0.8302544	-3.6557550
H	4.9746412	-1.1283495	-3.6664173
H	4.2780136	-1.4360051	-1.3153410
H	1.6182442	1.4567239	-4.3140591
C	0.5950969	1.3111879	-1.7459836
C	-0.1455501	0.3236035	-0.8510000
C	2.1632753	-0.2820855	-0.0414180
N	0.7266087	-0.2529046	0.1601863
H	-0.0781271	1.6525718	-2.5333515
H	0.8695217	2.1957335	-1.1606158
H	-0.9671007	0.8418774	-0.3517092
H	-0.5898161	-0.4624385	-1.4783330
H	2.5507717	-1.2303160	0.3302768
C	2.8126291	0.8373291	0.8189218
H	2.0270536	-1.4176607	2.2410852
H	1.0430965	-2.6779832	4.0282169
C	0.1705794	-0.9967170	1.2033016
C	-1.2173818	-1.2227719	1.2736116
C	-1.7777831	-1.9461579	2.3102243
C	-0.9848684	-2.4826751	3.3222786
C	0.3888224	-2.2783713	3.2674134
C	0.9552781	-1.5466304	2.2270848
H	-1.8737539	-0.8471288	0.5041583
H	-2.8464609	-2.1134079	2.3434151
O	-1.6351782	-3.1807905	4.3005900
H	2.6434245	1.8220706	0.3968700
H	2.4416258	0.7790441	1.8396020
N	4.2949518	0.6467896	0.8999088
O	5.0147259	1.5120617	0.4363366
O	4.6817346	-0.3962512	1.4095035
C	-0.8568101	-3.7454627	5.3385632
H	-1.5561852	-4.2518640	6.0000216
H	-0.1361515	-4.4719933	4.9492576
H	-0.3198291	-2.9750566	5.9015534

6 References

[1] U. Jahn, P. Hartmann, I. Dix, P.G. Jones, *Eur. J. Org. Chem.* **2001**, *17*, 3333–3355.

[2] A. Peters, E. Kaifer, H.-J. Himmel, *Eur. J. Org. Chem.* **2008**, 5907–5914.

[3] J. Hornung, O. Hübner, E. Kaifer, H.-J. Himmel, *RSC Adv.* **2016**, *6*, 39323–39329.

[4] Z. Li, C.-J. Li, *J. Am. Chem. Soc.* **2005**, *127*, 6968–6969.

[5] F. Unglaube, P. Hünemörder, X. Guo, Z. Chen, D. Wang, E. Mejía, *Helv. Chim. Acta* **2020**, *103*, e2000184 (2-15).

[6] A. S.-K. Tsang, P. Jensen, J. M. Hook, A. S. K. Hashmi, M. H. Todd, *Pure Appl. Chem.* **2011**, *83*, 655–665.

[7] M. G. Martin, L. R. Clavero, S. E. Martín, P. M. Uberman, M. E. Budén, *Eur. J. Org. Chem.* **2025**, *28*, e202401003 (1-8).

[8] Q. Liu, Y.-N. Li, H.-H. Zhang, B. Chen, C.-H. Tung, L.-Z. Wu, *Chem. Eur. J.* **2012**, *18*, 620–627.

[9] H. Miyake, H. Iida, *Adv. Synth. Catal.* **2024**, *366*, 402–407.

[10] G. Zhao, C. Yang, L. Guo, H. Sun, C. Chen, W. Xia, *Chem. Commun.* **2012**, *48*, 2337–2339.

[11] TURBOMOLE V7.7 2022, a development of University of Karlsruhe and Forschungszentrum Karlsruhe GmbH, 1989-2007, TURBOMOLE GmbH, since 2007; available from https://www.turbomole.org.

[12] S. G. Balasubramani, G. P. Chen, S. Coriani, M. Diedenhofen, M. S. Frank, Y. J. Franzke, F. Furche, R. Grotjahn, M. E. Harding, C. Hättig, A. Hellweg, B. Helmich-Paris, C. Holzer, U. Huniar, M. Kaupp, A. Marefat Khah, S. Karbalaei Khani, T. Müller, F. Mack, B. D. Nguyen, S. M. Parker, E. Perlt, D. Rappoport, K. Reiter, S. Roy, M. Rückert, G. Schmitz, M. Sierka, E. Tapavicza, D. P. Tew, C. van Wüllen, V. K. Voora, F. Weigend, A. Wodyński, J. M. Yu, *J. Chem. Phys.* **2020,** *152,* 184107.

[13] R. Ahlrichs, M. Bär, M. Häser, H. Horn, C. Kölmel, *Chem. Phys. Lett.* **1989,** *162,* 165-169.

[14] O. Treutler, R. Ahlrichs, *J. Chem. Phys.* **1995,** *102,* 346–354.

[15] A. D. Becke, *J. Chem. Phys.* **1993,** *98,* 5648–5652.

[16] P. J. Stephens, F. J. Devlin, C. F. Chabalowski, M. J. Frisch, *J. Phys. Chem.* **1994,** *98,* 11623–11627.

[17] F. Weigend, R. Ahlrichs, *Phys. Chem. Chem. Phys.* **2005,** *7,* 3297–3305.

[18] K. Eichkorn, O. Treutler, H. Öhm, M. Häser, R. Ahlrichs, *Chem. Phys. Lett.* **1995,** *242,* 652–660.

[19] F. Weigend, *Phys. Chem. Chem. Phys.* **2006,** *8,* 1057–1065.

[20] S. Grimme, J. Antony, S. Ehrlich, H. Krieg, *J. Chem. Phys.* **2010,** *132,* 154104.

[21] S. Grimme, S. Ehrlich, L. Goerigk, *J. Comput. Chem.* **2011,** *32,* 1456–1465.

[22] P. Deglmann, F. Furche, R. Ahlrichs, *Chem. Phys. Lett.* **2002,** *362,* 511–518.
